# Exosomes isolated from cancer patients’ sera transfer malignant traits and confer the same phenotype of primary tumors to oncosuppressor-mutated cells

**DOI:** 10.1186/s13046-017-0587-0

**Published:** 2017-08-30

**Authors:** Mohamed Abdouh, Dana Hamam, Zu-Hua Gao, Vincenzo Arena, Manuel Arena, Goffredo Orazio Arena

**Affiliations:** 1 Cancer Research Program, McGill University Health Centre-Research Institute, 1001 Decarie Boulevard, Montreal, Quebec, H4A 3J1 Canada; 2 Department of Experimental Surgery, Faculty of Medicine, McGill University, 845 Rue Sherbrooke O, Montreal, Quebec, H3A 0G4 Canada; 3 Department of Pathology, McGill University Health Centre-Research Institute, 1001 Decarie Boulevard, Montreal, Quebec, H4A 3J1 Canada; 4 Department of Obstetrics and Gynecology, Santo Bambino Hospital, via Torre del Vescovo 4, Catania, Italy; 5 Department of Surgical Sciences, Organ Transplantation and Advances Technologies, University of Catania, via Santa Sofia, 84 Catania, Italy; 6 Department of Surgery, McGill University, St. Mary Hospital, 3830 Lacombe Avenue, Montreal, Quebec, H3T 1M5 Canada

**Keywords:** Cancer patients’ serum, Exosomes, Malignant transformation, Horizontal transfer, Tumor suppressor genes, Genometastasis, Phenotypical differentiation

## Abstract

**Background:**

Horizontal transfer of malignant traits from the primary tumor to distant organs, through blood circulating factors, has recently become a thoroughly studied metastatic pathway to explain cancer dissemination. Recently, we reported that oncosuppressor gene-mutated human cells undergo malignant transformation when exposed to cancer patients’ sera. We also observed that oncosuppressor mutated cells would show an increased uptake of cancer-derived exosomes and we suggested that oncosuppressor genes might protect the integrity of the cell genome by blocking integration of cancer-derived exosomes. In the present study, we tested the hypothesis that cancer patients’ sera-derived exosomes might be responsible for the malignant transformation of target cells and that oncosuppressor mutation would promote their increased uptake. We also sought to unveil the mechanisms behind the hypothesized phenomena.

**Methods:**

We used human *BRCA1* knockout (*BRCA1*-KO) fibroblasts as target cells. Cells were treated in vitro with cancer patients’ sera or cancer patients’ sera-derived exosomes. Treated cells were injected into NOD-SCID mice. Immunohistochemical analyses were performed to determine the differentiation state of the xenotransplants. Mass spectrometry analyses of proteins from cancer exosomes and the *BRCA1*-KO fibroblasts’ membrane were performed to investigate possible de novo expression of molecules involved in vesicles uptake. Blocking of the identified molecules in vitro was performed and in vivo experiments were conducted to confirm the role of these molecules in the malignant transformation carried out by cancer-derived exosomes.

**Results:**

Cells treated with exosomes isolated from cancer patients’ sera underwent malignant transformation and formed tumors when transplanted into immunodeficient mice. Histological analyses showed that the tumors were carcinomas that differentiated into the same lineage of the primary tumors of blood donors. Oncosuppressor mutation promoted the de novo expression, on the plasma membrane of target cells, of receptors, responsible for the increased uptake of cancer-derived exosomes. The selective blocking of these receptors inhibited the horizontal transfer of malignant traits.

**Conclusion:**

These findings strengthen the hypothesis that oncogenic factors transferred via circulating cancer exosomes, induce malignant transformation of target cells even at distance. Oncosuppressor genes might protect the integrity of the cell genome by inhibiting the uptake of cancer-derived exosomes.

**Electronic supplementary material:**

The online version of this article (10.1186/s13046-017-0587-0) contains supplementary material, which is available to authorized users.

## Background

Metastasis is the leading cause of cancer-related mortality [1, 2]. It is traditionally described as a process involving detachment of cancer cells, from the primary tumors, travelling in the blood stream, and homing at metastatic sites [3]. This paradigm has been challenged by recent studies reporting that cancer cells-derived factors could either prepare a niche to permit the engraftment of malignant cancer cell in distant organs or predispose target cells, located in distant organs, to their malignant transformation [4–9]. Horizontal transfer of malignant traits was first reported in immortalized mouse fibroblasts exposed to the serum of cancer patients’ plasma and was called “genometastasis” [10–12]. These observations were for the first time validated by our group in human-derived cell lines. We reported that immortalized human embryonic kidney (HEK293) cells as well as oncosuppressor gene-deficient human cells (*BRCA1-*KO fibroblasts) undergo malignant transformation when exposed to cancer patients’ sera [13, 14]. Histopathological analyses of the excised tumors, following injection of the treated HEK293 cells into immunodeficient mice, showed that the types of tumors grown were not dependent from the patient’s type of cancer. The histology confirmed that all tumors were poorly differentiated carcinomas as further attempts to characterize the tumors immunohistochemically, failed to show any more differentiating features [13]. In contrast, when we treated *BRCA1*-KO fibroblasts with sera of patients with colon or pancreatic cancer, histological analyses of the tumors generated, showed that they were poorly differentiated adenocarcinomas with phenotypical characteristics related to the cancers of the blood donor patients, proving, for the first time, that complete metastatic transformation, through horizontal transfer, is possible [14].

Horizontal transfer of malignant traits involves factors (i.e. proteins or nucleic acids) that could be either circulating as free molecules or circulating as exosomes-packed cargo [5, 7, 15–21]. Exosomes form from cellular endosomal compartment under both physiological and pathological conditions [22–24], and contain in their lumen molecules that mirror the content of their cell of origin [25–27]: for instance, exosomes shed by cancer cells contain oncogenic drivers [9, 28–31]. By delivering their cargo into target recipient cells, either by autocrine, paracrine, or endocrine pathways, exosomes affects cells’ functions (i.e. cells growth and clonogenicity) [4, 8, 23, 32, 33].

Following our observation that cells carrying oncosuppressor mutations, display a significantly increased uptake of cancer-derived exosomes, we suggested that cancer-derived exosomes might carry the oncogenic information through the blood and be responsible for the malignant transformation of the target cells. We hypothesized that oncosuppressor genes might protect the integrity of the cell genome not only by repairing DNA damages and controlling cell cycle checkpoints, but also by blocking the uptake of oncogenic traits contained in cancer exosomes and thus preventing cell transformation [14]. In this study, we sought to validate our hypotheses and therefore we aimed to establish if cancer patients’ sera-derived exosomes were responsible for the transfer of malignant traits and determine if a complete phenotypical transformation would be seen with sera from patients with types of cancers other than colon and pancreas. We also attempted to confirm both the role of oncosuppressor genes in cancer-derived exosomes uptake and unveil the mechanism of action behind this hypothesized function.

Cells treated with cancer patients’ sera-derived exosomes underwent malignant transformation as judged by their ability to form tumors in immunodeficient mice. Histological analyses showed that treated cells had changed completely their fate and the growing tumors were adenocarcinomas that differentiated into the same lineage of the primary tumors of blood donors. Oncosuppressor mutation promoted the de novo expression of receptors, on the plasma membrane of target cells, responsible for the increased uptake of cancer-derived exosomes. The selective blocking of these receptors inhibited the horizontal transfer of malignant traits.

## Methods

### Patients’ recruitment and characteristics of cancers

Patients for the current study were recruited form the department of General Surgery at the Royal Victoria Hospital and St-Mary’s Hospital (Montreal, Canada) and underwent a written consent for blood collection in accordance to a protocol approved by the Ethics Committee of our institution (SDR-10-057). Blood samples were collected from both healthy individuals and patients who underwent resection of primary cancer and who were readmitted for metastatic disease treatment (Table 1).

**Table 1:** Clinical features of patients recruited and the phenotype characterization of the xenografts obtained with cancer patients’ serum-treated cells

Cases	Tumor	Xenograft Phenotype (Pathology Report)	Differentiation Markers	Result
Case219 Case100216 Case272 Case274	CRC-LM CRC-LM CRC-LM CRC-LM	Convincing differentiation toward intestinal adenocarcinoma	CK7 CEA, CK20, CDX-2, and AE1/AE3	Negative positive
Case241 Case271	HCC HCC	HCC differentiation is pretty convincing	AFP, CK8/18, and CEA Hep par-1	positive negative
Case290116	PC	Differentiation toward typical pancreatic ductal adenocarcinoma	CK7, CK19, and AE1/AE3	positive
Case343	OC	Ovarian cancer differentiation can be appreciated	Pax-8 CK20, WT-1, p53, and EMA	negative positive
Case231Exo Case262Exo	CRC-LM CRC-LM	Convincing differentiation toward intestinal adenocarcinoma	CK7 CEA, CK20, CDX-2, and AE1/AE3	negative positive
Case348Exo Case247Exo	HCC HCC	HCC differentiation can be appreciated although Hep par-1 is negative	AFP, CK8/18, and CEA (canalicular pattern) Hep par-1	positive negative
Case290116Exo	PC	Differentiation toward typical pancreatic ductal adenocarcinoma	CK7, CK19, and AE1/AE3	positive
Case343Exo	OC	Ovarian cancer differentiation can be appreciated	Pax-8 CK20, WT-1, p53, and EMA	negative positive

*CRC* Colorectal cancer, *HCC* Hepatocellular carcinoma, *PC* Pancreatic cancer, *OC* Ovarian cancer, *LM* Liver metastasis

Upper part of table: data obtained with whole serum. Lower part of table: data obtained with serum-isolated exosomes

### Blood collection and serum preparation from cancer patients and healthy subjects

Blood samples (20 ml) were collected from a peripheral vein in vacutainer tubes (Becton Dickinson) containing clot-activation additive and a barrier gel to isolate serum. Blood samples were incubated for 60 min at room temperature to allow clotting and subsequently were centrifuged at 1500 x g for 15 min. Serum was collected and a second centrifugation was performed on the serum at 2000 x g for 10 min, to clear it from any contaminating cells. Serum samples were aliquoted and stored at −80 °C until use.

### Cell line and culture conditions

We used the CRISPR/Cas9 system to establish a stable *BRCA1*-KO in human fibroblasts as previously described [14]. Cells were maintained as per supplier’s recommendations. When cells reached 30% confluence, they were treated with DMEM-F12 medium (Wisent, Saint-Bruno, Canada) supplemented with antibiotics and 10% cancer patient sera or control sera, which had been filtered through 0.2 μm filters. Otherwise, cells were exposed to an exosome load of 25–40 μg/ml (which corresponded to 2.6–4.1e + 07 particles/ml of culture medium. Cells were maintained in these conditions at 37 °C in humidified atmosphere containing 95% air and 5% CO_2_ with medium change every second day for 3 weeks. When cells reached 80–90% confluence, they were passaged 1 in 6 using 0.05% Trypsin-EDTA (Wisent, Saint-Bruno, Canada). To confirm that there was no contamination or carry-over of cells from human serum, aliquots of the culture medium were placed in a culture plate and incubated at 37 °C, 5% CO_2_ for 4 weeks.

### Exosomes isolation and labeling

Exosomes were isolated from serum using the Total Exosome Isolation Kit according to the manufacturer’s protocol (Invitrogen, Burlington, Canada). Exosomes were labeled using the PKH26 dye following the manufacturer recommendations (Sigma, Oakville, Canada). Labeled exosomes were diluted in labeling stop solution (PBS/FBS) and pelleted by ultra-centrifugation for 80 min at 100,000 x g at 4 °C. The pellet was washed in Hank’s Balanced Salt Solution (HBSS) with an ultra-centrifugation using the same parameters. The pelleted exosomes were re-suspended in HBSS and stored at −80 °C. 10 μg of labeled exosomes was added to cells (~5 × 10^3^) maintained in 8-well chamber slides (VWR, Mont-Royal, Canada). Cells were washed, fixed for 10 min with Paraformaldehyde 4%. Slides were mounted with coverslip in VECTASHIELD Mounting Medium with DAPI (Vector Laboratories, Burlington, Canada). Stained cells were visualized using an LSM780 confocal microscope (Zeiss, Toronto, Canada). Exosomes internalization was quantified using ImageJ software. Where mentioned, a batch of cells was also analyzed by flow cytometry. Cells were acquired using a FACSCalibur flow cytometer (Becton-Dickinson) at a flow rate of ~300 cells/s. Dead cells and cell debris were excluded from acquisition by gating intact cells on a FCS and SSC biparametric plot.

### Exosomes characterization

Morphological examination of isolated exosomes was done using transmission electron microscope (JEM-2010, Jeol Ltd., Tokyo, Japan). Briefly, 20 μl of exosomes were loaded on a copper grid and stained with 2% phosphotungstic acid. Samples were dried by incubating them for 10 min under an electric incandescent lamp. Samples were examined under electron microscope and imaged using a Hitachi H-600 TEM operating at 60 kV. In parallel, an aliquot of exosome samples was run on a Nanosight NS500 system (Nanosight Ltd., Amesbury, UK), and size distribution was analyzed using the NTA 1.3 software.

### Immunoblotting

Cells and pelleted exosomes were lysed in RIPA buffer containing protease inhibitors (Sigma, Oakville, Canada). Equal amounts of proteins were resolved on 10% SDS-PAGE and transferred to a nitrocellulose membrane (BioRad, CA, USA). Membranes were blocked in TBS (20 mM Tris, 150 mM NaCl, pH. 7.6) containing 5% non-fat dry milk and exposed overnight at 4 °C to rabbit-anti-GM130 (ab52649) and mouse-anti-TSG101 (ab83) (Abcam, MA, USA), and mouse-anti-Alix (2127, Cell Signaling, MA, USA). Membranes were washed in TBST (TBS-0.05% Tween-20) and incubated with either anti-rabbit or anti-mouse peroxidase-conjugated secondary antibody for 1 h at room temperature. After several washes in TBST, the blots were developed using Immobilon Western HRP Substrate (Millipore, Etobicoke, Canada).

### Proteins preparation and mass spectrometry

Plasma membranes proteins were enriched from fibroblast lysates (control vs. *BRCA1*-KO) using the Plasma Membrane Protein Extraction Kit (ab65400, Abcam, MA, USA). Proteins were also prepared from pelleted exosomes. Samples were processed for mass spectrometry. Briefly, proteins were run on a stacking gel. The stacking gel bands were reduced with DTT, alkylated with iodoacetic acid and then digested with trypsin with re-solubilization in 0.1% aqueous formic acid/2% acetonitrile. Peptides were loaded onto a Thermo Acclaim Pepmap precolumn (Thermo, 75uM ID X 2 cm C18 3uM beads), and onto an Acclaim Pepmap Easyspray analytical column separation (Thermo, 75uM X 15 cm with C18 2uM beads) using a Dionex Ultimate 3000 uHPLC at 220 nl/min with a gradient of 2–35% organic (0.1% formic acid in acetonitrile) over 4 h. Peptides were analyzed using a Thermo Orbitrap Fusion mass spectrometer operating at 120,000 resolution (FWHM in MS1, 15,000 for MS/MS) with HCD sequencing all peptides with a charge of 2+ or greater. The raw data were converted into MGF format (Mascot Generic Format) searched using Mascot 2.3 against human sequences (Swissprot). The database search results were loaded onto Scaffold Q+ Scaffold_4.7.2 (Proteome Sciences) for spectral counting, statistical treatment and data visualization.

### Exosomes uptake inhibition

Cells were blocked for 1 h. with different receptor antagonists: Anti-β4 Integrin (10 μg/ml, ASC-8, ab77801, abcam), Cytostatin (1.4 μg/ml, 19,602, Cedarlane), and Heparin (10 μg/ml, H3149, Sigma). In parallel, exosomes were treated for 2 h. with RGD (300 nM, 14,501–1, Cedarlane), and Collagenase I (500 μg/ml, C0130, Sigma). Afterwards, cells were washed, mixed with treated exosomes and incubated for 6 h. Exosomes internalization was analyzed as described in previous section.

### In vivo tumor growth

Five-week-old female NOD-SCID mice (Jackson Laboratory) were used in compliance with McGill University Health Centre Animal Compliance Office (Protocol 2012–7280). Cells growing in log phase were harvested by trypsinization and washed twice with HBSS. Mice were injected subcutaneously with 2 million cells in 200 μl HBSS/Matrigel. Mice were euthanized one month post-injection. The resulting xenotransplants were photographed and processed as indicated below.

### Immunohistochemistry labelling procedures and histological analyses

Mice xenotransplants were collected, fixed in 10% buffered formalin, embedded in paraffin, and stained with H&E (hematoxylin and eosin) according to standard protocols or processed for immunohistochemistry. Briefly, 5 μm tissue sections were dewaxed in xylene and rehydrated with distilled water. After antigen unmasking, and blocking of endogenous peroxidase (3% hydrogen peroxide), the slides were incubated with primary antibodies (Additional file 1: Table S1). Labeling was performed using iView DAB Detection Kit (Ventana) on the Ventana automated immunostainer. Sections were counterstained lightly with Hematoxylin before mounting. Histological analyses were performed by a certified pathologist.

### Statistical analysis

Statistical differences were analyzed using Student’s t test for unpaired samples. An ANOVA followed by the Dunnett test was used for multiple comparisons with one control group. The criterion for significance (*p* value) was set as mentioned in figures.

## Results

### Cells treated with cancer patient sera differentiated into the same lineages of the primary cancers.

**Fig. 1:**
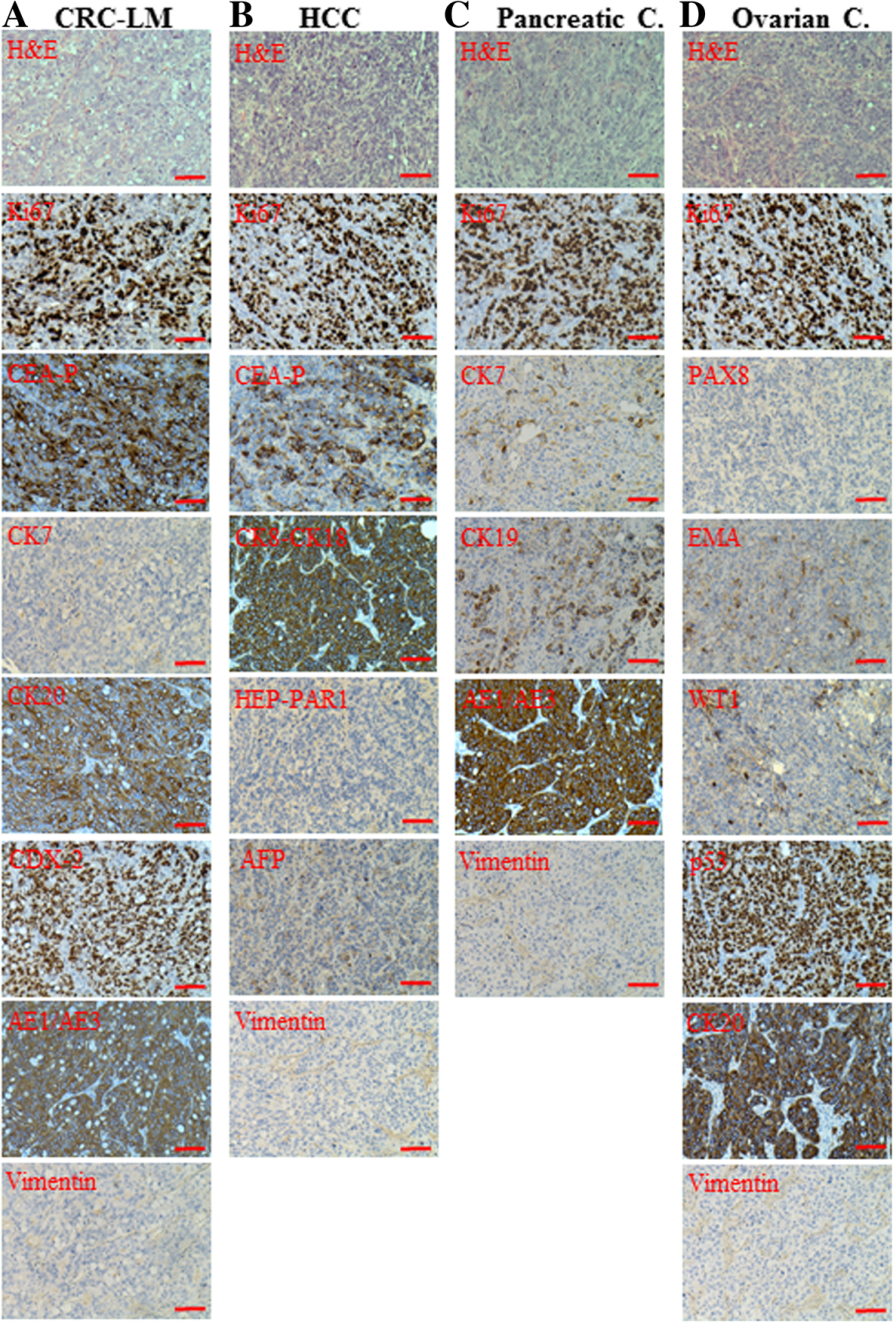
Cancer patient sera-treated cells differentiated into the same lineages of the primary cancers. *BRCA1*-KO fibroblasts were treated for 3 weeks with sera from patients with CRC-LM (**a** Case272), HCC (**b** Case271), Pancreatic cancer (Pancreatic C.; **c** Case290116), or Ovarian cancer (Ovarian C.; **d** Case343). Treated cells were resuspended in HBSS/Matrigel mixture and injected subcutaneously into NOD/SCID mice (*n* = 3 mice per group). Four weeks after cells transplantation, mice were euthanized and the xenotransplants excised. Formalin-fixed paraffin-embedded tumors were processed for H&E staining, or immunolabeled with antibodies against tumor specific markers (Additional file 1: Table S1). Scale bar: 100 μm

For this study, human *BRCA1*-KO fibroblasts were used as target cells. These cells were exposed for three weeks to different types of cancer patients’ sera (Table 1) and healthy patients’ sera and subsequently cells were injected subcutaneously into NOD/SCID mice. Independently of the cancer serum used, mice developed visible tumors as early as one week following inoculation. None of the mice injected with *BRCA1-*KO fibroblasts treated with healthy patients’ sera was found to have grown tumors after the mice were euthanized. Histopathological analyses of developing xenotransplants displayed features of adenocarcinomas (H&E staining) with high proliferation index (80–93% Ki67 positivity) (Fig. 1). These data confirm that cancer patients’ sera harbor signaling factors with tumorigenic capabilities that, once delivered to recipient target cells, are capable to complete the cascade of events that eventually lead the cells to acquire malignant traits [10–14].

We analyzed the expression of specific markers to further characterize these xenotransplants for differentiation patterns based on the primary tumor of the blood donors (Table 1 and Fig. 1). We observed that cancer sera-treated cells had completely changed their fate since all developing tumors stained negative for vimentin, which is normally expressed on fibroblasts (Fig. 1). Notably, xenotransplants obtained with cells exposed to the sera of patients with colorectal cancer, hepatocellular carcinoma, pancreatic cancer, and ovarian cancer displayed phenotypical characteristics of blood donors’ primary tumors (Table 1 and Fig. 1). Tumors, generated with colorectal cancer sera-treated cells, displayed epithelial features typical of colorectal adenocarcinomas, as they all stained negative for CK7, and expressed CEA, CK20, CDX-2 and AE1/AE3, which are universal markers of colorectal cancer (Fig. 1a). Tumors, generated with hepatocellular carcinoma sera-exposed cells, showed convincing hepatocellular carcinoma differentiation, although Hep par-1 staining was negative (Fig. 1b) in keeping with early stages of differentiation [34, 35]. The tumors, generated with cells treated with pancreatic cancer serum, stained strongly positive for CK7 and CK19, which are typical markers of pancreatic adenocarcinoma differentiation. Strikingly enough, different stages of differentiation could be visible in the xenotransplants with areas featuring poorly differentiated pancreatic cancer cells and areas containing cells displaying full differentiation (Fig. 1c). The tumors, produced with ovarian cancer sera-exposed cells, showed convincing differentiation toward ovarian carcinoma (Fig. 1d).

Altogether, these observations showed that treated cells had completely changed their fate and adopted features mimicking those of primary cancers of the blood donors. These findings suggest that cells in target organs, might have the potential to incorporate oncogenic factors delivered from primary cancers, acquire aberrant phenotypical traits identical or similar to the primary cancer and be therefore responsible for metastatic disease.

### Cells treated with exosomes, isolated from cancer patient sera, differentiated into the same lineages of the primary cancers.

**Fig. 2:**
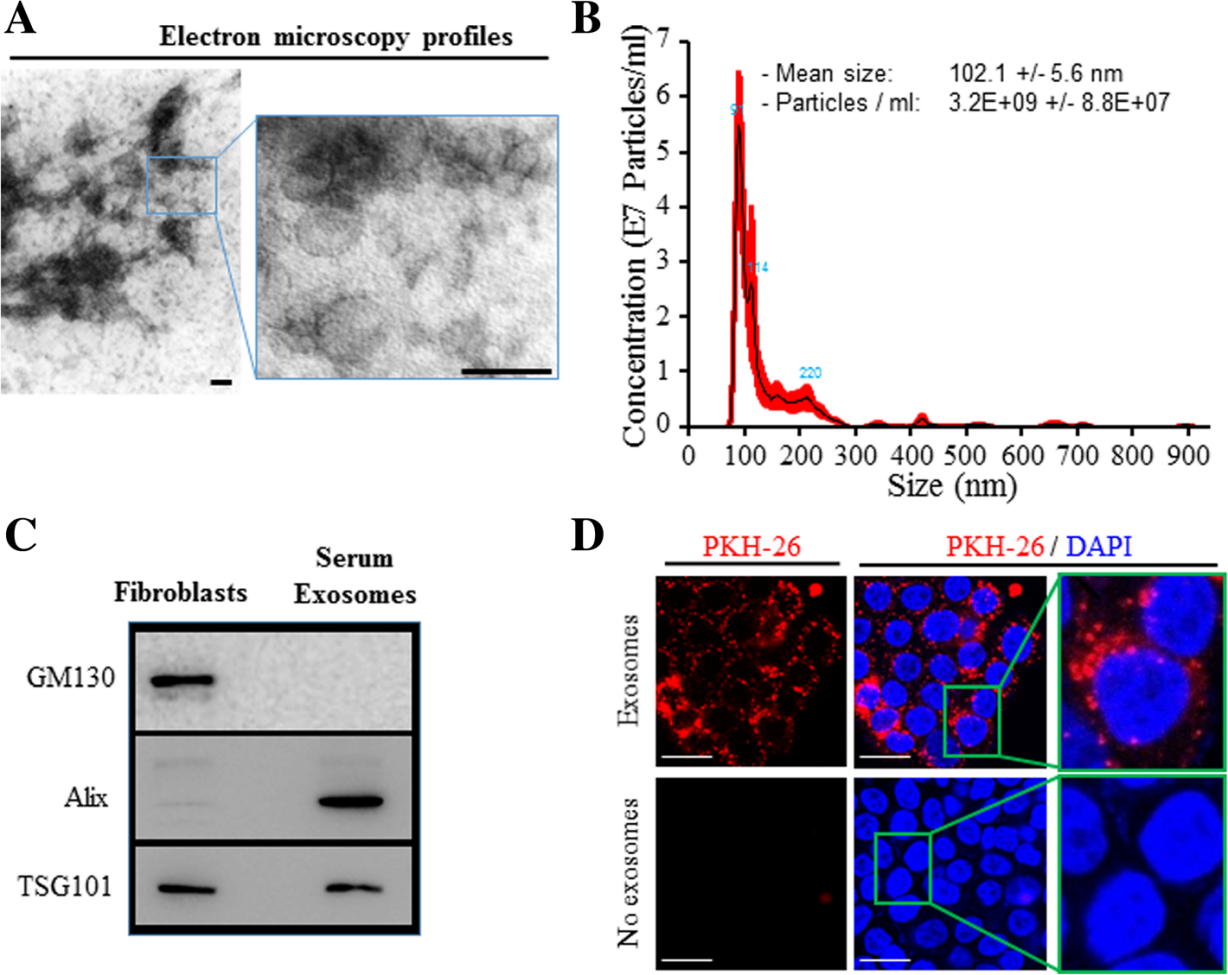
Cancer patient sera-isolated exosomes are efficiently internalized by target cells. **a** Representative micrographs of transmission electron microscopy on cancer patient sera exosome preparations. The image showed small vesicles of approximately 50–120 nm in diameter. Scale bars 100 nm. **b** Nanosight exosomes size analyses. Exosomes size was centered on 91 nm in diameter. Data are expressed as concentration average (black line) +/− standard error (red lines) of six measurements and are representative of 3 exosome preparations. **c** Proteins isolated from fibroblasts, or serum-isolated exosomes were analyzed by Western blot for the expression of specific markers. **d** Confocal microscopy monitoring of PKH-26-labeled exosome uptake in vitro into *BRCA1*-KO fibroblasts. Note that exosomes were dispersed in the cytoplasm and tended to form aggregates in the perinuclear regions. Scale bars 20 μm

It has been shown that exosomes exert different biological effects by transferring their cargo into target cells [14, 36, 37]. Specifically, these microvesicles have been reported to be involved in cancer invasion and metastasis by aiding invasion of cancer cells into the blood stream and conditioning the metastatic niche in target organs [4, 8, 23, 38]. We hypothesized that the malignant transformation we observed on target cells, following cancer patients’ serum exposure, might be due to serum-carried cancer exosomes and therefore we sought to evaluate and confirm the validity of this theory. To test this hypothesis, we isolated exosomes from cancer patients’ sera, and confirmed their identity both physically and phenotypically (Fig. 2). As visualized by electron microscopy, and measured by Nanosight tracking analyses, the isolated entities were rounded structures of 92 to 112 nm in diameter, which is in the range of the expected size for exosomes (102 +/− 6 nm; Fig. 2a, b) [39]. The identity of these exosomes was further characterized by labeling for selective markers that distinguish them from other cellular microvesicles (i.e. Alix, TSG101) and by confirming the absence of other cell organelle markers (i.e. GM130) to rule out contamination with other vesicles or cellular components (Fig. 2c) [39–41]. In order to exert their effect, exosomes must be internalized by target cells and deliver their cargo. To assess exosomes uptake, exosomes were tagged with the membrane dye PKH-26 and were incubated with *BRCA1*-KO fibroblasts. We observed that after 6 h incubation, treated cells efficiently internalized exosomes. Internalized exosomes were uniformly dispersed in the cytoplasm and tended to form aggregates in the perinuclear regions (Fig. 2d).

**Fig. 3:**
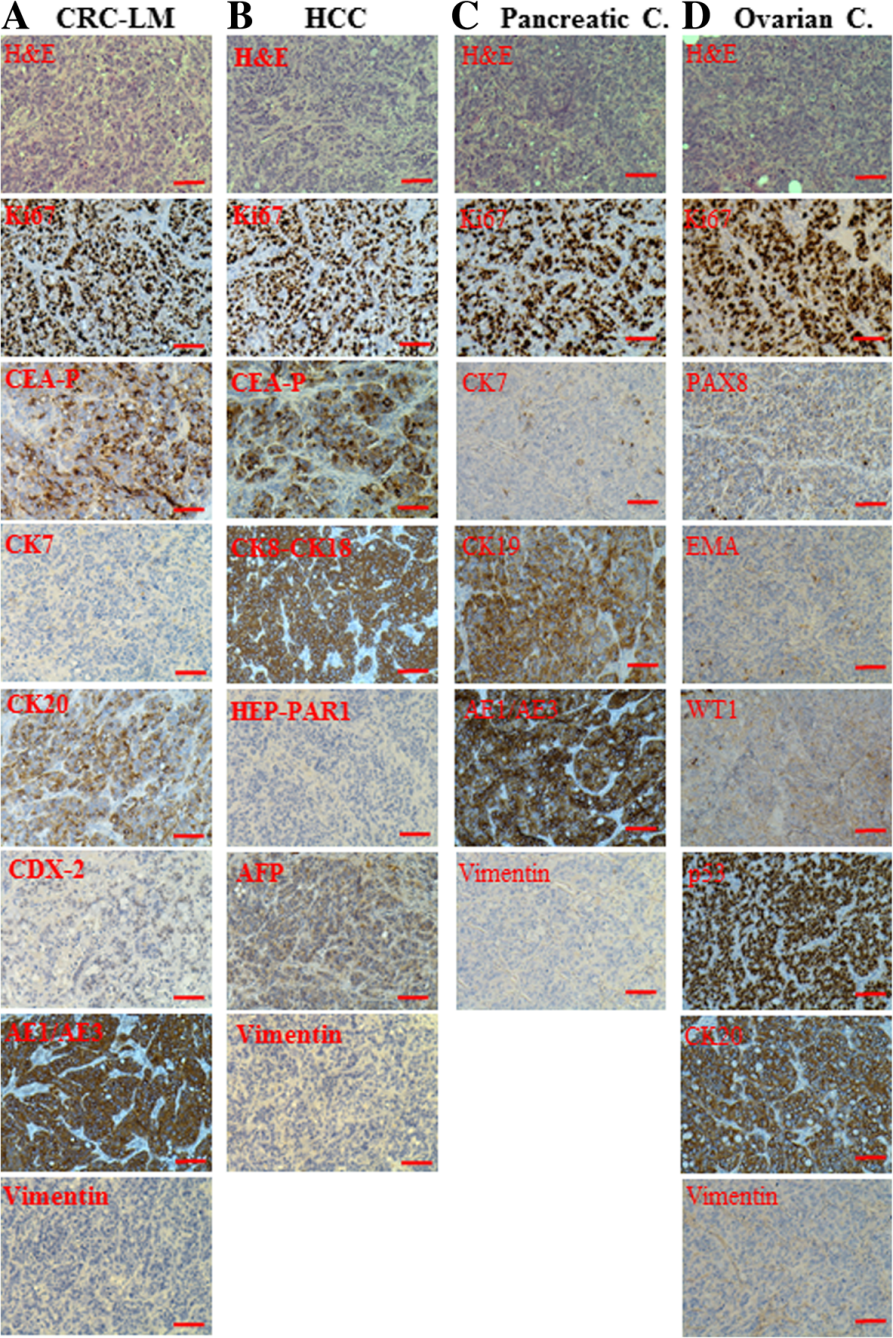
Cells treated with exosomes isolated from cancer patient sera differentiated into the same lineages of the primary cancers. *BRCA1*-KO fibroblasts were treated for 3 weeks with medium containing exosomes isolated from the sera of patients with CRC-LM (**a** Case262Exo), HCC (**b** Case348Exo), Pancreatic cancer (Pancreatic C.; **c** Case290116Exo), or Ovarian cancer (Ovarian C.; **d** Case343Exo). Treated cells were injected subcutaneously into NOD/SCID mice (*n* = 3 mice per group). Four weeks after cells transplantation, mice were euthanized and the xenotransplants excised. Formalin-fixed paraffin-embedded tumors generated following injection of treated cells were processed for H&E staining, or immunolabeled with antibodies against tumor specific markers (Additional file 1: Table S1). Scale bar: 100 μm

We reported previously that sera of both healthy donors and cancer patients had no effects on the behaviour of wild type fibroblasts [14]. Moreover, herein, we observed that when wild type fibroblasts were exposed to exosomes isolated from both types of sera, none of mice injected with these cells developed visible tumors even after longer latency periods following subcutaneous inoculation. Afterwards, to analyze the effects that exosomes isolated from cancer patients’ sera had on the behaviour of exposed *BRCA1*-KO cells, we extracted exosomes from the sera of patients with colorectal cancer, hepatocellular carcinoma, pancreatic cancer and ovarian cancer (Table 1). In parallel, exosomes were also isolated from healthy patients’sera. *BRCA1*-KO fibroblasts were cultured for 3 weeks with cancer exosomes and exosomes isolated from healthy patients. After treatment, the cells were injected subcutaneously into NOD/SCID mice to verify their tumorigenic potential. All mice injected with *BRCA1-*KO fibroblasts treated with cancer exosomes developed tumors. None of the mice injected with *BRCA1-*KO fibroblasts treated with healthy patients’ exosomes, developed tumors. Developing tumors had characteristics of highly proliferative adenocarcinomas (H&E staining and 85–90% Ki67 positivity) (Fig. 3). To determine if these xenotransplants displayed features resembling those of the primary tumors of blood donors, we analyzed the cancer masses for the expression of specific immunohistochemistry markers (Table 1 and Fig. 3). All generated tumors stained negative for vimentin, suggesting that treated cells had completely changed their fate (Fig. 3). Moreover, cells treated with exosomes derived from the sera of patients with colorectal cancer gave rise to tumors that displayed epithelial features typical of colorectal adenocarcinomas (negative for CK7, and positive for CEA, CK20, CDX-2 and AE1/AE3) (Fig. 3a). Also, cells treated with exosomes isolated from the sera of patients with hepatocellular carcinoma generated tumors with patterns of hepatocellular carcinoma differentiation (Fig. 3b). Cells treated with pancreatic cancer serum formed tumors strongly positive for cytokeratins CK19 and AE1/AE3, and with CK7 focal positive patches, reflecting early differentiation into pancreatic cancer (Fig. 3c). The tumors produced with ovarian cancer sera-exposed cells, were weakly positive for WT-1 and EMA suggesting a tendency to differentiate toward ovarian carcinoma (Fig. 3d). These data suggest that exosomes are indeed the main carriers of oncogenic factors through the blood and confirm that their cargo is able to transfer malignant traits at distance.

### Increased exosomes uptake by BRCA1-KO cells is due to de novo expression or overexpression of plasma membrane receptors

**Fig. 4:**
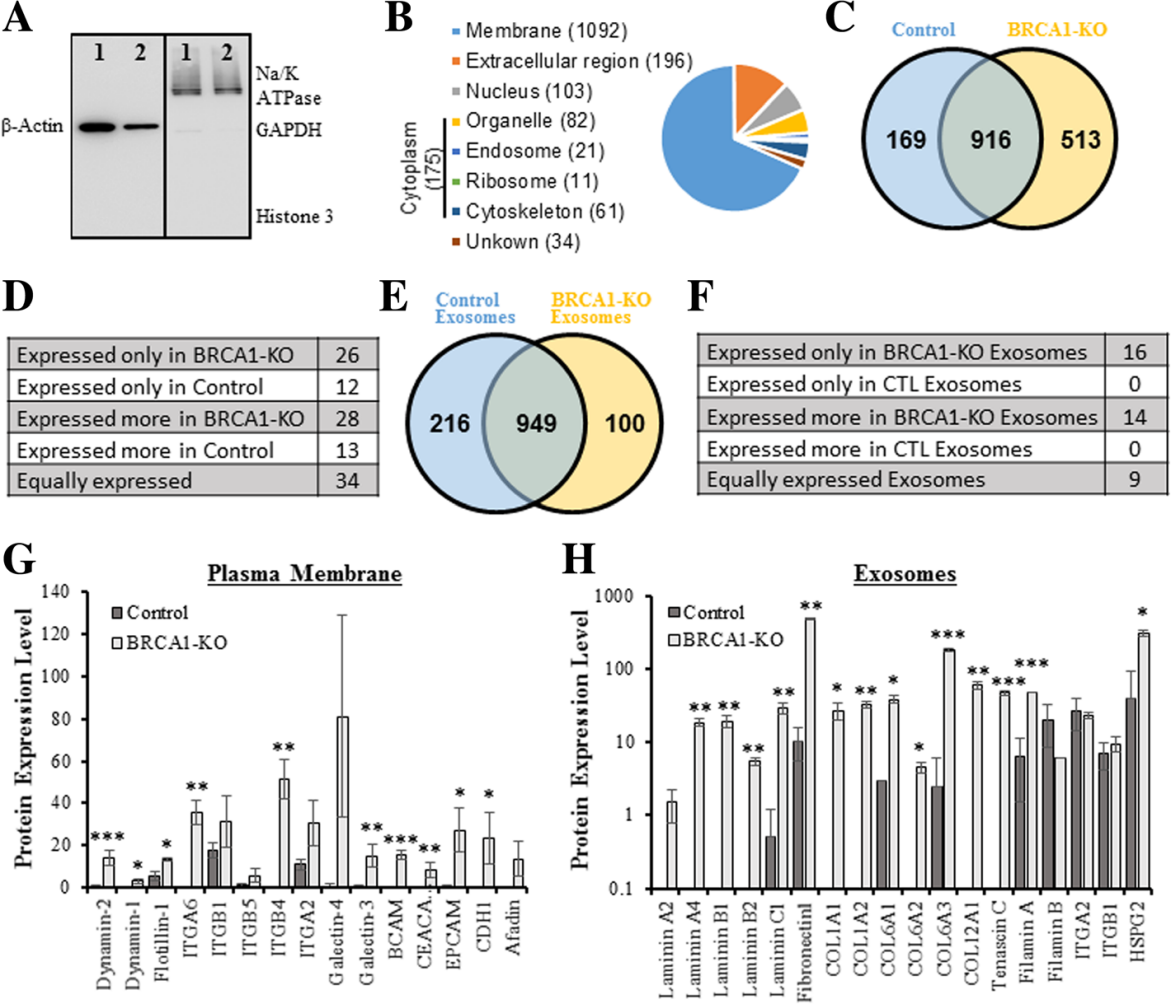
*BRCA1*-KO fibroblasts plasma membrane and cancerous cells-derived exosomes are enriched for proteins involved in vesicles uptake. **a** Control [1] and *BRCA1*-KO [2] fibroblasts were lyzed and enriched for plasma membrane proteins as described under Materials and Methods. Isolated proteins were subjected to an SDS-PAGE to analyze isolation efficiency using an anti-β actin antibody (left panel) and the Plasma Membrane Fraction Western Blot Cocktail (right panel). Note that the plasma membrane-bound sodium/potassium (Na/K) ATPase and β-Actin were highly expressed, while the cytoplasmic GAPDH and nuclear Histone H3 are mildly or not expressed. **b** Protein samples were analyzed by mass spectrometry. We detected 1598 proteins. Gene Ontology (GO) analyses confirmed that the protein isolation from plasma membrane was efficient (1092 proteins are membrane-bound or proteins known to link to membrane proteins), with some contamination from other cell compartments. **c** The Venn diagram shows that there are 916 proteins shared, 169 expressed only in CTL and 513 expressed only in *BRCA1*-KO. **d** The number of proteins putatively involved in vesicles uptake. Note that *BRCA1*-KO fibroblasts express more proteins involved in vesicles uptake when compared to control fibroblasts. **e-f** *BRCA1*-KO and control fibroblasts were treated for 3 weeks with serum from patients with CRC-LM, or serum from healthy donor, respectively. Following 3 weeks of treatment, cells were maintained in exosome-free FBS to collect conditioned media. Exosomes were isolated from the respective conditioned media and subjected to mass spectrometry analyses. **e** Venn diagram: we detected 1265 proteins: 949 were shared proteins, 216 proteins were expressed only in CTL-exosomes and 100 proteins were expressed only in *BRCA1*-KO-exosomes. **f** The number of proteins putatively involved in vesicles uptake. Note that *BRCA1*-KO fibroblasts-derived exosomes express more ligands involved in vesicles uptake when compared to control fibroblasts-derived exosomes. **g-h** Graphs show the top list of proteins expressed in fibroblast plasma membrane (**g**) and exosomes (**h**), and that are involved in vesicles uptake. Data are mean +/− SD ((**g**) *n* = 3 plasma membrane preparations, and (H) *n* = 2 exosomes preparations. *P < 0.05, **P < 0.01, ***P < 0.001). Note that Y axis in H is in log scale

We reported that *BRCA1*-KO cells are more prone to internalize exosomes when compared to normal cells [14]. Herein, we sought to determine the mechanisms behind this phenomenon. We isolated the plasma membranes from *BRCA1*-KO and control fibroblasts using a plasma membrane enrichment kit. This kit enriched for plasma membrane proteins. Western blot analyses of the proteins extracts showed that the plasma membrane isolation was very efficient, as there was enrichment for the transmembrane protein Na/K ATPase and the plasma membrane-bound protein β-actin (Fig. 4a). In contrast, there was a low cytoplasmic proteins contamination, and undetectable nuclear proteins, as judged by the mild expression of GAPDH and absence of Histone H3, respectively (Fig. 4a). The efficiency of plasma membrane enrichment was confirmed following MS analyses. Cellular component GO showed that 1092 proteins were membrane-anchored or proteins known to bind to membrane proteins, while few were from other cellular compartments (i.e. ER, Golgi apparatus, nucleus) (Fig. 4b). Overall, MS detected 1598 proteins: 169 were present only in control fibroblasts, 513 were expressed only in *BRCA1*-KO fibroblasts, and 916 were shared by both cell types (Fig. 4c and Additional file 2: Table S2).

Exosomes uptake involves a group of cell surface proteins that facilitate exosome/cell interactions and subsequent endocytosis [42, 43]. We scanned the MS outputs for proteins involved in exosomes uptake and compared their expression in the plasma membrane purified from *BRCA1*-KO and control fibroblasts. We found that *BRCA1*-KO cells, when compared to control fibroblasts, expressed de novo proteins involved in exosomes uptake such as flotillin, galectin, integrins, dynamin and afadin among others that were overexpressed (Fig. 4d, g, and Additional file 3: Table S3). This observation confirms our hypothesis that oncosuppressor gene mutations might induce expression of proteins on the membrane that would allow cancer exosomes to enter the cell, deliver their cargo and damage the genome [14, 44].

### Transformed cells produced exosomes that overexpress ligands for receptors involved in vesicles internalization

In an attempt to determine whether exosomes shed by cancer cells are more prone than their normal counterpart to internalize into target cells, we isolated exosomes from normal fibroblasts exposed to the serum of healthy individuals (i.e. normal cells) and exosomes from *BRCA1*-KO fibroblasts after treatment with serum from CRC-LM patients (i.e. transformed cells). Cells were first treated for 3 weeks, then conditioned media from both cultures were collected and exosomes were isolated. Isolated exosomes were subjected to MS analyses that showed efficient exosomes isolation. Our MS analyses detected 62% of the top 100 exosomes proteins reported in the Exocarta protein list (http://www.exocarta.org) (Additional file 4: Table S4). Overall, we detected 1265 proteins: 216 were expressed only in control fibroblasts-derived exosomes, 100 were expressed only in *BRCA1*-KO fibroblasts-derived exosomes, and 949 were shared by both exosome populations (Fig. 4e and Additional file 5: Table S5).

We screened our MS data for exosomes-expressed ligands specific to the cell receptors involved in vesicles uptake (as shown in Additional file 3: Table S3). Notably, out of 41 sorted proteins, 39 proteins (96%) overlapped with the Exocarta protein list (Additional file 6: Table S6). Based on differential expression dataset, we found that transformed *BRCA1*-KO cells-derived exosomes overexpressed these ligands when compared to control fibroblasts-derived exosomes. These include laminin (integrins ligands), collagen, tenascin, cadherin, heparan-sulfate proteoglycan and filamin (Fig. 4f, h and Additional file 6: Table S6). Taken together these data show that cancer exosomes express proteins, which enable them to interact with receptors, de novo expressed on the membrane of *BRCA1* mutated fibroblasts. These proteins are either not expressed or under-expressed in exosomes shed by non-cancerous cells.

### Exosomes internalization blockage inhibited target cells transformation

**Fig. 5:**
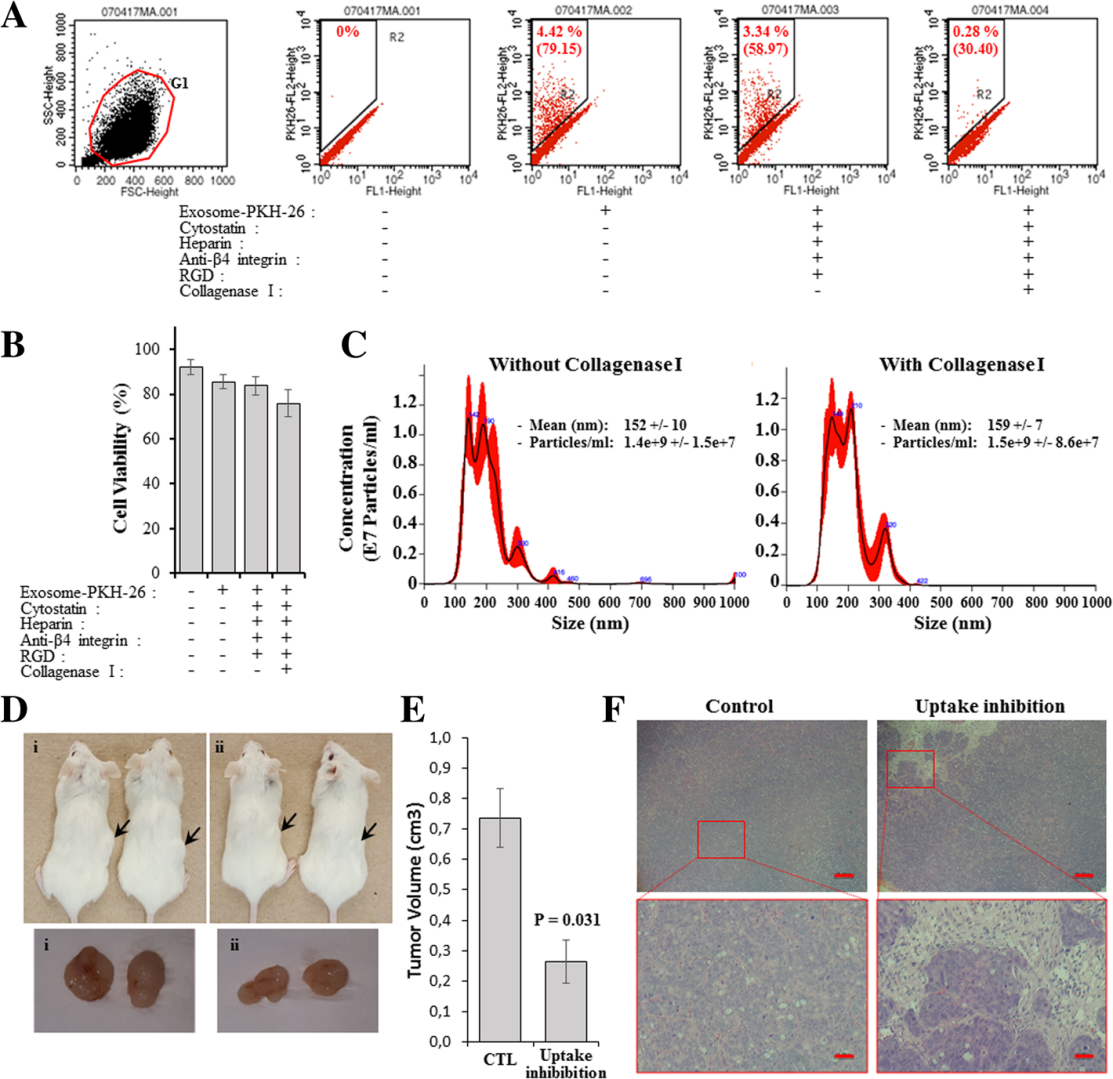
Exosomes internalization blockage inhibited target cells transformation. **a** Exosomes were isolated and labeled with PKH-26. Cells were treated or not with Cytostatin (1.4 μg/ml), Heparin (10 μg/ml) and the anti-β4 integrin antibody (ASC-8; 10 μg/ml). In parallel, exosomes were treated or not with RGD (300 nM) and Collagenase I (500 μg/ml). Cells were exposed to exosomes and analyzed by flow cytometry after gating on cells (G1 population). Data are expressed as the percentage of PKH-26 positive cells. Values in brackets are the mean fluorescence intensity (MFI). Note that antagonists treatments reduced exosomes internalization. **b** Viability of cells treated as in (**a**). Note that treatments slightly affected cell viability. Values are mean +/− SD, (*n* = 3 independent cell cultures). **c** NanoSight analyses of exosomes treated or not with collagenase I. Note that exosome sizes are identical. (D-F) *BRCA1*-KO fibroblasts and exosomes were treated as in (**a**). Cells were washed and mixed with treated exosomes. This treatment was repeated every second day for 2 weeks. Antagonists untreated cells exposed to untreated exosomes served as control. Both cell population were transplanted into NOD/SCID mice. **d** 4 weeks after injection, mice injected with control cells (i) and blocked cells (ii) were photographed and euthanized. Representative pictures of tumors are shown. **e** Tumor volumes at euthanasia. Values are mean +/− SD, (*n* = 2 mice per group), *P* < 0.05. **f** Formalin-fixed paraffin-embedded xenotransplant samples were processed for H&E staining. Note that tumors obtained with treated cells displayed areas of necrosis. Scale bars, 50 μm

To determine if the de novo expressed cell receptors after oncosuppressor mutation (Additional file 3: Table S3) and the newly identified cancer exosome ligands (Additional file 6: Table S6) played a role in the increased cancer exosomes uptake, displayed by *BRCA1*-KO fibroblasts, we used a panel of pharmacological antagonists. For this purpose, *BRCA1*-KO fibroblasts were treated with the anti-β4 integrin-neutralizing antibody (ASC-8), with Cytostatin (an inhibitor of cell adhesion to extracellular matrix; i.e. laminin and collagen) [45], and with heparin (a mimetic of the heparan sulfate in the heparan sulfate proteoglycan) [46]. In parallel, exosomes were exposed to RGD (an integrins tripeptide binding site found within fibronectin), and Collagenase I, before culturing them with the *BRCA1*-KO fibroblasts for 6 h. Non-treated *BRCA1*-KO fibroblasts exposed to non-treated exosomes were used as control. Cells were analyzed by flow cytometry (Fig. 5a). We noted that the percentage of cells that internalized exosomes (i.e. PKH-26 positive cells) dropped by 25% following treatments with all antagonists without collagenase I. Addition of collagenase I to the antagonists cocktail decreased this percentage to 93% (Fig. 5a). Also, when compared to control cells, we observed that the mean fluorescence intensity (MFI) decreased by 1.5 to 2.6 times following treatments with the antagonists (Fig. 5a). This finding suggests that the blocking treatment had decreased both the percentage of cells internalizing the exosomal cargo and the number of exosomes internalized per cell.

To rule-out the possibility that the observed inhibitory effect was due to decreased cell viability or secondary to exosomes damage following antagonist treatments, we performed cell counting following trypan blue labeling and NanoSight analyses of exosomes (Fig. 5b, c). We found that the treatment with antagonists had a feeble effect on cell viability (Fig. 5b), and no effect on exosomes integrity (Fig. 5c). Altogether theses findings suggest that the newly expressed membrane proteins on both *BRCA1*-KO fibroblasts and cancer exosomes might have a role in exosomes uptake in target cells.

To determine if inhibition of the receptors involved in the exosomes uptake may inhibit cancer exosomes-induced cell transformation, we isolated exosomes from the serum of CRC-LM patients. Exosomes uptake was blocked using the same protocol shown in Fig. 5a. Antagonists-treated and non-treated cells were transplanted into NOD/SCID mice to analyze their tumorigenic behavior. Mice were followed up for tumor growth, and they were euthanatized 4 weeks after cell inoculation. Mice injected with non-treated cells displayed visible tumors as early as 10 days following inoculation, which continue growing until euthanasia. In contrast, mice injected with treated cells developed minimal palpable masses (0.73 +/− 0.09 cm^3^ vs. 0.26 +/− 0.07 cm^3^, *P* = 0.031; Fig. 5d, e). Histopathological analyses of tumors generated with non-treated cells showed a uniform sheet of poorly differentiated adenocarcinoma. In contrast, tumors obtained following injection of treated cells displayed zones of poorly differentiated adenocarcinomas with discrete areas of necrotic and non-proliferating cells (Fig. 5f).

### Serum from patients with dysplastic lesions only, was able to transform BRCA1-KO fibroblasts into cancer cells

**Fig. 6:**
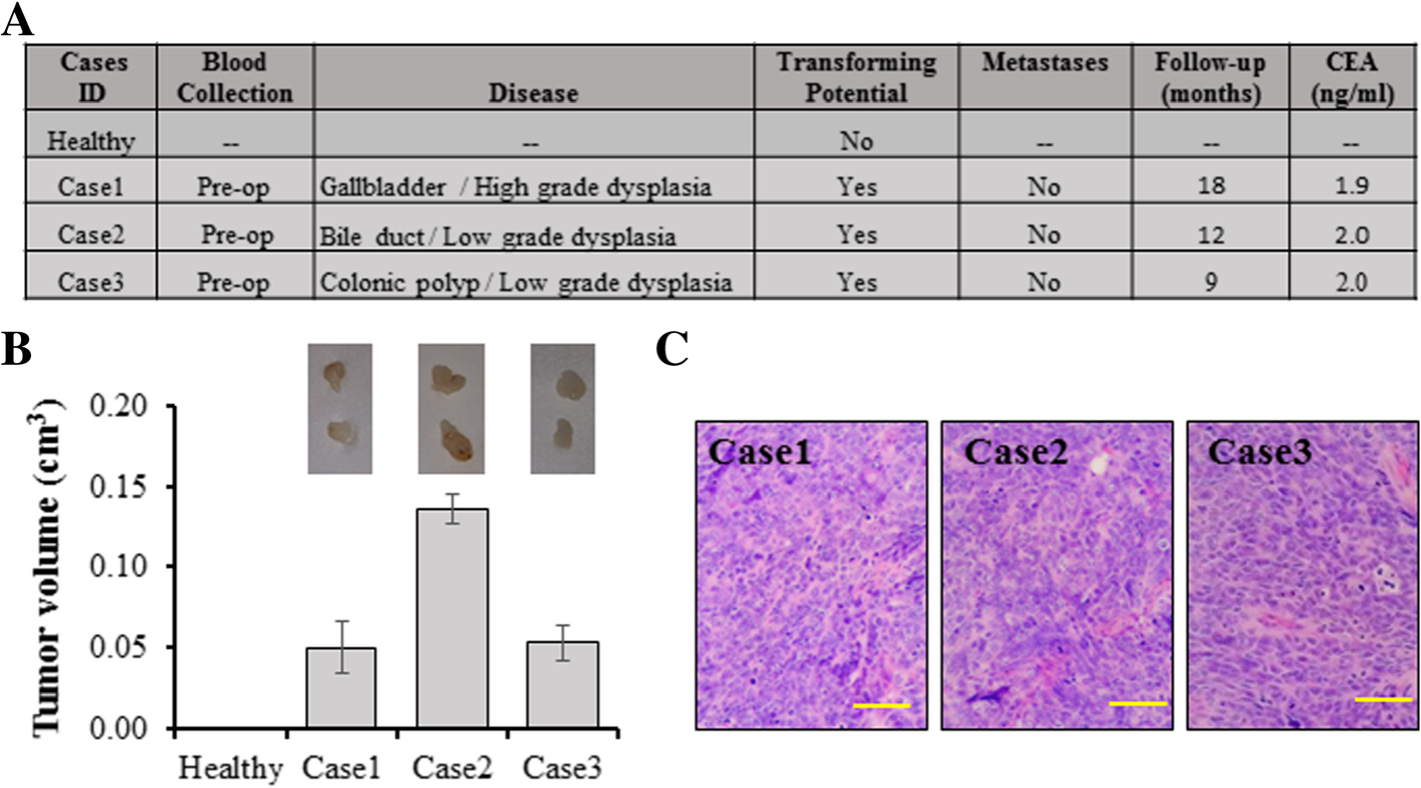
Sera from patients with dysplastic lesions were able to transform *BRCA1*-KO Fibroblasts. **a** Clinical profiles of the patients enrolled in this screening study. **b**-**c** *BRCA1-*KO cells were cultured for three weeks in medium containing the different cases sera. Cells were injected subcutaneously into NOD/SCID mice, which were euthanized four weeks later. **b** Tumor volumes at euthanasia were calculated and values presented as mean +/− SD (*n* = 2 mice per group). Pictures of excised tumors obtained are shown. **c** Formalin-fixed paraffin-embedded xenotransplant samples were processed for H&E staining. Scale bars = 100 μm

We recently reported that *BRCA1-*KO cells were able to “sense” neoplastic factors in the serum of patients with cancers even at early stages and turn malignant regardless of the presence of positive tumor markers (i.e. pancreatic cancer in situ and early colon cancer with negative CEA) [47]. Furthermore, the evidence reported of patients presenting with metastasis years after the resection of dysplastic lesions doesn’t seem to fit in the conventional metastatic model, where cells are supposed to invade the basal membrane in order to metastasize [48]. Herein, we report data obtained with sera from three patients that presented with dysplastic lesions only. The dysplastic lesions were located in the gallbladder, in the colon and in the common bile duct (Fig. 6a). Strikingly enough, the three sera transformed *BRCA1-*KO cells, as confirmed by tumor formation, following transplantation in NOD/SCID mice (Fig. 6b). Histopathological analyses of these tumors indicated that they had histological appearances compatible with gallbladder cancer, colon cancer and bile duct cancer and all masses showed high mitotic index (over 90%, Fig. 6c). Taken together, these data show that dysplastic lesions, which are premalignant lesions, also shed transforming factors that turn *BRCA1-*KO cells into cancer. This finding suggests that the metastatic process might not be secondary to migrating cells since dysplastic lesions, by definition, have not yet invaded the basal membrane. Moreover, this outstanding evidence suggests that horizontal transfer of malignant traits occurs even before cancer cells invasion.

## Discussion

The results obtained in our experiments confirmed that horizontal transfer of malignant traits to target cells is not limited to colon and pancreatic cancer, as we previously demonstrated, but it is a concept applicable also to hepatocellular carcinoma and ovarian cancer. The evidence that *BRCA1*-KO fibroblasts can be reprogrammed and turn into several types of cancers by exposing them to only cancer sera, strengthens the hypothesis that metastasis is a pathological process that might not necessarily require transfer of cells. The evidence, herein demonstrated for the first time, that cancer exosomes are the main effectors of that cascade of events that lead a target cell to a full malignant transformation, is a fascinating discovery, which has important implications. Our data suggest a different role that cancer exosomes might have in the setting of metastatic disease and strengthen the evidence that cancer exosomes may be involved in cancer invasion and metastasis not simply by preparing the niche for the engraftment of circulating cells as it was thought until now [4–9, 23, 42]. Carcinogenesis steps such as initiation, promotion and progression may actually be a process reproducible through horizontal transfer of cancer factors, shed by primary tumors and carried in exosomes through the blood, to susceptible cells located at metastatic sites.

The metastatic process as described in the seed to soil hypothesis is a very inefficient and extraordinarily complex process, which requires a set of features that the cells must acquire to develop new foci of disease in other organs [49]. In order to be able to metastasize, cancer cells must break intercellular junction, invade the basal membrane, acquire migratory capability, incorporate into the lymphatic and blood stream and ultimately home in the parenchyma of target organs [50]. The inefficiency of this process is demonstrated by the evidence, shown in experimental models, that only 0.01% of cancer cells injected into the circulation form metastatic foci [49]. The success of the seed to soil model to explain metastatic disease was nurtured by the evidence that metastatic lesions had immunohistochemical features similar to the cells of the primary tumor and therefore it was obvious to conclude, that metastases were secondary to cells, detaching from the primary tumor, travelling through the blood stream and invading other organs. Our results demonstrate that cancer exosomes can transfer oncogenic factors to cells and determine dramatic changes with acquisition of malignant characteristics and gain of immunohistochemical features similar or identical to the cancer cells that release the exosomes. This model of horizontal transfer offers an easier explanation to unexplained phenomena seen in the conventional model such as genomic differences between primary and metastatic cells [51–54], inefficiency of the process and patterns of metastatic spread. According to this model, exosomes would easily enter the blood stream, would be uptaken by target cells owing to newly expressed receptors and eventually the target cells would change their phenotype according to the type of cancer cell that released the “onco-information”. The metastatic cell would therefore be similar to the primary cancer cell; hence, the genomic differences would be a natural consequence of being alike but not the same cell [49]. In other words, the molecular profiles of primary and metastatic lesions are not usually identical because metastases wouldn’t necessarily derive from cells detached from the primary tumor [51–54].

The full transformation of a fibroblast into such a variety of different cancers after exposure to cancer exosomes proves that the concept of horizontal transfer is scientifically sound and paves the way to a new understanding of the metastatic process, which deserves further study. Although these results are striking, it is still necessary to determine the nature of the exosome-carried factors involved and fully unveil the molecular mechanisms, which underlie the transfer of malignant phenotypical traits. While putative factors (i.e. DNA, mRNA, miRNA, proteins) were already described as serum-derived exosomal cargo, their respective role has not yet been fully defined [4, 5, 8, 9, 15, 16, 22, 28–32, 39] and, in light of the results of this study, further attention to this alternative pathway is certainly warranted.

The observation, already published by our group, that exosomes are uptaken more and faster by oncosuppressor mutated cells, prompted us to verify the hypothesis of a novel function of the oncosuppressor genes. In this model, oncosuppressor genes might protect the cell’s genome, not only by repairing DNA damages and controlling cell cycle checkpoints, but also by inhibiting the uptake of mutating extracellular oncogenic material. In order to confirm this view, we sought to verify if the mutation of the BRCA1 oncosuppressor would trigger some membrane changes, which would lead to an active uptake of cancer exosomes as opposed to passive penetration and selective membrane fusion [43, 49]. The discovery that the knock-out of the oncosuppressor BRCA1 is associated with the de novo expression of proteins already associated with metastasis and aggressiveness such as dynamin [55], integrins [38], galectin [56, 57] and EPCAM [58] is intriguing. According to the conventional theory, these molecules would facilitate cell migration, would promote metastasis and be a hallmark for aggressiveness. In the model that we hypothesized instead, these proteins enable the active uptake of cancer exosomes and their antagonistic blockage would inhibit the malignant transformation at distance and therefore the metastatic event. In other words, these results, once again, strengthen the concept that cell migration is not necessary to explain cancer dissemination and offer novel evidence that the molecules involved in cancer cell dissemination are the same molecules implicated in cancer exosome uptake, malignant cargo delivery and malignant transformation at distance.

In our experiments we used oncosuppressor mutated fibroblasts in the attempt to recreate in vitro the same conditions that characterize carcinogenesis in human beings. As a matter of fact, carcinogenesis is not a sudden process and it requires accumulation of several mutations that cause a normal cell to become first metaplastic, then anaplastic and eventually dysplastic. Therefore, a cell that turns into cancer is never a normal cell but it is a cell already abnormal and with a definite instability, of its genomic asset, that has matured over the years. As we already described [44], we postulate that in cancer patients, multiple or chronic cellular stresses due to several factors (metabolic, viral, environmental, etc.) might cause mutations that would favor the uptake of circulating cancer exosomes in cells located in distant organs with their subsequent malignant transformation.

To further corroborate the validity of this concept and demonstrate that truly cell migration might not be the only model to explain metastasis, we included in this work the exceptional results that were obtained, when we exposed *BRCA1*-KO fibroblasts to sera of patients who had only low and high-grade dysplastic lesions. Dysplastic lesions are precancerous lesions, which, by definition, have not invaded the basal membrane. They are not malignant cells and they have not metastatic potential since one of the hallmarks of cancer is invasion with subsequent ability to metastasize. Contrary to the predictions and dogmas of the conventional metastatic model, some dysplastic lesions have been found to have the capability to metastasize in clinical scenarios, raising suspicion of misdiagnosis or missed cancer lesions. The evidence, shown in this paper, that *BRCA1*-KO fibroblasts turn into gallbladder, colon and bile duct cancer after exposure to sera of patients with only dysplastic lesions is, in our opinion, the definitive proof that the metastatic process might be independent from cell migration and entirely reproducible at distance by malignant transformation mediated through onco-factors circulating in the serum.

## Conclusion

The results of this study demonstrate that blood-circulating exosomes are the major contributing factors involved in the horizontal transfer of malignant traits to target cells. The evidence that BRCA1 mutation triggers the de novo expression or overexpression of proteins and receptors involved in vesicles uptake suggests a novel function that oncosuppressor genes might exhibit to protect the integrity of the genome. The observation that the serum from patients with dysplastic lesions was able to transform target cells strengthens the notion that circulating factors, originating from primary lesions, rather than circulating cells, might be the major players at metastatic sites.

## Additional files

Additional file 1: Table S1. List of antibodies used in this study. (PDF 16 kb)

Additional file 2: Table S2. MS data from the plasma membrane as visualized on Scaffold Q+. (PDF 1967 kb)

Additional file 3: Table S3. Plasma membrane proteins involved in microvesicles uptake*. (PDF 239 kb)

Additional file 4: Table S4. List of top 100 proteins that are often identified in exosomes (ExoCarta). (PDF 158 kb)

Additional file 5: Table S5. MS data from the exosomes as visualized on Scaffold Q+. (PDF 1267 kb)

Additional file 6: Table S6. Exosome proteins involved in microvesicles uptake (Ligands for the receptors determined by MS on fibroblasts plasma membrane)*. (PDF 216 kb)
