# Supplementary material for: Exosomes isolated from cancer patients’ sera transfer malignant traits and confer the same phenotype of primary tumors to oncosuppressor-mutated cells
Source: J Exp Clin Cancer Res. 2017 Aug 30;36:113. doi: 10.1186/s13046-017-0587-0 (PMC5577828; doi:10.1186/s13046-017-0587-0)
Supplement: Supplementary file 1 — List of antibodies used in this study. (PDF 16 kb) [file 13046_2017_587_MOESM1_ESM.pdf]

Supplementary Table1. List of antibodies used in this study.

Antibodies ID	Species	Manufacturer	Dilutions used
Ki67	Rabbit Monoclonal	Ventana (USA)	Dispenser (Pre-diluted)
CEA-P	Rabbit Polyclonal	DAKO (Denmark)	1:5000
CK7	Mouse Monoclonal	DAKO (Denmark)	1:2000
CK20	Mouse Monoclonal	DAKO (Denmark)	1:1000
CDX-2	Rabbit Monoclonal	Cell MARQUE (USA)	Dispenser (Pre-diluted)
AE1/AE3	Mouse Monoclonal	DAKO (Denmark)	1:30
Vimentin	Mouse Monoclonal	Ventana (USA)	Dispenser (Pre-diluted)
CK8-CK18	Mouse Monoclonal	DAKO (Denmark)	Dispenser (Pre-diluted)
HEP-PAR1	Mouse Monoclonal	Ventana (USA)	1:50
AFP	Mouse Monoclonal	Cell MARQUE (USA)	Dispenser (Pre-diluted)
CK19	Mouse Monoclonal	DAKO (Denmark)	Dispenser (Pre-diluted)
PAX8	Rabbit Polyclonal	DAKO (Denmark)	Dispenser (Pre-diluted)
EMA	Rabbit Polyclonal	DAKO (Denmark)	1:250
WT1	Mouse Monoclonal	DAKO (Denmark)	Dispenser (Pre-diluted)
p53	Mouse Monoclonal	DAKO (Denmark)	Dispenser (Pre-diluted)
