# Supplementary material for: Exosomes isolated from cancer patients’ sera transfer malignant traits and confer the same phenotype of primary tumors to oncosuppressor-mutated cells
Source: J Exp Clin Cancer Res. 2017 Aug 30;36:113. doi: 10.1186/s13046-017-0587-0 (PMC5577828; doi:10.1186/s13046-017-0587-0)
Supplement: Supplementary file 2 — MS data from the plasma membrane as visualized on Scaffold Q+. (PDF 1967 kb) [file 13046_2017_587_MOESM2_ESM.pdf]

Supplementary Table 2. MS data from the plasma membrane as visualized on Scaffold Q+.

			T-Test		Control			BRCA1-KO		
**Identified Proteins**	**Alternate Name**	**MW**	**(p-value)**	**Quantitative Profile**	**1**	**2**	**3**	**1**	**2**	**3**
1-phosphatidylinositol 4,5-bisphosphate phosphodiesterase beta-3 OS=Homo sapiens GN=PLCB3 PE=1 SV=2	PLCB3_HUMAN	139 kDa	0.0032	BRAC1 high, Naive low	0	0	0	13	15	11
1-phosphatidylinositol 4,5-bisphosphate phosphodiesterase delta-3 OS=Homo sapiens GN=PLCD3 PE=1 SV=3	PLCD3_HUMAN	89 kDa	0.0082	BRAC1 high, Naive low	0	0	0	8	6	5
2',3'-cyclic-nucleotide 3'-phosphodiesterase OS=Homo sapiens GN=CNP PE=3 SV=1	A0A024R1T5_HUMAN (+1)	45 kDa	0.054	[]	3	6	6	17	18	13
2'-5'-oligoadenylate synthase 2 OS=Homo sapiens GN=OAS2 PE=1 SV=1	A0A087X0V5_HUMAN (+1)	83 kDa	0.72	[]	0	0	4	1	2	5
2'-5'-oligoadenylate synthetase 3, 100kDa, isoform CRA_a OS=Homo sapiens GN=OAS3 PE=4 SV=1	A0A024RBQ5_HUMAN (+2)	121 kDa	0.088	[]	0	1	2	3	4	10
2,4-dienoyl CoA reductase 1, mitochondrial, isoform CRA_b OS=Homo sapiens GN=DECR1 PE=4 SV=1	A0A024R9D7_HUMAN (+1)	36 kDa	0.00047	BRAC1 low, Naive high	3	3	4	1	1	1
2-oxoglutarate dehydrogenase, mitochondrial OS=Homo sapiens GN=OGDH PE=1 SV=3	ODO1_HUMAN	116 kDa	0.42	[]	0	3	6	2	1	4
3-beta-hydroxysteroid-Delta(8),Delta(7)-isomerase OS=Homo sapiens GN=EBP PE=1 SV=3	EBP_HUMAN (+1)	26 kDa	0.37	[]	0	0	0	0	0	4
3-hydroxyacyl-CoA dehydrogenase type-2 OS=Homo sapiens GN=HSD17B10 PE=1 SV=3	HCD2_HUMAN	27 kDa	0.92	[]	2	3	2	5	2	5
3-hydroxyisobutyrate dehydrogenase, mitochondrial OS=Homo sapiens GN=HIBADH PE=1 SV=2	3HIDH_HUMAN (+1)	35 kDa	0.063	[]	1	3	3	0	2	1
3-ketoacyl-CoA thiolase, mitochondrial OS=Homo sapiens GN=ACAA2 PE=1 SV=2	THIM_HUMAN (+3)	42 kDa	0.4	[]	2	1	4	0	1	6
3-mercaptopyruvate sulfurtransferase OS=Homo sapiens GN=MPST PE=1 SV=3	THTM_HUMAN	33 kDa	0.37	[]	0	0	0	0	0	3
4-hydroxyphenylpyruvate dioxygenase-like protein OS=Homo sapiens GN=HPDL PE=1 SV=1	HPDL_HUMAN	39 kDa	0.37	[]	0	0	0	0	2	0
4-trimethylaminobutyraldehyde dehydrogenase OS=Homo sapiens GN=ALDH9A1 PE=1 SV=3	AL9A1_HUMAN (+1)	54 kDa	0.37	[]	0	0	2	0	0	0
4F2 cell-surface antigen heavy chain OS=Homo sapiens GN=SLC3A2 PE=1 SV=3	4F2_HUMAN (+2)	68 kDa	0.22	[]	8	14	23	54	38	26
5'-AMP-activated protein kinase catalytic subunit alpha-1 OS=Homo sapiens GN=PRKAA1 PE=1 SV=4	AAPK1_HUMAN	64 kDa	0.17	[]	0	0	0	1	0	3
5'-AMP-activated protein kinase subunit gamma-1 OS=Homo sapiens GN=PRKAG1 PE=1 SV=1	AAKG1_HUMAN (+2)	38 kDa	0.37	[]	0	0	0	0	0	2
5'-nucleotidase OS=Homo sapiens GN=NT5E PE=1 SV=1	5NTD_HUMAN (+3)	63 kDa	0.021	BRAC1 low, Naive high	13	16	12	9	7	9
6-phosphogluconate dehydrogenase, decarboxylating OS=Homo sapiens GN=PGD PE=1 SV=3	6PGD_HUMAN	53 kDa	0.93	[]	1	1	7	3	3	7
6-phosphogluconate dehydrogenase, decarboxylating OS=Homo sapiens PE=2 SV=1	B4DL86_HUMAN	47 kDa	0.57	[]	0	0	5	2	4	5
6-phosphogluconolactonase OS=Homo sapiens GN=PGLS PE=1 SV=2	6PGL_HUMAN (+3)	28 kDa	0.22	[]	1	1	1	1	1	2
7-dehydrocholesterol reductase, isoform CRA_a OS=Homo sapiens GN=DHCR7 PE=4 SV=1	A0A024R5F7_HUMAN (+2)	54 kDa	0.001	BRAC1 high, Naive low	0	0	0	2	3	3
10 kDa heat shock protein, mitochondrial OS=Homo sapiens GN=HSPE1 PE=1 SV=2	CH10_HUMAN (+1)	11 kDa	0.93	[]	2	2	0	4	2	1
14-3-3 protein beta/alpha OS=Homo sapiens GN=YWHAB PE=1 SV=3	1433B_HUMAN (+1)	28 kDa	0.052	[]	1	0	7	12	11	12
14-3-3 protein epsilon OS=Homo sapiens GN=YWHAE PE=1 SV=1	1433E_HUMAN (+1)	29 kDa	0.14	[]	1	2	8	15	10	10
14-3-3 protein eta OS=Homo sapiens GN=YWHAH PE=1 SV=4	1433F_HUMAN (+1)	28 kDa	0.38	[]	2	2	7	3	3	6
14-3-3 protein gamma OS=Homo sapiens GN=YWHAG PE=1 SV=2	1433G_HUMAN (+2)	28 kDa	0.083	[]	0	0	5	6	7	9
14-3-3 protein sigma OS=Homo sapiens GN=SFN PE=1 SV=1	1433S_HUMAN	28 kDa	0.12	[]	0	0	0	0	2	3
14-3-3 protein theta OS=Homo sapiens GN=YWHAQ PE=1 SV=1	1433T_HUMAN	28 kDa	0.85	[]	1	1	7	4	4	7
14-3-3 protein zeta/delta OS=Homo sapiens GN=YWHAZ PE=1 SV=1	1433Z_HUMAN (+1)	28 kDa	0.35	[]	6	5	8	17	17	9
26S protease regulatory subunit 4 OS=Homo sapiens GN=PSMC1 PE=1 SV=1	PRS4_HUMAN (+2)	49 kDa	0.55	[]	0	0	1	0	0	5
26S protease regulatory subunit 6B OS=Homo sapiens GN=PSMC4 PE=1 SV=2	PRS6B_HUMAN (+1)	47 kDa	0.37	[]	0	0	0	0	0	12
26S protease regulatory subunit 7 OS=Homo sapiens GN=PSMC2 PE=1 SV=3	PRS7_HUMAN (+1)	49 kDa	0.8	[]	1	1	1	0	1	4
26S protease regulatory subunit 8 OS=Homo sapiens GN=PSMC5 PE=1 SV=1	PRS8_HUMAN (+1)	46 kDa	0.31	[]	1	1	3	0	0	4
26S protease regulatory subunit 10B OS=Homo sapiens GN=PSMC6 PE=1 SV=1	PRS10_HUMAN (+2)	44 kDa	0.8	[]	1	0	1	0	1	4
26S proteasome non-ATPase regulatory subunit 1 OS=Homo sapiens GN=PSMD1 PE=1 SV=2	PSMD1_HUMAN (+1)	106 kDa	0.51	[]	0	0	1	0	0	6
26S proteasome non-ATPase regulatory subunit 2 (Fragment) OS=Homo sapiens GN=PSMD2 PE=1 SV=1	H7C1H2_HUMAN	22 kDa	0.37	[]	0	0	0	0	0	7
26S proteasome non-ATPase regulatory subunit 2 OS=Homo sapiens GN=PSMD2 PE=1 SV=3	PSMD2_HUMAN (+1)	100 kDa	0.42	[]	0	1	4	2	2	17
26S proteasome non-ATPase regulatory subunit 5 OS=Homo sapiens GN=PSMD5 PE=1 SV=3	PSMD5_HUMAN (+1)	56 kDa	0.37	[]	0	0	1	0	1	7
26S proteasome non-ATPase regulatory subunit 6 OS=Homo sapiens GN=PSMD6 PE=1 SV=1	PSMD6_HUMAN	46 kDa	0.69	[]	0	0	2	0	0	6
26S proteasome non-ATPase regulatory subunit 11 OS=Homo sapiens GN=PSMD11 PE=1 SV=3	PSD11_HUMAN	47 kDa	0.55	[]	0	0	1	0	0	5
26S proteasome non-ATPase regulatory subunit 12 OS=Homo sapiens GN=PSMD12 PE=1 SV=3	PSD12_HUMAN	53 kDa	0.24	[]	0	0	1	1	1	7
40S ribosomal protein S3 OS=Homo sapiens GN=RPS3 PE=1 SV=2	RS3_HUMAN	27 kDa	0.41	[]	2	3	9	4	3	8
40S ribosomal protein S3a OS=Homo sapiens GN=RPS3A PE=2 SV=1	A8K4W0_HUMAN (+3)	30 kDa	0.91	[]	1	3	4	3	3	8
40S ribosomal protein S4 OS=Homo sapiens GN=RPS4X PE=2 SV=1	B2R491_HUMAN (+3)	30 kDa	0.81	[]	0	3	7	4	3	11
40S ribosomal protein S6 OS=Homo sapiens GN=RPS6 PE=1 SV=1	A2A3R5_HUMAN	25 kDa	0.067	[]	3	3	3	3	1	4
40S ribosomal protein S7 OS=Homo sapiens GN=RPS7 PE=1 SV=1	B5MCP9_HUMAN (+1)	21 kDa	0.86	[]	0	3	2	2	3	4
40S ribosomal protein S8 OS=Homo sapiens GN=RPS8 PE=2 SV=1	Q5JR94_HUMAN (+2)	24 kDa	0.43	[]	5	4	4	5	5	9
40S ribosomal protein S9 OS=Homo sapiens GN=RPS9 PE=1 SV=1	A0A024R4M0_HUMAN (+1)	23 kDa	0.95	[]	1	1	3	2	2	4
40S ribosomal protein S10 OS=Homo sapiens GN=RPS10 PE=1 SV=1	RS10_HUMAN	19 kDa	0.86	[]	0	0	3	1	1	3
40S ribosomal protein S11 OS=Homo sapiens GN=RPS11 PE=1 SV=3	RS11_HUMAN	18 kDa	0.28	[]	1	1	4	1	2	2
40S ribosomal protein S12 OS=Homo sapiens GN=RPS12 PE=1 SV=3	RS12_HUMAN	15 kDa	0.4	[]	0	1	0	2	2	0
40S ribosomal protein S13 OS=Homo sapiens GN=RPS13 PE=1 SV=1	J3KMX5_HUMAN (+1)	17 kDa	0.05	BRAC1 high, Naive low	0	0	2	3	3	4
40S ribosomal protein S14 OS=Homo sapiens GN=RPS14 PE=1 SV=3	RS14_HUMAN	16 kDa	0.46	[]	2	3	1	3	3	1
40S ribosomal protein S18 OS=Homo sapiens GN=RPS18 PE=1 SV=3	RS18_HUMAN	18 kDa	0.73	[]	4	2	1	4	5	1
40S ribosomal protein S20 OS=Homo sapiens GN=RPS20 PE=1 SV=1	RS20_HUMAN	13 kDa	0.57	[]	0	1	2	3	2	1
40S ribosomal protein S23 OS=Homo sapiens GN=RPS23 PE=1 SV=3	RS23_HUMAN (+1)	16 kDa	0.21	[]	1	1	1	0	1	2
40S ribosomal protein SA OS=Homo sapiens GN=RPSA PE=3 SV=1	A0A024R2P0_HUMAN (+3)	33 kDa	0.2	[]	5	7	6	8	8	8

60S ribosomal protein L3 OS=Homo sapiens GN=RPL3 PE=1 SV=2	RL3_HUMAN	46 kDa	0.42	[]	2	3	5	3	2	8
60S ribosomal protein L4 OS=Homo sapiens GN=RPL4 PE=1 SV=5	RL4_HUMAN	48 kDa	0.13	[]	9	12	12	13	14	20
60S ribosomal protein L6 OS=Homo sapiens GN=RPL6 PE=3 SV=1	A0A024RBK3_HUMAN (+4)	33 kDa	0.12	[]	4	9	10	14	16	21
60S ribosomal protein L7a OS=Homo sapiens GN=RPL7A PE=1 SV=2	RL7A_HUMAN	30 kDa	0.61	[]	5	7	7	9	11	15
60S ribosomal protein L8 (Fragment) OS=Homo sapiens GN=RPL8 PE=1 SV=1	E9PKZ0_HUMAN (+1)	22 kDa	0.29	[]	1	4	3	2	2	4
60S ribosomal protein L9 (Fragment) OS=Homo sapiens GN=RPL9 PE=1 SV=6	D6RAN4_HUMAN (+2)	21 kDa	0.34	[]	0	0	4	2	3	8
60S ribosomal protein L10a OS=Homo sapiens GN=RPL10A PE=1 SV=2	RL10A_HUMAN (+1)	25 kDa	0.91	[]	0	0	2	0	2	1
60S ribosomal protein L12 OS=Homo sapiens GN=RPL12 PE=1 SV=1	RL12_HUMAN	18 kDa	0.14	[]	2	4	3	3	3	4
60S ribosomal protein L13 OS=Homo sapiens GN=RPL13 PE=2 SV=1	A8K4C8_HUMAN (+2)	24 kDa	0.11	[]	2	3	2	1	1	4
60S ribosomal protein L14 OS=Homo sapiens GN=RPL14 PE=1 SV=4	RL14_HUMAN	23 kDa	0.18	[]	2	2	4	2	4	3
60S ribosomal protein L18a OS=Homo sapiens PE=2 SV=1	B2R4C0_HUMAN (+5)	21 kDa	0.11	[]	2	1	2	1	1	2
60S ribosomal protein L23a OS=Homo sapiens GN=RPL23A PE=1 SV=1	A8MUS3_HUMAN (+5)	22 kDa	0.077	[]	0	1	1	2	2	4
60S ribosomal protein L26-like 1 OS=Homo sapiens GN=RPL26L1 PE=1 SV=1	RL26L_HUMAN (+8)	17 kDa	0.87	[]	0	0	1	0	0	2
60S ribosomal protein L27a OS=Homo sapiens GN=RPL27A PE=1 SV=1	E9PLL6_HUMAN (+3)	12 kDa	0.8	[]	1	0	1	2	1	1
60S ribosomal protein L28 OS=Homo sapiens GN=RPL28 PE=1 SV=1	H0YKD8_HUMAN (+3)	19 kDa	0.69	[]	1	2	0	3	3	1
60S ribosomal protein L38 OS=Homo sapiens GN=RPL38 PE=1 SV=2	RL38_HUMAN (+4)	8 kDa	0.66	[]	1	1	0	0	1	2
78 kDa glucose-regulated protein OS=Homo sapiens GN=HSPA5 PE=1 SV=2	GRP78_HUMAN (+1)	72 kDa	0.1	[]	54	56	53	52	49	90
116 kDa U5 small nuclear ribonucleoprotein component OS=Homo sapiens GN=EFTUD2 PE=1 SV=1	U5S1_HUMAN (+4)	109 kDa	0.37	[]	0	0	0	0	0	6
A-kinase anchor protein 12 OS=Homo sapiens GN=AKAP12 PE=1 SV=4	AKA12_HUMAN	191 kDa	0.16	[]	2	5	0	0	0	0
ADAM metallopeptidase domain 10, isoform CRA_b OS=Homo sapiens GN=ADAM10 PE=4 SV=1	A0A024R5U5_HUMAN (+1)	84 kDa	0.011	BRAC1 high, Naive low	1	0	1	12	10	8
ADP-dependent glucokinase OS=Homo sapiens GN=ADPGK PE=1 SV=1	ADPGK_HUMAN (+1)	54 kDa	0.37	[]	0	0	0	0	0	2
ADP-ribosyl cyclase/cyclic ADP-ribose hydrolase 2 OS=Homo sapiens GN=BST1 PE=1 SV=2	BST1_HUMAN (+1)	36 kDa	0.017	BRAC1 low, Naive high	1	1	3	0	0	0
ADP-ribosylation factor 3, isoform CRA_b OS=Homo sapiens GN=ARF3 PE=3 SV=1	A0A024R0Y6_HUMAN (+5)	21 kDa	0.27	[]	0	0	2	2	2	2
ADP-ribosylation factor 4 OS=Homo sapiens GN=ARF4 PE=1 SV=3	ARF4_HUMAN (+1)	21 kDa	0.43	[]	0	0	2	2	2	1
ADP-ribosylation factor 6 OS=Homo sapiens GN=ARF6 PE=1 SV=2	ARF6_HUMAN (+1)	20 kDa	0.25	[]	1	0	0	2	3	1
ADP-ribosylation factor GTPase-activating protein 1 OS=Homo sapiens GN=ARFGAP1 PE=1 SV=2	ARFG1_HUMAN (+5)	45 kDa	0.17	[]	0	0	0	1	0	3
ADP-ribosylation factor GTPase-activating protein 2 OS=Homo sapiens GN=ARFGAP2 PE=1 SV=1	ARFG2_HUMAN (+3)	57 kDa	0.37	[]	0	0	0	0	0	3
ADP/ATP translocase 1 OS=Homo sapiens GN=SLC25A4 PE=1 SV=4	ADT1_HUMAN (+1)	33 kDa	0.96	[]	3	0	3	4	3	3
ADP/ATP translocase 2 OS=Homo sapiens GN=SLC25A5 PE=1 SV=7	ADT2_HUMAN	33 kDa	0.025	BRAC1 high, Naive low	3	2	3	7	9	10
ADP/ATP translocase 3 OS=Homo sapiens GN=SLC25A6 PE=1 SV=4	ADT3_HUMAN (+1)	33 kDa	0.32	[]	4	2	4	4	4	4
AFG3-like protein 2 OS=Homo sapiens GN=AFG3L2 PE=1 SV=2	AFG32_HUMAN (+2)	89 kDa	0.87	[]	0	0	1	0	0	2
AGTRAP-BRAF fusion protein OS=Homo sapiens PE=2 SV=1	D7RF68_HUMAN	66 kDa	0.66	[]	1	1	0	1	0	2
AH receptor-interacting protein OS=Homo sapiens GN=AIP PE=1 SV=2	AIP_HUMAN (+13)	38 kDa	0.37	[]	0	0	0	0	0	3
AP-1 complex subunit gamma-1 OS=Homo sapiens GN=AP1G1 PE=1 SV=5	AP1G1_HUMAN (+2)	91 kDa	0.07	[]	0	0	0	1	1	4
AP-1 complex subunit mu-2 OS=Homo sapiens GN=AP1M2 PE=1 SV=4	AP1M2_HUMAN (+2)	48 kDa	0.00029	BRAC1 high, Naive low	0	0	0	2	2	2
AP-2 complex subunit alpha-1 OS=Homo sapiens GN=AP2A1 PE=1 SV=3	AP2A1_HUMAN	108 kDa	0.034	BRAC1 low, Naive high	6	6	10	8	8	6
AP-2 complex subunit alpha-2 OS=Homo sapiens GN=AP2A2 PE=1 SV=2	AP2A2_HUMAN (+1)	104 kDa	0.86	[]	3	4	7	9	7	5
AP-1 complex subunit beta-1 OS=Homo sapiens GN=AP1B1 PE=1 SV=2	AP1B1_HUMAN	105 kDa	0.032	BRAC1 high, Naive low	3	2	3	9	15	12
AP-2 complex subunit beta OS=Homo sapiens GN=AP2B1 PE=1 SV=1	A0A087X253_HUMAN (+4)	101 kDa	0.7	[]	8	10	9	12	20	7
AP-2 complex subunit mu OS=Homo sapiens GN=AP2M1 PE=1 SV=1	A0A087WY71_HUMAN (+4)	50 kDa	0.55	[]	0	0	2	0	0	1
AP-3 complex subunit beta-1 OS=Homo sapiens GN=AP3B1 PE=1 SV=3	AP3B1_HUMAN (+2)	121 kDa	0.69	[]	0	0	1	0	0	3
ARHG protein (Fragment) OS=Homo sapiens GN=ARHG PE=2 SV=1	Q6ICQ8_HUMAN (+1)	21 kDa	0.17	[]	2	2	2	7	6	4
ARP3 actin-related protein 3 homolog (Yeast), isoform CRA_a OS=Homo sapiens GN=ACTR3 PE=3 SV=1	A0A024RAI1_HUMAN (+1)	47 kDa	0.54	[]	6	10	12	25	19	11
ATP synthase subunit O, mitochondrial OS=Homo sapiens GN=ATP5O PE=1 SV=1	ATPO_HUMAN	23 kDa	0.2	[]	6	5	5	6	7	5
ATP synthase subunit alpha, mitochondrial OS=Homo sapiens GN=ATP5A1 PE=1 SV=1	ATPA_HUMAN (+1)	60 kDa	0.14	[]	31	27	18	23	22	21
ATP synthase subunit beta, mitochondrial OS=Homo sapiens GN=ATP5B PE=1 SV=3	ATPB_HUMAN (+1)	57 kDa	0.017	BRAC1 low, Naive high	23	22	25	16	20	24
ATP synthase subunit g, mitochondrial OS=Homo sapiens GN=ATP5L PE=1 SV=3	ATP5L_HUMAN (+1)	11 kDa	0.54	[]	2	1	0	1	2	0
ATP synthase subunit gamma, mitochondrial OS=Homo sapiens GN=ATP5C1 PE=1 SV=1	ATPG_HUMAN (+2)	33 kDa	0.71	[]	0	2	4	2	2	3
ATP-binding cassette sub-family D member 1 OS=Homo sapiens GN=ABCD1 PE=1 SV=2	ABCD1_HUMAN	83 kDa	0.88	[]	0	2	0	2	1	1
ATP-binding cassette sub-family D member 3 OS=Homo sapiens GN=ABCD3 PE=1 SV=1	ABCD3_HUMAN	75 kDa	0.28	[]	0	1	0	1	1	7
ATP-binding cassette sub-family E member 1 OS=Homo sapiens GN=ABCE1 PE=1 SV=1	ABCE1_HUMAN (+1)	67 kDa	0.17	[]	0	0	0	1	1	7
ATP-binding cassette sub-family F member 1 OS=Homo sapiens GN=ABCF1 PE=1 SV=2	ABCF1_HUMAN (+3)	96 kDa	0.19	[]	0	0	0	2	0	7
ATP-binding cassette sub-family F member 2 OS=Homo sapiens GN=ABCF2 PE=1 SV=2	ABCF2_HUMAN (+2)	71 kDa	0.13	[]	0	0	0	0	1	2
ATP-binding cassette, sub-family G (WHITE), member 2, isoform CRA_a OS=Homo sapiens GN=ABCG2 PE=4 SV=1	A0A024RDD4_HUMAN (+2)	72 kDa	0.0027	BRAC1 high, Naive low	0	0	0	1	1	2
ATP-citrate synthase OS=Homo sapiens GN=ACLY PE=3 SV=1	A0A024R1T9_HUMAN (+3)	121 kDa	0.81	[]	1	2	9	1	2	13
ATP-dependent 6-phosphofructokinase, liver type OS=Homo sapiens GN=PFKL PE=1 SV=6	PFKAL_HUMAN	85 kDa	0.68	[]	1	0	3	0	1	11
ATP-dependent 6-phosphofructokinase, muscle type OS=Homo sapiens GN=PFKM PE=1 SV=2	PFKAM_HUMAN (+2)	85 kDa	0.69	[]	0	0	1	0	0	3
ATP-dependent 6-phosphofructokinase, platelet type OS=Homo sapiens GN=PFKP PE=1 SV=2	PFKAP_HUMAN	86 kDa	0.39	[]	1	0	4	2	3	14
ATP-dependent RNA helicase A OS=Homo sapiens GN=DHX9 PE=1 SV=4	DHX9_HUMAN	141 kDa	1	[]	0	0	6	0	1	8
ATP-dependent RNA helicase DDX1 OS=Homo sapiens GN=DDX1 PE=1 SV=1	A0A087X2G1_HUMAN (+3)	74 kDa	0.82	[]	0	1	3	1	2	4
ATP-dependent RNA helicase DDX3X OS=Homo sapiens GN=DDX3X PE=1 SV=1	A0A0D9SF53_HUMAN (+4)	81 kDa	0.23	[]	1	4	5	6	6	20
ATPase family AAA domain-containing protein 3A (Fragment) OS=Homo sapiens GN=ATAD3A PE=1 SV=1	H0Y2W2_HUMAN	64 kDa	0.2	[]	1	3	4	2	2	4

ATPase family AAA domain-containing protein 3A OS=Homo sapiens GN=ATAD3A PE=1 SV=2	ATD3A_HUMAN	71 kDa	0.38	[]	1	4	3	2	2	5
ATPase, H+ transporting, lysosomal 42kDa, V1 subunit C1, isoform CRA_a OS=Homo sapiens GN=ATP6V1C1 PE=4 SV=1	A0A024R9I0_HUMAN (+1)	44 kDa	0.9	[]	0	1	1	1	0	2
Abl interactor 1 OS=Homo sapiens GN=ABI1 PE=1 SV=1	A0A0A0MRT6_HUMAN (+5)	52 kDa	0.16	[]	1	0	0	2	2	2
Acetyl-CoA acetyltransferase, cytosolic OS=Homo sapiens GN=ACAT2 PE=1 SV=2	THIC_HUMAN (+2)	41 kDa	0.37	[]	0	0	0	0	0	3
Acetyl-CoA acetyltransferase, mitochondrial OS=Homo sapiens GN=ACAT1 PE=1 SV=1	THIL_HUMAN	45 kDa	0.038	BRAC1 low, Naive high	2	3	2	2	0	1
Acetyl-Coenzyme A acyltransferase 1 (Peroxisomal 3-oxoacyl-Coenzyme A thiolase), isoform CRA_a OS=Homo sapiens GN=ACAA1 PE	A0A024R2M6_HUMAN (+1)	44 kDa	0.51	[]	3	3	2	4	3	5
Acetyl-coenzyme A synthetase 2-like, mitochondrial OS=Homo sapiens GN=ACSS1 PE=1 SV=2	ACS2L_HUMAN (+2)	75 kDa	0.13	[]	0	0	0	0	1	2
Acetyltransferase component of pyruvate dehydrogenase complex OS=Homo sapiens GN=DLAT PE=3 SV=1	A0A024R3D8_HUMAN (+3)	69 kDa	0.49	[]	0	1	1	1	1	3
Actin related protein 2/3 complex, subunit 1B, 41kDa OS=Homo sapiens GN=ARPC1B PE=2 SV=1	A4D275_HUMAN (+1)	41 kDa	0.63	[]	3	4	9	9	5	8
Actin-binding LIM protein 1 OS=Homo sapiens GN=ABLIM1 PE=1 SV=1	A0A0A0MRL6_HUMAN (+4)	84 kDa	0.16	[]	0	0	0	1	2	0
ACTA2 protein (Fragment) OS=Homo sapiens GN=ACTA2 PE=3 SV=1	Q13707_HUMAN	37 kDa	0.27	[]	35	31	30	48	39	20
Actin-like protein (Fragment) OS=Homo sapiens GN=ACT PE=3 SV=1	Q562P8_HUMAN (+2)	12 kDa	0.37	[]	0	0	6	0	0	0
Actin-like protein (Fragment) OS=Homo sapiens GN=ACT PE=3 SV=1	Q562N4_HUMAN	12 kDa	0.37	[]	0	0	16	0	0	0
Actin-like protein (Fragment) OS=Homo sapiens GN=ACT PE=3 SV=1	Q562S0_HUMAN	12 kDa	0.11	[]	29	22	32	32	30	21
Actin-like protein (Fragment) OS=Homo sapiens GN=ACT PE=3 SV=1	Q562M5_HUMAN	12 kDa	0.2	[]	0	16	24	0	0	13
Actin-like protein (Fragment) OS=Homo sapiens GN=ACT PE=3 SV=1	Q562U2_HUMAN	11 kDa	0.5	[]	0	9	0	0	20	15
Actin-like protein (Fragment) OS=Homo sapiens GN=ACT PE=3 SV=1	Q562Z4_HUMAN	12 kDa	0.37	[]	0	7	0	0	0	0
Beta actin (Fragment) OS=Homo sapiens PE=4 SV=1	K4ENJ5_HUMAN (+2)	11 kDa	0.33	[]	19	17	0	0	21	0
Beta-actin-like protein 2 OS=Homo sapiens GN=ACTBL2 PE=1 SV=2	ACTBL_HUMAN	42 kDa	0.11	[]	21	31	30	30	36	17
Epididymis luminal protein 176 OS=Homo sapiens GN=HEL-176 PE=2 SV=1	V9HVZ7_HUMAN	25 kDa	0.037	BRAC1 low, Naive high	48	47	55	54	47	38
POTE ankyrin domain family member F OS=Homo sapiens GN=POTEF PE=1 SV=2	POTEF_HUMAN	121 kDa	0.26	[]	45	40	46	62	60	33
POTE ankyrin domain family member I OS=Homo sapiens GN=POTEI PE=3 SV=1	POTEI_HUMAN	121 kDa	0.31	[]	28	25	31	43	41	23
Actin, aortic smooth muscle OS=Homo sapiens GN=ACTA2 PE=1 SV=1	ACTA_HUMAN (+1)	42 kDa	0.17	[]	43	40	45	58	51	26
POTE ankyrin domain family member J OS=Homo sapiens GN=POTEJ PE=3 SV=1	POTEJ_HUMAN	117 kDa	0.24	[]	16	15	24	20	30	16
cDNA FLJ39583 fis, clone SKMUS2004897, highly similar to ACTIN, ALPHA SKELETAL MUSCLE OS=Homo sapiens PE=2 SV=1	B3KUD3_HUMAN	38 kDa	0.22	[]	42	39	39	57	49	25
cDNA FLJ53662, highly similar to Actin, alpha skeletal muscle OS=Homo sapiens PE=2 SV=1	B7Z6P1_HUMAN	39 kDa	0.26	[]	43	39	46	60	60	29
cDNA FLJ55253, highly similar to Actin, cytoplasmic 1 OS=Homo sapiens PE=2 SV=1	B4DW52_HUMAN	39 kDa	0.12	[]	94	83	100	117	97	66
cDNA FLJ57283, highly similar to Actin, cytoplasmic 2 OS=Homo sapiens PE=2 SV=1	B4E3A4_HUMAN	40 kDa	0.12	[]	88	83	99	117	105	69
cDNA, FLJ79260, highly similar to Actin, cytoplasmic 2 OS=Homo sapiens PE=2 SV=1	B7ZAP6_HUMAN	33 kDa	0.047	BRAC1 low, Naive high	67	61	73	74	58	45
Actin-like protein (Fragment) OS=Homo sapiens GN=ACT PE=3 SV=1	Q562V5_HUMAN	11 kDa	0.14	[]	10	19	23	19	18	29
Actin-like protein (Fragment) OS=Homo sapiens GN=ACT PE=3 SV=1	Q562L5_HUMAN	12 kDa	0.17	[]	29	24	36	35	33	30
Actin-like protein (Fragment) OS=Homo sapiens GN=ACT PE=3 SV=1	Q562Z6_HUMAN	11 kDa	0.08	[]	22	18	20	24	14	13
Actin-like protein (Fragment) OS=Homo sapiens GN=ACT PE=3 SV=1	Q562N0_HUMAN	11 kDa	0.049	BRAC1 low, Naive high	29	25	33	32	31	24
Actin-like protein (Fragment) OS=Homo sapiens GN=ACT PE=3 SV=1	Q562P9_HUMAN	11 kDa	0.43	[]	10	7	15	14	17	12
Actin-like protein (Fragment) OS=Homo sapiens GN=ACT PE=3 SV=1	Q562Y8_HUMAN	12 kDa	0.11	[]	20	15	26	23	24	15
Actin-like protein (Fragment) OS=Homo sapiens GN=ACT PE=3 SV=1	Q562U1_HUMAN	11 kDa	0.91	[]	0	0	10	5	10	0
Actin-related protein 2 OS=Homo sapiens GN=ACTR2 PE=1 SV=1	ARP2_HUMAN	45 kDa	0.81	[]	1	2	11	7	5	12
cDNA FLJ51656, highly similar to Actin-like protein 2 OS=Homo sapiens PE=2 SV=1	B4DHK9_HUMAN	39 kDa	0.85	[]	0	2	7	3	4	8
Actin-related protein 2/3 complex subunit 1A OS=Homo sapiens GN=ARPC1A PE=1 SV=2	ARC1A_HUMAN (+1)	42 kDa	0.93	[]	0	0	3	1	3	0
Actin-related protein 2/3 complex subunit 2 OS=Homo sapiens GN=ARPC2 PE=1 SV=1	ARPC2_HUMAN (+1)	34 kDa	0.51	[]	3	3	12	12	12	10
Actin-related protein 2/3 complex subunit 3 OS=Homo sapiens GN=ARPC3 PE=1 SV=3	ARPC3_HUMAN (+2)	21 kDa	0.18	[]	0	0	1	1	5	2
Actin-related protein 2/3 complex subunit 5 OS=Homo sapiens GN=ARPC5L PE=3 SV=1	A0A024R897_HUMAN (+2)	17 kDa	0.064	[]	0	0	0	5	4	1
Activator of 90 kDa heat shock protein ATPase homolog 1 OS=Homo sapiens GN=AHSA1 PE=1 SV=1	AHSA1_HUMAN (+1)	38 kDa	0.13	[]	0	0	0	1	0	2
Active breakpoint cluster region-related protein OS=Homo sapiens GN=ABR PE=2 SV=2	ABR_HUMAN (+3)	98 kDa	0.37	[]	0	0	0	0	0	2
Acyl-CoA dehydrogenase family member 9, mitochondrial OS=Homo sapiens GN=ACAD9 PE=1 SV=1	ACAD9_HUMAN	69 kDa	0.54	[]	0	2	1	2	2	3
Acyl-CoA synthetase family member 2, mitochondrial OS=Homo sapiens GN=ACSF2 PE=1 SV=2	ACSF2_HUMAN (+2)	68 kDa	0.07	[]	0	0	0	1	1	4
Acyl-CoA synthetase long-chain family member 3, isoform CRA_a OS=Homo sapiens GN=ACSL3 PE=4 SV=1	A0A024R497_HUMAN (+2)	80 kDa	0.32	[]	2	4	5	5	7	17
Acyl-coenzyme A thioesterase 9, mitochondrial OS=Homo sapiens GN=ACOT9 PE=1 SV=2	ACOT9_HUMAN	50 kDa	0.00091	BRAC1 low, Naive high	1	1	2	0	0	0
Acylglycerol kinase, mitochondrial OS=Homo sapiens GN=AGK PE=1 SV=2	AGK_HUMAN (+3)	47 kDa	0.5	[]	0	0	1	0	1	2
Adenine phosphoribosyltransferase OS=Homo sapiens GN=APRT PE=1 SV=2	APT_HUMAN (+2)	20 kDa	0.05	BRAC1 high, Naive low	0	0	0	2	2	7
Adenosylhomocysteinase OS=Homo sapiens GN=AHCY PE=1 SV=4	SAHH_HUMAN	48 kDa	0.0014	BRAC1 high, Naive low	1	1	1	5	6	6
Adenosylhomocysteinase OS=Homo sapiens GN=AHCYL1 PE=3 SV=1	A0A024R0A8_HUMAN (+6)	59 kDa	0.2	[]	0	0	0	1	3	0
Adenylate kinase 2, mitochondrial OS=Homo sapiens GN=AK2 PE=1 SV=1	F8VY04_HUMAN (+2)	21 kDa	0.29	[]	1	0	4	6	5	3
Adenylosuccinate synthetase isozyme 2 OS=Homo sapiens GN=ADSS PE=1 SV=3	PURA2_HUMAN (+2)	50 kDa	0.37	[]	0	0	0	0	0	3
Adenylyl cyclase-associated protein OS=Homo sapiens PE=2 SV=1	B4DI38_HUMAN (+1)	49 kDa	0.85	[]	4	4	11	7	14	11
tr|B4DNA3|B4DNA3_HUMAN Adenylyl cyclase-associated protein OS=Homo sapiens PE=2 SV=1	B4DNA3_HUMAN	?	0.68	[]	2	0	8	0	4	7
Adipocyte plasma membrane-associated protein OS=Homo sapiens GN=APMAP PE=1 SV=2	APMAP_HUMAN	46 kDa	0.61	[]	0	1	4	2	2	7
Afadin (Fragment) OS=Homo sapiens GN=MLLT4 PE=1 SV=1	H0Y7R8_HUMAN (+1)	43 kDa	0.12	[]	0	0	0	5	5	0
Afadin OS=Homo sapiens GN=MLLT4 PE=1 SV=2	A8MQ02_HUMAN (+3)	202 kDa	0.061	[]	0	0	0	19	18	4
Aflatoxin B1 aldehyde reductase member 2 OS=Homo sapiens GN=AKR7A2 PE=1 SV=3	ARK72_HUMAN (+2)	40 kDa	0.011	BRAC1 high, Naive low	0	0	0	1	2	3
Agrin OS=Homo sapiens GN=AGRN PE=1 SV=5	AGRIN_HUMAN	217 kDa	0.0037	BRAC1 high, Naive low	0	0	0	6	7	5
Aladin OS=Homo sapiens GN=AAAS PE=1 SV=1	AAAS_HUMAN (+3)	60 kDa	0.37	[]	0	0	0	0	0	2

Alanine--tRNA ligase, cytoplasmic OS=Homo sapiens GN=AARS PE=1 SV=2	SYAC_HUMAN	107 kDa	0.04	BRAC1 high, Naive low	0	0	0	8	8	26
Alcohol dehydrogenase [NADP(+)] OS=Homo sapiens GN=AKR1A1 PE=1 SV=3	AK1A1_HUMAN (+1)	37 kDa	0.07	[]	0	0	0	1	1	4
Alcohol dehydrogenase class-3 OS=Homo sapiens GN=ADH5 PE=1 SV=4	ADHX_HUMAN (+3)	40 kDa	0.98	[]	0	0	2	0	0	3
Aldehyde dehydrogenase 4 family, member A1, isoform CRA_a OS=Homo sapiens GN=ALDH4A1 PE=3 SV=1	A0A024RAD8_HUMAN (+1)	62 kDa	0.27	[]	1	1	1	2	2	8
Aldehyde dehydrogenase X, mitochondrial OS=Homo sapiens GN=ALDH1B1 PE=1 SV=3	AL1B1_HUMAN	57 kDa	0.0054	BRAC1 low, Naive high	3	2	5	0	0	0
Aldehyde dehydrogenase family 16 member A1 OS=Homo sapiens GN=ALDH16A1 PE=1 SV=2	A16A1_HUMAN	85 kDa	0.26	[]	0	0	1	0	2	4
Aldehyde dehydrogenase, mitochondrial OS=Homo sapiens GN=ALDH2 PE=1 SV=2	ALDH2_HUMAN (+1)	56 kDa	0.45	[]	0	1	5	4	7	3
Aldo-keto reductase family 1 member C3 OS=Homo sapiens GN=AKR1C3 PE=1 SV=1	A0A0A0MSS8_HUMAN (+2)	37 kDa	0.07	[]	0	0	0	1	1	4
Alkyldihydroxyacetonephosphate synthase, peroxisomal OS=Homo sapiens GN=AGPS PE=1 SV=1	ADAS_HUMAN	73 kDa	0.023	BRAC1 high, Naive low	0	1	0	3	5	9
Alpha-1-antitrypsin OS=Homo sapiens GN=SERPINA1 PE=1 SV=3	A1AT_HUMAN (+3)	47 kDa	0.37	[]	0	0	0	0	0	6
Alpha-2-macroglobulin receptor-associated protein OS=Homo sapiens GN=LRPAP1 PE=1 SV=1	AMRP_HUMAN (+1)	41 kDa	0.81	[]	0	1	2	0	0	4
Alpha-2A adrenergic receptor OS=Homo sapiens GN=ADRA2A PE=1 SV=3	ADA2A_HUMAN	49 kDa	0.12	[]	0	0	0	5	10	1
Alpha-N-acetylglucosaminidase OS=Homo sapiens GN=NAGLU PE=1 SV=2	ANAG_HUMAN (+2)	82 kDa	0.37	[]	0	0	0	0	0	2
Actinin, alpha 1, isoform CRA_a OS=Homo sapiens GN=ACTN1 PE=4 SV=1	A0A024R694_HUMAN (+1)	103 kDa	0.75	[]	27	27	58	80	75	39
Alpha-actinin-2 OS=Homo sapiens GN=ACTN2 PE=1 SV=1	ACTN2_HUMAN	104 kDa	0.37	[]	0	0	0	0	19	0
Alpha-actinin-3 OS=Homo sapiens GN=ACTN3 PE=1 SV=2	ACTN3_HUMAN (+1)	103 kDa	0.37	[]	0	0	7	0	0	0
Alpha-actinin-4 OS=Homo sapiens GN=ACTN4 PE=1 SV=2	ACTN4_HUMAN	105 kDa	0.074	[]	26	27	54	174	166	88
Alpha-actinin-4 OS=Homo sapiens GN=ACTN4 PE=1 SV=2	F5GXS2_HUMAN	60 kDa	0.073	[]	14	15	31	106	97	50
Alpha-adducin OS=Homo sapiens GN=ADD1 PE=1 SV=2	ADDA_HUMAN (+2)	81 kDa	0.19	[]	0	2	0	2	3	5
Alpha-aminoadipic semialdehyde dehydrogenase OS=Homo sapiens GN=ALDH7A1 PE=1 SV=5	AL7A1_HUMAN	58 kDa	0.0044	BRAC1 low, Naive high	3	2	4	0	0	0
Alpha-centractin OS=Homo sapiens GN=ACTR1A PE=1 SV=1	ACTZ_HUMAN (+3)	43 kDa	0.022	BRAC1 low, Naive high	2	3	6	0	1	3
Beta-centractin OS=Homo sapiens GN=ACTR1B PE=1 SV=1	ACTY_HUMAN (+1)	42 kDa	0.37	[]	0	0	2	0	0	0
Alpha-mannosidase 2 OS=Homo sapiens GN=MAN2A1 PE=1 SV=2	MA2A1_HUMAN	131 kDa	0.37	[]	0	0	0	0	0	2
Amine oxidase [flavin-containing] A OS=Homo sapiens GN=MAOA PE=1 SV=1	AOFA_HUMAN (+3)	60 kDa	0.0068	BRAC1 high, Naive low	0	0	0	3	3	2
Amino acid transporter (Fragment) OS=Homo sapiens PE=2 SV=1	Q59ES3_HUMAN	57 kDa	0.049	BRAC1 high, Naive low	4	6	6	42	40	20
Neutral amino acid transporter B(0) OS=Homo sapiens GN=SLC1A5 PE=1 SV=2	AAAT_HUMAN (+1)	57 kDa	0.038	BRAC1 high, Naive low	4	5	5	38	37	20
Amino acid transporter OS=Homo sapiens PE=2 SV=1	B2R7N6_HUMAN (+2)	56 kDa	0.04	BRAC1 high, Naive low	0	0	1	5	7	3
Aminoacyl tRNA synthase complex-interacting multifunctional protein 1 OS=Homo sapiens GN=AIMP1 PE=1 SV=2	AIMP1_HUMAN	34 kDa	0.21	[]	0	0	0	1	0	4
Aminoacyl tRNA synthase complex-interacting multifunctional protein 2 OS=Homo sapiens GN=AIMP2 PE=1 SV=2	AIMP2_HUMAN (+2)	35 kDa	0.37	[]	0	0	0	0	0	5
tr|A8MU58|A8MU58_HUMAN Aminoacyl tRNA synthase complex-interacting multifunctional...	A8MU58_HUMAN	?	0.37	[]	0	0	0	0	0	5
Aminopeptidase B OS=Homo sapiens GN=RNPEP PE=1 SV=2	AMPB_HUMAN	73 kDa	0.11	[]	0	0	1	2	1	5
Aminopeptidase N OS=Homo sapiens GN=ANPEP PE=1 SV=4	AMPN_HUMAN (+2)	110 kDa	0.0092	BRAC1 low, Naive high	76	83	58	0	0	0
Anion exchange protein 2 OS=Homo sapiens GN=SLC4A2 PE=1 SV=4	B3A2_HUMAN (+1)	137 kDa	0.12	[]	0	0	0	2	2	0
Ankycorbin OS=Homo sapiens GN=RAI14 PE=1 SV=2	RAI14_HUMAN	110 kDa	0.2	[]	1	1	8	1	0	0
Annexin (Fragment) OS=Homo sapiens PE=2 SV=1	Q59EP1_HUMAN (+1)	55 kDa	0.41	[]	3	3	15	6	4	9
Annexin A1 OS=Homo sapiens GN=ANXA1 PE=1 SV=2	ANXA1_HUMAN (+1)	39 kDa	0.00029	BRAC1 low, Naive high	43	42	69	10	12	11
Annexin A3 OS=Homo sapiens GN=ANXA3 PE=1 SV=3	ANXA3_HUMAN	36 kDa	0.54	[]	0	0	5	4	3	4
Annexin A5 OS=Homo sapiens GN=ANXA5 PE=1 SV=2	ANXA5_HUMAN (+1)	36 kDa	0.013	BRAC1 low, Naive high	31	20	44	13	12	11
Annexin A6 OS=Homo sapiens GN=ANXA6 PE=1 SV=3	ANXA6_HUMAN	76 kDa	0.00067	BRAC1 low, Naive high	26	39	58	2	5	15
Annexin OS=Homo sapiens GN=ANXA6 PE=1 SV=1	E5RK69_HUMAN	52 kDa	0.0017	BRAC1 low, Naive high	18	24	42	2	5	12
Annexin A7 OS=Homo sapiens GN=ANXA7 PE=1 SV=3	ANXA7_HUMAN (+6)	53 kDa	0.52	[]	0	0	2	1	1	3
Annexin A9 OS=Homo sapiens GN=ANXA9 PE=1 SV=3	ANXA9_HUMAN	38 kDa	0.37	[]	0	0	0	0	0	2
Annexin A13 OS=Homo sapiens GN=ANXA13 PE=1 SV=3	ANX13_HUMAN (+1)	35 kDa	0.12	[]	0	0	0	12	11	0
Annexin OS=Homo sapiens PE=2 SV=1	A8K2L6_HUMAN	40 kDa	0.12	[]	0	0	0	10	10	0
Annexin (Fragment) OS=Homo sapiens GN=ANXA2 PE=1 SV=1	H0YNP5_HUMAN	20 kDa	0.85	[]	80	83	62	205	164	44
Annexin OS=Homo sapiens GN=ANXA2 PE=3 SV=1	A0A024R5Z7_HUMAN (+2)	39 kDa	0.86	[]	114	134	100	303	251	74
Annexin OS=Homo sapiens GN=HEL-S-274 PE=2 SV=1	V9HW59_HUMAN	36 kDa	0.82	[]	3	5	15	12	12	14
Annexin OS=Homo sapiens PE=2 SV=1	B4DE02_HUMAN	34 kDa	0.95	[]	3	5	15	11	11	14
Anoctamin-6 OS=Homo sapiens GN=ANO6 PE=1 SV=2	ANO6_HUMAN (+1)	106 kDa	0.0062	BRAC1 high, Naive low	0	1	0	6	4	5
Anterior gradient protein 2 homolog OS=Homo sapiens GN=AGR2 PE=1 SV=1	AGR2_HUMAN (+4)	20 kDa	0.0074	BRAC1 high, Naive low	0	0	0	6	8	5
Antigen peptide transporter 1 OS=Homo sapiens GN=TAP1 PE=1 SV=2	TAP1_HUMAN (+1)	87 kDa	0.059	[]	4	2	9	1	1	3
TAP1 OS=Homo sapiens GN=TAP1 PE=2 SV=1	Q6QWC0_HUMAN (+1)	81 kDa	0.059	[]	4	2	9	1	1	3
Apolipoprotein B-100 OS=Homo sapiens GN=APOB PE=1 SV=1	A0A087WTM7_HUMAN (+3)	490 kDa	0.2	[]	0	0	1	3	4	0
Apolipoprotein L2 OS=Homo sapiens GN=APOL2 PE=1 SV=1	APOL2_HUMAN (+3)	37 kDa	0.1	[]	1	2	1	0	0	2
Apoptosis inhibitor 5 OS=Homo sapiens GN=API5 PE=1 SV=3	API5_HUMAN (+1)	59 kDa	0.5	[]	0	0	1	0	1	2
Apoptosis-inducing factor 1, mitochondrial OS=Homo sapiens GN=AIFM1 PE=1 SV=1	AIFM1_HUMAN	67 kDa	0.69	[]	2	1	1	1	2	8
Archain 1, isoform CRA_b OS=Homo sapiens GN=ARCN1 PE=2 SV=1	B0YIW5_HUMAN (+3)	57 kDa	0.74	[]	0	1	2	1	1	4
Arf-GAP with Rho-GAP domain, ANK repeat and PH domain-containing protein 1 OS=Homo sapiens GN=ARAP1 PE=1 SV=3	ARAP1_HUMAN (+3)	162 kDa	0.37	[]	0	0	0	0	0	2
Arfaptin-1 OS=Homo sapiens GN=ARFIP1 PE=1 SV=2	ARFP1_HUMAN (+1)	42 kDa	0.12	[]	0	0	0	3	0	5
Arginine--tRNA ligase, cytoplasmic OS=Homo sapiens GN=RARS PE=1 SV=2	SYRC_HUMAN	75 kDa	0.32	[]	0	0	1	2	0	18
Argininosuccinate lyase OS=Homo sapiens GN=ASL PE=1 SV=4	ARLY_HUMAN (+2)	52 kDa	0.17	[]	0	0	0	0	1	3
Armadillo repeat protein deleted in velo-cardio-facial syndrome OS=Homo sapiens GN=ARVCF PE=1 SV=1	ARVC_HUMAN (+3)	105 kDa	0.0031	BRAC1 high, Naive low	0	0	0	25	24	19

Armadillo repeat-containing protein 10 OS=Homo sapiens GN=ARMC10 PE=1 SV=1	ARM10_HUMAN (+2)	38 kDa	0.37	[]	0	0	0	0	0	2
Asparagine--tRNA ligase, cytoplasmic OS=Homo sapiens GN=NARS PE=1 SV=1	SYNC_HUMAN	63 kDa	0.6	[]	1	0	1	0	2	4
Aspartate aminotransferase OS=Homo sapiens GN=GOT2 PE=3 SV=1	A0A024R6W0_HUMAN (+2)	47 kDa	0.031	BRAC1 low, Naive high	5	7	6	5	6	4
Aspartate aminotransferase OS=Homo sapiens PE=2 SV=1	A8K482_HUMAN	48 kDa	0.0056	BRAC1 low, Naive high	2	3	3	2	3	3
Aspartate aminotransferase, cytoplasmic OS=Homo sapiens GN=GOT1 PE=1 SV=3	AATC_HUMAN	46 kDa	0.37	[]	0	0	0	0	0	2
Aspartyl-tRNA synthetase, isoform CRA_b OS=Homo sapiens GN=DARS PE=3 SV=1	D3DP78_HUMAN (+1)	53 kDa	0.93	[]	2	2	2	2	1	8
Aspartyl/asparaginyl beta-hydroxylase OS=Homo sapiens GN=ASPH PE=1 SV=3	ASPH_HUMAN	86 kDa	0.1	[]	8	11	11	3	6	19
Ataxin-2-like protein OS=Homo sapiens GN=ATXN2L PE=1 SV=2	ATX2L_HUMAN (+3)	113 kDa	0.37	[]	0	0	0	0	0	2
Atlastin-3 OS=Homo sapiens GN=ATL3 PE=1 SV=1	ATLA3_HUMAN (+2)	61 kDa	0.78	[]	0	0	9	0	0	9
Autoantigen La (Fragment) OS=Homo sapiens GN=SSB PE=2 SV=1	B5BUB5_HUMAN (+1)	47 kDa	0.88	[]	0	0	2	1	1	1
Autophagy protein 5 OS=Homo sapiens GN=ATG5 PE=1 SV=2	ATG5_HUMAN (+2)	32 kDa	0.37	[]	0	0	0	0	0	2
B-cell receptor-associated protein 31 OS=Homo sapiens GN=BCAP31 PE=1 SV=3	BAP31_HUMAN (+1)	28 kDa	0.68	[]	1	0	3	1	2	6
BAG family molecular chaperone regulator 2 OS=Homo sapiens GN=BAG2 PE=1 SV=1	BAG2_HUMAN (+2)	24 kDa	0.2	[]	1	2	0	0	0	1
BRI3-binding protein OS=Homo sapiens GN=BRI3BP PE=1 SV=1	BRI3B_HUMAN	28 kDa	0.00029	BRAC1 high, Naive low	0	0	0	2	2	2
BRO1 domain-containing protein BROX OS=Homo sapiens GN=BROX PE=1 SV=1	BROX_HUMAN	46 kDa	0.12	[]	0	0	0	2	0	2
Band 4.1-like protein 1 OS=Homo sapiens GN=EPB41L1 PE=1 SV=1	A0A0C4DH22_HUMAN (+1)	98 kDa	0.042	BRAC1 high, Naive low	0	0	0	13	12	4
Band 4.1-like protein 2 OS=Homo sapiens GN=EPB41L2 PE=1 SV=1	E41L2_HUMAN (+5)	113 kDa	0.23	[]	0	1	5	0	0	0
Band 4.1-like protein 3 OS=Homo sapiens GN=EPB41L3 PE=1 SV=2	E41L3_HUMAN (+1)	121 kDa	0.066	[]	1	5	3	0	0	0
Band 4.1-like protein 5 OS=Homo sapiens GN=EPB41L5 PE=1 SV=3	E41L5_HUMAN (+1)	82 kDa	0.0079	BRAC1 high, Naive low	0	0	0	3	2	2
Basal cell adhesion molecule (Lutheran blood group) OS=Homo sapiens GN=BCAM PE=4 SV=1	A0A068W6H0_HUMAN (+6)	67 kDa	0.00019	BRAC1 high, Naive low	0	0	0	16	13	17
Basic leucine zipper and W2 domain-containing protein 1 OS=Homo sapiens GN=BZW1 PE=1 SV=1	BZW1_HUMAN (+2)	48 kDa	0.37	[]	0	0	0	0	0	4
Basic leucine zipper and W2 domains 2, isoform CRA_a OS=Homo sapiens GN=BZW2 PE=4 SV=1	A0A024RA42_HUMAN (+4)	48 kDa	0.13	[]	0	0	0	1	0	2
Beta 5-tubulin OS=Homo sapiens GN=TUBB PE=1 SV=1	Q5SU16_HUMAN (+1)	50 kDa	0.46	[]	25	30	34	37	42	64
Tubulin beta-2B chain OS=Homo sapiens GN=TUBB2B PE=1 SV=1	TBB2B_HUMAN	50 kDa	0.86	[]	16	24	25	30	31	49
Tubulin beta-6 chain OS=Homo sapiens GN=TUBB6 PE=1 SV=1	TBB6_HUMAN	50 kDa	0.14	[]	6	7	15	10	6	14
Tubulin beta-8 chain OS=Homo sapiens GN=TUBB8 PE=1 SV=1	A0A075B736_HUMAN (+2)	46 kDa	0.37	[]	0	0	8	0	0	0
cDNA FLJ11352 fis, clone HEMBA1000020, highly similar to Tubulin beta-2C chain OS=Homo sapiens PE=2 SV=1	B3KML9_HUMAN	45 kDa	0.86	[]	8	9	15	16	12	29
cDNA, FLJ93005, highly similar to Homo sapiens tubulin, beta polypeptide (TUBB), mRNA OS=Homo sapiens PE=2 SV=1	B2R6L0_HUMAN (+1)	50 kDa	0.46	[]	17	25	26	29	31	48
Beta globin (Fragment) OS=Homo sapiens PE=3 SV=1	B3VL05_HUMAN (+18)	11 kDa	0.011	BRAC1 low, Naive high	1	2	3	1	0	0
Beta-1-syntrophin OS=Homo sapiens GN=SNTB1 PE=1 SV=3	SNTB1_HUMAN	58 kDa	0.0015	BRAC1 high, Naive low	0	1	0	11	8	11
Beta-hexosaminidase subunit alpha OS=Homo sapiens GN=HEXA PE=1 SV=2	HEXA_HUMAN (+4)	61 kDa	0.37	[]	0	0	0	0	0	7
Beta-hexosaminidase subunit beta OS=Homo sapiens GN=HEXB PE=1 SV=3	HEXB_HUMAN (+1)	63 kDa	0.005	BRAC1 low, Naive high	3	4	4	0	1	3
Bifunctional 3'-phosphoadenosine 5'-phosphosulfate synthase 2 OS=Homo sapiens GN=PAPSS2 PE=1 SV=2	PAPS2_HUMAN (+2)	70 kDa	0.37	[]	0	0	2	0	0	0
Bifunctional coenzyme A synthase OS=Homo sapiens GN=COASY PE=1 SV=4	COASY_HUMAN	62 kDa	0.19	[]	0	0	0	1	1	8
Bifunctional glutamate/proline--tRNA ligase OS=Homo sapiens GN=EPRS PE=1 SV=5	SYEP_HUMAN	171 kDa	0.34	[]	0	1	1	2	1	30
Bifunctional purine biosynthesis protein PURH OS=Homo sapiens GN=ATIC PE=1 SV=3	PUR9_HUMAN (+1)	65 kDa	0.19	[]	0	0	3	7	3	29
cDNA FLJ57133, highly similar to Bifunctional purine biosynthesis protein PURH OS=Homo sapiens PE=2 SV=1	B4DP06_HUMAN	59 kDa	0.21	[]	0	0	3	5	3	25
Bone marrow stromal cell antigen 2, isoform CRA_a OS=Homo sapiens GN=BST2 PE=4 SV=1	A0A024R7H5_HUMAN (+2)	20 kDa	0.64	[]	1	0	2	2	2	2
Brain acid soluble protein 1 OS=Homo sapiens GN=BASP1 PE=1 SV=2	BASP1_HUMAN	23 kDa	0.00029	BRAC1 low, Naive high	14	14	18	0	0	0
Brain-specific angiogenesis inhibitor 1-associated protein 2 OS=Homo sapiens GN=BAIAP2 PE=1 SV=1	BAIP2_HUMAN (+1)	61 kDa	0.017	BRAC1 high, Naive low	0	0	0	15	16	8
Brain-specific angiogenesis inhibitor 1-associated protein 2-like protein 1 OS=Homo sapiens GN=BAIAP2L1 PE=1 SV=2	BI2L1_HUMAN	57 kDa	0.00029	BRAC1 high, Naive low	0	0	0	2	2	2
Brefeldin A-inhibited guanine nucleotide-exchange protein 1 OS=Homo sapiens GN=ARFGEF1 PE=1 SV=2	BIG1_HUMAN (+3)	209 kDa	0.37	[]	0	0	0	0	0	3
Brefeldin A-inhibited guanine nucleotide-exchange protein 2 OS=Homo sapiens GN=ARFGEF2 PE=1 SV=3	BIG2_HUMAN	202 kDa	0.37	[]	0	0	0	0	0	5
Bumetanide-sensitive Na-K-Cl cotransporter OS=Homo sapiens GN=SLC12A2 PE=2 SV=1	Q53ZR1_HUMAN (+1)	131 kDa	0.036	BRAC1 high, Naive low	0	1	0	48	55	19
C-1-tetrahydrofolate synthase, cytoplasmic OS=Homo sapiens GN=MTHFD1 PE=1 SV=3	C1TC_HUMAN (+1)	102 kDa	0.082	[]	1	3	3	6	6	14
C-terminal-binding protein 1 OS=Homo sapiens GN=CTBP1 PE=1 SV=2	CTBP1_HUMAN (+1)	48 kDa	0.37	[]	0	0	0	0	0	4
C-type mannose receptor 2 OS=Homo sapiens GN=MRC2 PE=1 SV=2	MRC2_HUMAN	167 kDa	0.0028	BRAC1 low, Naive high	7	13	11	0	0	0
CAAX prenyl protease 1 homolog OS=Homo sapiens GN=ZMPSTE24 PE=1 SV=2	FACE1_HUMAN (+1)	55 kDa	0.37	[]	0	0	0	0	0	4
CAD protein OS=Homo sapiens GN=CAD PE=1 SV=1	F8VPD4_HUMAN (+1)	236 kDa	0.019	BRAC1 high, Naive low	0	1	1	6	5	13
CCR4-NOT transcription complex subunit 1 OS=Homo sapiens GN=CNOT1 PE=1 SV=2	CNOT1_HUMAN	267 kDa	0.012	BRAC1 high, Naive low	0	0	0	3	5	3
CD2-associated protein OS=Homo sapiens GN=CD2AP PE=1 SV=1	CD2AP_HUMAN	71 kDa	0.17	[]	0	0	0	0	1	3
CD59 glycoprotein OS=Homo sapiens GN=CD59 PE=1 SV=1	CD59_HUMAN (+3)	14 kDa	0.89	[]	1	3	3	4	5	1
CD82 antigen OS=Homo sapiens GN=CD82 PE=1 SV=1	CD82_HUMAN (+1)	30 kDa	0.039	BRAC1 high, Naive low	0	0	0	3	3	1
CD99 antigen OS=Homo sapiens GN=CD99 PE=1 SV=1	CD99_HUMAN (+1)	19 kDa	0.014	BRAC1 low, Naive high	2	1	3	0	0	0
CD109 antigen OS=Homo sapiens GN=CD109 PE=1 SV=2	CD109_HUMAN	162 kDa	0.023	BRAC1 low, Naive high	16	15	9	0	0	0
CD133 isoform H OS=Homo sapiens GN=CD133 PE=2 SV=1	A0A0A0N0M1_HUMAN (+3)	96 kDa	0.004	BRAC1 high, Naive low	0	0	0	4	3	7
CD166 antigen OS=Homo sapiens GN=ALCAM PE=1 SV=2	CD166_HUMAN	65 kDa	0.011	BRAC1 low, Naive high	5	8	16	1	3	0
CD276 antigen OS=Homo sapiens GN=CD276 PE=1 SV=1	A0A0C4DGH0_HUMAN (+2)	42 kDa	0.84	[]	1	3	4	4	3	7
CDC42 effector protein (Rho GTPase binding) 4, isoform CRA_a OS=Homo sapiens GN=CDC42EP4 PE=2 SV=1	B2R6D8_HUMAN (+1)	38 kDa	0.028	BRAC1 high, Naive low	0	0	0	1	2	1
CDGSH iron-sulfur domain-containing protein 2 OS=Homo sapiens GN=CISD2 PE=1 SV=1	CISD2_HUMAN (+1)	15 kDa	0.37	[]	0	0	0	0	3	0
CDK5 regulatory subunit-associated protein 3 OS=Homo sapiens GN=CDK5RAP3 PE=1 SV=2	CK5P3_HUMAN	57 kDa	0.58	[]	0	2	1	0	1	2
COP9 signalosome complex subunit 2 OS=Homo sapiens GN=COPS2 PE=1 SV=1	CSN2_HUMAN (+4)	52 kDa	0.37	[]	0	0	2	0	0	0

CTD (Carboxy-terminal domain, RNA polymerase II, polypeptide A) small phosphatase 1, isoform CRA_b (Fragment) OS=Homo sapien:	A0A024R414_HUMAN (+2)	29 kDa	0.3	[]	0	0	1	2	2	0
CTP synthase 1 OS=Homo sapiens GN=CTPS1 PE=1 SV=2	PYRG1_HUMAN	67 kDa	0.67	[]	0	0	3	0	1	7
CUB domain containing protein 1, isoform CRA_a OS=Homo sapiens GN=CDCP1 PE=4 SV=1	A0A024R2T5_HUMAN (+1)	93 kDa	0.0027	BRAC1 high, Naive low	0	0	0	1	1	2
Cadherin-1 OS=Homo sapiens GN=CDH1 PE=1 SV=1	A0A087WXI5_HUMAN (+4)	100 kDa	0.048	BRAC1 high, Naive low	0	0	0	31	30	9
Cadherin-2 OS=Homo sapiens GN=CDH2 PE=1 SV=4	CADH2_HUMAN (+1)	100 kDa	0.005	BRAC1 low, Naive high	2	3	6	0	0	0
Cadherin-11 OS=Homo sapiens GN=CDH11 PE=2 SV=2	CAD11_HUMAN (+3)	88 kDa	0.37	[]	0	2	0	0	0	0
Cadherin-13 OS=Homo sapiens GN=CDH13 PE=1 SV=1	CAD13_HUMAN (+2)	78 kDa	0.069	[]	3	1	2	0	0	0
Calcium binding protein 39, isoform CRA_a OS=Homo sapiens GN=CAB39 PE=4 SV=1	A0A024R496_HUMAN (+3)	40 kDa	0.36	[]	0	1	1	2	2	1
Calcium-binding mitochondrial carrier protein Aralar2 OS=Homo sapiens GN=SLC25A13 PE=1 SV=2	CMC2_HUMAN (+1)	74 kDa	0.18	[]	0	2	0	2	3	8
Calcium-transporting ATPase OS=Homo sapiens GN=ATP2B1 PE=3 SV=1	A0A024RBC7_HUMAN (+1)	135 kDa	0.21	[]	14	19	17	24	21	12
Calcium-transporting ATPase OS=Homo sapiens GN=ATP2B4 PE=3 SV=1	A0A024R968_HUMAN (+1)	134 kDa	0.0017	BRAC1 low, Naive high	30	37	35	7	10	3
Calcium/calmodulin-dependent protein kinase (CaM kinase) II alpha, isoform CRA_b OS=Homo sapiens GN=CAMK2A PE=2 SV=1	A8K161_HUMAN (+3)	55 kDa	0.37	[]	0	0	0	1	0	0
Calcium/calmodulin-dependent protein kinase (CaM kinase) II delta, isoform CRA_a OS=Homo sapiens GN=CAMK2D PE=4 SV=1	A0A024RDK3_HUMAN (+5)	56 kDa	0.46	[]	0	3	4	3	5	8
Calcium/calmodulin-dependent protein kinase (CaM kinase) II gamma, isoform CRA_a OS=Homo sapiens GN=CAMK2G PE=1 SV=1	A0A0A0MS52_HUMAN (+8)	60 kDa	0.37	[]	0	0	0	0	0	3
Calcyclin-binding protein OS=Homo sapiens GN=CACYBP PE=1 SV=2	CYBP_HUMAN (+2)	26 kDa	0.37	[]	0	0	0	0	0	3
Calnexin OS=Homo sapiens GN=CANX PE=1 SV=2	CALX_HUMAN	68 kDa	0.94	[]	21	18	25	30	33	48
Calpain 1, (Mu/I) large subunit, isoform CRA_a OS=Homo sapiens GN=CAPN1 PE=4 SV=1	A0A024R580_HUMAN (+3)	82 kDa	0.86	[]	0	3	8	3	1	11
Calpain-1 catalytic subunit (Fragment) OS=Homo sapiens GN=CAPN1 PE=1 SV=1	E9PL37_HUMAN	21 kDa	0.37	[]	0	0	0	0	0	3
Calpain small subunit 1 OS=Homo sapiens GN=CAPNS1 PE=1 SV=1	A0A0C4DGQ5_HUMAN (+3)	34 kDa	0.94	[]	0	1	4	3	3	0
Calpain-5 OS=Homo sapiens GN=CAPN5 PE=1 SV=1	A0A087X0C8_HUMAN (+2)	73 kDa	0.017	BRAC1 high, Naive low	0	0	0	7	10	5
Calponin OS=Homo sapiens GN=CNN2 PE=1 SV=1	B4DUT8_HUMAN (+4)	36 kDa	0.39	[]	4	5	4	8	5	2
Calponin-1 OS=Homo sapiens GN=CNN1 PE=1 SV=2	CNN1_HUMAN (+2)	33 kDa	0.049	BRAC1 low, Naive high	1	1	4	0	0	0
Calponin-3 OS=Homo sapiens GN=CNN3 PE=1 SV=1	CNN3_HUMAN	36 kDa	0.63	[]	0	0	3	0	0	2
Calreticulin OS=Homo sapiens GN=CALR PE=1 SV=1	CALR_HUMAN (+2)	48 kDa	0.013	BRAC1 low, Naive high	27	26	31	16	15	33
cDNA FLJ53009, highly similar to Calreticulin OS=Homo sapiens PE=2 SV=1	B4DHR1_HUMAN	24 kDa	0.026	BRAC1 low, Naive high	22	21	20	13	10	21
Calumenin OS=Homo sapiens GN=CALU PE=1 SV=2	CALU_HUMAN (+2)	37 kDa	0.056	[]	8	4	14	0	0	9
tr|A0A024R755|A0A024R755_HUMAN Calumenin, isoform CRA_a OS=Homo sapiens GN=CALU PE=4...	A0A024R755_HUMAN (+1)	?	0.083	[]	5	3	11	0	0	8
Canalicular multispecific organic anion transporter 2 OS=Homo sapiens GN=ABCC3 PE=1 SV=3	MRP3_HUMAN	169 kDa	0.0059	BRAC1 high, Naive low	1	0	1	19	14	14
Capping protein (Actin filament) muscle Z-line, alpha 1, isoform CRA_a OS=Homo sapiens GN=CAPZA1 PE=4 SV=1	A0A024R0E5_HUMAN (+2)	33 kDa	0.16	[]	3	3	6	7	9	9
Capping protein (Actin filament) muscle Z-line, alpha 2 OS=Homo sapiens GN=CAPZA2 PE=2 SV=1	A4D0V4_HUMAN (+3)	33 kDa	0.0028	BRAC1 high, Naive low	1	2	2	5	6	7
Capping protein (Actin filament) muscle Z-line, beta, isoform CRA_a OS=Homo sapiens GN=CAPZB PE=1 SV=1	B1AK87_HUMAN (+2)	29 kDa	0.3	[]	1	1	4	5	3	6
Caprin-1 OS=Homo sapiens GN=CAPRIN1 PE=1 SV=1	A0A087X082_HUMAN (+1)	73 kDa	0.022	BRAC1 high, Naive low	0	0	0	1	3	3
Carbonic anhydrase 9 OS=Homo sapiens GN=CA9 PE=1 SV=2	CAH9_HUMAN	50 kDa	0.036	BRAC1 high, Naive low	0	0	0	7	11	4
Carbonic anhydrase 12 OS=Homo sapiens GN=CA12 PE=1 SV=1	CAH12_HUMAN	39 kDa	0.029	BRAC1 high, Naive low	0	0	0	15	11	6
Carbonyl reductase [NADPH] 1 OS=Homo sapiens GN=CBR1 PE=1 SV=3	CBR1_HUMAN	30 kDa	0.74	[]	1	2	1	1	3	2
Carboxypeptidase D OS=Homo sapiens GN=CPD PE=1 SV=2	CBPD_HUMAN	153 kDa	0.36	[]	0	1	1	2	2	1
Carcinoembryonic antigen-related cell adhesion molecule 1 OS=Homo sapiens GN=CEACAM1 PE=1 SV=2	CEAM1_HUMAN (+1)	58 kDa	0.0042	BRAC1 high, Naive low	0	0	0	5	7	12
Carcinoembryonic antigen-related cell adhesion molecule 5 OS=Homo sapiens GN=CEACAM5 PE=1 SV=1	A0A024R0K5_HUMAN (+6)	77 kDa	0.12	[]	0	0	0	2	2	0
Carcinoembryonic antigen-related cell adhesion molecule 6 OS=Homo sapiens GN=CEACAM6 PE=1 SV=3	CEAM6_HUMAN	37 kDa	0.16	[]	0	0	0	2	1	0
Carnitine O-acetyltransferase OS=Homo sapiens GN=CRAT PE=1 SV=5	CACP_HUMAN (+2)	71 kDa	0.37	[]	0	0	0	0	0	2
Carnitine O-palmitoyltransferase 1, liver isoform OS=Homo sapiens GN=CPT1A PE=1 SV=2	CPT1A_HUMAN (+2)	88 kDa	0.014	BRAC1 low, Naive high	1	1	2	0	1	0
Casein kinase I isoform alpha OS=Homo sapiens GN=CSNK1A1 PE=1 SV=2	KC1A_HUMAN (+1)	39 kDa	0.03	BRAC1 high, Naive low	0	0	0	1	1	3
Casein kinase I isoform gamma-3 OS=Homo sapiens GN=CSNK1G3 PE=1 SV=2	KC1G3_HUMAN	51 kDa	0.12	[]	0	0	0	2	2	0
Casein kinase II subunit alpha OS=Homo sapiens GN=CSNK2A1 PE=1 SV=1	CSK21_HUMAN (+2)	45 kDa	0.45	[]	0	0	2	1	2	2
Caspase-8 OS=Homo sapiens GN=CASP8 PE=1 SV=1	CASP8_HUMAN (+3)	55 kDa	0.37	[]	0	0	0	0	0	2
Catalase OS=Homo sapiens GN=CAT PE=1 SV=3	CATA_HUMAN (+1)	60 kDa	0.91	[]	5	4	5	3	9	15
Catechol O-methyltransferase OS=Homo sapiens GN=COMT PE=1 SV=2	COMT_HUMAN	30 kDa	0.38	[]	1	1	2	2	2	7
Catenin (Cadherin-associated protein), beta 1, 88kDa, isoform CRA_a OS=Homo sapiens GN=CTNNB1 PE=4 SV=1	A0A024R2Q3_HUMAN (+3)	85 kDa	0.04	BRAC1 high, Naive low	3	9	11	53	47	27
CTNNA2 protein OS=Homo sapiens GN=CTNNA2 PE=2 SV=1	Q49AD3_HUMAN	76 kDa	0.37	[]	0	0	0	0	10	0
Catenin alpha-1 OS=Homo sapiens GN=CTNNA1 PE=1 SV=1	CTNA1_HUMAN	100 kDa	0.041	BRAC1 high, Naive low	9	10	15	111	100	52
Catenin alpha-2 OS=Homo sapiens GN=CTNNA2 PE=1 SV=5	CTNA2_HUMAN	105 kDa	0.37	[]	0	0	0	10	0	0
Catenin delta-1 OS=Homo sapiens GN=CTNND1 PE=1 SV=2	C9JZR2_HUMAN (+2)	105 kDa	0.026	BRAC1 high, Naive low	5	9	14	95	88	50
Cathepsin B OS=Homo sapiens GN=CTSB PE=1 SV=3	CATB_HUMAN (+5)	38 kDa	0.37	[]	0	0	4	0	0	0
Cathepsin D OS=Homo sapiens GN=CTSD PE=1 SV=1	CATD_HUMAN (+2)	45 kDa	0.98	[]	3	2	0	2	4	4
Cathepsin Z OS=Homo sapiens GN=CTSZ PE=1 SV=1	CATZ_HUMAN (+1)	34 kDa	0.65	[]	0	1	0	0	2	1
Cation-dependent mannose-6-phosphate receptor OS=Homo sapiens GN=M6PR PE=1 SV=1	MPRD_HUMAN (+1)	31 kDa	0.66	[]	0	1	1	1	1	2
Caveolin-1 OS=Homo sapiens GN=CAV1 PE=1 SV=4	CAV1_HUMAN (+1)	20 kDa	0.003	BRAC1 low, Naive high	9	7	10	0	0	0
tr|A0A024R757|A0A024R757_HUMAN Caveolin OS=Homo sapiens GN=CAV1 PE=3 SV=1	A0A024R757_HUMAN (+2)	?	0.00055	BRAC1 low, Naive high	5	5	6	0	0	0
Cell cycle control protein 50B OS=Homo sapiens GN=TMEM30B PE=1 SV=1	CC50B_HUMAN	39 kDa	0.0027	BRAC1 high, Naive low	0	0	0	1	1	2
Cell division cycle 2, G1 to S and G2 to M, isoform CRA_c OS=Homo sapiens GN=CDC2 PE=4 SV=1	A0A024QZJ8_HUMAN (+3)	28 kDa	0.0014	BRAC1 high, Naive low	0	0	0	3	2	3
Cell division cycle 42 (GTP binding protein, 25kDa), isoform CRA_a OS=Homo sapiens GN=CDC42 PE=3 SV=1	A0A024RAE4_HUMAN (+2)	21 kDa	0.64	[]	3	10	11	18	15	11
Cell migration-inducing and hyaluronan-binding protein OS=Homo sapiens GN=CEMIP PE=1 SV=2	CEMIP_HUMAN	153 kDa	0.089	[]	4	3	18	0	0	0

Cell surface A33 antigen OS=Homo sapiens GN=GPA33 PE=1 SV=1	GPA33_HUMAN	36 kDa	0.16	[]	0	0	0	4	2	0
Centromere/kinetochore protein zw10 homolog OS=Homo sapiens GN=ZW10 PE=1 SV=3	ZW10_HUMAN (+1)	89 kDa	0.13	[]	0	0	0	0	1	2
Ceramide synthase 2 OS=Homo sapiens GN=CERS2 PE=1 SV=1	CERS2_HUMAN (+1)	45 kDa	0.6	[]	0	0	1	0	0	4
Ceramide synthase 6 OS=Homo sapiens GN=CERS6 PE=1 SV=1	CERS6_HUMAN	45 kDa	0.13	[]	0	0	0	1	0	2
Chaperonin containing TCP1, subunit 6A (Zeta 1), isoform CRA_a OS=Homo sapiens GN=CCT6A PE=3 SV=1	A0A024RDL1_HUMAN (+3)	58 kDa	0.057	[]	0	1	6	8	10	20
Chaperonin containing TCP1, subunit 7 (Eta) variant (Fragment) OS=Homo sapiens PE=2 SV=1	Q53HV2_HUMAN (+1)	59 kDa	0.22	[]	1	2	10	10	9	17
Chaperonin containing TCP1, subunit 8 (Theta) variant (Fragment) OS=Homo sapiens PE=2 SV=1	Q53HU0_HUMAN (+1)	60 kDa	0.67	[]	3	4	7	8	8	8
Charged multivesicular body protein 2a OS=Homo sapiens GN=CHMP2A PE=1 SV=1	CHM2A_HUMAN (+1)	25 kDa	0.37	[]	0	0	0	0	0	3
Charged multivesicular body protein 4a OS=Homo sapiens GN=CHMP4A PE=1 SV=3	CHM4A_HUMAN (+4)	25 kDa	0.37	[]	0	0	0	0	0	2
Charged multivesicular body protein 4b OS=Homo sapiens GN=CHMP4B PE=1 SV=1	CHM4B_HUMAN	25 kDa	0.94	[]	2	2	0	2	1	6
Chitobiosyldiphosphodolichol beta-mannosyltransferase OS=Homo sapiens GN=ALG1 PE=1 SV=2	ALG1_HUMAN	53 kDa	0.82	[]	0	0	2	0	1	1
Chloride intracellular channel protein 1 OS=Homo sapiens GN=CLIC1 PE=1 SV=4	CLIC1_HUMAN (+2)	27 kDa	0.14	[]	2	6	5	15	12	10
Chloride intracellular channel protein 3 OS=Homo sapiens GN=CLIC3 PE=1 SV=2	CLIC3_HUMAN (+1)	27 kDa	0.23	[]	0	0	0	0	1	5
Chloride intracellular channel protein OS=Homo sapiens PE=2 SV=1	B4DWC4_HUMAN (+2)	28 kDa	0.97	[]	2	3	5	8	5	2
Chondroitin sulfate proteoglycan 4 OS=Homo sapiens GN=CSPG4 PE=1 SV=2	CSPG4_HUMAN	251 kDa	0.056	[]	14	24	6	0	0	0
Chromosome 6 open reading frame 55, isoform CRA_b OS=Homo sapiens GN=VTA1 PE=1 SV=1	A0A087WY55_HUMAN (+1)	31 kDa	0.1	[]	1	0	0	2	2	3
Chromosome 7 open reading frame 24 OS=Homo sapiens GN=C7orf24 PE=4 SV=1	A0A090N7V5_HUMAN (+2)	21 kDa	0.12	[]	0	0	0	2	2	0
Chromosome 9 open reading frame 88, isoform CRA_a OS=Homo sapiens GN=C9orf88 PE=4 SV=1	A0A024R872_HUMAN (+1)	83 kDa	0.031	BRAC1 high, Naive low	2	2	6	35	29	18
Chromosome-associated kinesin KIF4A OS=Homo sapiens GN=KIF4A PE=1 SV=3	KIF4A_HUMAN (+2)	140 kDa	0.19	[]	0	0	0	2	0	1
Cingulin OS=Homo sapiens GN=CGN PE=2 SV=1	B9EK46_HUMAN (+1)	137 kDa	0.042	BRAC1 high, Naive low	0	0	0	3	2	1
Citrate synthase OS=Homo sapiens GN=CS PE=1 SV=1	A0A0C4DGI3_HUMAN	45 kDa	0.34	[]	4	3	5	5	4	8
Citron Rho-interacting kinase OS=Homo sapiens GN=CIT PE=1 SV=2	CTRO_HUMAN	231 kDa	0.12	[]	0	0	0	2	2	0
Clarin-3 OS=Homo sapiens GN=CLRN3 PE=2 SV=1	CLRN3_HUMAN	25 kDa	0.028	BRAC1 high, Naive low	0	0	0	1	2	1
Clathrin heavy chain OS=Homo sapiens GN=CLTC PE=1 SV=1	A0A087WVQ6_HUMAN (+1)	192 kDa	0.14	[]	19	29	51	21	31	55
Clathrin interactor 1 OS=Homo sapiens GN=CLINT1 PE=1 SV=1	EPN4_HUMAN	68 kDa	0.21	[]	0	0	0	0	1	4
Claudin OS=Homo sapiens PE=2 SV=1	B3KW38_HUMAN (+3)	21 kDa	0.37	[]	0	0	0	0	0	2
Claudin-3 OS=Homo sapiens GN=CLDN3 PE=1 SV=1	CLD3_HUMAN (+1)	23 kDa	0.047	BRAC1 high, Naive low	0	0	0	7	7	2
Cleft lip and palate transmembrane protein 1 OS=Homo sapiens GN=CLPTM1 PE=1 SV=1	CLPT1_HUMAN	76 kDa	0.72	[]	1	3	2	2	4	6
Coagulation factor V OS=Homo sapiens GN=F5 PE=1 SV=1	A0A0A0MRJ7_HUMAN (+1)	252 kDa	0.4	[]	1	1	13	2	0	5
Coatomer protein complex, subunit zeta 1, isoform CRA_a OS=Homo sapiens GN=COPZ1 PE=4 SV=1	A0A024RB72_HUMAN (+3)	20 kDa	0.13	[]	0	0	0	1	0	2
Coatomer subunit alpha OS=Homo sapiens GN=COPA PE=1 SV=2	COPA_HUMAN	138 kDa	0.58	[]	2	2	4	3	2	18
Coatomer subunit beta OS=Homo sapiens GN=COPB1 PE=1 SV=3	COPB_HUMAN	107 kDa	0.32	[]	0	0	4	3	2	13
Coatomer subunit beta' OS=Homo sapiens GN=COPB2 PE=1 SV=2	COPB2_HUMAN	102 kDa	0.29	[]	0	0	2	1	2	11
Coatomer subunit epsilon OS=Homo sapiens GN=COPE PE=1 SV=3	COPE_HUMAN (+3)	34 kDa	0.87	[]	0	0	1	0	0	2
Coatomer subunit gamma-1 OS=Homo sapiens GN=COPG1 PE=1 SV=1	COPG1_HUMAN	98 kDa	0.34	[]	1	0	1	2	1	19
Coatomer subunit gamma-2 OS=Homo sapiens GN=COPG2 PE=1 SV=1	COPG2_HUMAN	98 kDa	0.51	[]	1	0	0	0	0	10
Cofilin-1 OS=Homo sapiens GN=CFL1 PE=1 SV=3	COF1_HUMAN (+1)	19 kDa	0.25	[]	7	5	6	19	24	10
Cofilin-2 OS=Homo sapiens GN=CFL2 PE=1 SV=1	COF2_HUMAN (+1)	19 kDa	0.24	[]	1	3	3	0	4	0
Coiled-coil domain-containing protein 47 OS=Homo sapiens GN=CCDC47 PE=1 SV=1	CCD47_HUMAN	56 kDa	0.23	[]	0	1	1	1	2	4
Coiled-coil-helix-coiled-coil-helix domain containing 3 OS=Homo sapiens GN=CHCHD3 PE=4 SV=1	A4D1N4_HUMAN (+4)	26 kDa	0.36	[]	2	0	2	1	1	1
Cold shock domain-containing protein E1 OS=Homo sapiens GN=CSDE1 PE=1 SV=2	CSDE1_HUMAN (+4)	89 kDa	0.37	[]	0	0	0	0	0	2
Collagen alpha-1(I) chain OS=Homo sapiens GN=COL1A1 PE=1 SV=5	CO1A1_HUMAN	139 kDa	< 0.00010	BRAC1 low, Naive high	12	15	18	0	0	0
tr|D3DTX7|D3DTX7_HUMAN Collagen, type I, alpha 1, isoform CRA_a OS=Homo sapiens GN=COL1A1...	D3DTX7_HUMAN	?	< 0.00010	BRAC1 low, Naive high	10	12	17	0	0	0
Collagen alpha-1(VI) chain OS=Homo sapiens GN=COL6A1 PE=1 SV=3	CO6A1_HUMAN (+1)	109 kDa	0.00019	BRAC1 low, Naive high	7	8	9	0	0	0
Collagen alpha-1(XII) chain OS=Homo sapiens GN=COL12A1 PE=1 SV=2	COCA1_HUMAN (+1)	333 kDa	0.12	[]	10	13	0	0	0	0
Collagen alpha-2(I) chain OS=Homo sapiens GN=COL1A2 PE=1 SV=7	CO1A2_HUMAN (+1)	129 kDa	0.00018	BRAC1 low, Naive high	6	7	12	0	0	0
Collagen alpha-2(VI) chain OS=Homo sapiens GN=COL6A2 PE=1 SV=4	CO6A2_HUMAN	109 kDa	0.022	BRAC1 low, Naive high	3	6	3	0	0	0
Collagen alpha-3(VI) chain OS=Homo sapiens GN=COL6A3 PE=1 SV=5	CO6A3_HUMAN (+1)	344 kDa	0.043	BRAC1 low, Naive high	39	52	16	0	0	0
tr|B7ZW00|B7ZW00_HUMAN COL6A3 protein OS=Homo sapiens GN=COL6A3 PE=2 SV=1	B7ZW00_HUMAN (+1)	?	0.045	BRAC1 low, Naive high	38	50	15	0	0	0
Collectin-12 OS=Homo sapiens GN=COLEC12 PE=1 SV=3	COL12_HUMAN	82 kDa	0.0064	BRAC1 low, Naive high	2	3	2	0	0	0
Complement C5 OS=Homo sapiens GN=C5 PE=1 SV=4	CO5_HUMAN (+1)	188 kDa	0.0044	BRAC1 low, Naive high	3	2	4	0	0	0
Complement component 9, isoform CRA_a OS=Homo sapiens GN=C9 PE=4 SV=1	A0A024R035_HUMAN (+1)	63 kDa	0.14	[]	5	5	1	2	2	0
Complement decay-accelerating factor OS=Homo sapiens GN=CD55 PE=1 SV=1	B1AP13_HUMAN (+3)	49 kDa	< 0.00010	BRAC1 high, Naive low	0	0	0	5	6	8
Conserved oligomeric Golgi complex subunit 1 OS=Homo sapiens GN=COG1 PE=1 SV=1	COG1_HUMAN (+4)	109 kDa	0.37	[]	0	0	0	0	0	3
Conserved oligomeric Golgi complex subunit 2 OS=Homo sapiens GN=COG2 PE=1 SV=1	COG2_HUMAN (+2)	83 kDa	0.37	[]	0	0	0	0	0	3
Conserved oligomeric Golgi complex subunit 3 OS=Homo sapiens GN=COG3 PE=1 SV=3	COG3_HUMAN	94 kDa	0.13	[]	0	0	0	1	0	2
Conserved oligomeric Golgi complex subunit 5 OS=Homo sapiens GN=COG5 PE=1 SV=3	COG5_HUMAN	93 kDa	0.37	[]	0	0	0	0	0	2
Conserved oligomeric Golgi complex subunit 7 OS=Homo sapiens GN=COG7 PE=1 SV=1	COG7_HUMAN	86 kDa	0.37	[]	0	0	0	0	0	3
Constitutive coactivator of PPAR-gamma-like protein 1 OS=Homo sapiens GN=FAM120A PE=1 SV=2	F120A_HUMAN	122 kDa	0.042	BRAC1 low, Naive high	1	2	2	0	1	2
Copine-1 OS=Homo sapiens GN=CPNE1 PE=1 SV=1	B0QZ18_HUMAN (+1)	60 kDa	0.042	BRAC1 high, Naive low	0	0	4	8	6	8
Copine-3 OS=Homo sapiens GN=CPNE3 PE=1 SV=1	CPNE3_HUMAN (+1)	60 kDa	0.093	[]	1	6	7	0	0	3
Core histone macro-H2A.1 OS=Homo sapiens GN=H2AFY PE=1 SV=4	H2AY_HUMAN	40 kDa	0.71	[]	1	0	2	1	1	5

Coronin OS=Homo sapiens GN=CORO1B PE=3 SV=1	A0A024R5K1_HUMAN (+1)	54 kDa	0.14	[]	1	2	2	8	9	3
Coronin OS=Homo sapiens GN=CORO1C PE=3 SV=1	A0A024RBI5_HUMAN (+4)	53 kDa	0.11	[]	8	12	12	15	10	7
Cortactin isoform a variant (Fragment) OS=Homo sapiens PE=1 SV=1	Q53HG7_HUMAN (+1)	62 kDa	0.45	[]	1	3	11	11	10	10
cDNA FLJ54828, highly similar to Src substrate cortactin OS=Homo sapiens PE=2 SV=1	B4E358_HUMAN	35 kDa	0.37	[]	0	0	0	3	0	0
Creatine kinase B-type OS=Homo sapiens GN=CKB PE=1 SV=1	KCRB_HUMAN (+2)	43 kDa	0.37	[]	0	0	0	0	0	5
Crk-like protein OS=Homo sapiens GN=CRKL PE=1 SV=1	CRKL_HUMAN	34 kDa	0.37	[]	0	0	0	0	0	3
Cullin-4B OS=Homo sapiens GN=CUL4B PE=1 SV=4	CUL4B_HUMAN (+1)	104 kDa	0.37	[]	0	0	3	0	0	0
Cysteine and glycine-rich protein 2 OS=Homo sapiens GN=CSRP2 PE=1 SV=3	CSRP2_HUMAN (+1)	21 kDa	0.017	BRAC1 low, Naive high	1	1	3	0	0	0
Cysteine-rich protein 2 OS=Homo sapiens GN=CRIP2 PE=1 SV=1	CRIP2_HUMAN (+2)	22 kDa	0.01	BRAC1 high, Naive low	0	0	0	5	5	3
Cystine/glutamate transporter OS=Homo sapiens GN=SLC7A11 PE=1 SV=1	XCT_HUMAN	55 kDa	0.37	[]	0	0	0	0	0	2
Cytochrome P450 2S1 OS=Homo sapiens GN=CYP2S1 PE=1 SV=2	CP2S1_HUMAN	56 kDa	0.031	BRAC1 high, Naive low	0	0	0	2	1	1
Cytochrome b5 OS=Homo sapiens GN=CYB5A PE=1 SV=2	CYB5_HUMAN (+2)	15 kDa	0.98	[]	1	1	1	2	2	1
Cytochrome b5 type B OS=Homo sapiens GN=CYB5B PE=1 SV=2	CYB5B_HUMAN (+3)	16 kDa	0.38	[]	0	1	3	2	2	9
Cytochrome b reductase 1 OS=Homo sapiens GN=CYBRD1 PE=1 SV=1	CYBR1_HUMAN	32 kDa	0.37	[]	0	0	2	0	0	0
Cytochrome b-c1 complex subunit 1, mitochondrial OS=Homo sapiens GN=UQCRC1 PE=1 SV=3	QCR1_HUMAN	53 kDa	0.96	[]	1	3	7	1	7	10
Cytochrome b-c1 complex subunit 2, mitochondrial OS=Homo sapiens GN=UQCRC2 PE=1 SV=1	H3BRG4_HUMAN (+1)	45 kDa	0.77	[]	1	3	10	3	5	12
Cytochrome b-c1 complex subunit Rieske, mitochondrial OS=Homo sapiens GN=UQCRFS1 PE=1 SV=2	UCRI_HUMAN	30 kDa	0.55	[]	0	0	2	0	0	1
Cytochrome c1, heme protein, mitochondrial OS=Homo sapiens GN=CYC1 PE=1 SV=3	CY1_HUMAN (+1)	35 kDa	0.47	[]	1	0	1	1	1	5
Cytochrome c (Fragment) OS=Homo sapiens GN=CYCS PE=1 SV=1	C9JFR7_HUMAN (+3)	11 kDa	0.0075	BRAC1 low, Naive high	2	2	2	1	0	0
Cytochrome c oxidase subunit 2 OS=Homo sapiens GN=COX2 PE=3 SV=1	A0A023QZ25_HUMAN (+258)	26 kDa	0.44	[]	2	0	1	1	1	0
Cytochrome c oxidase subunit 5B, mitochondrial OS=Homo sapiens GN=COX5B PE=1 SV=2	COX5B_HUMAN (+2)	14 kDa	0.37	[]	0	0	0	0	2	0
Cytochrome c oxidase subunit 7A2, mitochondrial OS=Homo sapiens GN=COX7A2 PE=1 SV=1	CX7A2_HUMAN (+4)	9 kDa	0.37	[]	0	0	0	0	2	0
Cytoplasmic FMR1-interacting protein 1 (Fragment) OS=Homo sapiens GN=CYFIP1 PE=1 SV=1	A0A087WVZ5_HUMAN (+1)	21 kDa	0.37	[]	0	0	0	0	1	0
Cytoplasmic FMR1-interacting protein 1 OS=Homo sapiens GN=CYFIP1 PE=1 SV=1	CYFP1_HUMAN (+1)	145 kDa	0.15	[]	3	2	4	17	16	5
Cytoplasmic FMR1-interacting protein 2 OS=Homo sapiens GN=CYFIP2 PE=1 SV=1	E7EVJ5_HUMAN	143 kDa	0.032	BRAC1 high, Naive low	0	0	0	8	6	3
Cytoplasmic dynein 1 heavy chain 1 OS=Homo sapiens GN=DYNC1H1 PE=1 SV=5	DYHC1_HUMAN	532 kDa	0.58	[]	7	18	6	10	9	24
Cytoskeleton-associated protein 4, isoform CRA_c OS=Homo sapiens GN=CKAP4 PE=4 SV=1	A0A024RBH2_HUMAN (+1)	66 kDa	0.017	BRAC1 low, Naive high	57	77	42	4	7	7
Cytoskeleton-associated protein 5 OS=Homo sapiens GN=CKAP5 PE=1 SV=3	CKAP5_HUMAN	226 kDa	0.15	[]	0	1	1	2	2	2
Cytosol aminopeptidase OS=Homo sapiens GN=LAP3 PE=1 SV=3	AMPL_HUMAN (+1)	56 kDa	0.62	[]	1	1	3	2	1	10
Cytosolic 10-formyltetrahydrofolate dehydrogenase OS=Homo sapiens GN=ALDH1L1 PE=1 SV=2	AL1L1_HUMAN (+2)	99 kDa	0.37	[]	0	0	0	0	0	2
Cytosolic purine 5'-nucleotidase OS=Homo sapiens GN=NT5C2 PE=1 SV=1	5NTC_HUMAN (+1)	65 kDa	0.87	[]	0	0	1	0	0	2
D-3-phosphoglycerate dehydrogenase OS=Homo sapiens GN=PHGDH PE=1 SV=4	SERA_HUMAN	57 kDa	0.91	[]	2	2	3	1	4	7
DBH-like monooxygenase protein 1 OS=Homo sapiens GN=MOXD1 PE=1 SV=1	MOXD1_HUMAN	70 kDa	0.12	[]	1	0	2	0	0	0
DDRGK domain-containing protein 1 OS=Homo sapiens GN=DDRGK1 PE=1 SV=2	DDRGK_HUMAN	36 kDa	0.58	[]	1	4	2	2	1	6
DEAD (Asp-Glu-Ala-Asp) box polypeptide 48, isoform CRA_a OS=Homo sapiens GN=DDX48 PE=3 SV=1	A0A024R8W0_HUMAN (+1)	47 kDa	0.38	[]	1	1	2	2	2	8
DERP12 (Dermal papilla derived protein 12) OS=Homo sapiens GN=derp12 PE=2 SV=1	Q8TE01_HUMAN	38 kDa	0.027	BRAC1 low, Naive high	25	29	16	7	7	15
DNA-(apurinic or apyrimidinic site) lyase OS=Homo sapiens GN=APEX1 PE=1 SV=2	APEX1_HUMAN (+3)	36 kDa	0.07	[]	0	0	1	1	2	3
DNA-dependent protein kinase catalytic subunit OS=Homo sapiens GN=PRKDC PE=1 SV=3	PRKDC_HUMAN	469 kDa	0.045	BRAC1 high, Naive low	0	0	0	1	4	6
Dedicator of cytokinesis protein 7 OS=Homo sapiens GN=DOCK7 PE=1 SV=4	DOCK7_HUMAN	243 kDa	0.04	BRAC1 high, Naive low	0	0	0	2	3	1
Dehydrogenase/reductase SDR family member 7B OS=Homo sapiens GN=DHRS7B PE=1 SV=2	DRS7B_HUMAN (+2)	35 kDa	0.37	[]	0	0	0	0	0	2
Delta(3,5)-Delta(2,4)-dienoyl-CoA isomerase, mitochondrial OS=Homo sapiens GN=ECH1 PE=1 SV=2	ECH1_HUMAN	36 kDa	0.12	[]	2	0	2	3	9	9
Delta(14)-sterol reductase OS=Homo sapiens GN=TM7SF2 PE=2 SV=3	ERG24_HUMAN (+2)	46 kDa	0.37	[]	0	0	0	0	0	2
Delta(24)-sterol reductase OS=Homo sapiens GN=DHCR24 PE=1 SV=2	DHC24_HUMAN (+1)	60 kDa	0.17	[]	0	0	0	1	0	3
Delta-1-pyrroline-5-carboxylate synthase OS=Homo sapiens GN=ALDH18A1 PE=1 SV=2	P5CS_HUMAN	87 kDa	0.04	BRAC1 low, Naive high	4	4	5	5	1	3
Delta-sarcoglycan OS=Homo sapiens GN=SGCD PE=1 SV=2	SGCD_HUMAN	32 kDa	0.00091	BRAC1 low, Naive high	1	1	2	0	0	0
Deoxynucleoside triphosphate triphosphohydrolase SAMHD1 OS=Homo sapiens GN=SAMHD1 PE=1 SV=2	SAMH1_HUMAN	72 kDa	0.22	[]	0	1	2	0	0	1
Deoxyribonuclease-1-like 1 (Fragment) OS=Homo sapiens GN=DNASE1L1 PE=1 SV=1	A6QRJ0_HUMAN (+2)	24 kDa	0.48	[]	1	0	3	2	0	1
Desmoglein-2 OS=Homo sapiens GN=DSG2 PE=1 SV=2	DSG2_HUMAN	122 kDa	0.023	BRAC1 high, Naive low	0	0	0	11	11	5
Desmoplakin OS=Homo sapiens GN=DSP PE=1 SV=3	DESP_HUMAN	332 kDa	0.028	BRAC1 high, Naive low	0	0	0	2	4	2
Developmentally-regulated GTP-binding protein 1 OS=Homo sapiens GN=DRG1 PE=1 SV=1	DRG1_HUMAN	41 kDa	0.013	BRAC1 high, Naive low	0	0	0	2	3	6
Dihydrolipoamide S-succinyltransferase (E2 component of 2-oxo-glutarate complex), isoform CRA_a OS=Homo sapiens GN=DLST PE=3	A0A024R6C9_HUMAN (+1)	49 kDa	0.0092	BRAC1 low, Naive high	4	3	5	1	1	3
Dihydrolipoyl dehydrogenase OS=Homo sapiens GN=DLD PE=4 SV=1	A0A024R713_HUMAN (+3)	54 kDa	0.074	[]	2	2	5	2	3	2
Dihydropyrimidinase-related protein 2 OS=Homo sapiens GN=DPYSL2 PE=1 SV=1	DPYL2_HUMAN (+1)	62 kDa	0.35	[]	0	1	5	1	0	1
Dihydroxyacetone phosphate acyltransferase OS=Homo sapiens GN=GNPAT PE=1 SV=1	GNPAT_HUMAN (+1)	77 kDa	0.37	[]	0	0	0	0	0	9
Dimethylarginine dimethylaminohydrolase 1, isoform CRA_b OS=Homo sapiens GN=DDAH1 PE=2 SV=1	B1AKK2_HUMAN (+1)	31 kDa	0.93	[]	0	2	0	1	1	2
Dinucleotide oxidase disulfide thiol exchanger 3 superfamily member 3 OS=Homo sapiens PE=2 SV=1	A0A024QYS2_HUMAN (+2)	68 kDa	0.4	[]	0	1	1	1	1	5
Dipeptidase 1 OS=Homo sapiens GN=DPEP1 PE=1 SV=3	DPEP1_HUMAN (+1)	46 kDa	0.001	BRAC1 high, Naive low	0	0	0	6	9	9
Dipeptidyl peptidase 2 OS=Homo sapiens GN=DPP7 PE=1 SV=3	DPP2_HUMAN	54 kDa	0.37	[]	0	0	0	0	0	2
Dipeptidyl peptidase 3 OS=Homo sapiens GN=DPP3 PE=1 SV=2	DPP3_HUMAN (+3)	83 kDa	0.00037	BRAC1 high, Naive low	0	0	0	4	3	5
Dipeptidyl peptidase 4 OS=Homo sapiens GN=DPP4 PE=1 SV=2	DPP4_HUMAN	88 kDa	0.031	BRAC1 low, Naive high	3	3	6	2	4	1
Discoidin domain receptor family, member 1, isoform CRA_b OS=Homo sapiens GN=DDR1 PE=4 SV=1	A0A024RCJ0_HUMAN (+3)	102 kDa	0.039	BRAC1 high, Naive low	0	0	0	3	3	1
Discoidin domain-containing receptor 2 OS=Homo sapiens GN=DDR2 PE=1 SV=2	DDR2_HUMAN (+2)	97 kDa	0.0036	BRAC1 low, Naive high	1	2	3	0	0	0

Disintegrin and metalloproteinase domain-containing protein 15 OS=Homo sapiens GN=ADAM15 PE=1 SV=4	ADA15_HUMAN (+2)	93 kDa	0.16	[]	0	0	0	1	2	0
Disks large homolog 1 OS=Homo sapiens GN=DLG1 PE=1 SV=1	A0A0C4DFT3_HUMAN	100 kDa	0.01	BRAC1 high, Naive low	1	2	1	10	8	7
Disks large homolog 3 OS=Homo sapiens GN=DLG3 PE=1 SV=2	DLG3_HUMAN (+2)	90 kDa	0.063	[]	0	0	0	4	5	1
DnaJ (Hsp40) homolog, subfamily A, member 2, isoform CRA_a OS=Homo sapiens GN=DNAJA2 PE=3 SV=1	A0A024R6S1_HUMAN (+1)	46 kDa	0.17	[]	0	0	0	1	1	7
DnaJ (Hsp40) homolog, subfamily C, member 3 OS=Homo sapiens GN=DNAJC3 PE=2 SV=1	A8KA82_HUMAN (+1)	58 kDa	0.16	[]	0	1	1	1	4	4
DnaJ homolog subfamily A member 1 OS=Homo sapiens GN=DNAJA1 PE=1 SV=2	DNJA1_HUMAN	45 kDa	0.044	BRAC1 high, Naive low	0	0	1	2	4	8
DnaJ homolog subfamily A member 3, mitochondrial OS=Homo sapiens GN=DNAJA3 PE=1 SV=2	DNJA3_HUMAN (+3)	52 kDa	0.37	[]	0	0	0	0	0	3
DnaJ homolog subfamily B member 4 OS=Homo sapiens GN=DNAJB4 PE=1 SV=1	DNJB4_HUMAN (+1)	38 kDa	0.37	[]	0	0	2	0	0	0
DnaJ homolog subfamily B member 11 OS=Homo sapiens GN=DNAJB11 PE=1 SV=1	DJB11_HUMAN	41 kDa	0.39	[]	0	1	2	1	2	7
DnaJ homolog subfamily C member 2 OS=Homo sapiens GN=DNAJC2 PE=1 SV=4	DNJC2_HUMAN	72 kDa	0.37	[]	0	0	0	0	0	3
DnaJ homolog subfamily C member 5 OS=Homo sapiens GN=DNAJC5 PE=1 SV=1	DNJC5_HUMAN	22 kDa	0.99	[]	0	0	4	1	0	5
DnaJ homolog subfamily C member 10 OS=Homo sapiens GN=DNAJC10 PE=1 SV=2	DJC10_HUMAN	91 kDa	0.37	[]	0	0	1	1	0	7
DnaJ homolog subfamily C member 11 OS=Homo sapiens GN=DNAJC11 PE=1 SV=2	DJC11_HUMAN	63 kDa	0.73	[]	0	0	2	0	2	2
DnaJ homolog subfamily C member 13 OS=Homo sapiens GN=DNAJC13 PE=1 SV=5	DJC13_HUMAN (+1)	254 kDa	0.35	[]	0	1	3	0	0	2
Dolichol-phosphate mannosyltransferase subunit 1 OS=Homo sapiens GN=DPM1 PE=1 SV=1	DPM1_HUMAN (+2)	30 kDa	0.45	[]	0	0	2	1	1	4
Dolichyl-diphosphooligosaccharide--protein glycosyltransferase 48 kDa subunit OS=Homo sapiens GN=DDOST PE=3 SV=1	A0A024RAD5_HUMAN (+2)	51 kDa	0.5	[]	8	7	8	7	10	18
Dolichyl-diphosphooligosaccharide--protein glycosyltransferase subunit 1 OS=Homo sapiens GN=RPN1 PE=1 SV=1	RPN1_HUMAN	69 kDa	0.27	[]	11	20	21	15	20	35
Dolichyl-diphosphooligosaccharide--protein glycosyltransferase subunit 2 OS=Homo sapiens GN=RPN2 PE=1 SV=3	RPN2_HUMAN	69 kDa	0.86	[]	7	16	17	14	23	26
Dolichyl-diphosphooligosaccharide--protein glycosyltransferase subunit STT3B OS=Homo sapiens GN=STT3B PE=1 SV=1	STT3B_HUMAN	94 kDa	0.056	[]	0	1	1	2	3	3
Dolichyl-phosphate beta-glucosyltransferase OS=Homo sapiens GN=ALG5 PE=1 SV=1	ALG5_HUMAN (+2)	37 kDa	0.5	[]	0	0	1	0	1	2
Drebrin OS=Homo sapiens GN=DBN1 PE=1 SV=4	DREB_HUMAN	71 kDa	0.039	BRAC1 low, Naive high	3	5	6	4	5	2
Drebrin-like protein OS=Homo sapiens GN=DBNL PE=1 SV=1	DBNL_HUMAN	48 kDa	0.29	[]	3	3	4	3	6	3
Drebrin-like protein OS=Homo sapiens GN=DBNL PE=1 SV=1	B4DDD6_HUMAN	46 kDa	0.66	[]	2	3	3	3	6	2
Drug-sensitive protein 1 OS=Homo sapiens GN=YA61 PE=2 SV=1	Q9NZ23_HUMAN	15 kDa	0.046	BRAC1 low, Naive high	39	33	20	5	6	8
Dynactin 2 (P50), isoform CRA_b OS=Homo sapiens GN=DCTN2 PE=2 SV=1	A8K8J9_HUMAN (+4)	34 kDa	0.0013	BRAC1 low, Naive high	2	2	3	1	0	0
Dynactin subunit 1 OS=Homo sapiens GN=DCTN1 PE=1 SV=3	DCTN1_HUMAN (+2)	142 kDa	0.12	[]	1	1	5	1	0	0
Dynamin-1 OS=Homo sapiens GN=DNM1 PE=1 SV=1	A0A0D9SFB1_HUMAN (+3)	94 kDa	0.01	BRAC1 high, Naive low	0	0	0	2	4	3
Dynamin-2 OS=Homo sapiens GN=DNM2 PE=1 SV=2	DYN2_HUMAN	98 kDa	0.00022	BRAC1 high, Naive low	0	0	1	13	11	18
Dynamin-like 120 kDa protein, mitochondrial OS=Homo sapiens GN=OPA1 PE=1 SV=1	E5KLJ5_HUMAN (+11)	118 kDa	0.5	[]	0	0	1	2	0	1
Dynein assembly factor 5, axonemal OS=Homo sapiens GN=DNAAF5 PE=1 SV=4	DAAF5_HUMAN	94 kDa	0.25	[]	0	0	0	0	1	6
Cytoplasmic dynein 1 light intermediate chain 1 OS=Homo sapiens GN=DYNC1LI1 PE=1 SV=3	DC1L1_HUMAN (+3)	57 kDa	0.37	[]	0	0	1	0	0	0
Dynein, cytoplasmic 1, light intermediate chain 2, isoform CRA_a OS=Homo sapiens GN=DYNC1LI2 PE=4 SV=1	A0A024R6Z0_HUMAN (+1)	54 kDa	0.29	[]	1	2	2	1	0	4
E3 SUMO-protein ligase RanBP2 OS=Homo sapiens GN=RANBP2 PE=1 SV=2	RBP2_HUMAN	358 kDa	0.37	[]	0	0	0	0	0	3
E3 UFM1-protein ligase 1 OS=Homo sapiens GN=UFL1 PE=1 SV=2	UFL1_HUMAN	90 kDa	0.3	[]	1	0	1	1	2	6
E3 ubiquitin-protein ligase DTX3L OS=Homo sapiens GN=DTX3L PE=1 SV=1	DTX3L_HUMAN	84 kDa	0.048	BRAC1 high, Naive low	0	1	1	2	3	5
E3 ubiquitin-protein ligase NEDD4-like OS=Homo sapiens GN=NEDD4L PE=1 SV=2	NED4L_HUMAN	112 kDa	0.15	[]	0	0	0	2	0	5
E3 ubiquitin-protein ligase UBR4 OS=Homo sapiens GN=UBR4 PE=1 SV=1	UBR4_HUMAN	574 kDa	0.37	[]	0	0	0	0	0	6
E3 ubiquitin/ISG15 ligase TRIM25 OS=Homo sapiens GN=TRIM25 PE=1 SV=2	TRI25_HUMAN	71 kDa	0.37	[]	0	0	1	1	0	5
Tripartite motif-containing 25 variant (Fragment) OS=Homo sapiens PE=1 SV=1	Q59GW5_HUMAN	72 kDa	0.67	[]	0	0	2	1	0	4
EFR3A protein (Fragment) OS=Homo sapiens GN=EFR3A PE=2 SV=1	A2VDI4_HUMAN (+1)	76 kDa	0.041	BRAC1 low, Naive high	2	2	3	2	3	2
EH domain-binding protein 1-like protein 1 OS=Homo sapiens GN=EHBP1L1 PE=1 SV=2	EH1L1_HUMAN	162 kDa	0.031	BRAC1 high, Naive low	0	0	0	2	1	1
EH domain-containing protein 1 OS=Homo sapiens GN=EHD1 PE=1 SV=1	A0A024R571_HUMAN (+2)	62 kDa	0.41	[]	3	5	9	4	8	10
EH domain-containing protein 3 OS=Homo sapiens GN=EHD3 PE=1 SV=1	A0A0C4DH83_HUMAN (+1)	62 kDa	0.46	[]	0	0	5	1	0	0
EH domain-containing protein 2 OS=Homo sapiens GN=EHD2 PE=1 SV=2	EHD2_HUMAN (+1)	61 kDa	0.0013	BRAC1 low, Naive high	12	10	15	0	0	0
EH-domain containing 4, isoform CRA_a OS=Homo sapiens GN=EHD4 PE=4 SV=1	A0A024R9N6_HUMAN (+2)	61 kDa	0.022	BRAC1 high, Naive low	2	1	2	7	5	8
EIF2AK2 protein OS=Homo sapiens GN=EIF2AK2 PE=2 SV=1	B7ZKK7_HUMAN (+1)	62 kDa	0.68	[]	0	2	4	2	2	9
EIF4G1 protein OS=Homo sapiens GN=EIF4G1 PE=2 SV=1	B2RU06_HUMAN (+8)	176 kDa	0.31	[]	0	2	2	2	3	11
ELAV-like protein 1 OS=Homo sapiens GN=ELAVL1 PE=1 SV=2	ELAV1_HUMAN	36 kDa	0.87	[]	0	0	1	0	0	2
EMILIN-1 OS=Homo sapiens GN=EMILIN1 PE=1 SV=2	EMIL1_HUMAN (+1)	107 kDa	0.0081	BRAC1 low, Naive high	3	2	3	0	0	0
ER lumen protein-retaining receptor 1 OS=Homo sapiens GN=KDELR1 PE=1 SV=1	ERD21_HUMAN	25 kDa	0.69	[]	0	0	1	0	0	3
ER lumen protein-retaining receptor 2 OS=Homo sapiens GN=KDELR2 PE=1 SV=1	ERD22_HUMAN (+1)	24 kDa	0.37	[]	0	0	0	0	0	4
ER membrane protein complex subunit 1 OS=Homo sapiens GN=EMC1 PE=1 SV=1	EMC1_HUMAN	112 kDa	0.33	[]	1	3	4	1	1	6
ER membrane protein complex subunit 3 OS=Homo sapiens GN=EMC3 PE=1 SV=3	EMC3_HUMAN	30 kDa	0.55	[]	0	0	2	0	0	1
ER membrane protein complex subunit 7 OS=Homo sapiens GN=EMC7 PE=1 SV=1	EMC7_HUMAN (+2)	26 kDa	0.69	[]	0	1	0	0	1	2
ERO1-like protein alpha OS=Homo sapiens GN=ERO1A PE=1 SV=2	ERO1A_HUMAN	54 kDa	0.22	[]	3	3	6	2	4	8
Early endosome antigen 1 OS=Homo sapiens GN=EEA1 PE=1 SV=2	EEA1_HUMAN	162 kDa	0.37	[]	0	0	0	0	0	5
Echinoderm microtubule-associated protein-like 4 OS=Homo sapiens GN=EML4 PE=1 SV=3	B5MBZ0_HUMAN (+1)	110 kDa	< 0.00010	BRAC1 high, Naive low	0	0	0	4	4	5
Electron transfer flavoprotein subunit alpha, mitochondrial OS=Homo sapiens GN=ETFA PE=1 SV=1	ETFA_HUMAN	35 kDa	0.18	[]	5	5	8	12	15	12
Electron transfer flavoprotein subunit beta OS=Homo sapiens GN=ETFB PE=1 SV=3	ETFB_HUMAN	28 kDa	0.029	BRAC1 high, Naive low	0	0	2	3	4	6
EEF1A1 protein OS=Homo sapiens GN=EEF1A1 PE=2 SV=1	Q504Z0_HUMAN	33 kDa	0.29	[]	7	8	10	12	18	20
Elongation factor 1-alpha 1 OS=Homo sapiens GN=EEF1A1 PE=1 SV=1	EF1A1_HUMAN (+8)	50 kDa	0.43	[]	13	13	24	18	28	107
Elongation factor 1-alpha OS=Homo sapiens GN=PTI-1 PE=2 SV=1	Q16577_HUMAN	43 kDa	0.6	[]	6	6	7	8	10	13

tr|Q96EB3|Q96EB3_HUMAN EEF1A1 protein (Fragment) OS=Homo sapiens GN=EEF1A1 PE=1 SV=1	Q96EB3_HUMAN	?	0.37	[]	0	0	0	0	1	0
Elongation factor 1-delta OS=Homo sapiens GN=EEF1D PE=1 SV=1	A0A087X1X7_HUMAN (+7)	69 kDa	0.07	[]	3	3	5	5	3	4
Elongation factor 1-gamma OS=Homo sapiens GN=EEF1G PE=1 SV=3	EF1G_HUMAN (+1)	50 kDa	0.81	[]	5	5	12	10	11	13
Elongation factor 2 OS=Homo sapiens GN=EEF2 PE=1 SV=4	EF2_HUMAN	95 kDa	0.78	[]	13	19	23	17	15	57
Elongation factor Tu, mitochondrial OS=Homo sapiens GN=TUFM PE=1 SV=2	EFTU_HUMAN	50 kDa	0.037	BRAC1 low, Naive high	5	7	11	7	8	12
Elongation of very long chain fatty acids protein 1 OS=Homo sapiens GN=ELOVL1 PE=1 SV=1	ELOV1_HUMAN	33 kDa	0.16	[]	0	0	0	1	2	0
Emerin OS=Homo sapiens GN=EMD PE=1 SV=1	EMD_HUMAN	29 kDa	0.12	[]	2	2	4	1	1	5
Endoglin OS=Homo sapiens GN=ENG PE=1 SV=2	EGLN_HUMAN (+3)	71 kDa	0.017	BRAC1 low, Naive high	8	12	6	0	0	0
tr|Q96CG0|Q96CG0_HUMAN ENG protein OS=Homo sapiens GN=ENG PE=2 SV=1	Q96CG0_HUMAN	?	0.014	BRAC1 low, Naive high	7	11	6	0	0	0
Endonuclease domain-containing 1 protein OS=Homo sapiens GN=ENDOD1 PE=1 SV=2	ENDD1_HUMAN	55 kDa	0.49	[]	1	2	1	2	2	1
Endophilin-B1 OS=Homo sapiens GN=SH3GLB1 PE=1 SV=1	A0A087WW40_HUMAN (+1)	44 kDa	0.03	BRAC1 high, Naive low	0	0	0	1	1	3
Endoplasmic reticulum aminopeptidase 2 OS=Homo sapiens GN=ERAP2 PE=1 SV=2	ERAP2_HUMAN (+1)	110 kDa	0.17	[]	0	0	0	0	1	3
Endoplasmic reticulum lectin 1 OS=Homo sapiens GN=ERLEC1 PE=1 SV=1	ERLEC_HUMAN (+1)	55 kDa	0.98	[]	0	0	2	0	0	3
Endoplasmic reticulum metallopeptidase 1 OS=Homo sapiens GN=ERMP1 PE=1 SV=2	ERMP1_HUMAN	100 kDa	0.013	BRAC1 high, Naive low	0	0	0	8	8	20
Endoplasmic reticulum resident protein 29 OS=Homo sapiens GN=ERP29 PE=1 SV=4	ERP29_HUMAN (+1)	29 kDa	0.99	[]	0	0	4	1	3	1
Endoplasmic reticulum resident protein 44 OS=Homo sapiens GN=ERP44 PE=1 SV=1	ERP44_HUMAN	47 kDa	0.2	[]	4	2	3	1	2	5
Endoplasmin OS=Homo sapiens GN=HSP90B1 PE=1 SV=1	ENPL_HUMAN (+2)	92 kDa	0.35	[]	25	32	23	25	29	58
Endosialin OS=Homo sapiens GN=CD248 PE=1 SV=1	CD248_HUMAN	81 kDa	0.14	[]	1	2	0	0	0	0
Endothelial cell-selective adhesion molecule OS=Homo sapiens GN=ESAM PE=1 SV=1	ESAM_HUMAN	41 kDa	0.13	[]	0	0	0	0	1	2
Endothelial protein C receptor OS=Homo sapiens GN=PROCR PE=1 SV=1	EPCR_HUMAN	27 kDa	0.68	[]	0	0	2	1	3	0
Endothelin-converting enzyme 1 OS=Homo sapiens GN=ECE1 PE=1 SV=2	ECE1_HUMAN (+3)	87 kDa	0.75	[]	0	3	5	0	3	7
Enhancer of mRNA-decapping protein 4 OS=Homo sapiens GN=EDC4 PE=1 SV=1	EDC4_HUMAN	152 kDa	0.37	[]	0	0	0	0	0	3
Enolase 1, (Alpha), isoform CRA_a OS=Homo sapiens GN=ENO1 PE=2 SV=1	A0A024R4F1_HUMAN (+1)	47 kDa	0.11	[]	9	9	11	31	31	21
Enoyl-CoA delta isomerase 1, mitochondrial OS=Homo sapiens GN=ECI1 PE=1 SV=1	ECI1_HUMAN	33 kDa	0.89	[]	0	0	2	1	1	0
Enoyl-CoA delta isomerase 2, mitochondrial OS=Homo sapiens GN=ECI2 PE=1 SV=4	ECI2_HUMAN (+11)	44 kDa	0.00091	BRAC1 low, Naive high	1	1	2	0	0	0
Enoyl-CoA hydratase, mitochondrial OS=Homo sapiens GN=ECHS1 PE=1 SV=4	ECHM_HUMAN	31 kDa	0.039	BRAC1 high, Naive low	1	2	3	6	8	6
Ephrin type-B receptor 4 OS=Homo sapiens GN=EPHB4 PE=1 SV=2	EPHB4_HUMAN (+3)	108 kDa	0.051	[]	0	0	0	9	5	3
Receptor protein-tyrosine kinase OS=Homo sapiens GN=EPHB3 PE=4 SV=1	D3DNT9_HUMAN (+1)	106 kDa	0.13	[]	0	0	0	4	3	0
Ephrin-B1 OS=Homo sapiens GN=EFNB1 PE=1 SV=1	EFNB1_HUMAN	38 kDa	0.064	[]	0	0	0	10	10	2
Epidermal growth factor receptor kinase substrate 8 OS=Homo sapiens GN=EPS8 PE=1 SV=1	EPS8_HUMAN	92 kDa	< 0.00010	BRAC1 high, Naive low	0	0	0	6	6	9
Epidermal growth factor receptor kinase substrate 8-like protein 1 OS=Homo sapiens GN=EPS8L1 PE=1 SV=3	ES8L1_HUMAN	80 kDa	0.11	[]	0	0	0	2	2	10
Epidermal growth factor receptor kinase substrate 8-like protein 2 OS=Homo sapiens GN=EPS8L2 PE=1 SV=2	ES8L2_HUMAN	81 kDa	0.073	[]	0	0	0	2	3	10
Epidermal growth factor receptor substrate 15 OS=Homo sapiens GN=EPS15 PE=1 SV=2	EPS15_HUMAN	99 kDa	0.44	[]	0	0	1	2	1	0
Epididymis luminal protein 113 OS=Homo sapiens GN=HEL113 PE=2 SV=1	V9HWE1_HUMAN (+1)	54 kDa	0.0061	BRAC1 low, Naive high	52	86	59	0	2	0
cDNA FLJ34494 fis, clone HLUNG2005030, highly similar to VIMENTIN OS=Homo sapiens PE=2 SV=1	B3KRK8_HUMAN	47 kDa	0.0088	BRAC1 low, Naive high	39	75	50	0	0	0
Epididymis secretory protein Li 96 (Fragment) OS=Homo sapiens GN=HEL-S-96 PE=2 SV=1	A0A0K0K1J6_HUMAN (+3)	16 kDa	0.0068	BRAC1 high, Naive low	0	0	0	2	3	2
Epididymis secretory protein Li 276 OS=Homo sapiens GN=HEL-S-276 PE=2 SV=1	A0A0K0K1K4_HUMAN (+1)	28 kDa	0.88	[]	1	0	4	2	1	4
Epididymis secretory sperm binding protein Li 129m OS=Homo sapiens GN=HEL-S-129m PE=2 SV=1	A0A0K0K1L8_HUMAN (+1)	29 kDa	0.7	[]	0	2	5	1	5	8
Epididymis secretory sperm binding protein Li 284 OS=Homo sapiens GN=HEL-S-284 PE=2 SV=1	A0A0K0K1H7_HUMAN (+4)	86 kDa	0.0085	BRAC1 low, Naive high	9	7	10	3	4	2
Epididymis tissue sperm binding protein Li 3a OS=Homo sapiens PE=1 SV=1	E9KL35_HUMAN (+1)	35 kDa	0.12	[]	5	5	6	7	7	7
Epididymis tissue sperm binding protein Li 14m OS=Homo sapiens PE=2 SV=1	E9KL44_HUMAN (+1)	83 kDa	0.013	BRAC1 low, Naive high	5	8	10	4	6	10
Epiplakin OS=Homo sapiens GN=EPPK1 PE=1 SV=2	A0A075B730_HUMAN (+1)	553 kDa	0.2	[]	0	0	0	4	3	30
Epiplakin OS=Homo sapiens GN=EPPK1 PE=1 SV=2	EPIPL_HUMAN	556 kDa	0.19	[]	0	0	0	4	3	29
Epithelial cell adhesion molecule OS=Homo sapiens GN=EPCAM PE=1 SV=2	EPCAM_HUMAN	35 kDa	0.019	BRAC1 high, Naive low	0	0	1	30	36	16
Epoxide hydrolase 1 OS=Homo sapiens GN=EPHX1 PE=1 SV=1	HYEP_HUMAN (+1)	53 kDa	0.53	[]	1	0	3	2	1	9
Equilibrative nucleoside transporter 1 OS=Homo sapiens GN=SLC29A1 PE=1 SV=3	S29A1_HUMAN	50 kDa	0.17	[]	0	0	0	1	5	1
Erythrocyte band 7 integral membrane protein OS=Homo sapiens GN=STOM PE=1 SV=3	STOM_HUMAN (+1)	32 kDa	0.0097	BRAC1 low, Naive high	8	8	6	0	0	0
Ethanolamine-phosphate cytidylyltransferase OS=Homo sapiens GN=PCYT2 PE=1 SV=1	PCY2_HUMAN (+1)	44 kDa	0.37	[]	0	0	0	0	0	3
Ethanolaminephosphotransferase 1 OS=Homo sapiens GN=EPT1 PE=1 SV=1	A0A087WW58_HUMAN (+5)	45 kDa	0.028	BRAC1 high, Naive low	0	0	0	1	2	1
Ethylmalonic encephalopathy 1, isoform CRA_a OS=Homo sapiens GN=ETHE1 PE=2 SV=1	B2RCZ7_HUMAN (+2)	25 kDa	0.19	[]	1	2	2	2	2	0
Eukaryotic translation elongation factor 1 beta 2, isoform CRA_a OS=Homo sapiens GN=EEF1B2 PE=3 SV=1	A0A024R3W7_HUMAN (+1)	25 kDa	0.68	[]	2	0	2	2	2	1
Eukaryotic translation initiation factor 2 subunit 1 OS=Homo sapiens GN=EIF2S1 PE=1 SV=3	IF2A_HUMAN (+1)	36 kDa	0.22	[]	1	1	1	1	1	2
Eukaryotic translation initiation factor 2 subunit 2 OS=Homo sapiens GN=EIF2S2 PE=1 SV=2	IF2B_HUMAN (+2)	38 kDa	0.65	[]	0	0	3	0	1	1
Eukaryotic translation initiation factor 2, subunit 3 gamma, 52kDa, isoform CRA_a OS=Homo sapiens GN=EIF2S3 PE=4 SV=1	A0A024RBY4_HUMAN (+3)	51 kDa	0.61	[]	2	3	1	4	4	5
Eukaryotic translation initiation factor 2A OS=Homo sapiens GN=EIF2A PE=1 SV=3	EIF2A_HUMAN (+1)	65 kDa	0.11	[]	0	0	0	1	1	5
Eukaryotic translation initiation factor 3 subunit A OS=Homo sapiens GN=EIF3A PE=1 SV=1	EIF3A_HUMAN (+2)	167 kDa	0.35	[]	2	1	2	3	3	7
Eukaryotic translation initiation factor 3 subunit B OS=Homo sapiens GN=EIF3B PE=3 SV=1	A0A024R821_HUMAN (+3)	92 kDa	0.4	[]	1	1	1	1	2	10
Eukaryotic translation initiation factor 3 subunit C OS=Homo sapiens GN=EIF3C PE=1 SV=1	EIF3C_HUMAN (+6)	105 kDa	0.94	[]	1	1	1	0	2	4
Eukaryotic translation initiation factor 3 subunit E OS=Homo sapiens GN=EIF3E PE=1 SV=1	EIF3E_HUMAN (+2)	52 kDa	0.55	[]	0	0	2	0	0	1
Eukaryotic translation initiation factor 3 subunit F OS=Homo sapiens GN=EIF3F PE=2 SV=1	B3KSH1_HUMAN (+2)	39 kDa	0.71	[]	2	1	2	2	2	4
Eukaryotic translation initiation factor 3 subunit L OS=Homo sapiens GN=EIF3L PE=1 SV=1	B0QY89_HUMAN (+2)	71 kDa	0.59	[]	2	2	2	1	2	6
Eukaryotic translation initiation factor 3 subunit M OS=Homo sapiens GN=EIF3M PE=1 SV=1	EIF3M_HUMAN	43 kDa	0.86	[]	1	1	1	1	0	6

Eukaryotic translation initiation factor 4B OS=Homo sapiens GN=EIF4B PE=1 SV=2	IF4B_HUMAN (+3)	69 kDa	0.87	[]	0	0	1	0	0	2
Eukaryotic translation initiation factor 4E OS=Homo sapiens GN=EIF4E PE=1 SV=2	IF4E_HUMAN	25 kDa	0.37	[]	0	0	0	0	0	4
Eukaryotic translation initiation factor 4H OS=Homo sapiens GN=EIF4H PE=1 SV=5	IF4H_HUMAN	27 kDa	0.13	[]	0	0	0	0	1	2
Eukaryotic translation initiation factor 5A (Fragment) OS=Homo sapiens GN=EIF5A PE=1 SV=6	I3L397_HUMAN (+2)	16 kDa	0.59	[]	0	0	3	1	1	6
Eukaryotic translation initiation factor 5B OS=Homo sapiens GN=EIF5B PE=1 SV=1	A0A087WUT6_HUMAN (+4)	139 kDa	0.36	[]	0	0	1	1	0	10
Eukaryotic translation initiation factor 6 OS=Homo sapiens GN=EIF6 PE=1 SV=1	IF6_HUMAN	27 kDa	0.00025	BRAC1 high, Naive low	0	0	0	3	4	5
Exocyst complex component 1, isoform CRA_a OS=Homo sapiens GN=EXOC1 PE=4 SV=1	A0A024RDA1_HUMAN (+2)	100 kDa	0.85	[]	0	1	1	1	0	3
Exocyst complex component 2, isoform CRA_a OS=Homo sapiens GN=EXOC2 PE=4 SV=1	A0A024QZT2_HUMAN (+2)	104 kDa	0.5	[]	0	0	1	1	0	2
Exocyst complex component 3 OS=Homo sapiens GN=EXOC3 PE=1 SV=2	EXOC3_HUMAN (+2)	87 kDa	0.69	[]	0	0	1	0	0	3
Exocyst complex component 4 OS=Homo sapiens GN=EXOC4 PE=1 SV=1	EXOC4_HUMAN (+1)	111 kDa	0.3	[]	2	1	3	3	5	6
Exocyst complex component 7 OS=Homo sapiens GN=EXOC7 PE=1 SV=3	EXOC7_HUMAN (+4)	83 kDa	0.37	[]	0	0	0	0	0	2
Exocyst complex component 8 OS=Homo sapiens GN=EXOC8 PE=1 SV=2	EXOC8_HUMAN	82 kDa	0.12	[]	0	0	0	0	2	2
Exportin-1 OS=Homo sapiens GN=XPO1 PE=1 SV=1	XPO1_HUMAN	123 kDa	0.37	[]	0	0	0	0	0	10
Exportin-2 OS=Homo sapiens GN=CSE1L PE=1 SV=3	XPO2_HUMAN	110 kDa	0.098	[]	0	0	1	5	5	22
Exportin-5 OS=Homo sapiens GN=XPO5 PE=1 SV=1	XPO5_HUMAN (+1)	136 kDa	0.37	[]	0	0	0	0	0	2
Exportin-T OS=Homo sapiens GN=XPOT PE=1 SV=2	XPOT_HUMAN (+2)	110 kDa	0.37	[]	0	0	0	0	0	2
Extended synaptotagmin-2 (Fragment) OS=Homo sapiens GN=ESYT2 PE=1 SV=1	H7BXI1_HUMAN	98 kDa	0.36	[]	3	1	4	5	5	9
FACT complex subunit SSRP1 OS=Homo sapiens GN=SSRP1 PE=1 SV=1	SSRP1_HUMAN (+1)	81 kDa	0.37	[]	0	0	0	0	0	3
FAD synthase OS=Homo sapiens GN=FLAD1 PE=1 SV=1	FAD1_HUMAN (+2)	65 kDa	0.37	[]	0	0	0	0	0	3
FARSB protein (Fragment) OS=Homo sapiens GN=FARSB PE=2 SV=2	Q9BR63_HUMAN (+1)	66 kDa	0.64	[]	1	0	1	1	1	3
FERM, RhoGEF and pleckstrin domain-containing protein 1 OS=Homo sapiens GN=FARP1 PE=1 SV=1	FARP1_HUMAN	119 kDa	0.095	[]	4	3	5	4	4	6
FERM, RhoGEF and pleckstrin domain-containing protein 2 OS=Homo sapiens GN=FARP2 PE=1 SV=3	FARP2_HUMAN	120 kDa	0.12	[]	0	0	0	4	5	0
Family with sequence similarity 49, member B, isoform CRA_a OS=Homo sapiens GN=FAM49B PE=4 SV=1	A0A024R9G4_HUMAN (+2)	37 kDa	0.0092	BRAC1 high, Naive low	0	0	1	3	4	7
Family with sequence similarity 62 (C2 domain containing), member A, isoform CRA_a OS=Homo sapiens GN=FAM62A PE=4 SV=1	A0A024RB16_HUMAN (+3)	124 kDa	0.0032	BRAC1 low, Naive high	11	13	20	11	10	19
Far upstream element-binding protein 1 OS=Homo sapiens GN=FUBP1 PE=1 SV=3	FUBP1_HUMAN (+4)	68 kDa	0.37	[]	0	0	0	0	0	2
Fascin OS=Homo sapiens GN=FSCN1 PE=1 SV=3	FSCN1_HUMAN (+1)	55 kDa	0.0017	BRAC1 low, Naive high	3	6	7	0	0	0
Fatty acid synthase OS=Homo sapiens GN=FASN PE=1 SV=3	FAS_HUMAN	273 kDa	0.029	BRAC1 high, Naive low	0	0	2	15	19	51
Fatty aldehyde dehydrogenase OS=Homo sapiens GN=ALDH3A2 PE=1 SV=1	AL3A2_HUMAN (+3)	55 kDa	0.018	BRAC1 high, Naive low	0	0	0	2	2	1
Fermitin family homolog 2 OS=Homo sapiens GN=FERMT2 PE=1 SV=1	FERM2_HUMAN (+3)	78 kDa	0.12	[]	1	1	6	0	0	0
Fibulin-2 OS=Homo sapiens GN=FBLN2 PE=1 SV=2	FBLN2_HUMAN (+2)	127 kDa	0.025	BRAC1 low, Naive high	1	3	2	0	0	0
Filamin-A OS=Homo sapiens GN=FLNA PE=1 SV=4	FLNA_HUMAN	281 kDa	0.00083	BRAC1 low, Naive high	102	117	134	55	40	26
Filamin-B OS=Homo sapiens GN=FLNB PE=1 SV=2	FLNB_HUMAN	278 kDa	0.05	[]	13	12	8	103	86	48
Filamin-C OS=Homo sapiens GN=FLNC PE=1 SV=3	FLNC_HUMAN	291 kDa	< 0.00010	BRAC1 low, Naive high	43	48	63	5	3	1
Flavin reductase (NADPH) OS=Homo sapiens GN=BLVRB PE=1 SV=3	BLVRB_HUMAN (+1)	22 kDa	0.5	[]	0	0	1	1	0	2
Flotillin-1 OS=Homo sapiens GN=FLOT1 PE=1 SV=3	FLOT1_HUMAN (+2)	47 kDa	0.023	BRAC1 high, Naive low	4	4	8	13	13	14
Focal adhesion kinase 1 OS=Homo sapiens GN=PTK2 PE=1 SV=2	FAK1_HUMAN (+1)	119 kDa	0.37	[]	0	0	0	0	0	2
Fragile X mental retardation syndrome-related protein 1 OS=Homo sapiens GN=FXR1 PE=1 SV=3	FXR1_HUMAN (+9)	70 kDa	0.77	[]	0	0	2	0	0	5
Fructose-1,6-bisphosphatase 1 OS=Homo sapiens GN=FBP1 PE=1 SV=5	F16P1_HUMAN (+1)	37 kDa	0.13	[]	0	0	0	3	2	0
Fructose-bisphosphate aldolase A OS=Homo sapiens GN=ALDOA PE=1 SV=2	ALDOA_HUMAN (+1)	39 kDa	0.51	[]	20	23	31	40	33	40
Fructose-bisphosphate aldolase OS=Homo sapiens GN=ALDOC PE=3 SV=1	A0A024QZ64_HUMAN (+4)	39 kDa	0.011	BRAC1 high, Naive low	5	7	10	12	14	17
Fumarate hydratase, mitochondrial OS=Homo sapiens GN=FH PE=1 SV=3	FUMH_HUMAN	55 kDa	0.03	BRAC1 low, Naive high	1	3	3	1	0	1
GDH/6PGL endoplasmic bifunctional protein OS=Homo sapiens GN=H6PD PE=1 SV=2	G6PE_HUMAN (+1)	89 kDa	0.46	[]	2	1	0	1	0	2
GDP-L-fucose synthase OS=Homo sapiens GN=TSTA3 PE=1 SV=1	FCL_HUMAN (+1)	36 kDa	0.37	[]	0	0	0	0	0	2
GDP-fucose protein O-fucosyltransferase 2 OS=Homo sapiens GN=POFUT2 PE=1 SV=3	OFUT2_HUMAN (+1)	50 kDa	0.78	[]	0	0	2	0	0	2
GIPC PDZ domain containing family, member 1, isoform CRA_a OS=Homo sapiens GN=GIPC1 PE=4 SV=1	A0A024R7I0_HUMAN (+2)	36 kDa	0.15	[]	0	1	0	2	2	9
GRAM domain-containing protein 3 OS=Homo sapiens GN=GRAMD3 PE=1 SV=1	GRAM3_HUMAN (+2)	48 kDa	0.37	[]	0	4	0	0	0	0
GTP-binding nuclear protein Ran OS=Homo sapiens GN=RAN PE=1 SV=1	B5MDF5_HUMAN (+1)	26 kDa	0.29	[]	0	0	2	2	1	4
GTP-binding protein 1 OS=Homo sapiens GN=GTPBP1 PE=1 SV=3	GTPB1_HUMAN (+1)	72 kDa	0.37	[]	0	0	0	0	0	3
GTP-binding protein SAR1b OS=Homo sapiens GN=SAR1B PE=1 SV=1	SAR1B_HUMAN (+2)	22 kDa	0.58	[]	0	1	1	0	0	2
GTP:AMP phosphotransferase AK3, mitochondrial OS=Homo sapiens GN=AK3 PE=1 SV=4	KAD3_HUMAN (+3)	26 kDa	0.14	[]	3	2	1	0	2	0
Galectin OS=Homo sapiens GN=hCG_22119 PE=4 SV=1	A0A024R693_HUMAN (+3)	26 kDa	0.53	[]	2	2	2	7	6	1
Galectin-1 OS=Homo sapiens GN=LGALS1 PE=1 SV=2	LEG1_HUMAN	15 kDa	0.0096	BRAC1 low, Naive high	3	6	4	0	0	0
Galectin-4 OS=Homo sapiens GN=LGALS4 PE=1 SV=1	LEG4_HUMAN (+1)	36 kDa	0.057	[]	0	0	2	104	113	26
Gamma-adducin OS=Homo sapiens GN=ADD3 PE=1 SV=1	ADDG_HUMAN (+2)	79 kDa	0.0014	BRAC1 high, Naive low	0	0	0	3	2	3
Gamma-glutamyltransferase 5 OS=Homo sapiens GN=GGT5 PE=1 SV=2	GGT5_HUMAN (+2)	62 kDa	0.13	[]	0	1	2	0	0	0
Gamma-interferon-inducible protein 16 OS=Homo sapiens GN=IFI16 PE=1 SV=3	IF16_HUMAN (+3)	88 kDa	0.37	[]	0	0	2	0	0	0
Gamma-synuclein OS=Homo sapiens GN=SNCG PE=1 SV=1	Q6FHG5_HUMAN (+1)	13 kDa	0.028	BRAC1 high, Naive low	0	0	0	1	2	1
Gamma-tubulin complex component 3 OS=Homo sapiens GN=TUBGCP3 PE=1 SV=2	GCP3_HUMAN	104 kDa	0.37	[]	0	0	0	0	0	4
Gap junction alpha-1 protein OS=Homo sapiens GN=GJA1 PE=1 SV=2	CXA1_HUMAN (+5)	43 kDa	0.0021	BRAC1 low, Naive high	1	2	2	0	0	0
Gasdermin-D OS=Homo sapiens GN=GSDMD PE=1 SV=1	GSDMD_HUMAN (+2)	53 kDa	0.37	[]	0	0	0	0	0	2
Gem-associated protein 5 OS=Homo sapiens GN=GEMIN5 PE=1 SV=3	GEMI5_HUMAN (+1)	169 kDa	0.37	[]	0	0	0	0	0	2
Glia-derived nexin OS=Homo sapiens GN=SERPINE2 PE=1 SV=1	GDN_HUMAN (+3)	44 kDa	0.14	[]	1	2	0	0	0	0

Glucosamine (N-acetyl)-6-sulfatase (Sanfilippo disease IIID), isoform CRA_a OS=Homo sapiens GN=GNS PE=4 SV=1	A0A024RBC5_HUMAN (+7)	62 kDa	0.5	[]	0	0	1	1	0	2
Glucosaminyl (N-acetyl) transferase 3, mucin type, isoform CRA_a OS=Homo sapiens GN=GCNT3 PE=4 SV=1	A0A024R5T9_HUMAN (+2)	51 kDa	0.038	BRAC1 high, Naive low	0	0	0	1	4	5
Glucose-6-phosphate 1-dehydrogenase OS=Homo sapiens PE=2 SV=1	A8K8D9_HUMAN (+3)	59 kDa	0.61	[]	4	3	5	6	6	12
Glucose-6-phosphate isomerase (Fragment) OS=Homo sapiens GN=GPI PE=1 SV=1	A0A0A0MTS2_HUMAN (+2)	65 kDa	0.0043	BRAC1 high, Naive low	1	1	5	15	16	28
Glucosylceramidase OS=Homo sapiens GN=GBA PE=1 SV=3	GLCM_HUMAN (+5)	60 kDa	0.14	[]	1	7	4	0	0	3
GLUD1 protein (Fragment) OS=Homo sapiens GN=GLUD1 PE=2 SV=1	Q14400_HUMAN	29 kDa	0.73	[]	1	0	4	2	3	0
Glutamate dehydrogenase OS=Homo sapiens PE=2 SV=1	B4DMF5_HUMAN (+2)	57 kDa	0.087	[]	5	6	6	5	8	6
Glutamate-rich WD repeat-containing protein 1 OS=Homo sapiens GN=GRWD1 PE=1 SV=1	GRWD1_HUMAN (+4)	49 kDa	0.37	[]	0	0	0	0	0	2
Glutaminase kidney isoform, mitochondrial OS=Homo sapiens GN=GLS PE=1 SV=1	GLSK_HUMAN (+1)	73 kDa	< 0.00010	BRAC1 low, Naive high	4	6	7	0	0	0
tr|B3KM58|B3KM58_HUMAN cDNA FLJ10358 fis, clone NT2RM2001238, highly similar to Glutaminase...	B3KM58_HUMAN	?	0.034	BRAC1 low, Naive high	1	2	5	0	0	0
Glutamine--fructose-6-phosphate aminotransferase [isomerizing] 1 OS=Homo sapiens GN=GFPT1 PE=1 SV=3	GFPT1_HUMAN	79 kDa	0.34	[]	2	2	3	5	3	24
Glutamine--tRNA ligase OS=Homo sapiens GN=QARS PE=1 SV=1	SYQ_HUMAN	88 kDa	0.5	[]	0	1	3	1	1	14
Glutaminyl-peptide cyclotransferase-like protein OS=Homo sapiens GN=QPCTL PE=1 SV=1	K7EQG1_HUMAN (+1)	14 kDa	0.13	[]	0	0	0	1	0	2
Glutaredoxin-3 OS=Homo sapiens GN=GLRX3 PE=1 SV=2	GLRX3_HUMAN	37 kDa	0.37	[]	0	0	0	0	0	2
Glutathione S-transferase P OS=Homo sapiens GN=GSTP1 PE=1 SV=2	GSTP1_HUMAN (+2)	23 kDa	0.073	[]	1	0	3	14	11	5
Glutathione S-transferase pi (Fragment) OS=Homo sapiens GN=GSTP1 PE=2 SV=1	C7DJS2_HUMAN	17 kDa	0.019	BRAC1 high, Naive low	0	0	0	5	8	4
Glutathione S-transferase kappa 1 OS=Homo sapiens GN=GSTK1 PE=1 SV=1	E9PFN5_HUMAN (+2)	22 kDa	0.82	[]	1	1	1	2	1	2
Glyceraldehyde-3-phosphate dehydrogenase OS=Homo sapiens GN=GAPDH PE=1 SV=3	G3P_HUMAN (+1)	36 kDa	0.11	[]	26	34	57	53	40	38
Glycerol-3-phosphate dehydrogenase OS=Homo sapiens PE=2 SV=1	A8K1Z2_HUMAN (+2)	81 kDa	0.52	[]	0	3	3	1	1	4
Glycogen [starch] synthase, muscle OS=Homo sapiens GN=GYS1 PE=1 SV=2	GYS1_HUMAN (+2)	84 kDa	0.37	[]	0	0	0	0	0	6
Glycogen phosphorylase, brain form OS=Homo sapiens GN=PYGB PE=1 SV=5	PYGB_HUMAN	97 kDa	0.14	[]	0	0	4	9	8	42
Glycyl-tRNA synthetase OS=Homo sapiens GN=GARS PE=3 SV=1	A0A090N8G0_HUMAN (+1)	78 kDa	0.75	[]	1	2	6	3	4	8
Glycylpeptide N-tetradecanoyltransferase 1 OS=Homo sapiens GN=NMT1 PE=1 SV=2	NMT1_HUMAN (+1)	57 kDa	0.37	[]	0	0	0	0	0	2
Glypican-1 OS=Homo sapiens GN=GPC1 PE=1 SV=2	GPC1_HUMAN (+3)	62 kDa	0.37	[]	0	2	0	0	0	0
Golgi apparatus protein 1 OS=Homo sapiens GN=GLG1 PE=1 SV=2	GSLG1_HUMAN	135 kDa	0.015	BRAC1 high, Naive low	1	1	1	4	5	4
Golgi integral membrane protein 4 OS=Homo sapiens GN=GOLIM4 PE=1 SV=1	GOLI4_HUMAN (+1)	82 kDa	0.37	[]	0	0	3	0	0	0
Golgi phosphoprotein 3 OS=Homo sapiens GN=GOLPH3 PE=1 SV=1	GOLP3_HUMAN (+4)	34 kDa	0.37	[]	0	0	0	0	0	4
Golgi resident protein GCP60 OS=Homo sapiens GN=ACBD3 PE=1 SV=4	GCP60_HUMAN (+1)	61 kDa	0.82	[]	0	1	0	0	0	3
Golgi-associated plant pathogenesis-related protein 1 OS=Homo sapiens GN=GLIPR2 PE=1 SV=3	GAPR1_HUMAN (+2)	17 kDa	0.061	[]	2	1	1	0	0	0
Golgi-specific brefeldin A-resistance guanine nucleotide exchange factor 1 OS=Homo sapiens GN=GBF1 PE=1 SV=2	GBF1_HUMAN	206 kDa	0.37	[]	0	0	0	0	0	2
Golgin subfamily A member 2 OS=Homo sapiens GN=GOLGA2 PE=1 SV=3	GOGA2_HUMAN (+1)	113 kDa	0.38	[]	1	3	0	0	1	2
Golgin subfamily B member 1 OS=Homo sapiens GN=GOLGB1 PE=1 SV=2	GOGB1_HUMAN	376 kDa	0.14	[]	1	2	0	0	0	0
Gremlin-1 OS=Homo sapiens GN=GREM1 PE=1 SV=1	GREM1_HUMAN (+2)	21 kDa	0.017	BRAC1 low, Naive high	1	1	3	0	0	0
Growth factor receptor-bound protein 2 OS=Homo sapiens GN=GRB2 PE=2 SV=1	B0LPF3_HUMAN (+3)	25 kDa	0.031	BRAC1 high, Naive low	0	0	0	2	1	1
Growth factor receptor-bound protein 7 OS=Homo sapiens GN=GRB7 PE=1 SV=2	GRB7_HUMAN	60 kDa	0.0027	BRAC1 high, Naive low	0	0	0	1	1	2
Guanine deaminase, isoform CRA_b OS=Homo sapiens GN=GDA PE=4 SV=1	A0A024R231_HUMAN (+1)	51 kDa	0.0027	BRAC1 high, Naive low	0	0	0	1	1	2
Guanine nucleotide binding protein (G protein), alpha 13, isoform CRA_a OS=Homo sapiens GN=GNA13 PE=4 SV=1	A0A024R8M0_HUMAN (+1)	44 kDa	0.42	[]	1	2	3	5	4	3
Guanine nucleotide binding protein (G protein), beta polypeptide 1, isoform CRA_a OS=Homo sapiens GN=GNB1 PE=4 SV=1	A0A024R056_HUMAN (+2)	37 kDa	0.6	[]	7	9	10	24	17	10
Guanine nucleotide-binding protein G(I)/G(S)/G(T) subunit beta-2 OS=Homo sapiens GN=GNB2 PE=1 SV=3	GBB2_HUMAN (+1)	37 kDa	0.66	[]	6	8	11	19	14	12
Guanine nucleotide-binding protein subunit beta-4 OS=Homo sapiens GN=GNB4 PE=1 SV=3	GBB4_HUMAN	38 kDa	0.75	[]	3	3	5	5	7	5
Guanine nucleotide binding protein (G protein), q polypeptide, isoform CRA_c OS=Homo sapiens GN=GNAQ PE=4 SV=1	A0A024R240_HUMAN (+2)	42 kDa	0.37	[]	1	3	6	4	2	4
Guanine nucleotide-binding protein G(I)/G(S)/G(O) subunit gamma-12 OS=Homo sapiens GN=GNG12 PE=1 SV=3	GBG12_HUMAN	8 kDa	0.88	[]	4	2	2	5	6	2
Guanine nucleotide-binding protein G(i) subunit alpha-1 OS=Homo sapiens GN=GNAI1 PE=1 SV=2	GNAI1_HUMAN	40 kDa	0.23	[]	2	2	10	10	11	48
Guanine nucleotide-binding protein G(i) subunit alpha-2 OS=Homo sapiens GN=GNAI2 PE=1 SV=3	GNAI2_HUMAN	40 kDa	0.18	[]	12	15	22	27	32	67
Guanine nucleotide-binding protein G(k) subunit alpha OS=Homo sapiens GN=GNAI3 PE=1 SV=3	GNAI3_HUMAN	41 kDa	0.087	[]	1	2	9	15	19	55
Guanine nucleotide-binding protein G(s) subunit alpha isoforms XLas OS=Homo sapiens GN=GNAS PE=1 SV=2	GNAS1_HUMAN (+1)	111 kDa	0.038	BRAC1 high, Naive low	1	1	5	28	21	13
Guanine nucleotide-binding protein subunit alpha-11 OS=Homo sapiens GN=GNA11 PE=1 SV=2	GNA11_HUMAN (+1)	42 kDa	0.68	[]	1	5	10	6	8	5
Guanine nucleotide-binding protein-like 1 OS=Homo sapiens GN=GNL1 PE=1 SV=2	GNL1_HUMAN (+9)	69 kDa	0.37	[]	0	0	0	0	0	2
H.sapiens ras-related Hrab1A protein OS=Homo sapiens GN=RAB1A PE=2 SV=1	Q5U0I6_HUMAN (+1)	23 kDa	0.065	[]	2	1	2	6	7	5
H.sapiens ras-related Hrab3B protein OS=Homo sapiens PE=2 SV=1	A0A0A1HAV6_HUMAN (+1)	25 kDa	0.37	[]	0	0	3	0	0	0
H/ACA ribonucleoprotein complex subunit 4 OS=Homo sapiens GN=DKC1 PE=1 SV=3	DKC1_HUMAN (+3)	58 kDa	0.37	[]	0	0	0	0	0	3
HCG24487, isoform CRA_c OS=Homo sapiens GN=hCG_24487 PE=3 SV=1	A0A024R261_HUMAN (+5)	21 kDa	0.55	[]	0	1	3	1	3	5
HCG26477 OS=Homo sapiens GN=RPS28 PE=2 SV=1	B2R4R9_HUMAN (+1)	8 kDa	0.65	[]	2	0	0	1	1	0
HCG27198, isoform CRA_c OS=Homo sapiens GN=hCG_27198 PE=4 SV=1	A0A024QZ63_HUMAN (+2)	231 kDa	0.016	BRAC1 high, Naive low	0	0	0	12	6	8
cDNA FLJ59649, highly similar to Myosin-18A OS=Homo sapiens PE=2 SV=1	B4E0T8_HUMAN	134 kDa	0.014	BRAC1 high, Naive low	0	0	0	10	5	7
HCG30204, isoform CRA_a OS=Homo sapiens GN=hCG_30204 PE=4 SV=1	A0A024R0Y2_HUMAN (+2)	257 kDa	0.14	[]	0	0	0	3	1	11
HCG1640809, isoform CRA_b OS=Homo sapiens GN=hCG_1640809 PE=4 SV=1	A0A024R4N0_HUMAN (+2)	35 kDa	0.15	[]	2	2	6	3	3	1
HCG1821276, isoform CRA_a OS=Homo sapiens GN=hCG_1821276 PE=4 SV=1	A0A024RCJ8_HUMAN (+2)	68 kDa	0.62	[]	0	0	2	1	0	0
HCG1991735, isoform CRA_a OS=Homo sapiens GN=hCG_1991735 PE=4 SV=1	A0A024RC65_HUMAN (+2)	189 kDa	0.053	[]	18	24	34	118	97	70
HCG1996054, isoform CRA_d OS=Homo sapiens GN=hCG_1996054 PE=3 SV=1	D3DV69_HUMAN	19 kDa	0.16	[]	0	0	0	2	1	0
HCG1998851, isoform CRA_c OS=Homo sapiens GN=hCG_1998851 PE=4 SV=1	A0A024QZ62_HUMAN (+4)	47 kDa	0.42	[]	3	2	9	10	11	8
HCG2005638, isoform CRA_a OS=Homo sapiens GN=hCG_2005638 PE=4 SV=1	A0A024RCM3_HUMAN (+1)	49 kDa	0.15	[]	0	0	2	2	2	6

HCG2016482, isoform CRA_b OS=Homo sapiens GN=hCG_2016482 PE=4 SV=1	A0A024RB41_HUMAN (+7)	23 kDa	0.48	[]	2	0	3	2	1	2
HCG2032701, isoform CRA_a OS=Homo sapiens GN=hCG_2032701 PE=4 SV=1	A0A024R3J7_HUMAN (+1)	80 kDa	0.2	[]	1	2	2	3	3	6
HECT, UBA and WWE domain containing 1, isoform CRA_c OS=Homo sapiens GN=HUWE1 PE=4 SV=1	A0A024R9U8_HUMAN (+3)	481 kDa	0.048	BRAC1 high, Naive low	0	0	0	4	3	12
HLA-B associated transcript 3, isoform CRA_a OS=Homo sapiens GN=BAT3 PE=1 SV=1	A0A024RCR6_HUMAN (+3)	119 kDa	0.03	BRAC1 high, Naive low	0	0	0	1	1	3
HSPA9 protein (Fragment) OS=Homo sapiens GN=HSPA9 PE=2 SV=1	Q8N1C8_HUMAN	74 kDa	0.26	[]	16	15	15	19	22	20
Heat shock 60kDa protein 1 (Chaperonin), isoform CRA_a OS=Homo sapiens GN=HSPD1 PE=3 SV=1	A0A024R3X4_HUMAN (+1)	61 kDa	0.15	[]	15	22	14	32	50	53
Heat shock 70 kDa protein 4 OS=Homo sapiens GN=HSPA4 PE=1 SV=1	A0A087WYC1_HUMAN (+4)	79 kDa	0.15	[]	0	0	3	3	3	6
Heat shock 70 kDa protein 14 OS=Homo sapiens GN=HSPA14 PE=1 SV=1	HSP7E_HUMAN	55 kDa	0.37	[]	0	0	0	0	0	2
Heat shock 105kDa/110kDa protein 1, isoform CRA_a OS=Homo sapiens GN=HSPH1 PE=3 SV=1	A0A024RDQ0_HUMAN (+2)	92 kDa	0.015	BRAC1 high, Naive low	0	0	2	9	11	24
Heat shock 70kDa protein 2, isoform CRA_a OS=Homo sapiens GN=HSPA2 PE=3 SV=1	A0A024R6B5_HUMAN (+1)	70 kDa	0.12	[]	16	13	18	20	15	18
Heat shock cognate 71 kDa protein OS=Homo sapiens GN=HSPA8 PE=1 SV=1	HSP7C_HUMAN (+1)	71 kDa	0.43	[]	24	20	27	44	42	49
Heat shock protein HSP 90-alpha OS=Homo sapiens GN=HSP90AA1 PE=1 SV=5	HS90A_HUMAN (+1)	85 kDa	0.42	[]	9	4	19	18	15	48
Heat shock protein beta-1 OS=Homo sapiens GN=HSPB1 PE=1 SV=2	HSPB1_HUMAN (+1)	23 kDa	0.41	[]	4	3	11	2	1	15
Helicase SKI2W OS=Homo sapiens GN=SKIV2L PE=1 SV=3	SKIV2_HUMAN (+7)	138 kDa	0.37	[]	0	0	0	0	0	5
Hemoglobin subunit alpha OS=Homo sapiens GN=HBA1 PE=1 SV=2	HBA_HUMAN (+4)	15 kDa	0.092	[]	1	2	6	0	0	2
Heparan sulfate 2-O-sulfotransferase 1 OS=Homo sapiens GN=HS2ST1 PE=1 SV=1	HS2ST_HUMAN (+1)	42 kDa	0.37	[]	0	0	0	0	0	2
Heparan sulfate proteoglycan 2 (Perlecan), isoform CRA_b OS=Homo sapiens GN=HSPG2 PE=4 SV=1	A0A024RAB6_HUMAN (+1)	464 kDa	0.25	[]	9	6	0	2	4	0
Hephaestin OS=Homo sapiens GN=HEPH PE=1 SV=1	A0A0C4DG76_HUMAN (+3)	131 kDa	0.16	[]	0	0	0	1	2	0
Heterogeneous nuclear ribonucleoprotein A1, isoform CRA_b OS=Homo sapiens GN=HNRPA1 PE=4 SV=1	A0A024RAZ7_HUMAN (+4)	39 kDa	0.68	[]	1	1	13	1	1	13
Heterogeneous nuclear ribonucleoprotein A2/B1, isoform CRA_c OS=Homo sapiens GN=HNRPA2B1 PE=4 SV=1	A0A024RA61_HUMAN (+1)	37 kDa	0.81	[]	0	1	16	1	2	16
Heterogeneous nuclear ribonucleoprotein A3 OS=Homo sapiens GN=HNRNPA3 PE=1 SV=2	ROA3_HUMAN (+2)	40 kDa	0.53	[]	1	1	4	0	0	6
Heterogeneous nuclear ribonucleoprotein A/B OS=Homo sapiens GN=HNRNPAB PE=1 SV=1	A0A087WZV1_HUMAN (+5)	37 kDa	0.25	[]	1	1	3	1	2	2
Heterogeneous nuclear ribonucleoprotein D (AU-rich element RNA binding protein 1, 37kDa), isoform CRA_d OS=Homo sapiens GN=H	A0A024RDF3_HUMAN (+2)	38 kDa	0.69	[]	0	1	5	1	0	5
Heterogeneous nuclear ribonucleoprotein D-like OS=Homo sapiens GN=HNRNPDL PE=1 SV=3	HNRDL_HUMAN (+3)	46 kDa	0.37	[]	0	0	3	0	0	0
Heterogeneous nuclear ribonucleoprotein F, isoform CRA_a OS=Homo sapiens GN=HNRPF PE=4 SV=1	A0A024R7T3_HUMAN (+1)	46 kDa	0.28	[]	0	1	1	1	2	9
Heterogeneous nuclear ribonucleoprotein H3 OS=Homo sapiens GN=HNRNPH3 PE=1 SV=2	HNRH3_HUMAN (+4)	37 kDa	0.87	[]	0	0	1	0	0	2
Heterogeneous nuclear ribonucleoprotein H OS=Homo sapiens GN=HNRNPH1 PE=1 SV=1	E9PCY7_HUMAN (+2)	47 kDa	0.9	[]	0	2	5	2	2	8
Heterogeneous nuclear ribonucleoprotein K, isoform CRA_d OS=Homo sapiens GN=HNRPK PE=4 SV=1	A0A024R228_HUMAN (+2)	51 kDa	0.97	[]	3	2	10	6	4	14
Heterogeneous nuclear ribonucleoprotein L OS=Homo sapiens GN=HNRNPL PE=1 SV=2	HNRPL_HUMAN (+2)	64 kDa	0.76	[]	0	1	2	0	0	8
Heterogeneous nuclear ribonucleoprotein Q OS=Homo sapiens GN=SYNCRIP PE=1 SV=2	HNRPQ_HUMAN (+1)	70 kDa	0.91	[]	1	2	6	1	2	11
cDNA, FLJ93632, highly similar to Homo sapiens heterogeneous nuclear ribonucleoprotein R (HNRPR), mRNA OS=Homo sapiens PE=2	B2R7W4_HUMAN (+3)	71 kDa	0.96	[]	0	0	2	1	0	2
Heterogeneous nuclear ribonucleoproteins C1/C2 OS=Homo sapiens GN=HNRNPC PE=1 SV=4	HNRPC_HUMAN (+7)	34 kDa	0.37	[]	0	0	4	0	0	0
Hexokinase OS=Homo sapiens PE=2 SV=1	A8K7J7_HUMAN (+3)	102 kDa	0.036	BRAC1 low, Naive high	5	9	6	2	4	6
Putative hexokinase HKDC1 OS=Homo sapiens GN=HKDC1 PE=1 SV=3	HKDC1_HUMAN (+3)	103 kDa	1	[]	0	0	0	0	0	0
High-mobility group box 1, isoform CRA_a OS=Homo sapiens GN=HMGB1 PE=4 SV=1	A0A024RDR0_HUMAN (+1)	25 kDa	0.35	[]	0	0	2	1	3	2
Histidine--tRNA ligase, cytoplasmic OS=Homo sapiens GN=HARS PE=1 SV=1	B3KWE1_HUMAN (+3)	50 kDa	0.44	[]	0	0	1	2	1	0
Histone H1.0 OS=Homo sapiens GN=H1F0 PE=1 SV=3	H10_HUMAN	21 kDa	0.056	[]	1	1	2	0	0	2
Histone H1.2 OS=Homo sapiens GN=HIST1H1C PE=1 SV=2	H12_HUMAN	21 kDa	0.63	[]	1	1	3	0	0	6
Histone H1.4 OS=Homo sapiens GN=HIST1H1E PE=1 SV=2	H14_HUMAN (+4)	22 kDa	0.47	[]	1	1	3	0	0	5
Histone H1.5 OS=Homo sapiens GN=HIST1H1B PE=1 SV=3	H15_HUMAN	23 kDa	0.69	[]	0	0	1	0	0	3
Histone H2A type 2-A OS=Homo sapiens GN=HIST2H2AA3 PE=1 SV=3	H2A2A_HUMAN (+1)	14 kDa	0.16	[]	1	2	1	1	0	2
Histone H2A type 2-B OS=Homo sapiens GN=HIST2H2AB PE=1 SV=3	H2A2B_HUMAN (+1)	14 kDa	0.92	[]	0	0	3	0	0	4
Histone H2B OS=Homo sapiens GN=HIST1H2BD PE=3 SV=1	A0A024QZZ7_HUMAN (+16)	14 kDa	0.6	[]	1	1	3	2	2	7
Histone H2B OS=Homo sapiens GN=HIST1H2BJ PE=3 SV=1	A0A024RCJ2_HUMAN (+5)	14 kDa	0.89	[]	1	1	3	1	1	6
Histone H3 OS=Homo sapiens PE=1 SV=1	A8K4Y7_HUMAN (+4)	15 kDa	0.15	[]	0	0	1	1	1	2
Histone H3.2 OS=Homo sapiens GN=HIST2H3A PE=1 SV=3	H32_HUMAN	15 kDa	0.00029	BRAC1 high, Naive low	0	0	0	1	1	1
Histone H4 OS=Homo sapiens GN=HIST1H4L PE=2 SV=1	B2R4R0_HUMAN (+2)	11 kDa	0.7	[]	5	3	3	6	6	5
Histone deacetylase 6 OS=Homo sapiens GN=HDAC6 PE=1 SV=2	HDAC6_HUMAN (+8)	131 kDa	0.37	[]	0	0	0	0	0	3
Hormone-sensitive lipase OS=Homo sapiens GN=LIPE PE=1 SV=4	LIPS_HUMAN (+1)	117 kDa	0.37	[]	0	0	0	0	0	9
Huntingtin interacting protein 1 related, isoform CRA_a OS=Homo sapiens GN=HIP1R PE=4 SV=1	A0A024RBR4_HUMAN (+1)	99 kDa	0.031	BRAC1 high, Naive low	0	0	0	2	1	1
Hydroxyacyl-coenzyme A dehydrogenase, mitochondrial OS=Homo sapiens GN=HADH PE=1 SV=1	A0A0A0MSE2_HUMAN (+2)	42 kDa	0.91	[]	2	0	0	1	2	2
Hydroxymethylglutaryl-CoA synthase, cytoplasmic OS=Homo sapiens GN=HMGCS1 PE=1 SV=2	HMCS1_HUMAN (+3)	57 kDa	0.37	[]	0	0	0	0	0	5
Hydroxysteroid (17-beta) dehydrogenase 12, isoform CRA_a OS=Homo sapiens GN=HSD17B12 PE=3 SV=1	D3DR22_HUMAN (+1)	39 kDa	0.53	[]	1	1	6	3	4	12
Hydroxysteroid dehydrogenase-like protein 2 OS=Homo sapiens GN=HSDL2 PE=1 SV=1	HSDL2_HUMAN	45 kDa	0.87	[]	1	1	3	1	1	8
Hypoxia up-regulated protein 1 OS=Homo sapiens GN=HYOU1 PE=1 SV=1	HYOU1_HUMAN	111 kDa	0.075	[]	2	2	9	12	20	43
IQ motif containing GTPase activating protein 2, isoform CRA_b OS=Homo sapiens GN=IQGAP2 PE=4 SV=1	A0A024RAJ8_HUMAN	181 kDa	0.025	BRAC1 high, Naive low	0	0	0	52	54	23
cDNA FLJ52376, highly similar to Ras GTPase-activating-like protein IQGAP2 OS=Homo sapiens PE=2 SV=1	B7Z881_HUMAN	99 kDa	0.034	BRAC1 high, Naive low	0	0	0	28	22	10
IQ motif containing GTPase activating protein 3 OS=Homo sapiens GN=IQGAP3 PE=1 SV=1	A2RRC9_HUMAN (+2)	185 kDa	0.031	BRAC1 high, Naive low	0	0	0	4	2	2
ITGAV protein OS=Homo sapiens GN=ITGAV PE=2 SV=1	A5YM53_HUMAN (+2)	116 kDa	0.68	[]	5	10	15	17	16	16
Immunoglobulin superfamily member 3 OS=Homo sapiens GN=IGSF3 PE=2 SV=3	IGSF3_HUMAN	135 kDa	0.031	BRAC1 high, Naive low	0	0	0	4	2	2
Immunoglobulin superfamily member 8 OS=Homo sapiens GN=IGSF8 PE=1 SV=1	IGSF8_HUMAN	65 kDa	0.58	[]	1	0	0	3	1	0
Importin subunit alpha-3 OS=Homo sapiens GN=KPNA4 PE=1 SV=1	IMA3_HUMAN	58 kDa	0.27	[]	0	0	2	2	2	2

Importin-4 OS=Homo sapiens GN=IPO4 PE=1 SV=2	IPO4_HUMAN (+1)	119 kDa	0.26	[]	0	0	0	0	2	13
Importin-7 OS=Homo sapiens GN=IPO7 PE=1 SV=1	IPO7_HUMAN	120 kDa	0.31	[]	0	0	3	1	3	11
Importin-9 OS=Homo sapiens GN=IPO9 PE=1 SV=3	IPO9_HUMAN	116 kDa	0.018	BRAC1 high, Naive low	0	0	0	3	3	8
Inactive tyrosine-protein kinase 7 OS=Homo sapiens GN=PTK7 PE=1 SV=2	PTK7_HUMAN	118 kDa	0.44	[]	6	11	13	16	15	1
Inhibitor of nuclear factor kappa-B kinase-interacting protein OS=Homo sapiens GN=IKBIP PE=1 SV=1	IKIP_HUMAN	39 kDa	0.00091	BRAC1 low, Naive high	2	2	4	0	0	0
Inorganic pyrophosphatase 2, mitochondrial OS=Homo sapiens GN=PPA2 PE=1 SV=2	IPYR2_HUMAN (+3)	38 kDa	0.26	[]	0	0	1	0	2	4
Inorganic pyrophosphatase OS=Homo sapiens GN=PPA1 PE=1 SV=2	IPYR_HUMAN (+1)	33 kDa	0.14	[]	0	0	0	1	2	9
Inosine-5'-monophosphate dehydrogenase 2 (Fragment) OS=Homo sapiens GN=IMPDH2 PE=1 SV=1	H0Y4R1_HUMAN (+1)	51 kDa	0.38	[]	1	0	2	2	1	18
Inositol 1,4,5-trisphosphate receptor type 3 OS=Homo sapiens GN=ITPR3 PE=1 SV=2	ITPR3_HUMAN (+1)	304 kDa	0.17	[]	0	0	0	0	2	6
Inositol monophosphatase 3 OS=Homo sapiens GN=IMPAD1 PE=1 SV=1	IMPA3_HUMAN (+1)	39 kDa	0.86	[]	0	1	1	0	0	3
Insulin receptor substrate 2 OS=Homo sapiens GN=IRS2 PE=1 SV=2	IRS2_HUMAN (+2)	137 kDa	0.016	BRAC1 high, Naive low	0	0	0	3	4	2
Insulin-like growth factor 2 mRNA-binding protein 2 OS=Homo sapiens GN=IGF2BP2 PE=1 SV=2	IF2B2_HUMAN (+1)	66 kDa	0.25	[]	2	4	4	0	1	8
Insulin-like growth factor 2 mRNA-binding protein 3 OS=Homo sapiens GN=IGF2BP3 PE=1 SV=2	IF2B3_HUMAN	64 kDa	0.21	[]	0	0	1	1	2	10
Insulin-like growth factor-binding protein 5 OS=Homo sapiens GN=IGFBP5 PE=1 SV=1	IBP5_HUMAN (+1)	31 kDa	0.37	[]	0	0	2	0	0	0
Integrin alpha-2 OS=Homo sapiens GN=ITGA2 PE=1 SV=1	ITA2_HUMAN	129 kDa	0.2	[]	10	12	14	41	34	20
Integrin alpha-2 OS=Homo sapiens GN=ITGA2 PE=1 SV=1	E7ESP4_HUMAN	103 kDa	0.18	[]	8	12	13	40	33	19
Integrin alpha-5 OS=Homo sapiens GN=ITGA5 PE=1 SV=2	ITA5_HUMAN (+1)	115 kDa	0.00086	BRAC1 low, Naive high	7	7	8	0	0	0
Integrin alpha-6 OS=Homo sapiens GN=ITGA6 PE=1 SV=5	ITA6_HUMAN	127 kDa	0.0036	BRAC1 high, Naive low	0	0	0	41	36	30
Integrin alpha-11 OS=Homo sapiens GN=ITGA11 PE=1 SV=2	ITA11_HUMAN	133 kDa	0.37	[]	0	0	5	0	0	0
Integrin beta OS=Homo sapiens GN=ITGB4 PE=3 SV=1	A0A024R8K7_HUMAN (+2)	195 kDa	0.0049	BRAC1 high, Naive low	0	0	0	59	54	41
Integrin beta OS=Homo sapiens PE=2 SV=1	A8K2N5_HUMAN (+3)	86 kDa	0.013	BRAC1 high, Naive low	0	0	0	2	3	6
Integrin beta-1 OS=Homo sapiens GN=ITGB1 PE=1 SV=2	ITB1_HUMAN	88 kDa	0.77	[]	14	18	21	38	39	17
Integrin beta-5 OS=Homo sapiens GN=ITGB5 PE=1 SV=1	ITB5_HUMAN (+1)	88 kDa	0.061	[]	1	1	2	3	5	9
Intercellular adhesion molecule 1 OS=Homo sapiens GN=ICAM1 PE=1 SV=2	ICAM1_HUMAN (+1)	58 kDa	0.003	BRAC1 low, Naive high	7	11	10	3	4	2
Interferon-induced GTP-binding protein Mx1 OS=Homo sapiens GN=MX1 PE=1 SV=4	MX1_HUMAN	76 kDa	0.0051	BRAC1 low, Naive high	8	11	21	3	4	6
Interferon-induced GTP-binding protein Mx2 OS=Homo sapiens GN=MX2 PE=1 SV=1	MX2_HUMAN	82 kDa	0.00012	BRAC1 low, Naive high	6	7	11	2	2	4
Interferon-induced protein with tetratricopeptide repeats 1 OS=Homo sapiens GN=IFIT1 PE=1 SV=2	IFIT1_HUMAN	55 kDa	0.48	[]	0	2	11	2	1	5
Interferon-induced protein with tetratricopeptide repeats 2 OS=Homo sapiens GN=IFIT2 PE=1 SV=1	IFIT2_HUMAN (+2)	55 kDa	0.37	[]	0	0	2	0	0	0
Interferon-induced protein with tetratricopeptide repeats 3 OS=Homo sapiens GN=IFIT3 PE=1 SV=1	IFIT3_HUMAN (+1)	56 kDa	0.44	[]	1	0	3	0	0	3
Interferon-induced protein with tetratricopeptide repeats 5 OS=Homo sapiens GN=IFIT5 PE=1 SV=1	IFIT5_HUMAN	56 kDa	0.37	[]	0	0	0	0	0	3
Interleukin enhancer-binding factor 2 OS=Homo sapiens GN=ILF2 PE=1 SV=2	ILF2_HUMAN (+2)	43 kDa	0.66	[]	0	0	4	0	0	3
Interleukin enhancer-binding factor 3 OS=Homo sapiens GN=ILF3 PE=1 SV=3	ILF3_HUMAN (+5)	95 kDa	0.52	[]	0	0	3	0	1	0
Inverted formin-2 OS=Homo sapiens GN=INF2 PE=1 SV=2	INF2_HUMAN	136 kDa	0.11	[]	1	2	4	4	8	13
Isocitrate dehydrogenase [NADP] cytoplasmic OS=Homo sapiens GN=IDH1 PE=1 SV=2	IDHC_HUMAN (+3)	47 kDa	0.00054	BRAC1 high, Naive low	0	0	1	7	6	10
Isocitrate dehydrogenase [NADP], mitochondrial OS=Homo sapiens GN=IDH2 PE=1 SV=2	IDHP_HUMAN (+1)	51 kDa	0.04	BRAC1 low, Naive high	10	9	7	1	6	4
Isocitrate dehydrogenase [NAD] subunit, mitochondrial OS=Homo sapiens PE=2 SV=1	B4DJB4_HUMAN (+6)	35 kDa	0.31	[]	1	1	2	1	1	3
Isoleucyl-tRNA synthetase, cytoplasmic variant (Fragment) OS=Homo sapiens PE=2 SV=1	Q59G75_HUMAN (+1)	146 kDa	0.36	[]	0	0	2	1	1	20
Junction plakoglobin, isoform CRA_a OS=Homo sapiens GN=JUP PE=4 SV=1	A0A024R1X8_HUMAN (+1)	82 kDa	0.019	BRAC1 high, Naive low	0	1	2	75	69	37
Junction adhesion molecule OS=Homo sapiens PE=2 SV=1	Q9Y5B2_HUMAN	28 kDa	0.0046	BRAC1 high, Naive low	0	0	0	10	9	7
Junctional adhesion molecule A OS=Homo sapiens GN=F11R PE=1 SV=1	A0A087WY82_HUMAN (+4)	30 kDa	0.0015	BRAC1 high, Naive low	0	0	0	12	11	10
KIAA0528, isoform CRA_b OS=Homo sapiens GN=KIAA0528 PE=4 SV=1	A0A024RB03_HUMAN (+3)	121 kDa	0.028	BRAC1 high, Naive low	0	0	0	1	2	1
KIF1-binding protein OS=Homo sapiens GN=KIF1BP PE=1 SV=1	KBP_HUMAN	72 kDa	0.37	[]	0	0	0	0	0	2
KN motif and ankyrin repeat domain-containing protein 2 OS=Homo sapiens GN=KANK2 PE=1 SV=1	KANK2_HUMAN	91 kDa	0.01	BRAC1 low, Naive high	2	3	2	0	0	1
Kallikrein 6 (Neurosin, zyme), isoform CRA_b OS=Homo sapiens GN=KLK6 PE=3 SV=1	A0A024R4J8_HUMAN (+1)	27 kDa	0.0027	BRAC1 high, Naive low	0	0	0	1	1	2
Keratin 18, isoform CRA_a OS=Homo sapiens GN=KRT18 PE=3 SV=1	A0A024RAY2_HUMAN (+2)	48 kDa	0.0015	BRAC1 high, Naive low	4	3	7	42	44	39
Keratin, type I cytoskeletal 9 OS=Homo sapiens GN=KRT9 PE=1 SV=3	K1C9_HUMAN	62 kDa	0.088	[]	8	4	18	4	7	2
Keratin, type I cytoskeletal 10 OS=Homo sapiens GN=KRT10 PE=1 SV=6	K1C10_HUMAN (+1)	59 kDa	0.22	[]	10	8	15	2	20	2
Keratin 14 (Epidermolysis bullosa simplex, Dowling-Meara, Koebner), isoform CRA_a OS=Homo sapiens GN=KRT14 PE=3 SV=1	A0A024R1X6_HUMAN	30 kDa	0.7	[]	1	1	0	1	3	1
Keratin, type I cytoskeletal 14 OS=Homo sapiens GN=KRT14 PE=1 SV=4	K1C14_HUMAN	52 kDa	0.066	[]	3	2	2	0	2	0
Keratin, type I cytoskeletal 16 OS=Homo sapiens GN=KRT16 PE=1 SV=4	K1C16_HUMAN	51 kDa	0.37	[]	0	0	2	0	0	0
Keratin, type I cytoskeletal 17 OS=Homo sapiens GN=KRT17 PE=1 SV=2	K1C17_HUMAN	48 kDa	0.37	[]	0	0	0	0	2	0
Keratin, type I cytoskeletal 19 OS=Homo sapiens GN=KRT19 PE=1 SV=4	K1C19_HUMAN	44 kDa	0.12	[]	2	4	7	13	15	9
Keratin, type I cytoskeletal 20 OS=Homo sapiens GN=KRT20 PE=1 SV=1	K1C20_HUMAN	48 kDa	0.04	BRAC1 high, Naive low	0	0	1	3	4	2
Keratin, type II cytoskeletal 7 OS=Homo sapiens GN=KRT7 PE=1 SV=5	K2C7_HUMAN	51 kDa	0.0063	BRAC1 low, Naive high	5	6	14	0	0	0
Keratin, type II cytoskeletal 72 OS=Homo sapiens GN=KRT72 PE=1 SV=2	K2C72_HUMAN	56 kDa	0.71	[]	0	0	3	0	2	0
cDNA FLJ46620 fis, clone TLUNG2000654, highly similar to Keratin, type II cytoskeletal 7 OS=Homo sapiens PE=2 SV=1	B3KY79_HUMAN	49 kDa	0.04	BRAC1 low, Naive high	3	4	13	0	0	0
Keratin 1 OS=Homo sapiens GN=KRT1 PE=3 SV=1	H6VRF8_HUMAN (+4)	66 kDa	0.24	[]	26	9	27	11	25	20
Keratin, type II cytoskeletal 2 epidermal OS=Homo sapiens GN=KRT2 PE=1 SV=2	K22E_HUMAN (+1)	65 kDa	0.44	[]	4	2	12	4	10	1
Keratin, type II cytoskeletal 6B OS=Homo sapiens GN=KRT6B PE=1 SV=5	K2C6B_HUMAN	60 kDa	0.37	[]	0	0	4	0	0	0
Keratin, type II cytoskeletal 8 OS=Homo sapiens GN=KRT8 PE=1 SV=7	K2C8_HUMAN	54 kDa	0.0029	BRAC1 high, Naive low	1	3	5	49	35	41
cDNA FLJ54081, highly similar to Keratin, type II cytoskeletal 5 OS=Homo sapiens PE=2 SV=1	B4E1T1_HUMAN (+1)	59 kDa	0.37	[]	1	0	0	0	0	0
Keratin, type II cytoskeletal 80 OS=Homo sapiens GN=KRT80 PE=1 SV=2	K2C80_HUMAN	51 kDa	0.37	[]	0	0	0	0	0	12

Kin of IRRE-like protein 1 OS=Homo sapiens GN=KIRREL PE=1 SV=2	KIRR1_HUMAN	84 kDa	0.028	BRAC1 low, Naive high	2	2	1	0	0	0
Kinectin 1 (Kinesin receptor), isoform CRA_a OS=Homo sapiens GN=KTN1 PE=4 SV=1	A0A024R663_HUMAN (+1)	156 kDa	0.24	[]	5	9	3	6	5	5
Kinesin light chain 4, isoform CRA_a OS=Homo sapiens GN=KLC4 PE=4 SV=1	A0A024RCZ8_HUMAN (+1)	69 kDa	0.0027	BRAC1 high, Naive low	0	0	0	1	1	2
Kinesin-like protein KIF13B OS=Homo sapiens GN=KIF13B PE=1 SV=2	KI13B_HUMAN (+1)	203 kDa	0.37	[]	0	0	0	0	0	3
Kinesin-like protein OS=Homo sapiens GN=KIF5B PE=3 SV=1	D3DRX6_HUMAN (+2)	110 kDa	0.82	[]	1	3	12	8	6	13
Kinesin-like protein OS=Homo sapiens GN=KIF23 PE=1 SV=1	H7BYN4_HUMAN (+1)	109 kDa	0.019	BRAC1 high, Naive low	0	0	1	11	14	7
L-lactate dehydrogenase A chain OS=Homo sapiens GN=LDHA PE=1 SV=2	LDHA_HUMAN (+1)	37 kDa	0.25	[]	9	7	17	11	11	20
L-lactate dehydrogenase B chain OS=Homo sapiens GN=LDHB PE=1 SV=2	LDHB_HUMAN (+1)	37 kDa	0.14	[]	3	2	7	8	10	11
L-xylulose reductase OS=Homo sapiens GN=DCXR PE=1 SV=2	DCXR_HUMAN (+5)	26 kDa	0.028	BRAC1 high, Naive low	0	0	0	1	2	1
LDLR chaperone MESD OS=Homo sapiens GN=MESDC2 PE=1 SV=2	MESD_HUMAN	26 kDa	0.54	[]	0	0	2	0	1	6
LIM and SH3 protein 1, isoform CRA_b OS=Homo sapiens GN=LASP1 PE=4 SV=1	A0A024R1S8_HUMAN (+2)	30 kDa	0.61	[]	0	2	2	3	2	3
LIM domain and actin-binding protein 1 OS=Homo sapiens GN=LIMA1 PE=1 SV=1	LIMA1_HUMAN (+1)	85 kDa	0.004	BRAC1 high, Naive low	3	5	12	27	26	31
LIM domain only 7 protein OS=Homo sapiens GN=LMO7 PE=2 SV=1	F8J2B5_HUMAN (+3)	186 kDa	0.55	[]	5	11	13	13	18	3
LIM domain only protein 7 OS=Homo sapiens GN=LMO7 PE=1 SV=1	E9PMS6_HUMAN	145 kDa	0.69	[]	4	12	11	13	18	3
Lactadherin OS=Homo sapiens GN=MFGE8 PE=1 SV=2	MFGM_HUMAN (+1)	43 kDa	0.0071	BRAC1 low, Naive high	12	13	32	0	0	2
tr|X6R3G6|X6R3G6_HUMAN Lactadherin OS=Homo sapiens GN=MFGE8 PE=1 SV=1	X6R3G6_HUMAN	?	0.006	BRAC1 low, Naive high	11	13	30	0	0	2
Ladinin 1 OS=Homo sapiens GN=LAD1 PE=2 SV=1	Q6IPJ9_HUMAN	57 kDa	0.003	BRAC1 high, Naive low	0	0	0	6	4	5
Lamin-B1 OS=Homo sapiens GN=LMNB1 PE=1 SV=2	LMNB1_HUMAN	66 kDa	0.37	[]	0	0	0	0	0	2
Lamin-B2 OS=Homo sapiens GN=LMNB2 PE=1 SV=4	LMNB2_HUMAN (+1)	70 kDa	0.29	[]	0	1	6	0	0	1
Laminin subunit alpha-4 OS=Homo sapiens GN=LAMA4 PE=1 SV=4	LAMA4_HUMAN (+5)	203 kDa	0.37	[]	0	3	0	0	0	0
Laminin subunit beta-2 OS=Homo sapiens GN=LAMB2 PE=1 SV=2	LAMB2_HUMAN (+1)	196 kDa	0.0023	BRAC1 low, Naive high	3	3	3	0	0	0
Laminin subunit gamma-1 OS=Homo sapiens GN=LAMC1 PE=1 SV=3	LAMC1_HUMAN (+1)	178 kDa	0.0023	BRAC1 low, Naive high	5	5	5	0	0	0
Lanosterol 14-alpha demethylase OS=Homo sapiens GN=CYP51A1 PE=1 SV=3	CP51A_HUMAN (+2)	57 kDa	0.59	[]	0	0	2	0	1	5
Lanosterol synthase OS=Homo sapiens GN=LSS PE=1 SV=1	ERG7_HUMAN	83 kDa	0.15	[]	0	0	1	1	1	2
Large neutral amino acids transporter 1 (Fragment) OS=Homo sapiens GN=SLC7A5 PE=4 SV=1	Q2MCL6_HUMAN	7 kDa	0.13	[]	0	0	0	8	6	0
Large neutral amino acids transporter small subunit 1 OS=Homo sapiens GN=SLC7A5 PE=1 SV=2	LAT1_HUMAN	55 kDa	0.22	[]	4	4	5	13	9	9
Lck-interacting transmembrane adapter 1 OS=Homo sapiens GN=LIME1 PE=1 SV=1	LIME1_HUMAN	31 kDa	0.0027	BRAC1 high, Naive low	0	0	0	2	2	4
Lectin, mannose-binding, 1, isoform CRA_b OS=Homo sapiens GN=LMAN1 PE=4 SV=1	A0A024R2A7_HUMAN (+2)	58 kDa	0.88	[]	1	2	4	1	2	8
Leucine rich repeat containing 47, isoform CRA_a OS=Homo sapiens GN=LRRC47 PE=4 SV=1	A0A024R4G1_HUMAN (+1)	63 kDa	0.67	[]	1	1	0	2	1	2
Leucine zipper-EF-hand containing transmembrane protein 1, isoform CRA_a OS=Homo sapiens GN=LETM1 PE=4 SV=1	D3DVQ1_HUMAN (+1)	64 kDa	0.15	[]	0	1	1	2	2	2
Leucine-rich PPR-motif containing OS=Homo sapiens GN=LRPPRC PE=4 SV=1	E5KNY5_HUMAN (+1)	158 kDa	0.081	[]	2	1	8	8	19	21
Leucine-rich repeat and calponin homology domain-containing protein 1 OS=Homo sapiens GN=LRCH1 PE=1 SV=3	LRCH1_HUMAN (+1)	81 kDa	0.37	[]	0	0	0	0	3	0
Leucine-rich repeat-containing protein 59 OS=Homo sapiens GN=LRRC59 PE=1 SV=1	LRC59_HUMAN	35 kDa	0.077	[]	5	9	6	3	7	6
Lipid phosphate phosphohydrolase 1 OS=Homo sapiens GN=PPAP2A PE=1 SV=1	LPP1_HUMAN (+1)	32 kDa	0.6	[]	0	0	1	0	0	4
Lipid phosphate phosphohydrolase 2 (Fragment) OS=Homo sapiens GN=PPAP2C PE=1 SV=1	A0A0J9YXA3_HUMAN (+3)	24 kDa	0.12	[]	0	0	0	2	0	2
Lipolysis-stimulated lipoprotein receptor OS=Homo sapiens GN=LSR PE=1 SV=4	LSR_HUMAN (+1)	71 kDa	0.0068	BRAC1 high, Naive low	0	0	0	14	12	9
Lipopolysaccharide-responsive and beige-like anchor protein OS=Homo sapiens GN=LRBA PE=1 SV=1	E9PEM5_HUMAN (+3)	287 kDa	0.37	[]	0	0	0	0	0	4
LisH domain-containing protein ARMC9 OS=Homo sapiens GN=ARMC9 PE=1 SV=2	ARMC9_HUMAN (+2)	92 kDa	0.87	[]	0	0	1	0	0	2
Lon protease homolog OS=Homo sapiens PE=2 SV=1	B3KU28_HUMAN (+6)	95 kDa	0.0089	BRAC1 low, Naive high	3	4	6	1	2	5
Long-chain-fatty-acid--CoA ligase 1 OS=Homo sapiens GN=ACSL1 PE=1 SV=1	ACSL1_HUMAN (+4)	78 kDa	0.37	[]	0	0	0	0	0	2
Long-chain-fatty-acid--CoA ligase 4 OS=Homo sapiens GN=ACSL4 PE=1 SV=2	ACSL4_HUMAN (+1)	79 kDa	0.049	BRAC1 high, Naive low	3	2	4	7	11	18
Long-chain-fatty-acid--CoA ligase 5 OS=Homo sapiens GN=ACSL5 PE=1 SV=1	ACSL5_HUMAN	76 kDa	0.0027	BRAC1 high, Naive low	0	0	0	6	6	12
Low-density lipoprotein receptor OS=Homo sapiens GN=LDLR PE=1 SV=1	LDLR_HUMAN (+4)	95 kDa	0.37	[]	0	0	0	0	0	3
Lymphocyte antigen 75 OS=Homo sapiens GN=LY75 PE=1 SV=3	LY75_HUMAN	198 kDa	0.093	[]	0	0	0	10	16	2
Lymphocyte antigen 75 variant (Fragment) OS=Homo sapiens PE=2 SV=1	Q59H44_HUMAN	155 kDa	0.12	[]	0	0	0	10	12	0
Lymphocyte function-associated antigen 3 OS=Homo sapiens GN=CD58 PE=1 SV=1	LFA3_HUMAN (+5)	28 kDa	0.37	[]	0	0	0	0	2	0
Lysine--tRNA ligase OS=Homo sapiens GN=KARS PE=1 SV=3	SYK_HUMAN	68 kDa	0.17	[]	0	0	0	1	1	7
Lysophosphatidic acid receptor 1 OS=Homo sapiens GN=LPAR1 PE=1 SV=3	LPAR1_HUMAN (+3)	41 kDa	0.00091	BRAC1 low, Naive high	1	1	2	0	0	0
Lysophosphatidylcholine acyltransferase 1 OS=Homo sapiens GN=LPCAT1 PE=1 SV=2	PCAT1_HUMAN	59 kDa	0.38	[]	0	0	1	0	1	5
Lysophosphatidylcholine acyltransferase 2 OS=Homo sapiens GN=LPCAT2 PE=1 SV=1	PCAT2_HUMAN	60 kDa	0.11	[]	0	0	0	1	1	5
Lysophospholipid acyltransferase 7 OS=Homo sapiens GN=MBOAT7 PE=1 SV=2	MBOA7_HUMAN	53 kDa	0.15	[]	0	2	3	4	4	9
Lysophospholipid acyltransferase LPCAT4 OS=Homo sapiens GN=LPCAT4 PE=1 SV=1	LPCT4_HUMAN (+1)	57 kDa	0.37	[]	0	0	0	0	0	4
Lysosomal Pro-X carboxypeptidase OS=Homo sapiens GN=PRCP PE=1 SV=1	PCP_HUMAN (+2)	56 kDa	0.19	[]	0	2	1	0	0	0
Lysosomal-associated membrane protein 1, isoform CRA_a OS=Homo sapiens GN=LAMP1 PE=4 SV=1	A0A024RDY3_HUMAN (+2)	45 kDa	0.1	[]	8	6	3	2	1	4
MARCKS-related protein OS=Homo sapiens GN=MARCKSL1 PE=1 SV=2	MRP_HUMAN	20 kDa	0.063	[]	1	0	0	10	10	3
MHC class I antigen (Fragment) OS=Homo sapiens GN=HLA-A PE=3 SV=1	E9LXZ0_HUMAN	21 kDa	0.24	[]	14	12	10	16	14	8
HLA class I antigen OS=Homo sapiens GN=HLA-C PE=3 SV=1	A0A090MEW7_HUMAN	41 kDa	0.079	[]	12	11	13	12	12	0
MHC class I antigen (Fragment) OS=Homo sapiens GN=HLA-A PE=3 SV=1	X5MFD7_HUMAN	38 kDa	0.12	[]	20	15	17	18	18	12
MHC class I antigen (Fragment) OS=Homo sapiens GN=HLA-A PE=3 SV=1	A6YT90_HUMAN	36 kDa	0.56	[]	15	0	10	23	25	16
MHC class I antigen (Fragment) OS=Homo sapiens GN=HLA-A PE=3 SV=1	G1ENA6_HUMAN	21 kDa	0.032	BRAC1 high, Naive low	0	0	0	18	19	7
MHC class I antigen (Fragment) OS=Homo sapiens GN=HLA-A PE=3 SV=1	D5H3V9_HUMAN (+1)	34 kDa	0.87	[]	15	9	10	23	24	17
MHC class I antigen (Fragment) OS=Homo sapiens GN=HLA-A PE=3 SV=1	F6KRM6_HUMAN	21 kDa	0.024	BRAC1 high, Naive low	0	0	0	20	21	9

MHC class I antigen (Fragment) OS=Homo sapiens GN=HLA-A PE=3 SV=1	I7GY09_HUMAN	32 kDa	0.12	[]	14	13	0	0	0	0
MHC class I antigen (Fragment) OS=Homo sapiens GN=HLA-A PE=3 SV=1	D6MLL3_HUMAN	21 kDa	0.4	[]	0	9	6	15	15	8
MHC class I antigen (Fragment) OS=Homo sapiens GN=HLA-A PE=3 SV=1	F6IQY8_HUMAN	38 kDa	0.28	[]	24	15	12	18	19	14
MHC class I antigen (Fragment) OS=Homo sapiens GN=HLA-A PE=3 SV=1	V5L1W2_HUMAN	32 kDa	0.24	[]	19	13	12	17	16	12
HLA class I histocompatibility antigen, B-44 alpha chain OS=Homo sapiens GN=HLA-B PE=1 SV=1	1B44_HUMAN (+33)	40 kDa	0.29	[]	16	16	0	10	6	5
MHC class I antigen (Fragment) OS=Homo sapiens GN=HLA-A PE=3 SV=1	A0A0H3W567_HUMAN	32 kDa	0.22	[]	13	11	10	13	15	9
MHC class I antigen (Fragment) OS=Homo sapiens GN=HLA-A PE=3 SV=1	F2XI33_HUMAN	31 kDa	0.0046	BRAC1 high, Naive low	0	0	0	18	18	13
MHC class I antigen (Fragment) OS=Homo sapiens GN=HLA-A PE=3 SV=1	U5YJN1_HUMAN	38 kDa	0.011	BRAC1 high, Naive low	0	0	0	21	23	13
MHC class I antigen (Fragment) OS=Homo sapiens GN=HLA-A PE=3 SV=1	H9C5F6_HUMAN	21 kDa	0.81	[]	14	7	0	13	15	6
MHC class I antigen (Fragment) OS=Homo sapiens GN=HLA-A PE=3 SV=1	D6MJ95_HUMAN (+23)	21 kDa	0.64	[]	12	9	0	12	14	0
MHC class I antigen (Fragment) OS=Homo sapiens GN=HLA-A PE=3 SV=1	D5G2L1_HUMAN (+2)	21 kDa	0.018	BRAC1 high, Naive low	0	0	0	18	21	10
MHC class I antigen (Fragment) OS=Homo sapiens GN=HLA-A PE=3 SV=1	J9PWW7_HUMAN	21 kDa	0.37	[]	0	0	0	0	8	0
MHC class I antigen (Fragment) OS=Homo sapiens GN=HLA-A PE=3 SV=1	C9DSL3_HUMAN	21 kDa	0.37	[]	0	0	6	0	0	0
MHC class I antigen (Fragment) OS=Homo sapiens GN=HLA-A PE=4 SV=2	A0A0F7W082_HUMAN	23 kDa	0.091	[]	0	0	9	16	15	11
MHC class I antigen (Fragment) OS=Homo sapiens GN=HLA-B PE=3 SV=1	C7FDR3_HUMAN (+1)	21 kDa	0.044	BRAC1 low, Naive high	9	10	9	8	8	4
MHC class I antigen (Fragment) OS=Homo sapiens GN=HLA-B PE=3 SV=1	A0A0G3EHC5_HUMAN	32 kDa	0.0034	BRAC1 low, Naive high	17	21	18	3	5	0
HLA-A26 protein OS=Homo sapiens GN=HLA-A26 PE=3 SV=1	O19633_HUMAN	24 kDa	1	[]	0	0	0	0	0	0
MHC class I antigen (Fragment) OS=Homo sapiens GN=HLA-B PE=3 SV=1	W0G8G7_HUMAN	32 kDa	0.32	[]	0	19	19	0	12	10
MHC class I antigen (Fragment) OS=Homo sapiens GN=HLA-B PE=3 SV=1	D9UAZ0_HUMAN	34 kDa	0.026	BRAC1 low, Naive high	7	8	9	7	6	0
MHC class I antigen (Fragment) OS=Homo sapiens GN=HLA-B PE=3 SV=1	M5FJF4_HUMAN	21 kDa	0.0084	BRAC1 low, Naive high	10	12	8	0	0	0
MHC class I antigen (Fragment) OS=Homo sapiens GN=HLA-B PE=3 SV=1	G9HZ41_HUMAN	32 kDa	0.0058	BRAC1 low, Naive high	23	25	22	7	6	5
MHC class I antigen (Fragment) OS=Homo sapiens GN=HLA-B PE=3 SV=1	A0A0A7C605_HUMAN (+2)	21 kDa	0.28	[]	0	0	0	5	0	1
MHC class I antigen (Fragment) OS=Homo sapiens GN=HLA-B PE=3 SV=1	A0A0E3DDF4_HUMAN	21 kDa	0.65	[]	10	0	0	4	5	0
MHC class I antigen (Fragment) OS=Homo sapiens GN=HLA-B PE=3 SV=1	L7PEW7_HUMAN	21 kDa	0.0076	BRAC1 low, Naive high	19	23	16	2	0	0
MHC class I antigen (Fragment) OS=Homo sapiens GN=HLA-B PE=3 SV=1	F5A500_HUMAN	32 kDa	0.0025	BRAC1 low, Naive high	22	26	24	7	5	6
MHC class I antigen (Fragment) OS=Homo sapiens GN=HLA-B PE=3 SV=1	D5FZT2_HUMAN	21 kDa	0.013	BRAC1 low, Naive high	13	15	9	0	0	0
HLA-C protein (Fragment) OS=Homo sapiens GN=HLA-C PE=2 SV=1	Q29958_HUMAN	35 kDa	0.0041	BRAC1 low, Naive high	11	14	12	0	0	6
MHC class I antigen (Fragment) OS=Homo sapiens GN=HLA-B PE=3 SV=1	D9I8N4_HUMAN	34 kDa	< 0.00010	BRAC1 low, Naive high	11	13	17	0	0	0
MHC class I antigen (Fragment) OS=Homo sapiens GN=HLA-B PE=3 SV=1	F4NBS9_HUMAN	38 kDa	0.0012	BRAC1 low, Naive high	14	17	18	0	6	0
MHC class I antigen (Fragment) OS=Homo sapiens GN=HLA-B PE=3 SV=1	F4NBY7_HUMAN	38 kDa	0.26	[]	0	15	18	0	12	0
MHC class I antigen (Fragment) OS=Homo sapiens GN=HLA-B PE=3 SV=1	A0A0G2R0X5_HUMAN	21 kDa	0.62	[]	10	0	0	5	4	0
MHC class I antigen (Fragment) OS=Homo sapiens GN=HLA-B PE=3 SV=1	S4TZG0_HUMAN	21 kDa	0.015	BRAC1 low, Naive high	18	20	12	0	0	0
MHC class I antigen (Fragment) OS=Homo sapiens GN=HLA-B PE=3 SV=1	D3HIE9_HUMAN	34 kDa	0.66	[]	0	0	12	0	0	9
MHC class I antigen (Fragment) OS=Homo sapiens GN=HLA-B PE=3 SV=1	F4YTD6_HUMAN	38 kDa	0.12	[]	0	0	0	0	6	6
MHC class I antigen (Fragment) OS=Homo sapiens GN=HLA-B PE=3 SV=1	B2DG08_HUMAN	32 kDa	0.37	[]	0	0	0	13	0	0
MHC class I antigen (Fragment) OS=Homo sapiens GN=HLA-B PE=3 SV=1	J7K012_HUMAN	21 kDa	0.37	[]	0	0	0	0	11	0
MHC class I antigen (Fragment) OS=Homo sapiens GN=HLA-B PE=3 SV=1	A0A077GYB2_HUMAN	21 kDa	0.37	[]	0	0	0	11	0	0
Leucocyte antigen A OS=Homo sapiens GN=HLA-A PE=2 SV=1	Q95J05_HUMAN	41 kDa	0.58	[]	10	10	7	15	16	6
MHC class I antigen (Fragment) OS=Homo sapiens GN=HLA-B PE=3 SV=1	C9WCW0_HUMAN	21 kDa	0.37	[]	0	7	0	0	0	0
MHC class I antigen (Fragment) OS=Homo sapiens GN=HLA-B PE=4 SV=1	A0A0K0KR71_HUMAN (+1)	21 kDa	0.0074	BRAC1 low, Naive high	17	23	15	0	0	0
MHC class I antigen (Fragment) OS=Homo sapiens GN=HLA-B PE=4 SV=1	A0A0K2GUN1_HUMAN	32 kDa	0.056	[]	17	15	17	16	12	0
MHC class I antigen (Fragment) OS=Homo sapiens GN=HLA-C PE=3 SV=1	A2BCX7_HUMAN (+1)	21 kDa	0.37	[]	0	12	8	5	6	0
MHC class I antigen (Fragment) OS=Homo sapiens GN=HLA-C PE=3 SV=1	K7RE26_HUMAN	21 kDa	0.37	[]	10	0	0	0	0	0
MHC class I antigen (Fragment) OS=Homo sapiens GN=HLA-C PE=3 SV=1	A0A0B6VWV2_HUMAN (+1)	41 kDa	0.84	[]	15	0	9	15	13	9
MHC class I antigen (Fragment) OS=Homo sapiens GN=HLA-C PE=3 SV=1	F6IQL0_HUMAN (+1)	39 kDa	0.0062	BRAC1 low, Naive high	11	15	13	0	7	0
MHC class I antigen (Fragment) OS=Homo sapiens GN=HLA-C PE=3 SV=1	E2D5R5_HUMAN	21 kDa	0.2	[]	0	15	11	0	0	6
MHC class I antigen (Fragment) OS=Homo sapiens GN=HLA-C PE=3 SV=1	V5JAP0_HUMAN	21 kDa	0.015	BRAC1 low, Naive high	10	16	10	5	0	0
MHC class I antigen (Fragment) OS=Homo sapiens GN=HLA-C PE=3 SV=1	F6IQJ6_HUMAN	39 kDa	0.0078	BRAC1 low, Naive high	13	20	14	0	0	6
Lymphocyte antigen OS=Homo sapiens GN=HLA-G PE=2 SV=1	Q30182_HUMAN	38 kDa	0.13	[]	0	0	0	3	2	0
MHC class I antigen (Fragment) OS=Homo sapiens GN=HLA-C PE=3 SV=1	E0WMV4_HUMAN	32 kDa	0.25	[]	0	14	10	0	4	5
MHC class I antigen (Fragment) OS=Homo sapiens GN=HLA-C PE=3 SV=1	F6IQI7_HUMAN (+1)	39 kDa	0.006	BRAC1 low, Naive high	15	18	16	6	0	9
MHC class I antigen (Fragment) OS=Homo sapiens GN=HLA-C PE=3 SV=1	A0A0D5CB98_HUMAN	21 kDa	0.27	[]	0	13	7	0	5	0
MHC class I antigen (Fragment) OS=Homo sapiens GN=HLA-C PE=3 SV=1	C0KJY7_HUMAN (+1)	21 kDa	0.0084	BRAC1 low, Naive high	11	16	10	0	0	0
MHC class I antigen (Fragment) OS=Homo sapiens GN=HLA-C PE=3 SV=1	J7F7D0_HUMAN	21 kDa	0.17	[]	0	16	10	0	0	0
MHC class I antigen (Fragment) OS=Homo sapiens GN=HLA-C PE=3 SV=1	A0A0A7C4Z3_HUMAN	21 kDa	0.37	[]	0	9	0	0	0	0
MHC class I antigen (Fragment) OS=Homo sapiens GN=HLA-C PE=3 SV=1	A0A0E3DDD3_HUMAN	21 kDa	0.17	[]	0	16	10	0	0	0
MHC class I antigen (Fragment) OS=Homo sapiens GN=HLA-C PE=3 SV=1	D3JTB9_HUMAN	21 kDa	0.37	[]	0	0	0	0	0	2
MHC class I antigen (Fragment) OS=Homo sapiens GN=HLA-Cw PE=3 SV=1	E7CYP5_HUMAN	21 kDa	0.76	[]	0	15	0	14	13	7
MHC class I antigen (Fragment) OS=Homo sapiens GN=HLA-Cw PE=3 SV=1	A5PHT5_HUMAN (+1)	41 kDa	0.48	[]	9	0	0	0	0	5
MHC Class I antigen (Fragment) OS=Homo sapiens GN=HLA-A PE=3 SV=1	A0A078N209_HUMAN	38 kDa	0.35	[]	16	14	8	17	18	5
MHC class I antigen (Fragment) OS=Homo sapiens GN=HLA-G PE=3 SV=1	A0A0H4LTY3_HUMAN	31 kDa	0.37	[]	0	0	0	0	1	0
MHC class I antigen OS=Homo sapiens GN=HLA-A PE=3 SV=1	D5H3V5_HUMAN	41 kDa	0.17	[]	0	0	0	12	0	7

MHC class I antigen OS=Homo sapiens GN=HLA-A PE=3 SV=1	A0A0D6E067_HUMAN	41 kDa	0.38	[]	21	15	12	21	23	13
MHC class I antigen OS=Homo sapiens GN=HLA-B PE=3 SV=1	A0A0B7MI00_HUMAN	41 kDa	0.037	BRAC1 low, Naive high	14	13	13	0	11	11
MHC class I antigen OS=Homo sapiens GN=HLA-C PE=2 SV=1	Q5UGI4_HUMAN	27 kDa	0.018	BRAC1 low, Naive high	14	15	14	10	0	9
MHC class I antigen OS=Homo sapiens GN=HLA-C PE=3 SV=1	X5M224_HUMAN	41 kDa	0.015	BRAC1 low, Naive high	14	20	15	8	8	7
Putative HLA class I histocompatibility antigen, alpha chain H OS=Homo sapiens GN=HLA-H PE=5 SV=3	HLAH_HUMAN	41 kDa	0.51	[]	12	0	0	0	0	8
cDNA FLJ51097, highly similar to HLA class I histocompatibility antigen, B-7alpha chain OS=Homo sapiens PE=2 SV=1	B4DUK6_HUMAN	27 kDa	0.12	[]	11	0	15	0	0	0
cDNA FLJ52207, highly similar to HLA class I histocompatibility antigen, B-7 alpha chain OS=Homo sapiens PE=2 SV=1	B4DVB3_HUMAN	23 kDa	0.37	[]	0	0	0	2	0	0
MHC class I antigen (Fragment) OS=Homo sapiens GN=HLA PE=3 SV=1	Q0MX32_HUMAN	33 kDa	0.37	[]	0	0	0	0	0	7
MHC class I antigen (Fragment) OS=Homo sapiens GN=HLA-A PE=3 SV=1	F6IQX8_HUMAN	38 kDa	0.29	[]	21	15	10	18	18	11
MMS19 nucleotide excision repair protein homolog OS=Homo sapiens GN=MMS19 PE=1 SV=2	MMS19_HUMAN	113 kDa	0.37	[]	0	0	0	0	0	5
MYO1B variant protein OS=Homo sapiens GN=MYO1B PE=2 SV=1	B0I1S9_HUMAN (+2)	132 kDa	0.011	BRAC1 low, Naive high	7	17	16	4	2	3
Magnesium transporter protein 1 OS=Homo sapiens GN=MAGT1 PE=1 SV=1	MAGT1_HUMAN (+3)	38 kDa	0.87	[]	0	0	1	0	0	2
Major facilitator superfamily domain-containing protein 10 OS=Homo sapiens GN=MFSD10 PE=1 SV=1	MFS10_HUMAN	48 kDa	0.97	[]	1	1	0	1	1	2
Major vault protein OS=Homo sapiens GN=MVP PE=1 SV=4	MVP_HUMAN (+1)	99 kDa	0.0047	BRAC1 low, Naive high	10	10	22	3	1	6
Mal, T-cell differentiation protein 2, isoform CRA_a OS=Homo sapiens GN=MAL2 PE=4 SV=1	A0A024R9E4_HUMAN (+1)	19 kDa	0.004	BRAC1 high, Naive low	0	0	0	7	6	5
Malate dehydrogenase OS=Homo sapiens GN=MDH2 PE=3 SV=1	A0A024R4K3_HUMAN (+4)	36 kDa	0.33	[]	17	11	11	14	17	16
Malate dehydrogenase, cytoplasmic OS=Homo sapiens GN=MDH1 PE=1 SV=4	MDHC_HUMAN (+1)	36 kDa	0.82	[]	0	0	5	2	1	2
Malectin OS=Homo sapiens GN=MLEC PE=1 SV=1	MLEC_HUMAN	32 kDa	0.3	[]	3	2	3	4	6	10
Mannose-1-phosphate guanyltransferase alpha OS=Homo sapiens GN=GMPPA PE=1 SV=1	GMPPA_HUMAN (+1)	46 kDa	0.37	[]	0	0	0	0	0	2
Mannosyl-oligosaccharide glucosidase OS=Homo sapiens GN=MOGS PE=1 SV=5	MOGS_HUMAN	92 kDa	0.46	[]	1	2	6	3	5	16
Matrin-3 OS=Homo sapiens GN=MATR3 PE=1 SV=2	MATR3_HUMAN (+4)	95 kDa	0.55	[]	0	0	2	0	0	1
Matrix metallopeptidase 15 (Membrane-inserted), isoform CRA_a OS=Homo sapiens GN=MMP15 PE=4 SV=1	A0A024R6U8_HUMAN (+2)	76 kDa	0.0058	BRAC1 high, Naive low	0	0	0	6	5	4
Matrix-remodeling-associated protein 7 OS=Homo sapiens GN=MXRA7 PE=1 SV=1	MXRA7_HUMAN	21 kDa	0.37	[]	0	0	2	0	0	0
Medium-chain specific acyl-CoA dehydrogenase, mitochondrial OS=Homo sapiens GN=ACADM PE=1 SV=1	ACADM_HUMAN (+4)	47 kDa	0.69	[]	2	1	2	1	3	8
Melanoma cell adhesion molecule, isoform CRA_a OS=Homo sapiens GN=MCAM PE=4 SV=1	A0A024R3I5_HUMAN (+4)	72 kDa	0.64	[]	2	2	3	2	4	5
Melanoma inhibitory activity protein 3 OS=Homo sapiens GN=MIA3 PE=1 SV=1	MIA3_HUMAN	214 kDa	0.37	[]	0	0	0	0	0	2
Membrane cofactor protein (Fragment) OS=Homo sapiens GN=CD46 PE=1 SV=1	R4GN18_HUMAN	9 kDa	0.04	BRAC1 high, Naive low	0	0	0	2	3	1
Membrane-associated progesterone receptor component 1 OS=Homo sapiens GN=PGRMC1 PE=1 SV=3	PGRC1_HUMAN (+1)	22 kDa	0.043	BRAC1 low, Naive high	6	9	6	4	3	8
Membrane-associated progesterone receptor component 2 OS=Homo sapiens GN=PGRMC2 PE=1 SV=1	PGRC2_HUMAN	24 kDa	0.015	BRAC1 low, Naive high	3	3	3	1	1	3
Metal transporter CNNM3 OS=Homo sapiens GN=CNNM3 PE=1 SV=1	CNNM3_HUMAN	76 kDa	< 0.00010	BRAC1 high, Naive low	0	0	0	4	5	6
Metal transporter CNNM4 OS=Homo sapiens GN=CNNM4 PE=1 SV=3	CNNM4_HUMAN	87 kDa	0.00029	BRAC1 high, Naive low	0	0	0	2	2	2
Metastasis related protein (Fragment) OS=Homo sapiens GN=MB2 PE=2 SV=1	Q9HC85_HUMAN	10 kDa	0.19	[]	0	2	1	0	0	0
Methionine aminopeptidase 1 OS=Homo sapiens GN=METAP1 PE=1 SV=2	MAP11_HUMAN	43 kDa	0.37	[]	0	0	0	0	0	4
Microsomal glutathione S-transferase 1 OS=Homo sapiens GN=MGST1 PE=1 SV=1	MGST1_HUMAN (+2)	18 kDa	0.52	[]	0	2	1	0	2	0
Microsomal glutathione S-transferase 3 OS=Homo sapiens GN=MGST3 PE=1 SV=1	MGST3_HUMAN (+3)	17 kDa	0.092	[]	2	1	1	0	0	1
Microtubule-associated protein 1B OS=Homo sapiens GN=MAP1B PE=1 SV=2	MAP1B_HUMAN (+1)	271 kDa	0.033	BRAC1 low, Naive high	6	8	3	0	0	0
Microtubule-associated protein OS=Homo sapiens GN=MAP4 PE=1 SV=1	E7EVA0_HUMAN (+1)	245 kDa	0.024	BRAC1 low, Naive high	6	5	14	1	2	6
Microtubule-associated protein RP/EB family member 1 OS=Homo sapiens GN=MAPRE1 PE=1 SV=3	MARE1_HUMAN (+2)	30 kDa	0.42	[]	0	0	1	0	1	3
Minor histocompatibility antigen H13 OS=Homo sapiens GN=HM13 PE=1 SV=1	HM13_HUMAN	41 kDa	0.89	[]	0	3	1	0	3	2
Mitochondrial 2-oxoglutarate/malate carrier protein (Fragment) OS=Homo sapiens GN=SLC25A11 PE=1 SV=1	I3L1P8_HUMAN (+2)	32 kDa	0.27	[]	3	1	3	2	2	3
Mitochondrial 10-formyltetrahydrofolate dehydrogenase OS=Homo sapiens GN=ALDH1L2 PE=1 SV=2	AL1L2_HUMAN (+1)	102 kDa	0.017	BRAC1 low, Naive high	1	1	3	0	0	0
Mitochondrial carrier homolog 2 OS=Homo sapiens GN=MTCH2 PE=1 SV=1	MTCH2_HUMAN (+1)	33 kDa	0.48	[]	1	2	6	5	5	8
Mitochondrial import inner membrane translocase subunit TIM44 OS=Homo sapiens GN=TIMM44 PE=1 SV=2	TIM44_HUMAN (+3)	51 kDa	0.42	[]	0	0	1	0	1	3
Mitochondrial import receptor subunit TOM34 OS=Homo sapiens GN=TOMM34 PE=1 SV=2	TOM34_HUMAN	35 kDa	0.011	BRAC1 high, Naive low	0	0	0	2	1	3
Mitochondrial import receptor subunit TOM40 homolog OS=Homo sapiens GN=TOMM40 PE=1 SV=1	TOM40_HUMAN (+1)	38 kDa	0.5	[]	0	0	2	0	2	4
Mitochondrial import receptor subunit TOM70 OS=Homo sapiens GN=TOMM70A PE=1 SV=1	TOM70_HUMAN	67 kDa	0.38	[]	0	5	0	4	7	7
Mitogen-activated protein kinase 14 OS=Homo sapiens GN=MAPK14 PE=1 SV=3	MK14_HUMAN (+3)	41 kDa	0.37	[]	0	0	0	0	0	2
Dual specificity mitogen-activated protein kinase kinase 2 OS=Homo sapiens GN=MAP2K2 PE=1 SV=1	MP2K2_HUMAN	44 kDa	0.37	[]	0	0	0	0	0	3
Mitogen-activated protein kinase kinase 1 OS=Homo sapiens GN=MAP2K1 PE=2 SV=1	A4QPA9_HUMAN (+1)	43 kDa	0.078	[]	0	0	0	2	1	6
Mitotic interactor and substrate of PLK1 OS=Homo sapiens GN=MISP PE=1 SV=1	MISP_HUMAN	75 kDa	0.017	BRAC1 high, Naive low	0	0	0	4	3	2
Ezrin OS=Homo sapiens GN=EZR PE=1 SV=3	E7EQR4_HUMAN (+2)	69 kDa	0.09	[]	5	8	13	44	45	20
Moesin OS=Homo sapiens GN=MSN PE=1 SV=3	MOES_HUMAN (+1)	68 kDa	0.1	[]	9	13	17	36	34	27
Radixin OS=Homo sapiens GN=RDX PE=2 SV=1	B0YJ88_HUMAN (+1)	69 kDa	0.26	[]	0	0	6	6	8	5
Monoacylglycerol lipase ABHD12 OS=Homo sapiens GN=ABHD12 PE=1 SV=2	ABD12_HUMAN	45 kDa	0.18	[]	0	0	0	1	2	11
Monoglyceride lipase OS=Homo sapiens GN=MGLL PE=1 SV=1	A0A0C4DFN3_HUMAN (+1)	34 kDa	0.17	[]	0	0	0	1	0	3
Mothers against decapentaplegic homolog 2 OS=Homo sapiens GN=SMAD2 PE=1 SV=1	SMAD2_HUMAN (+5)	52 kDa	0.37	[]	0	0	0	0	0	3
Multidrug resistance-associated protein 1 OS=Homo sapiens GN=ABCC1 PE=1 SV=3	MRP1_HUMAN	172 kDa	0.019	BRAC1 low, Naive high	3	3	6	3	1	4
Multidrug resistance-associated protein 4 OS=Homo sapiens GN=ABCC4 PE=1 SV=3	MRP4_HUMAN	150 kDa	0.027	BRAC1 high, Naive low	0	0	0	12	15	6
Myelin protein zero-like 1, isoform CRA_b OS=Homo sapiens GN=MPZL1 PE=2 SV=1	A8K5D4_HUMAN (+3)	29 kDa	0.077	[]	0	0	0	4	2	1
Myoferlin OS=Homo sapiens GN=MYOF PE=1 SV=1	MYOF_HUMAN	235 kDa	0.00077	BRAC1 low, Naive high	40	72	89	1	0	5
tr|B3KSL7|B3KSL7_HUMAN cDNA FLJ36571 fis, clone TRACH2012242, highly similar to Myoferlin...	B3KSL7_HUMAN	?	0.37	[]	0	0	5	0	0	0
Myosin light chain kinase, smooth muscle OS=Homo sapiens GN=MYLK PE=1 SV=4	MYLK_HUMAN	211 kDa	0.023	BRAC1 low, Naive high	5	14	9	0	0	0

Myosin light polypeptide 6 OS=Homo sapiens GN=MYL6 PE=1 SV=1	F8W1R7_HUMAN (+4)	16 kDa	0.29	[]	8	4	5	6	7	3
Myosin regulatory light chain 12A OS=Homo sapiens GN=MYL12A PE=1 SV=1	J3QRS3_HUMAN (+3)	20 kDa	0.014	BRAC1 low, Naive high	2	2	3	2	1	0
Myosin regulatory light polypeptide 9 OS=Homo sapiens GN=MYL9 PE=1 SV=4	MYL9_HUMAN (+2)	20 kDa	0.015	BRAC1 low, Naive high	1	1	2	1	0	0
Myosin, heavy polypeptide 9, non-muscle, isoform CRA_a OS=Homo sapiens GN=MYH9 PE=4 SV=1	A0A024R1N1_HUMAN (+1)	227 kDa	< 0.00010	BRAC1 low, Naive high	91	119	148	66	61	89
Myosin-10 OS=Homo sapiens GN=MYH10 PE=1 SV=3	MYH10_HUMAN	229 kDa	0.028	BRAC1 low, Naive high	11	18	40	5	5	12
Myosin-14 (Fragment) OS=Homo sapiens GN=MYH14 PE=1 SV=6	M0QY43_HUMAN	114 kDa	0.00038	BRAC1 high, Naive low	0	0	0	26	27	26
Myosin-14 OS=Homo sapiens GN=MYH14 PE=1 SV=2	MYH14_HUMAN	228 kDa	0.0029	BRAC1 high, Naive low	8	9	10	63	59	56
Myristoylated alanine-rich C-kinase substrate OS=Homo sapiens GN=MARCKS PE=1 SV=1	A0A087WZH7_HUMAN (+1)	32 kDa	0.016	BRAC1 low, Naive high	13	19	20	14	16	7
N(G),N(G)-dimethylarginine dimethylaminohydrolase 2 OS=Homo sapiens GN=DDAH2 PE=1 SV=1	DDAH2_HUMAN (+1)	30 kDa	0.76	[]	1	1	0	2	0	1
N-acetyl-D-glucosamine kinase OS=Homo sapiens GN=NAGK PE=1 SV=4	NAGK_HUMAN (+4)	37 kDa	0.37	[]	0	0	0	0	0	3
N-acetylgalactosaminyltransferase 7 OS=Homo sapiens GN=GALNT7 PE=1 SV=1	GALT7_HUMAN	75 kDa	0.0027	BRAC1 high, Naive low	0	0	0	1	1	2
N-alpha-acetyltransferase 15, NatA auxiliary subunit OS=Homo sapiens GN=NAA15 PE=1 SV=1	NAA15_HUMAN (+4)	101 kDa	0.37	[]	0	0	0	0	0	2
N-ethylmaleimide-sensitive factor attachment protein, alpha, isoform CRA_c OS=Homo sapiens GN=NAPA PE=4 SV=1	A0A024R0R9_HUMAN (+1)	33 kDa	0.14	[]	0	0	3	4	3	12
N-ethylmaleimide-sensitive factor attachment protein, gamma, isoform CRA_b OS=Homo sapiens GN=NAPG PE=2 SV=1	Q6FHY4_HUMAN (+1)	35 kDa	0.0027	BRAC1 high, Naive low	0	0	0	1	1	2
N-myc downstream regulated gene 1, isoform CRA_b OS=Homo sapiens GN=NDRG1 PE=4 SV=1	A0A024R9I3_HUMAN (+11)	39 kDa	0.96	[]	1	1	2	1	1	5
N-terminal kinase-like protein OS=Homo sapiens GN=SCYL1 PE=1 SV=1	NTKL_HUMAN (+3)	90 kDa	0.37	[]	0	0	0	0	0	2
NAD(P) transhydrogenase, mitochondrial OS=Homo sapiens GN=NNT PE=1 SV=3	NNTM_HUMAN (+2)	114 kDa	0.17	[]	0	1	3	0	0	0
NAD(P)H dehydrogenase [quinone] 1 OS=Homo sapiens GN=NQO1 PE=1 SV=1	NQO1_HUMAN (+1)	31 kDa	0.062	[]	0	0	3	4	5	11
NADH dehydrogenase (Ubiquinone) 1 alpha subcomplex, 10, 42kDa, isoform CRA_b OS=Homo sapiens GN=NDUFA10 PE=4 SV=1	A0A024R4B3_HUMAN (+5)	41 kDa	0.45	[]	1	2	2	2	3	1
NADH dehydrogenase [ubiquinone] flavoprotein 1, mitochondrial OS=Homo sapiens GN=NDUFV1 PE=1 SV=4	NDUV1_HUMAN (+6)	51 kDa	0.82	[]	0	0	2	0	1	1
NADH dehydrogenase [ubiquinone] iron-sulfur protein 2, mitochondrial OS=Homo sapiens GN=NDUFS2 PE=1 SV=2	NDUS2_HUMAN (+1)	53 kDa	0.82	[]	0	1	2	0	1	3
NADH dehydrogenase [ubiquinone] iron-sulfur protein 3, mitochondrial OS=Homo sapiens GN=NDUFS3 PE=1 SV=1	NDUS3_HUMAN (+1)	30 kDa	0.78	[]	0	1	3	1	3	3
NADH-cytochrome b5 reductase OS=Homo sapiens GN=CYB5R3 PE=3 SV=1	A0A024R4X0_HUMAN (+1)	33 kDa	0.00085	BRAC1 low, Naive high	9	11	14	1	4	8
NADH-ubiquinone oxidoreductase 75 kDa subunit, mitochondrial OS=Homo sapiens GN=NDUFS1 PE=1 SV=1	B4DJ81_HUMAN (+2)	67 kDa	0.33	[]	1	3	7	3	3	4
NADPH--cytochrome P450 reductase OS=Homo sapiens PE=2 SV=1	A8K3B4_HUMAN (+4)	77 kDa	0.0076	BRAC1 high, Naive low	1	0	1	4	6	7
NADPH:adrenodoxin oxidoreductase, mitochondrial OS=Homo sapiens GN=FDXR PE=1 SV=3	ADRO_HUMAN (+9)	54 kDa	0.2	[]	1	0	2	0	0	1
Na(+)/H(+) exchange regulatory cofactor NHE-RF1 OS=Homo sapiens GN=SLC9A3R1 PE=1 SV=4	NHRF1_HUMAN	39 kDa	0.012	BRAC1 high, Naive low	0	0	0	7	7	4
Na(+)/H(+) exchange regulatory cofactor NHE-RF2 (Fragment) OS=Homo sapiens GN=SLC9A3R2 PE=1 SV=1	H3BMF5_HUMAN	17 kDa	0.37	[]	0	0	0	0	1	0
Na(+)/H(+) exchange regulatory cofactor NHE-RF2 OS=Homo sapiens GN=SLC9A3R2 PE=1 SV=2	NHRF2_HUMAN (+1)	37 kDa	0.00018	BRAC1 high, Naive low	0	0	0	6	7	7
Nck-associated protein 1 OS=Homo sapiens GN=NCKAP1 PE=1 SV=1	NCKP1_HUMAN	129 kDa	0.013	BRAC1 high, Naive low	1	0	2	5	5	7
Nectin-2 OS=Homo sapiens GN=PVRL2 PE=1 SV=1	PVRL2_HUMAN	58 kDa	0.58	[]	1	1	2	3	4	1
Polio virus related protein 2, alpha isoform (Fragment) OS=Homo sapiens PE=4 SV=1	Q9UEI6_HUMAN	48 kDa	0.8	[]	1	1	2	4	3	0
Neprilysin OS=Homo sapiens GN=MME PE=1 SV=2	NEP_HUMAN (+1)	86 kDa	0.049	BRAC1 low, Naive high	3	3	12	0	0	0
Nestin OS=Homo sapiens GN=NES PE=1 SV=2	NEST_HUMAN (+3)	177 kDa	0.37	[]	0	0	2	0	0	0
Neural cell adhesion molecule L1 OS=Homo sapiens GN=L1CAM PE=1 SV=2	L1CAM_HUMAN (+2)	140 kDa	< 0.00010	BRAC1 low, Naive high	4	5	7	0	0	0
Neurobeachin-like protein 2 OS=Homo sapiens GN=NBEAL2 PE=1 SV=2	NBEL2_HUMAN	303 kDa	0.37	[]	0	0	0	0	0	5
Neuroblast differentiation-associated protein AHNAK OS=Homo sapiens GN=AHNAK PE=1 SV=2	AHNK_HUMAN	629 kDa	0.47	[]	82	134	80	174	129	40
Neuroblastoma RAS viral (V-ras) oncogene homolog OS=Homo sapiens GN=NRAS PE=2 SV=1	Q5U091_HUMAN (+1)	21 kDa	0.58	[]	0	2	0	1	2	3
V-Ki-ras2 Kirsten rat sarcoma viral oncogene homolog, isoform CRA_b OS=Homo sapiens GN=KRAS PE=1 SV=1	A0A024RAV5_HUMAN	21 kDa	0.29	[]	0	1	1	3	2	1
Neurofascin OS=Homo sapiens GN=NFASC PE=1 SV=4	NFASC_HUMAN (+2)	150 kDa	0.37	[]	0	2	0	0	0	0
Neurofibromin OS=Homo sapiens GN=NF1 PE=1 SV=2	NF1_HUMAN	319 kDa	0.005	BRAC1 high, Naive low	0	0	0	2	3	5
Neuropilin-1 OS=Homo sapiens GN=NRP1 PE=1 SV=3	NRP1_HUMAN (+4)	103 kDa	0.0044	BRAC1 low, Naive high	3	2	4	0	0	0
Neuroplastin OS=Homo sapiens GN=NPTN PE=1 SV=2	NPTN_HUMAN (+1)	44 kDa	0.0014	BRAC1 low, Naive high	4	4	6	3	2	2
Neutral cholesterol ester hydrolase 1 OS=Homo sapiens GN=NCEH1 PE=1 SV=1	A0A0A0MTJ9_HUMAN (+1)	50 kDa	0.093	[]	4	2	5	1	3	4
Neutrophil gelatinase-associated lipocalin OS=Homo sapiens GN=NGAL PE=2 SV=1	B2ZDQ1_HUMAN (+2)	23 kDa	< 0.00010	BRAC1 high, Naive low	0	0	0	2	2	3
Nexilin OS=Homo sapiens GN=NEXN PE=1 SV=1	NEXN_HUMAN (+2)	81 kDa	0.37	[]	0	0	2	0	0	0
Nicalin OS=Homo sapiens PE=2 SV=1	B2RA56_HUMAN (+1)	62 kDa	0.52	[]	0	1	5	2	4	8
Nicastrin OS=Homo sapiens GN=NCSTN PE=1 SV=2	NICA_HUMAN	78 kDa	0.92	[]	1	1	3	1	2	6
Nicotinate phosphoribosyltransferase OS=Homo sapiens GN=NAPRT PE=1 SV=2	PNCB_HUMAN	58 kDa	0.3	[]	0	0	0	1	0	11
PP3856 OS=Homo sapiens PE=2 SV=1	Q8WY82_HUMAN	15 kDa	0.37	[]	0	0	0	0	0	2
Nidogen-2 OS=Homo sapiens GN=NID2 PE=1 SV=3	NID2_HUMAN (+3)	151 kDa	0.0021	BRAC1 low, Naive high	1	2	2	0	0	0
Niemann-Pick C1 protein OS=Homo sapiens GN=NPC1 PE=1 SV=2	NPC1_HUMAN (+2)	142 kDa	0.5	[]	0	2	0	0	0	1
Nodal modulator 1 OS=Homo sapiens GN=NOMO1 PE=1 SV=1	A0A087X117_HUMAN (+2)	139 kDa	0.87	[]	0	4	7	2	4	14
Nodal modulator 3 OS=Homo sapiens GN=NOMO3 PE=1 SV=1	J3KN36_HUMAN (+2)	139 kDa	1	[]	0	3	7	1	3	12
Non-POU domain-containing octamer-binding protein OS=Homo sapiens GN=NONO PE=1 SV=4	NONO_HUMAN (+1)	54 kDa	0.23	[]	0	0	0	0	1	5
Non-specific serine/threonine protein kinase OS=Homo sapiens GN=CDC42BPA PE=1 SV=1	A0A0A0MRJ0_HUMAN (+3)	194 kDa	0.091	[]	0	0	0	3	1	1
Non-specific serine/threonine protein kinase OS=Homo sapiens GN=PAK4 PE=4 SV=1	A0A024R0J1_HUMAN (+2)	64 kDa	0.012	BRAC1 high, Naive low	0	0	0	3	5	3
Non-specific serine/threonine protein kinase OS=Homo sapiens GN=PAK1 PE=4 SV=1	A0A024R5P0_HUMAN (+5)	61 kDa	0.028	BRAC1 high, Naive low	0	0	0	1	2	1
Non-specific serine/threonine protein kinase OS=Homo sapiens PE=2 SV=1	A8K5M4_HUMAN (+1)	58 kDa	0.0058	BRAC1 high, Naive low	0	0	0	1	2	2
Nuclear migration protein nudC OS=Homo sapiens GN=NUDC PE=1 SV=1	NUDC_HUMAN	38 kDa	0.12	[]	0	0	0	2	2	0
Nuclear pore complex protein Nup205 OS=Homo sapiens GN=NUP205 PE=1 SV=3	NU205_HUMAN	228 kDa	0.13	[]	0	0	0	0	1	2
Cold shock domain protein A, isoform CRA_a OS=Homo sapiens GN=CSDA PE=4 SV=1	A0A024RAQ1_HUMAN (+2)	40 kDa	0.99	[]	0	2	4	2	3	4

Nuclease-sensitive element-binding protein 1 OS=Homo sapiens GN=YBX1 PE=1 SV=3	YBOX1_HUMAN	36 kDa	0.29	[]	4	6	8	4	8	12
Nucleolin, isoform CRA_b OS=Homo sapiens GN=NCL PE=4 SV=1	A0A024R4A0_HUMAN (+4)	77 kDa	0.11	[]	0	0	4	4	5	10
Nucleoside diphosphate kinase A OS=Homo sapiens GN=NME1 PE=1 SV=1	NDKA_HUMAN	17 kDa	0.00029	BRAC1 high, Naive low	0	0	0	3	3	3
Nucleoside diphosphate kinase B OS=Homo sapiens GN=NME2 PE=1 SV=1	NDKB_HUMAN (+2)	17 kDa	0.022	BRAC1 high, Naive low	0	0	1	2	2	4
Nucleosome assembly protein 1-like 1, isoform CRA_a OS=Homo sapiens GN=NAP1L1 PE=3 SV=1	A0A024RBB7_HUMAN (+12)	45 kDa	0.22	[]	1	0	1	1	3	4
Nucleosome assembly protein 1-like 4 OS=Homo sapiens GN=NAP1L4 PE=1 SV=1	NP1L4_HUMAN (+4)	43 kDa	0.5	[]	0	0	1	0	1	2
OCIA domain-containing protein 1 OS=Homo sapiens GN=OCIAD1 PE=1 SV=1	OCAD1_HUMAN (+1)	28 kDa	0.37	[]	0	0	2	0	0	0
Occludin OS=Homo sapiens GN=OCLN PE=1 SV=1	A0A0G2JMZ8_HUMAN (+1)	59 kDa	0.04	BRAC1 high, Naive low	0	0	0	4	6	2
Omega-amidase NIT2 OS=Homo sapiens GN=NIT2 PE=1 SV=1	NIT2_HUMAN (+1)	31 kDa	0.028	BRAC1 high, Naive low	0	0	0	1	2	1
Opioid growth factor receptor OS=Homo sapiens GN=OGFR PE=1 SV=3	OGFR_HUMAN	73 kDa	0.37	[]	0	0	0	0	0	4
Ornithine aminotransferase, mitochondrial OS=Homo sapiens GN=OAT PE=1 SV=1	OAT_HUMAN	49 kDa	0.082	[]	2	4	3	0	3	3
Overexpressed in colon carcinoma 1 protein OS=Homo sapiens GN=OCC1 PE=1 SV=2	OCC1_HUMAN (+1)	6 kDa	0.37	[]	0	0	2	0	0	0
Oxysterol-binding protein 1 OS=Homo sapiens GN=OSBP PE=1 SV=1	OSBP1_HUMAN	89 kDa	0.25	[]	0	0	0	1	0	6
Oxysterol-binding protein OS=Homo sapiens GN=OSBPL8 PE=3 SV=1	A0A024RBB8_HUMAN (+2)	101 kDa	0.088	[]	0	0	1	2	1	2
PDZ and LIM domain 5, isoform CRA_c OS=Homo sapiens GN=PDLIM5 PE=4 SV=1	A0A024RDE8_HUMAN (+1)	64 kDa	0.31	[]	1	1	1	2	4	2
PDZ and LIM domain protein 1 OS=Homo sapiens GN=PDLIM1 PE=1 SV=4	PDLI1_HUMAN (+1)	36 kDa	0.95	[]	3	3	2	8	4	1
PDZ and LIM domain protein 7 OS=Homo sapiens GN=PDLIM7 PE=1 SV=1	PDLI7_HUMAN	50 kDa	0.054	[]	1	4	8	0	0	1
PDZ domain-containing protein GIPC2 OS=Homo sapiens GN=GIPC2 PE=1 SV=1	GIPC2_HUMAN	34 kDa	0.37	[]	0	0	0	0	0	3
PTGFRN protein (Fragment) OS=Homo sapiens GN=PTGFRN PE=2 SV=1	Q1WWL2_HUMAN (+1)	83 kDa	0.00029	BRAC1 high, Naive low	0	0	0	3	3	3
Prostaglandin F2 receptor negative regulator OS=Homo sapiens GN=PTGFRN PE=1 SV=2	FPRP_HUMAN	99 kDa	0.00029	BRAC1 high, Naive low	0	0	0	2	2	2
Pachytene checkpoint protein 2 homolog OS=Homo sapiens GN=TRIP13 PE=1 SV=2	PCH2_HUMAN	49 kDa	0.027	BRAC1 high, Naive low	0	0	0	1	2	4
Paladin OS=Homo sapiens GN=PALD1 PE=1 SV=3	PALD_HUMAN (+1)	97 kDa	0.37	[]	0	0	0	0	0	2
Palladin OS=Homo sapiens GN=PALLD PE=1 SV=3	PALLD_HUMAN (+4)	151 kDa	0.13	[]	0	1	2	0	0	0
Palmitoyltransferase OS=Homo sapiens GN=ZDHHC5 PE=3 SV=1	A0A024R546_HUMAN (+1)	78 kDa	0.12	[]	0	0	0	3	4	0
Palmitoyltransferase OS=Homo sapiens GN=ZDHHC20 PE=1 SV=1	B4DRN8_HUMAN (+1)	34 kDa	0.031	BRAC1 high, Naive low	0	0	0	2	1	1
Paraoxonase 2, isoform CRA_a OS=Homo sapiens GN=PON2 PE=1 SV=1	A0A0J9YXF2_HUMAN (+2)	42 kDa	0.67	[]	1	2	2	1	0	6
Paraspeckle component 1 OS=Homo sapiens GN=PSPC1 PE=1 SV=1	PSPC1_HUMAN (+2)	59 kDa	0.37	[]	0	0	0	0	0	2
Pentatricopeptide repeat domain-containing protein 3, mitochondrial OS=Homo sapiens GN=PTCD3 PE=1 SV=3	PTCD3_HUMAN	79 kDa	0.37	[]	0	0	0	0	0	3
Peptidyl-prolyl cis-trans isomerase B OS=Homo sapiens GN=PPIB PE=1 SV=2	PPIB_HUMAN (+1)	24 kDa	0.84	[]	5	5	4	8	9	9
Peptidyl-prolyl cis-trans isomerase FKBP4 OS=Homo sapiens GN=FKBP4 PE=1 SV=3	FKBP4_HUMAN	52 kDa	0.07	[]	0	0	0	1	1	4
Peptidyl-prolyl cis-trans isomerase FKBP9 OS=Homo sapiens GN=FKBP9 PE=1 SV=2	FKBP9_HUMAN (+2)	63 kDa	0.029	BRAC1 low, Naive high	1	3	5	0	0	1
Peptidyl-prolyl cis-trans isomerase FKBP10 OS=Homo sapiens GN=FKBP10 PE=1 SV=1	FKB10_HUMAN (+2)	64 kDa	0.2	[]	1	1	7	0	0	2
Peptidyl-prolyl cis-trans isomerase OS=Homo sapiens PE=2 SV=1	A8K486_HUMAN (+2)	18 kDa	0.088	[]	1	4	4	21	18	8
Perilipin-3 OS=Homo sapiens GN=PLIN3 PE=1 SV=3	PLIN3_HUMAN	47 kDa	0.0059	BRAC1 low, Naive high	2	2	5	0	0	0
Peripheral plasma membrane protein CASK OS=Homo sapiens GN=CASK PE=1 SV=3	CSKP_HUMAN	105 kDa	0.12	[]	0	1	0	7	10	1
Periplakin OS=Homo sapiens GN=PPL PE=1 SV=1	K7EKI8_HUMAN (+2)	204 kDa	0.13	[]	0	0	0	0	1	2
Peroxiredoxin-1 OS=Homo sapiens GN=PRDX1 PE=1 SV=1	PRDX1_HUMAN (+1)	22 kDa	0.77	[]	6	4	3	10	6	4
Peroxiredoxin-4 OS=Homo sapiens GN=PRDX4 PE=1 SV=1	PRDX4_HUMAN (+1)	31 kDa	0.2	[]	7	8	4	8	6	3
Peroxiredoxin-5, mitochondrial OS=Homo sapiens GN=PRDX5 PE=1 SV=4	PRDX5_HUMAN (+1)	22 kDa	0.071	[]	0	0	1	4	2	2
Peroxiredoxin-6 OS=Homo sapiens GN=PRDX6 PE=1 SV=3	PRDX6_HUMAN (+1)	25 kDa	0.28	[]	4	1	6	0	2	7
Peroxisomal acyl-coenzyme A oxidase 1 OS=Homo sapiens GN=ACOX1 PE=1 SV=3	ACOX1_HUMAN (+3)	74 kDa	0.29	[]	0	0	1	0	2	10
Peroxisomal membrane protein PEX14 OS=Homo sapiens GN=PEX14 PE=1 SV=1	PEX14_HUMAN (+1)	41 kDa	0.37	[]	0	0	0	0	0	2
Peroxisomal multifunctional enzyme type 2 OS=Homo sapiens GN=HSD17B4 PE=1 SV=3	DHB4_HUMAN	80 kDa	0.99	[]	10	16	15	19	25	26
Peroxisome proliferator activated receptor interacting complex protein OS=Homo sapiens GN=PRIC295 PE=2 SV=1	E1NZA1_HUMAN	293 kDa	0.043	BRAC1 high, Naive low	0	0	4	16	16	49
Phenylalanine--tRNA ligase alpha subunit (Fragment) OS=Homo sapiens GN=FARSA PE=1 SV=6	K7EK06_HUMAN (+3)	18 kDa	0.21	[]	2	1	2	2	1	2
Phosphatidylinositol 3,4,5-trisphosphate 5-phosphatase 1 OS=Homo sapiens GN=INPP5D PE=1 SV=2	SHIP1_HUMAN	133 kDa	0.37	[]	0	0	0	0	0	2
Phosphatidylinositol binding clathrin assembly protein, isoform CRA_c OS=Homo sapiens GN=PICALM PE=4 SV=1	A0A024R5L7_HUMAN (+4)	66 kDa	0.16	[]	3	3	4	7	9	7
Phosphatidylinositol-binding clathrin assembly protein (Fragment) OS=Homo sapiens GN=PICALM PE=1 SV=1	H0YEF7_HUMAN (+1)	31 kDa	0.27	[]	3	0	2	6	8	4
Phosphatidylinositol-4-phosphate 3-kinase, catalytic subunit type 2 alpha OS=Homo sapiens GN=PIK3C2A PE=4 SV=1	L7RRS0_HUMAN (+1)	191 kDa	0.13	[]	0	0	0	1	0	2
Phosphatidylserine synthase 1 OS=Homo sapiens GN=PTDSS1 PE=1 SV=1	PTSS1_HUMAN (+2)	56 kDa	0.47	[]	0	1	0	0	2	2
Phosphoenolpyruvate carboxykinase [GTP], mitochondrial OS=Homo sapiens GN=PCK2 PE=1 SV=3	PCKGM_HUMAN	71 kDa	0.2	[]	0	1	2	4	2	3
Phosphoglycerate kinase 1 OS=Homo sapiens GN=PGK1 PE=1 SV=3	PGK1_HUMAN (+1)	45 kDa	0.55	[]	7	8	13	20	12	20
Phosphoglycerate mutase 1 OS=Homo sapiens GN=PGAM1 PE=1 SV=2	PGAM1_HUMAN (+3)	29 kDa	0.68	[]	2	2	5	5	3	5
Phospholipase A-2-activating protein OS=Homo sapiens GN=PLAA PE=1 SV=2	PLAP_HUMAN (+2)	87 kDa	0.37	[]	0	0	0	0	0	2
Phospholipase D3 OS=Homo sapiens GN=PLD3 PE=1 SV=1	PLD3_HUMAN (+1)	55 kDa	0.14	[]	1	2	0	0	0	0
Phospholipid hydroperoxide glutathione peroxidase, mitochondrial OS=Homo sapiens GN=GPX4 PE=1 SV=3	GPX4_HUMAN (+9)	22 kDa	0.37	[]	0	0	0	0	0	2
Phospholipid scramblase 1 OS=Homo sapiens GN=PLSCR1 PE=1 SV=1	C9J7K9_HUMAN (+1)	34 kDa	0.23	[]	1	0	0	6	4	0
Phospholipid-transporting ATPase IC OS=Homo sapiens GN=ATP8B1 PE=1 SV=3	AT8B1_HUMAN	144 kDa	0.071	[]	0	0	0	8	3	3
Phosphoribosylaminoimidazole carboxylase, phosphoribosylaminoimidazole succinocarboxamide synthetase, isoform CRA_c OS=Hom	A0A024RD93_HUMAN (+2)	47 kDa	0.063	[]	0	1	1	2	7	9
Phosphoserine aminotransferase OS=Homo sapiens GN=PSAT1 PE=3 SV=1	A0A024R222_HUMAN (+3)	40 kDa	0.6	[]	0	0	1	0	0	3
Piezo-type mechanosensitive ion channel component 1 OS=Homo sapiens GN=PIEZO1 PE=1 SV=4	PIEZ1_HUMAN (+1)	287 kDa	0.89	[]	0	1	0	0	2	0
Plakophilin-2 OS=Homo sapiens GN=PKP2 PE=1 SV=1	B8QGS9_HUMAN (+1)	91 kDa	0.05	[]	0	0	0	4	1	3

Plakophilin-3 OS=Homo sapiens GN=PKP3 PE=1 SV=1	PKP3_HUMAN	87 kDa	0.00056	BRAC1 high, Naive low	0	0	0	9	13	15
Plakophilin-4 OS=Homo sapiens GN=PKP4 PE=1 SV=1	A0A0D9SF60_HUMAN (+2)	134 kDa	0.0086	BRAC1 high, Naive low	0	0	0	10	6	7
Plasma membrane citrate carrier OS=Homo sapiens GN=SLC25A1 PE=2 SV=1	D9HTE9_HUMAN (+2)	35 kDa	0.85	[]	0	2	0	0	1	2
Plasminogen activator inhibitor 1 RNA-binding protein OS=Homo sapiens GN=SERBP1 PE=1 SV=2	PAIRB_HUMAN (+1)	45 kDa	0.96	[]	1	1	6	0	1	12
Plastin-1 OS=Homo sapiens GN=PLS1 PE=1 SV=2	PLSI_HUMAN	70 kDa	0.0048	BRAC1 high, Naive low	1	1	1	12	13	10
Plastin-3 OS=Homo sapiens GN=PLS3 PE=1 SV=1	A0A0A0MSQ0_HUMAN (+3)	69 kDa	0.14	[]	2	4	3	3	3	4
Platelet-activating factor acetylhydrolase IB subunit alpha OS=Homo sapiens GN=PAFAH1B1 PE=1 SV=2	LIS1_HUMAN	47 kDa	0.59	[]	0	1	2	1	1	1
Platelet-activating factor acetylhydrolase IB subunit beta OS=Homo sapiens GN=PAFAH1B2 PE=1 SV=1	PA1B2_HUMAN (+1)	26 kDa	0.028	BRAC1 high, Naive low	0	0	1	2	2	2
Platelet-activating factor acetylhydrolase, isoform Ib, gamma subunit 29kDa, isoform CRA_a OS=Homo sapiens GN=PAFAH1B3 PE=4 S	A0A024R0L6_HUMAN (+1)	26 kDa	0.1	[]	0	0	0	2	1	7
Platelet-derived growth factor receptor beta OS=Homo sapiens GN=PDGFRB PE=1 SV=1	PGFRB_HUMAN	124 kDa	0.007	BRAC1 low, Naive high	3	4	9	0	0	0
Pleckstrin homology domain containing, family A member 5, isoform CRA_a OS=Homo sapiens GN=PLEKHA5 PE=4 SV=1	A0A024RAY7_HUMAN (+2)	134 kDa	0.033	BRAC1 high, Naive low	0	0	0	3	5	2
Pleckstrin homology domain-containing family A member 6 OS=Homo sapiens GN=PLEKHA6 PE=1 SV=4	PKHA6_HUMAN (+1)	117 kDa	0.088	[]	0	0	0	10	9	1
Pleckstrin homology-like domain family A member 2 OS=Homo sapiens GN=PHLDA2 PE=1 SV=2	PHLA2_HUMAN	17 kDa	0.37	[]	0	0	0	2	0	0
Pleckstrin homology-like domain family B member 2 OS=Homo sapiens GN=PHLDB2 PE=1 SV=2	PHLB2_HUMAN (+5)	142 kDa	0.37	[]	0	0	2	0	0	0
Plectin OS=Homo sapiens GN=PLEC PE=1 SV=3	PLEC_HUMAN	532 kDa	0.05	BRAC1 low, Naive high	34	65	40	18	24	45
Plexin-A1 OS=Homo sapiens GN=PLXNA1 PE=1 SV=3	PLXA1_HUMAN	211 kDa	0.001	BRAC1 high, Naive low	0	0	0	2	3	3
Plexin-B1 OS=Homo sapiens GN=PLXNB1 PE=1 SV=3	PLXB1_HUMAN	232 kDa	0.16	[]	0	0	0	2	4	0
Plexin-B2 OS=Homo sapiens GN=PLXNB2 PE=1 SV=3	PLXB2_HUMAN	205 kDa	0.0084	BRAC1 high, Naive low	2	4	10	27	21	27
Podocalyxin OS=Homo sapiens GN=PODXL PE=1 SV=2	PODXL_HUMAN (+2)	59 kDa	0.12	[]	0	0	0	0	2	2
Poliovirus receptor OS=Homo sapiens GN=PVR PE=1 SV=1	A0A0C4DG49_HUMAN (+3)	45 kDa	0.23	[]	0	1	1	1	2	4
Poly [ADP-ribose] polymerase 1 OS=Homo sapiens GN=PARP1 PE=1 SV=4	PARP1_HUMAN (+3)	113 kDa	0.17	[]	0	0	0	0	1	2
Poly [ADP-ribose] polymerase 14 OS=Homo sapiens GN=PARP14 PE=1 SV=3	PAR14_HUMAN	203 kDa	0.095	[]	0	0	1	5	5	1
Poly(rC)-binding protein 1 OS=Homo sapiens GN=PCBP1 PE=1 SV=2	PCBP1_HUMAN (+1)	37 kDa	0.96	[]	1	2	5	0	4	9
Poly(rC)-binding protein 2 (Fragment) OS=Homo sapiens GN=PCBP2 PE=1 SV=2	F8VRH0_HUMAN	32 kDa	0.13	[]	0	0	0	0	1	2
Poly(rC)-binding protein 2 OS=Homo sapiens GN=PCBP2 PE=1 SV=1	PCBP2_HUMAN	39 kDa	0.75	[]	1	2	3	1	2	6
Polyadenylate-binding protein OS=Homo sapiens GN=PABPC1 PE=3 SV=1	A0A024R9C1_HUMAN (+3)	71 kDa	0.74	[]	3	3	6	4	5	10
Polyadenylate-binding protein OS=Homo sapiens GN=PABPC4 PE=1 SV=1	B1ANR0_HUMAN (+2)	68 kDa	0.81	[]	0	2	4	2	2	7
Polymerase I and transcript release factor OS=Homo sapiens GN=PTRF PE=1 SV=1	PTRF_HUMAN	43 kDa	0.0041	BRAC1 low, Naive high	13	9	21	0	0	0
Polypeptide N-acetylgalactosaminyltransferase 2 OS=Homo sapiens GN=GALNT2 PE=1 SV=1	GALT2_HUMAN (+2)	65 kDa	0.39	[]	0	3	1	0	1	1
Polypeptide N-acetylgalactosaminyltransferase 3 OS=Homo sapiens GN=GALNT3 PE=1 SV=2	GALT3_HUMAN (+1)	73 kDa	0.25	[]	0	0	0	0	1	6
Polypyrimidine tract binding protein 1, isoform CRA_b OS=Homo sapiens GN=PTBP1 PE=1 SV=4	A6NLN1_HUMAN (+1)	57 kDa	0.85	[]	1	4	7	2	2	14
Polypyrimidine tract-binding protein 3 OS=Homo sapiens GN=PTBP3 PE=1 SV=2	PTBP3_HUMAN (+1)	60 kDa	0.37	[]	0	0	0	0	0	3
Pre-B-cell colony enhancing factor 1, isoform CRA_a OS=Homo sapiens GN=PBEF1 PE=4 SV=1	A0A024R718_HUMAN (+1)	56 kDa	0.31	[]	0	0	0	1	0	12
Pre-mRNA-processing-splicing factor 8 OS=Homo sapiens GN=PRPF8 PE=1 SV=2	PRP8_HUMAN	274 kDa	0.37	[]	0	0	0	0	0	4
Pre-mRNA-splicing factor ATP-dependent RNA helicase DHX15 OS=Homo sapiens GN=DHX15 PE=1 SV=2	DHX15_HUMAN	91 kDa	0.37	[]	0	0	0	0	0	4
Lamin A/C OS=Homo sapiens GN=LMNA PE=3 SV=1	W8QEH3_HUMAN	65 kDa	0.016	BRAC1 low, Naive high	12	24	38	4	2	18
Prelamin-A/C OS=Homo sapiens GN=LMNA PE=1 SV=1	LMNA_HUMAN	74 kDa	0.0061	BRAC1 low, Naive high	16	27	41	5	2	20
Presenilin OS=Homo sapiens GN=PSEN1 PE=3 SV=1	A0A024R6A3_HUMAN (+3)	53 kDa	0.16	[]	0	0	0	1	2	0
Probable ATP-dependent RNA helicase DDX5 OS=Homo sapiens GN=DDX5 PE=1 SV=1	DDX5_HUMAN (+3)	69 kDa	0.54	[]	0	0	2	0	1	6
Probable ATP-dependent RNA helicase DDX17 OS=Homo sapiens GN=DDX17 PE=1 SV=2	DDX17_HUMAN (+2)	80 kDa	0.76	[]	1	1	3	0	3	4
Procollagen galactosyltransferase 1 OS=Homo sapiens GN=COLGALT1 PE=1 SV=1	GT251_HUMAN	72 kDa	0.15	[]	1	3	1	0	0	2
Procollagen-lysine,2-oxoglutarate 5-dioxygenase 1 OS=Homo sapiens GN=PLOD1 PE=1 SV=2	PLOD1_HUMAN (+2)	84 kDa	0.0041	BRAC1 low, Naive high	5	5	5	0	0	2
Procollagen-lysine,2-oxoglutarate 5-dioxygenase 2 OS=Homo sapiens GN=PLOD2 PE=1 SV=2	PLOD2_HUMAN	85 kDa	0.3	[]	5	7	2	1	1	11
Procollagen-lysine,2-oxoglutarate 5-dioxygenase 3 OS=Homo sapiens GN=PLOD3 PE=1 SV=1	PLOD3_HUMAN	85 kDa	0.62	[]	3	3	1	3	3	14
Profilin-1 OS=Homo sapiens GN=PFN1 PE=1 SV=2	PROF1_HUMAN	15 kDa	0.69	[]	4	8	4	10	8	5
Programmed cell death 1 ligand 2 OS=Homo sapiens GN=PDCD1LG2 PE=1 SV=2	PD1L2_HUMAN (+2)	31 kDa	0.37	[]	0	0	2	0	0	0
Programmed cell death 6-interacting protein OS=Homo sapiens GN=PDCD6IP PE=1 SV=1	PDC6I_HUMAN	96 kDa	0.008	BRAC1 high, Naive low	2	2	6	11	13	14
Programmed cell death protein 4 OS=Homo sapiens GN=PDCD4 PE=1 SV=2	PDCD4_HUMAN (+2)	52 kDa	0.37	[]	0	0	0	0	0	5
Programmed cell death protein 6 OS=Homo sapiens GN=PDCD6 PE=1 SV=1	PDCD6_HUMAN (+1)	22 kDa	0.098	[]	1	2	2	6	8	4
Prohibitin, isoform CRA_a OS=Homo sapiens GN=PHB PE=2 SV=1	A8K401_HUMAN (+1)	30 kDa	0.14	[]	5	4	10	6	4	10
Prohibitin-2 OS=Homo sapiens GN=PHB2 PE=1 SV=2	J3KPX7_HUMAN (+1)	33 kDa	0.35	[]	4	6	6	3	9	9
Prolactin regulatory element-binding protein OS=Homo sapiens GN=PREB PE=1 SV=2	PREB_HUMAN (+2)	45 kDa	0.37	[]	0	0	0	0	0	2
Proliferating cell nuclear antigen OS=Homo sapiens GN=PCNA PE=1 SV=1	PCNA_HUMAN (+2)	29 kDa	0.0027	BRAC1 high, Naive low	0	0	0	1	1	2
Proliferation-associated 2G4, 38kDa, isoform CRA_a OS=Homo sapiens GN=PA2G4 PE=4 SV=1	A0A024RB85_HUMAN (+3)	44 kDa	0.32	[]	0	1	1	0	3	11
Prolow-density lipoprotein receptor-related protein 1 OS=Homo sapiens GN=LRP1 PE=1 SV=2	LRP1_HUMAN	505 kDa	0.21	[]	1	7	1	0	0	0
Prolyl 3-hydroxylase 1 OS=Homo sapiens GN=P3H1 PE=1 SV=2	P3H1_HUMAN (+1)	83 kDa	0.013	BRAC1 low, Naive high	1	3	3	0	0	0
Prolyl 3-hydroxylase 3 OS=Homo sapiens GN=P3H3 PE=1 SV=1	P3H3_HUMAN (+1)	82 kDa	0.19	[]	0	2	1	0	0	0
Prolyl 4-hydroxylase subunit alpha-1 OS=Homo sapiens GN=P4HA1 PE=1 SV=2	P4HA1_HUMAN	61 kDa	0.45	[]	4	5	9	0	2	18
Prolyl 4-hydroxylase subunit alpha-2 OS=Homo sapiens GN=P4HA2 PE=1 SV=1	P4HA2_HUMAN (+1)	61 kDa	0.28	[]	2	6	8	0	1	12
Prolyl endopeptidase FAP OS=Homo sapiens GN=FAP PE=1 SV=5	SEPR_HUMAN (+1)	88 kDa	0.1	[]	1	2	8	0	0	0
Prominin 2, isoform CRA_a OS=Homo sapiens GN=PROM2 PE=4 SV=1	A0A024RE18_HUMAN (+2)	92 kDa	0.028	BRAC1 high, Naive low	0	0	0	1	2	1
Prosaposin OS=Homo sapiens GN=PSAP PE=1 SV=2	SAP_HUMAN (+6)	58 kDa	0.37	[]	0	0	0	0	0	4

Prostacyclin synthase OS=Homo sapiens GN=PTGIS PE=1 SV=1	PTGIS_HUMAN (+2)	57 kDa	0.0019	BRAC1 low, Naive high	5	4	6	0	0	0
Prostaglandin E synthase 2 OS=Homo sapiens GN=PTGES2 PE=1 SV=1	PGES2_HUMAN	42 kDa	0.028	BRAC1 high, Naive low	0	0	0	2	1	4
Prostaglandin E synthase 3 (Cytosolic), isoform CRA_a OS=Homo sapiens GN=PTGES3 PE=4 SV=1	A0A024RB32_HUMAN (+3)	19 kDa	0.13	[]	0	0	0	1	0	2
Prostaglandin G/H synthase 1 OS=Homo sapiens GN=PTGS1 PE=1 SV=2	PGH1_HUMAN (+1)	69 kDa	0.16	[]	1	2	5	0	0	4
Prostasin OS=Homo sapiens GN=PRSS8 PE=1 SV=1	PRSS8_HUMAN	36 kDa	0.23	[]	0	0	0	0	1	5
Proteasomal ubiquitin receptor ADRM1 OS=Homo sapiens GN=ADRM1 PE=1 SV=2	ADRM1_HUMAN (+2)	42 kDa	0.87	[]	0	0	1	0	0	2
Proteasome (Prosome, macropain) subunit, beta type, 2, isoform CRA_b OS=Homo sapiens GN=PSMB2 PE=2 SV=1	B7Z478_HUMAN (+1)	20 kDa	0.018	BRAC1 high, Naive low	0	0	0	2	2	1
Proteasome activator complex subunit 2 OS=Homo sapiens GN=PSME2 PE=1 SV=1	A0A087X1Z3_HUMAN (+4)	29 kDa	0.35	[]	0	0	5	2	6	7
Proteasome subunit alpha type-1 OS=Homo sapiens GN=PSMA1 PE=1 SV=1	PSA1_HUMAN	30 kDa	0.6	[]	1	1	4	2	3	2
Proteasome subunit alpha type-2 OS=Homo sapiens GN=PSMA2 PE=1 SV=2	PSA2_HUMAN (+2)	26 kDa	0.87	[]	0	0	2	0	0	4
Proteasome subunit alpha type-5 OS=Homo sapiens GN=PSMA5 PE=1 SV=3	PSA5_HUMAN (+1)	26 kDa	0.82	[]	0	0	2	1	0	1
Proteasome-associated protein ECM29 homolog OS=Homo sapiens GN=ECM29 PE=1 SV=2	ECM29_HUMAN (+2)	204 kDa	0.37	[]	0	0	0	0	0	4
Protein 4.1 OS=Homo sapiens GN=EPB41 PE=1 SV=4	41_HUMAN (+4)	97 kDa	0.021	BRAC1 high, Naive low	0	0	0	8	5	4
Protein ARPC4-TTLL3 OS=Homo sapiens GN=ARPC4-TTLL3 PE=4 SV=1	A0A0A6YYG9_HUMAN (+3)	72 kDa	0.99	[]	2	1	1	3	3	1
Protein FAM83H OS=Homo sapiens GN=FAM83H PE=1 SV=3	FA83H_HUMAN (+1)	127 kDa	0.37	[]	0	0	0	0	0	3
Protein FAM91A1 OS=Homo sapiens GN=FAM91A1 PE=1 SV=3	F91A1_HUMAN	94 kDa	0.0027	BRAC1 high, Naive low	0	0	0	1	1	2
Protein FAM98A OS=Homo sapiens GN=FAM98A PE=1 SV=1	FA98A_HUMAN (+2)	55 kDa	0.87	[]	0	0	1	0	0	2
Protein GPR107 OS=Homo sapiens GN=GPR107 PE=1 SV=1	GP107_HUMAN (+1)	67 kDa	0.37	[]	0	0	0	0	0	2
Protein LAP2 OS=Homo sapiens GN=ERBB2IP PE=1 SV=2	LAP2_HUMAN (+1)	158 kDa	0.13	[]	0	3	1	7	10	4
Protein LYRIC OS=Homo sapiens GN=MTDH PE=1 SV=2	LYRIC_HUMAN (+1)	64 kDa	0.92	[]	0	3	0	0	2	3
Protein PML OS=Homo sapiens GN=PML PE=1 SV=3	PML_HUMAN (+1)	98 kDa	0.37	[]	0	0	2	0	0	0
Protein POF1B OS=Homo sapiens GN=POF1B PE=1 SV=3	POF1B_HUMAN	68 kDa	0.16	[]	0	0	0	1	2	10
Protein RER1 (Fragment) OS=Homo sapiens GN=RER1 PE=1 SV=1	A0A0A0MR06_HUMAN (+7)	21 kDa	0.36	[]	0	1	1	2	2	1
Protein S100-A4 OS=Homo sapiens GN=S100A4 PE=1 SV=1	S10A4_HUMAN	12 kDa	0.37	[]	0	0	0	2	0	0
Protein S100-A11 OS=Homo sapiens GN=S100A11 PE=1 SV=2	S10AB_HUMAN (+1)	12 kDa	0.014	BRAC1 low, Naive high	4	4	4	1	3	2
Protein S100-A14 OS=Homo sapiens GN=S100A14 PE=1 SV=1	S10AE_HUMAN	12 kDa	0.2	[]	0	0	0	1	3	0
Protein S100-A16 OS=Homo sapiens GN=S100A16 PE=1 SV=1	S10AG_HUMAN	12 kDa	0.16	[]	0	0	0	1	2	0
Protein S100-P OS=Homo sapiens GN=S100P PE=1 SV=2	S100P_HUMAN	10 kDa	0.13	[]	0	0	0	3	2	0
Protein SEC13 homolog OS=Homo sapiens GN=SEC13 PE=1 SV=1	A0A0C4DFR6_HUMAN (+3)	36 kDa	0.92	[]	0	0	3	1	0	4
Protein Shroom1 OS=Homo sapiens GN=SHROOM1 PE=1 SV=1	SHRM1_HUMAN (+1)	91 kDa	0.37	[]	0	0	0	0	0	2
Protein TSSC1 OS=Homo sapiens GN=TSSC1 PE=1 SV=2	TSSC1_HUMAN (+1)	44 kDa	0.37	[]	0	0	0	0	0	2
Protein Wnt OS=Homo sapiens PE=2 SV=1	Q5U0K5_HUMAN (+1)	39 kDa	0.16	[]	0	0	0	1	2	0
Protein XRP2 OS=Homo sapiens GN=RP2 PE=1 SV=4	XRP2_HUMAN (+1)	40 kDa	0.87	[]	0	0	1	0	0	2
Protein arginine N-methyltransferase 1 OS=Homo sapiens GN=PRMT1 PE=1 SV=2	ANM1_HUMAN (+3)	42 kDa	0.45	[]	0	0	2	1	2	2
Protein deglycase DJ-1 OS=Homo sapiens GN=PARK7 PE=1 SV=2	PARK7_HUMAN (+1)	20 kDa	0.15	[]	0	0	2	2	2	4
Protein diaphanous homolog 1 OS=Homo sapiens GN=DIAPH1 PE=1 SV=1	A0A0G2JH68_HUMAN (+2)	141 kDa	0.28	[]	0	0	0	1	0	8
Protein disulfide-isomerase A4 OS=Homo sapiens GN=ERP70 PE=3 SV=1	A0A090N8Y2_HUMAN (+1)	73 kDa	0.061	[]	4	5	7	15	17	41
Protein disulfide-isomerase A5 OS=Homo sapiens GN=PDIA5 PE=1 SV=1	PDIA5_HUMAN	60 kDa	0.37	[]	0	0	0	0	0	4
Protein disulfide-isomerase A6 OS=Homo sapiens GN=PDIA6 PE=1 SV=1	PDIA6_HUMAN	48 kDa	0.68	[]	23	25	18	34	29	45
Protein disulfide-isomerase OS=Homo sapiens GN=P4HB PE=1 SV=2	H7BZ94_HUMAN	53 kDa	0.058	[]	26	28	24	16	20	37
cDNA FLJ56766, highly similar to Protein disulfide-isomerase (EC5.3.4.1) OS=Homo sapiens PE=2 SV=1	B4DJS0_HUMAN	27 kDa	0.27	[]	21	24	16	17	26	35
Protein disulfide-isomerase A3 (Fragment) OS=Homo sapiens GN=PDIA3 PE=1 SV=1	H7BZJ3_HUMAN	14 kDa	0.15	[]	14	14	7	0	13	12
Protein disulfide-isomerase OS=Homo sapiens PE=2 SV=1	B3KQT9_HUMAN (+2)	54 kDa	0.38	[]	47	48	35	50	58	77
Protein disulfide-isomerase TMX3 OS=Homo sapiens GN=TMX3 PE=1 SV=2	TMX3_HUMAN	52 kDa	0.45	[]	0	0	4	0	0	1
Protein eva-1 homolog B OS=Homo sapiens GN=EVA1B PE=1 SV=1	EVA1B_HUMAN	18 kDa	0.12	[]	0	2	2	0	0	0
Protein kinase C OS=Homo sapiens PE=1 SV=1	B2RCZ4_HUMAN (+2)	67 kDa	0.13	[]	0	0	0	1	0	2
Protein kinase C alpha type OS=Homo sapiens GN=PRKCA PE=1 SV=4	KPCA_HUMAN (+3)	77 kDa	0.19	[]	0	2	1	0	0	0
Protein kinase C and casein kinase substrate in neurons 2, isoform CRA_a OS=Homo sapiens GN=PACSIN2 PE=4 SV=1	A0A024R4X1_HUMAN (+2)	50 kDa	0.11	[]	0	0	1	2	2	1
Protein kinase C delta type OS=Homo sapiens GN=PRKCD PE=1 SV=2	KPCD_HUMAN (+2)	78 kDa	0.13	[]	0	0	0	0	1	2
Protein kinase C delta-binding protein OS=Homo sapiens GN=PRKCDBP PE=1 SV=3	PRDBP_HUMAN	28 kDa	0.0001	BRAC1 low, Naive high	5	5	8	0	0	0
Protein kinase C substrate 80K-H, isoform CRA_a OS=Homo sapiens GN=PRKCSH PE=4 SV=1	A0A024R7F1_HUMAN (+2)	59 kDa	0.39	[]	2	2	3	2	1	6
Protein kinase, cAMP-dependent, regulatory, type II, alpha, isoform CRA_a OS=Homo sapiens GN=PRKAR2A PE=4 SV=1	A0A024R2W3_HUMAN (+2)	46 kDa	0.13	[]	0	0	0	2	0	4
Protein lin-7 homolog C OS=Homo sapiens GN=LIN7C PE=1 SV=1	LIN7C_HUMAN (+1)	22 kDa	0.12	[]	0	0	0	3	3	0
Protein lunapark OS=Homo sapiens GN=LNP PE=1 SV=2	LNP_HUMAN (+3)	48 kDa	0.25	[]	2	3	0	0	1	2
Protein pelota homolog OS=Homo sapiens GN=PELO PE=1 SV=2	PELO_HUMAN (+2)	43 kDa	0.69	[]	0	0	1	0	0	3
Protein phosphatase 1 regulatory subunit 12A OS=Homo sapiens GN=PPP1R12A PE=1 SV=1	MYPT1_HUMAN (+1)	115 kDa	0.0022	BRAC1 low, Naive high	2	3	4	0	0	2
Protein scribble homolog OS=Homo sapiens GN=SCRIB PE=1 SV=1	A0A0G2JNZ2_HUMAN (+1)	175 kDa	0.0075	BRAC1 high, Naive low	0	1	1	35	38	25
Protein sel-1 homolog 1 OS=Homo sapiens GN=SEL1L PE=1 SV=3	SE1L1_HUMAN	89 kDa	0.31	[]	0	2	3	0	1	2
Protein translocation complex beta variant (Fragment) OS=Homo sapiens PE=2 SV=1	Q53FA5_HUMAN (+1)	10 kDa	0.26	[]	0	1	0	1	1	3
Protein transport protein Sec16A OS=Homo sapiens GN=SEC16A PE=1 SV=3	SC16A_HUMAN (+4)	234 kDa	0.37	[]	0	0	0	0	0	4
Protein transport protein Sec23A OS=Homo sapiens GN=SEC23A PE=1 SV=2	SC23A_HUMAN	86 kDa	0.15	[]	5	3	9	1	2	10
Protein transport protein Sec24A OS=Homo sapiens GN=SEC24A PE=1 SV=2	SC24A_HUMAN (+1)	120 kDa	0.21	[]	0	0	0	0	1	4

Protein transport protein Sec24B OS=Homo sapiens GN=SEC24B PE=1 SV=2	SC24B_HUMAN	137 kDa	0.7	[]	0	1	0	0	0	4
Protein transport protein Sec24D OS=Homo sapiens GN=SEC24D PE=1 SV=2	SC24D_HUMAN (+5)	113 kDa	0.87	[]	0	0	1	0	0	2
Protein tweety homolog OS=Homo sapiens GN=TTYH3 PE=3 SV=1	A0A024R816_HUMAN (+1)	58 kDa	0.55	[]	0	2	0	1	0	0
Protein unc-45 homolog A OS=Homo sapiens GN=UNC45A PE=1 SV=1	UN45A_HUMAN	103 kDa	0.056	[]	0	0	0	4	1	7
Protein-glutamine gamma-glutamyltransferase 2 OS=Homo sapiens GN=TGM2 PE=1 SV=2	TGM2_HUMAN (+1)	77 kDa	0.66	[]	9	7	12	5	7	30
Protein-methionine sulfoxide oxidase MICAL1 OS=Homo sapiens GN=MICAL1 PE=1 SV=2	MICA1_HUMAN (+1)	118 kDa	0.37	[]	0	0	0	0	0	2
Protein-methionine sulfoxide oxidase MICAL2 OS=Homo sapiens GN=MICAL2 PE=1 SV=1	MICA2_HUMAN	127 kDa	0.37	[]	0	0	0	0	0	2
Protein-tyrosine-phosphatase OS=Homo sapiens GN=PTPRK PE=2 SV=1	Q86WJ2_HUMAN	162 kDa	0.093	[]	0	0	1	5	9	2
Proto-oncogene tyrosine-protein kinase Src OS=Homo sapiens GN=SRC PE=1 SV=3	SRC_HUMAN	60 kDa	0.0029	BRAC1 high, Naive low	0	1	1	8	12	11
Putative helicase MOV-10 OS=Homo sapiens GN=MOV10 PE=1 SV=2	MOV10_HUMAN (+2)	114 kDa	0.5	[]	0	2	0	0	0	1
Putative uncharacterized protein DKFZp547A0616 (Fragment) OS=Homo sapiens GN=DKFZp547A0616 PE=2 SV=1	Q5JQ44_HUMAN (+1)	20 kDa	0.2	[]	0	0	1	2	1	1
Putative uncharacterized protein DKFZp686E1899 OS=Homo sapiens GN=DKFZp686E1899 PE=2 SV=1	Q5HYL6_HUMAN (+1)	40 kDa	0.14	[]	3	1	1	6	6	7
Putative uncharacterized protein DKFZp686F17268 (Fragment) OS=Homo sapiens GN=DKFZp686F17268 PE=2 SV=1	A4GYY8_HUMAN (+7)	50 kDa	0.054	[]	9	9	12	12	12	10
Pyridoxal kinase OS=Homo sapiens GN=PDXK PE=1 SV=1	PDXK_HUMAN (+1)	35 kDa	0.27	[]	0	0	1	0	2	7
Pyridoxal-dependent decarboxylase domain-containing protein 1 OS=Homo sapiens GN=PDXDC1 PE=1 SV=2	PDXD1_HUMAN (+2)	87 kDa	0.37	[]	0	0	0	0	0	10
Pyrroline-5-carboxylate reductase 1, mitochondrial OS=Homo sapiens GN=PYCR1 PE=1 SV=2	P5CR1_HUMAN (+7)	33 kDa	0.63	[]	0	0	3	0	0	2
Pyruvate dehydrogenase E1 component subunit beta, mitochondrial OS=Homo sapiens GN=PDHB PE=1 SV=3	ODPB_HUMAN	39 kDa	0.34	[]	1	0	2	1	0	1
Pyruvate kinase OS=Homo sapiens GN=PKM2 PE=3 SV=1	A0A024R5Z9_HUMAN	58 kDa	0.22	[]	29	33	42	48	53	44
Pyruvate kinase OS=Homo sapiens PE=2 SV=1	B4DPM0_HUMAN (+3)	53 kDa	0.37	[]	0	0	0	0	1	0
Pyruvate kinase PKM OS=Homo sapiens GN=PKM PE=1 SV=4	KPYM_HUMAN (+1)	58 kDa	0.27	[]	35	39	47	55	64	54
Quinone oxidoreductase OS=Homo sapiens GN=CRYZ PE=1 SV=1	QOR_HUMAN	35 kDa	0.42	[]	0	1	3	2	2	7
RAB2, member RAS oncogene family, isoform CRA_a OS=Homo sapiens GN=RAB2 PE=3 SV=1	A0A024R7V6_HUMAN (+1)	24 kDa	0.66	[]	3	2	1	5	3	6
RAB5A, member RAS oncogene family, isoform CRA_a OS=Homo sapiens GN=RAB5A PE=3 SV=1	A0A024R2K1_HUMAN (+1)	24 kDa	0.82	[]	1	1	2	3	0	3
RAB5B, member RAS oncogene family, isoform CRA_a OS=Homo sapiens GN=RAB5B PE=3 SV=1	A0A024RB09_HUMAN (+1)	24 kDa	0.6	[]	3	2	0	1	2	4
RAB5C, member RAS oncogene family, isoform CRA_a OS=Homo sapiens GN=RAB5C PE=3 SV=1	A0A024R1U4_HUMAN (+1)	23 kDa	0.13	[]	6	4	8	4	8	6
RAB6A, member RAS oncogene family, isoform CRA_b OS=Homo sapiens GN=RAB6A PE=3 SV=1	A0A024R5H8_HUMAN (+2)	24 kDa	0.35	[]	1	1	1	5	3	1
RAB11A, member RAS oncogene family, isoform CRA_a OS=Homo sapiens GN=RAB11A PE=3 SV=1	A0A024R5Z8_HUMAN (+1)	24 kDa	0.46	[]	6	3	3	4	7	3
Ras-related protein Rab-11B OS=Homo sapiens GN=RAB11B PE=1 SV=4	RB11B_HUMAN	24 kDa	0.57	[]	5	2	2	3	6	2
RAB14, member RAS oncogene family, isoform CRA_a OS=Homo sapiens GN=RAB14 PE=3 SV=1	A0A024R845_HUMAN (+1)	24 kDa	0.18	[]	3	2	5	8	8	7
RAB21, member RAS oncogene family, isoform CRA_a OS=Homo sapiens GN=RAB21 PE=3 SV=1	A0A024RBA9_HUMAN (+1)	24 kDa	0.49	[]	1	2	1	3	4	2
RAN binding protein 5, isoform CRA_d OS=Homo sapiens GN=RANBP5 PE=4 SV=1	A0A024RDY0_HUMAN (+1)	124 kDa	0.31	[]	1	3	8	10	11	6
Ran-binding protein 6 OS=Homo sapiens GN=RANBP6 PE=1 SV=2	RNBP6_HUMAN	125 kDa	0.37	[]	0	0	0	0	0	1
RAP1A, member of RAS oncogene family OS=Homo sapiens GN=RAP1A PE=2 SV=1	A8KAH9_HUMAN (+1)	21 kDa	0.74	[]	9	8	4	22	17	5
RAP1B, member of RAS oncogene family, isoform CRA_a OS=Homo sapiens GN=RAP1B PE=4 SV=1	A0A024RB87_HUMAN (+1)	21 kDa	0.79	[]	10	8	5	22	18	6
RNA-binding motif protein, X chromosome OS=Homo sapiens GN=RBMX PE=1 SV=3	RBMX_HUMAN	42 kDa	0.21	[]	1	0	3	0	0	1
RNA-binding protein 39 OS=Homo sapiens GN=RBM39 PE=1 SV=2	RBM39_HUMAN (+10)	59 kDa	0.13	[]	0	0	0	0	1	2
RNA-binding protein 47 OS=Homo sapiens GN=RBM47 PE=1 SV=2	RBM47_HUMAN (+3)	64 kDa	0.13	[]	0	0	0	0	1	2
RNA-binding protein EWS OS=Homo sapiens GN=EWSR1 PE=1 SV=1	A0A0D9SFL3_HUMAN (+4)	61 kDa	0.0027	BRAC1 high, Naive low	0	0	0	1	1	2
RPLP1 protein OS=Homo sapiens GN=RPLP1 PE=2 SV=1	Q6FG99_HUMAN	12 kDa	0.37	[]	0	0	0	2	0	0
RSU1 protein (Fragment) OS=Homo sapiens GN=RSU1 PE=2 SV=1	Q32Q10_HUMAN (+1)	31 kDa	0.83	[]	0	1	2	2	1	2
Rab3 GTPase-activating protein non-catalytic subunit OS=Homo sapiens GN=RAB3GAP2 PE=1 SV=1	RBGPR_HUMAN (+1)	156 kDa	0.37	[]	0	0	0	0	0	2
Rac GTPase activating protein 1, isoform CRA_a OS=Homo sapiens GN=RACGAP1 PE=4 SV=1	A0A024R136_HUMAN (+2)	71 kDa	0.017	BRAC1 high, Naive low	0	1	1	6	8	5
Raftlin OS=Homo sapiens GN=RFTN1 PE=1 SV=4	RFTN1_HUMAN (+1)	63 kDa	0.041	BRAC1 low, Naive high	1	5	7	0	0	0
Ragulator complex protein LAMTOR1 OS=Homo sapiens GN=LAMTOR1 PE=1 SV=2	LTOR1_HUMAN	18 kDa	0.71	[]	2	1	0	2	2	0
Ran GTPase-activating protein 1 OS=Homo sapiens GN=RANGAP1 PE=1 SV=1	RAGP1_HUMAN (+2)	64 kDa	0.37	[]	0	0	0	0	0	3
Ran-specific GTPase-activating protein OS=Homo sapiens GN=RANBP1 PE=1 SV=1	RANG_HUMAN	23 kDa	0.37	[]	0	0	0	0	0	3
Ras GTPase-activating protein 3 OS=Homo sapiens GN=RASA3 PE=1 SV=3	RASA3_HUMAN	96 kDa	0.16	[]	0	0	0	1	2	0
Ras GTPase-activating protein-binding protein 1 OS=Homo sapiens GN=G3BP1 PE=1 SV=1	G3BP1_HUMAN (+2)	52 kDa	0.79	[]	0	0	3	0	1	2
HCG2043376, isoform CRA_b OS=Homo sapiens GN=hCG_2043376 PE=3 SV=1	A0A024R0I3_HUMAN (+3)	22 kDa	0.17	[]	3	3	3	9	9	6
Ras homolog gene family, member A, isoform CRA_a OS=Homo sapiens GN=RHOA PE=3 SV=1	A0A024R324_HUMAN (+3)	22 kDa	0.29	[]	3	4	3	11	8	6
Ras-GTPase activating protein SH3 domain-binding protein 2, isoform CRA_b OS=Homo sapiens GN=G3BP2 PE=4 SV=1	A0A024RDB2_HUMAN (+3)	51 kDa	0.13	[]	0	0	0	1	0	2
Ras-related C3 botulinum toxin substrate 1 (Rho family, small GTP binding protein Rac1) OS=Homo sapiens GN=RAC1 PE=2 SV=1	A4D2P0_HUMAN	23 kDa	0.13	[]	1	1	0	6	9	2
Ras-related C3 botulinum toxin substrate 1 (Rho family, small GTP binding protein Rac1) OS=Homo sapiens GN=RAC1 PE=2 SV=1	A4D2P1_HUMAN (+1)	21 kDa	0.086	[]	0	0	0	2	6	2
Ras-related C3 botulinum toxin substrate 2 (Rho family, small GTP binding protein Rac2), isoform CRA_a OS=Homo sapiens GN=RAC2	A0A024R1P2_HUMAN (+2)	21 kDa	0.5	[]	1	1	0	1	3	2
Ras-related protein Rab-7a OS=Homo sapiens GN=RAB7A PE=1 SV=1	RAB7A_HUMAN	23 kDa	0.55	[]	6	5	6	6	10	10
RAB8A, member RAS oncogene family, isoform CRA_a OS=Homo sapiens GN=RAB8A PE=3 SV=1	A0A024R7I3_HUMAN (+1)	24 kDa	0.0022	BRAC1 high, Naive low	0	0	1	5	4	5
Ras-related protein Rab-8B (Fragment) OS=Homo sapiens GN=RAB8B PE=1 SV=1	H0YNE9_HUMAN (+1)	22 kDa	0.12	[]	0	1	1	0	0	0
Ras-related protein Rab-10 OS=Homo sapiens GN=RAB10 PE=1 SV=1	RAB10_HUMAN	23 kDa	0.072	[]	2	1	3	14	13	6
Ras-related protein Rab-25 OS=Homo sapiens GN=RAB25 PE=1 SV=2	RAB25_HUMAN	23 kDa	0.04	BRAC1 high, Naive low	0	0	0	2	3	1
Ras-related protein Rab-34 OS=Homo sapiens GN=RAB34 PE=1 SV=1	RAB34_HUMAN (+3)	29 kDa	0.00091	BRAC1 low, Naive high	1	1	2	0	0	0
Ras-related protein Rab-35 (Fragment) OS=Homo sapiens GN=RAB35 PE=1 SV=1	F5H157_HUMAN (+1)	21 kDa	0.14	[]	1	0	0	7	5	1
Ras-related protein Ral-A (Fragment) OS=Homo sapiens GN=RALA PE=1 SV=1	H7C3P7_HUMAN (+1)	18 kDa	0.032	BRAC1 high, Naive low	0	1	2	9	8	5

RecName: Full=Serum albumin; AltName: Full=BSA; AltName:	sp|ALBU_BOVIN|	69 kDa	0.18	[]	7	11	43	4	5	19
RecName: Full=Trypsin; Flags: Precursor.	sp|TRYP_PIG|	24 kDa	0.081	[]	22	12	15	9	9	3
Receptor protein-tyrosine kinase OS=Homo sapiens GN=EPHA2 PE=4 SV=1	A0A024QZA8_HUMAN (+1)	108 kDa	0.26	[]	1	3	3	10	6	4
Receptor protein-tyrosine kinase OS=Homo sapiens GN=EPHB2 PE=1 SV=1	B1AKC9_HUMAN (+3)	106 kDa	0.12	[]	0	0	0	3	3	0
Receptor protein-tyrosine kinase OS=Homo sapiens GN=ERBB2 PE=1 SV=1	B4DTR1_HUMAN (+2)	108 kDa	0.21	[]	1	0	1	7	4	1
Receptor protein-tyrosine kinase OS=Homo sapiens GN=MET PE=3 SV=1	A0A024R759_HUMAN (+1)	156 kDa	0.12	[]	0	0	0	2	2	0
Receptor protein-tyrosine kinase OS=Homo sapiens PE=2 SV=1	A8K2T7_HUMAN (+5)	134 kDa	0.99	[]	2	3	2	4	5	3
Receptor protein-tyrosine kinase OS=Homo sapiens PE=2 SV=1	A8K2T6_HUMAN (+1)	108 kDa	0.00029	BRAC1 high, Naive low	0	0	0	2	2	2
Protein-tyrosine-phosphatase OS=Homo sapiens PE=2 SV=1	B3KWM1_HUMAN	143 kDa	0.00029	BRAC1 high, Naive low	0	0	0	4	3	4
Receptor-type tyrosine-protein phosphatase F (Fragment) OS=Homo sapiens GN=PTPRF PE=1 SV=1	H0Y6Z7_HUMAN (+1)	175 kDa	0.0043	BRAC1 high, Naive low	0	0	0	5	6	5
Receptor-type tyrosine-protein phosphatase OS=Homo sapiens PE=2 SV=1	B7Z2A4_HUMAN (+3)	92 kDa	0.24	[]	0	0	0	4	1	0
Related RAS viral (R-ras) oncogene homolog, isoform CRA_a OS=Homo sapiens GN=RRAS PE=4 SV=1	A0A024QZF2_HUMAN (+1)	23 kDa	0.053	[]	4	7	5	4	4	6
Reticulocalbin-1 OS=Homo sapiens GN=RCN1 PE=1 SV=1	RCN1_HUMAN (+1)	39 kDa	0.16	[]	5	6	8	3	6	13
Reticulocalbin-2 OS=Homo sapiens GN=RCN2 PE=1 SV=1	RCN2_HUMAN	37 kDa	0.83	[]	1	2	0	2	1	2
Reticulocalbin-3 OS=Homo sapiens GN=RCN3 PE=1 SV=1	RCN3_HUMAN	37 kDa	0.008	BRAC1 low, Naive high	5	3	8	0	0	0
Reticulon OS=Homo sapiens GN=RTN4 PE=1 SV=1	F8W914_HUMAN	37 kDa	0.11	[]	4	7	25	1	0	7
Reticulon-4 OS=Homo sapiens GN=RTN4 PE=1 SV=2	RTN4_HUMAN	130 kDa	0.12	[]	4	6	24	1	0	6
Retinal dehydrogenase 1 OS=Homo sapiens GN=ALDH1A1 PE=1 SV=2	AL1A1_HUMAN (+2)	55 kDa	0.65	[]	7	5	15	13	18	18
Retinol dehydrogenase 11 OS=Homo sapiens GN=RDH11 PE=1 SV=2	RDH11_HUMAN	35 kDa	0.84	[]	2	0	1	2	1	4
Retinol dehydrogenase 13 OS=Homo sapiens GN=RDH13 PE=1 SV=2	RDH13_HUMAN (+1)	36 kDa	0.37	[]	0	0	0	0	0	2
Rho GDP-dissociation inhibitor 1 OS=Homo sapiens GN=ARHGDIA PE=1 SV=3	GDIR1_HUMAN (+3)	23 kDa	0.48	[]	1	1	4	0	3	3
Rho GTPase activating protein OS=Homo sapiens PE=2 SV=1	A9UK01_HUMAN (+1)	75 kDa	0.27	[]	0	0	0	0	1	7
Rho GTPase-activating protein 1 OS=Homo sapiens GN=ARHGAP1 PE=1 SV=1	RHG01_HUMAN	50 kDa	0.57	[]	1	1	5	0	0	7
Rho GTPase-activating protein 35 OS=Homo sapiens GN=ARHGAP35 PE=1 SV=3	RHG35_HUMAN	171 kDa	0.13	[]	0	0	0	1	0	2
Rho guanine nucleotide exchange factor 2 OS=Homo sapiens GN=ARHGEF2 PE=1 SV=4	ARHG2_HUMAN (+2)	112 kDa	0.37	[]	0	0	0	0	0	3
Rho guanine nucleotide exchange factor 12 OS=Homo sapiens GN=ARHGEF12 PE=1 SV=1	ARHGC_HUMAN (+1)	173 kDa	0.37	[]	0	0	0	0	0	2
Rho guanine nucleotide exchange factor 16 OS=Homo sapiens GN=ARHGEF16 PE=1 SV=1	ARHGG_HUMAN (+1)	80 kDa	0.14	[]	0	0	0	1	1	6
Rho guanine nucleotide exchange factor (GEF) 1, isoform CRA_e OS=Homo sapiens GN=ARHGEF1 PE=4 SV=1	A0A024R0R1_HUMAN (+2)	102 kDa	0.095	[]	0	0	0	1	6	13
Rho-associated protein kinase 2 OS=Homo sapiens GN=ROCK2 PE=1 SV=4	ROCK2_HUMAN	161 kDa	0.17	[]	0	0	1	2	5	1
Rhophilin-2 OS=Homo sapiens GN=RHPN2 PE=1 SV=1	RHPN2_HUMAN	77 kDa	0.37	[]	0	0	0	0	0	3
Ribonuclease inhibitor (Fragment) OS=Homo sapiens GN=RNH1 PE=1 SV=1	H0YCR7_HUMAN	30 kDa	0.98	[]	0	0	5	2	0	7
Ribonuclease inhibitor OS=Homo sapiens GN=RNH1 PE=1 SV=2	RINI_HUMAN	50 kDa	0.58	[]	2	4	6	2	3	12
Ribonucleoside-diphosphate reductase large subunit OS=Homo sapiens GN=RRM1 PE=1 SV=1	RIR1_HUMAN (+2)	90 kDa	0.37	[]	0	0	0	0	0	6
Ribosomal L1 domain-containing protein 1 OS=Homo sapiens GN=RSL1D1 PE=1 SV=3	RL1D1_HUMAN (+3)	55 kDa	0.37	[]	0	0	0	0	0	4
Ribosomal protein L5 OS=Homo sapiens GN=RPL5 PE=2 SV=1	A2RUM7_HUMAN (+3)	34 kDa	0.092	[]	5	3	6	4	3	6
Ribosomal protein L7, isoform CRA_a OS=Homo sapiens GN=RPL7 PE=3 SV=1	A0A024R814_HUMAN (+1)	30 kDa	0.55	[]	3	2	6	6	5	11
Ribosomal protein L11, isoform CRA_b OS=Homo sapiens GN=RPL11 PE=2 SV=1	Q5VVD0_HUMAN (+1)	20 kDa	0.98	[]	1	1	1	2	2	1
Ribosomal protein L13a variant (Fragment) OS=Homo sapiens PE=2 SV=1	Q53H34_HUMAN (+2)	24 kDa	0.19	[]	3	3	3	3	4	5
Ribosomal protein L15 OS=Homo sapiens GN=RPL15 PE=3 SV=1	A0A024R2Q4_HUMAN (+3)	24 kDa	0.39	[]	0	0	2	1	1	6
Ribosomal protein L18, isoform CRA_c OS=Homo sapiens GN=RPL18 PE=4 SV=1	A0A024QZD1_HUMAN (+4)	22 kDa	0.64	[]	7	4	3	8	5	8
Ribosomal protein L19 OS=Homo sapiens GN=RPL19 PE=1 SV=1	J3KTE4_HUMAN (+4)	23 kDa	0.87	[]	2	1	1	3	2	3
Ribosomal protein L23, isoform CRA_b OS=Homo sapiens GN=RPL23 PE=3 SV=1	A0A024R1Q8_HUMAN (+4)	15 kDa	0.82	[]	1	1	1	2	1	2
Ribosomal protein L30, isoform CRA_b OS=Homo sapiens GN=RPL30 PE=3 SV=1	A0A024R9D3_HUMAN (+2)	13 kDa	0.74	[]	0	1	2	1	1	4
Ribosomal protein L35, isoform CRA_a OS=Homo sapiens GN=RPL35 PE=3 SV=1	A0A024R866_HUMAN (+2)	18 kDa	0.82	[]	1	2	1	3	2	1
Ribosomal protein S2 OS=Homo sapiens GN=RPS2 PE=2 SV=1	Q3KQT6_HUMAN (+2)	31 kDa	0.71	[]	1	1	4	2	1	11
Ribosomal protein S5, isoform CRA_a OS=Homo sapiens GN=RPS5 PE=3 SV=1	A0A024R4Q8_HUMAN (+3)	23 kDa	0.9	[]	3	3	3	3	4	9
Ribosomal protein S6 kinase alpha-1 OS=Homo sapiens GN=RPS6KA1 PE=1 SV=2	KS6A1_HUMAN (+2)	83 kDa	0.37	[]	0	0	0	0	0	4
Ribosomal protein S6 kinase alpha-3 OS=Homo sapiens GN=RPS6KA3 PE=1 SV=1	KS6A3_HUMAN (+3)	84 kDa	0.37	[]	0	0	0	0	0	3
Ribosomal protein, large, P0, isoform CRA_b OS=Homo sapiens GN=RPLP0 PE=3 SV=1	A0A024RBS2_HUMAN (+3)	34 kDa	0.11	[]	6	9	10	8	7	16
Ribosomal protein, large, P1, isoform CRA_a OS=Homo sapiens GN=RPLP1 PE=3 SV=1	A0A024R608_HUMAN (+2)	12 kDa	0.88	[]	0	0	2	1	1	1
Ribosomal protein, large, P2, isoform CRA_a OS=Homo sapiens GN=RPLP2 PE=3 SV=1	A0A024RCA7_HUMAN (+1)	12 kDa	0.063	[]	7	6	6	6	5	3
Ribosome-binding protein 1 OS=Homo sapiens GN=RRBP1 PE=1 SV=1	A0A0A0MRV0_HUMAN (+2)	152 kDa	0.0069	BRAC1 low, Naive high	22	29	23	7	10	11
RuvB-like 1 (Fragment) OS=Homo sapiens GN=RUVBL1 PE=2 SV=1	B5BUB1_HUMAN (+1)	50 kDa	0.13	[]	0	1	3	5	3	12
RuvB-like 2 OS=Homo sapiens GN=RUVBL2 PE=1 SV=3	RUVB2_HUMAN	51 kDa	0.11	[]	0	0	1	1	5	11
S100 calcium binding protein A10 (Annexin II ligand, calpactin I, light polypeptide (P11)), isoform CRA_b (Fragment) OS=Homo sapiens	D3DV26_HUMAN (+2)	22 kDa	0.4	[]	3	1	2	10	8	1
S-methyl-5'-thioadenosine phosphorylase OS=Homo sapiens GN=MTAP PE=1 SV=1	B4DUC8_HUMAN (+1)	33 kDa	0.23	[]	0	0	0	1	0	5
SCAMP3 protein (Fragment) OS=Homo sapiens GN=SCAMP3 PE=2 SV=1	Q6FHJ5_HUMAN (+1)	38 kDa	0.68	[]	0	3	2	1	2	3
SEC23-interacting protein OS=Homo sapiens GN=SEC23IP PE=1 SV=1	S23IP_HUMAN (+3)	111 kDa	0.37	[]	0	0	0	0	0	2
SEC24 related gene family, member C (S. cerevisiae), isoform CRA_a OS=Homo sapiens GN=SEC24C PE=4 SV=1	A0A024QZM6_HUMAN (+3)	118 kDa	0.2	[]	1	0	1	2	4	2
SEC31-like 1 (S. cerevisiae), isoform CRA_d OS=Homo sapiens GN=SEC31L1 PE=4 SV=1	A0A024RDD3_HUMAN (+3)	133 kDa	0.36	[]	0	0	1	1	0	9
SH3 domain-binding protein 1 OS=Homo sapiens GN=SH3BP1 PE=1 SV=3	3BP1_HUMAN (+4)	76 kDa	0.37	[]	0	0	0	0	0	2
SH3-domain GRB2-like endophilin B2, isoform CRA_a OS=Homo sapiens GN=SH3GLB2 PE=4 SV=1	A0A024R896_HUMAN (+2)	44 kDa	0.07	[]	0	0	0	1	1	4

SHC-transforming protein 1 OS=Homo sapiens GN=SHC1 PE=1 SV=4	SHC1_HUMAN (+4)	63 kDa	0.37	[]	0	0	0	0	0	2
SPARC OS=Homo sapiens GN=SPARC PE=1 SV=1	SPRC_HUMAN (+3)	35 kDa	0.37	[]	0	0	2	0	0	0
SPATS2-like protein OS=Homo sapiens GN=SPATS2L PE=1 SV=2	SPS2L_HUMAN (+3)	62 kDa	0.37	[]	0	0	0	0	0	2
SPINT2 protein (Fragment) OS=Homo sapiens GN=SPINT2 PE=2 SV=1	Q6LEU8_HUMAN (+1)	28 kDa	0.003	BRAC1 high, Naive low	0	0	0	3	4	3
Uncharacterized protein (Fragment) OS=Homo sapiens PE=4 SV=1	K7EQZ3_HUMAN	16 kDa	0.37	[]	0	0	0	0	1	0
START domain containing 10, isoform CRA_a OS=Homo sapiens GN=STARD10 PE=4 SV=1	A0A024R5L8_HUMAN (+5)	33 kDa	0.11	[]	0	0	0	1	1	5
STX12 protein (Fragment) OS=Homo sapiens GN=STX12 PE=2 SV=1	Q6LEU0_HUMAN (+1)	31 kDa	0.061	[]	3	1	3	1	0	0
SUMO-activating enzyme subunit 1 OS=Homo sapiens GN=SAE1 PE=1 SV=1	SAE1_HUMAN (+2)	38 kDa	0.13	[]	0	0	0	0	1	2
SUMO-activating enzyme subunit 2 OS=Homo sapiens GN=UBA2 PE=1 SV=2	SAE2_HUMAN (+3)	71 kDa	0.37	[]	0	0	0	0	0	3
SUN domain-containing protein 2 OS=Homo sapiens GN=SUN2 PE=1 SV=3	SUN2_HUMAN (+4)	80 kDa	0.48	[]	0	0	3	0	0	1
Sarcoplasmic/endoplasmic reticulum calcium ATPase 2 OS=Homo sapiens GN=ATP2A2 PE=1 SV=1	AT2A2_HUMAN	115 kDa	0.12	[]	7	18	11	7	7	14
Scavenger receptor class B, member 2, isoform CRA_a OS=Homo sapiens GN=SCARB2 PE=4 SV=1	A0A024RDG6_HUMAN (+1)	54 kDa	0.21	[]	7	7	2	2	4	7
Sec1 family domain-containing protein 1 OS=Homo sapiens GN=SCFD1 PE=1 SV=4	SCFD1_HUMAN (+1)	72 kDa	0.35	[]	1	0	2	0	0	2
Sec1 family domain-containing protein 2 OS=Homo sapiens GN=SCFD2 PE=1 SV=2	SCFD2_HUMAN	75 kDa	0.37	[]	0	0	0	0	0	2
Sec61 alpha 1 subunit (S. cerevisiae), isoform CRA_c OS=Homo sapiens GN=SEC61A1 PE=2 SV=1	B3KNF6_HUMAN (+2)	52 kDa	0.81	[]	1	3	4	1	4	11
Selenoprotein O OS=Homo sapiens GN=SELO PE=2 SV=3	SELO_HUMAN (+1)	73 kDa	0.37	[]	0	0	0	0	0	3
Sepiapterin reductase OS=Homo sapiens GN=SPR PE=1 SV=1	SPRE_HUMAN	28 kDa	0.0037	BRAC1 high, Naive low	0	0	0	4	4	3
Septin 9, isoform CRA_a OS=Homo sapiens GN=SEPT9 PE=3 SV=1	A0A024R8V0_HUMAN (+1)	64 kDa	0.61	[]	2	6	13	7	9	10
Septin-2 OS=Homo sapiens GN=SEPT2 PE=1 SV=1	SEPT2_HUMAN	41 kDa	0.28	[]	11	10	9	12	13	15
Septin-8 OS=Homo sapiens GN=SEPT8 PE=1 SV=1	A6NMH6_HUMAN (+1)	50 kDa	0.00019	BRAC1 high, Naive low	0	0	0	5	6	6
Septin-11 OS=Homo sapiens GN=SEPT11 PE=1 SV=1	D6RER5_HUMAN (+2)	50 kDa	0.075	[]	5	6	10	7	9	5
Sequestosome-1 OS=Homo sapiens GN=SQSTM1 PE=1 SV=1	E7EMC7_HUMAN (+1)	41 kDa	0.24	[]	1	2	0	3	3	4
Serine hydroxymethyltransferase OS=Homo sapiens GN=SHMT2 PE=3 SV=1	A0A024RB99_HUMAN (+4)	53 kDa	0.045	BRAC1 low, Naive high	4	4	4	3	4	5
Serine hydroxymethyltransferase OS=Homo sapiens PE=2 SV=1	B4DW25_HUMAN	48 kDa	0.045	BRAC1 low, Naive high	4	4	4	3	4	5
Serine hydroxymethyltransferase OS=Homo sapiens PE=2 SV=1	B4DP88_HUMAN	48 kDa	0.02	BRAC1 low, Naive high	4	4	4	2	3	4
Serine incorporator 1 OS=Homo sapiens GN=SERINC1 PE=1 SV=1	SERC1_HUMAN	50 kDa	0.62	[]	1	1	2	3	1	1
Serine palmitoyltransferase 2 OS=Homo sapiens GN=SPTLC2 PE=1 SV=1	SPTC2_HUMAN (+1)	63 kDa	0.17	[]	0	0	0	0	1	3
Serine palmitoyltransferase, long chain base subunit 1, isoform CRA_a OS=Homo sapiens GN=SPTLC1 PE=4 SV=1	A0A024R277_HUMAN (+1)	53 kDa	0.37	[]	0	0	2	1	1	7
STE20/SPS1-related proline-alanine-rich protein kinase OS=Homo sapiens GN=STK39 PE=1 SV=3	STK39_HUMAN (+2)	59 kDa	0.11	[]	0	0	0	1	4	13
Serine threonine kinase 39 isoform D (Fragment) OS=Homo sapiens GN=STK39 PE=2 SV=1	X5D7P8_HUMAN	52 kDa	0.11	[]	0	0	0	1	4	13
Serine--tRNA ligase, cytoplasmic OS=Homo sapiens GN=SARS PE=1 SV=3	SYSC_HUMAN (+3)	59 kDa	0.87	[]	0	0	3	0	0	6
Serine--tRNA ligase, mitochondrial OS=Homo sapiens GN=SARS2 PE=1 SV=1	M0QWZ7_HUMAN (+2)	58 kDa	0.011	BRAC1 high, Naive low	0	0	0	1	2	3
Serine-threonine kinase receptor-associated protein OS=Homo sapiens GN=STRAP PE=1 SV=1	STRAP_HUMAN	38 kDa	0.26	[]	0	0	1	2	0	5
Serine/arginine-rich splicing factor 3 OS=Homo sapiens GN=SRSF3 PE=1 SV=1	SRSF3_HUMAN (+2)	19 kDa	0.47	[]	2	1	0	0	0	3
Serine/arginine-rich splicing factor 6 OS=Homo sapiens GN=SRSF6 PE=1 SV=2	SRSF6_HUMAN (+3)	40 kDa	0.37	[]	0	0	0	0	0	2
Serine/arginine-rich splicing factor 7 OS=Homo sapiens GN=SRSF7 PE=1 SV=1	SRSF7_HUMAN (+3)	27 kDa	0.37	[]	0	0	0	0	0	2
Serine/arginine-rich splicing factor 10 OS=Homo sapiens GN=SRSF10 PE=1 SV=1	SRS10_HUMAN (+2)	31 kDa	0.37	[]	0	0	0	0	0	2
Serine/threonine-protein kinase 24 OS=Homo sapiens GN=STK24 PE=1 SV=1	B4DR80_HUMAN (+4)	46 kDa	0.077	[]	0	0	1	1	4	6
Serine/threonine-protein kinase 26 OS=Homo sapiens GN=STK26 PE=1 SV=1	B4E0Y9_HUMAN (+3)	49 kDa	0.078	[]	0	0	0	1	2	6
Serine/threonine-protein kinase MARK2 OS=Homo sapiens GN=MARK2 PE=1 SV=3	E9PC69_HUMAN	87 kDa	0.024	BRAC1 high, Naive low	0	0	0	5	2	8
Serine/threonine-protein kinase N2 OS=Homo sapiens GN=PKN2 PE=1 SV=1	PKN2_HUMAN	112 kDa	0.031	BRAC1 high, Naive low	0	0	0	2	1	1
Serine/threonine-protein kinase OSR1 OS=Homo sapiens GN=OXSR1 PE=1 SV=1	OXSR1_HUMAN (+1)	58 kDa	0.98	[]	0	0	2	0	0	3
Serine/threonine-protein kinase TBK1 OS=Homo sapiens GN=TBK1 PE=1 SV=1	TBK1_HUMAN (+1)	84 kDa	0.37	[]	0	0	0	0	0	3
Serine/threonine-protein kinase mTOR OS=Homo sapiens GN=MTOR PE=1 SV=1	MTOR_HUMAN (+3)	289 kDa	0.37	[]	0	0	0	0	0	2
Serine/threonine-protein phosphatase 2A 65 kDa regulatory subunit A alpha isoform OS=Homo sapiens GN=PPP2R1A PE=1 SV=4	2AAA_HUMAN (+3)	65 kDa	0.17	[]	0	1	7	6	9	11
Serine/threonine-protein phosphatase 2A catalytic subunit alpha isoform OS=Homo sapiens GN=PPP2CA PE=1 SV=1	PP2AA_HUMAN (+6)	36 kDa	0.79	[]	0	0	2	0	1	3
Serine/threonine-protein phosphatase 2B catalytic subunit alpha isoform OS=Homo sapiens GN=PPP3CA PE=1 SV=1	PP2BA_HUMAN	59 kDa	0.12	[]	0	0	0	0	2	3
Serine/threonine-protein phosphatase 6 regulatory subunit 1 OS=Homo sapiens GN=PPP6R1 PE=1 SV=5	PP6R1_HUMAN	97 kDa	0.37	[]	0	0	0	0	0	2
Serine/threonine-protein phosphatase 6 regulatory subunit 3 OS=Homo sapiens GN=PPP6R3 PE=1 SV=2	PP6R3_HUMAN (+3)	98 kDa	0.37	[]	0	0	0	0	0	4
Serine/threonine-protein phosphatase OS=Homo sapiens GN=PPP5C PE=3 SV=1	A0A024R0Q7_HUMAN (+6)	57 kDa	0.018	BRAC1 high, Naive low	0	0	0	2	2	1
Serine/threonine-protein phosphatase PGAM5, mitochondrial OS=Homo sapiens GN=PGAM5 PE=1 SV=2	PGAM5_HUMAN	32 kDa	0.13	[]	0	0	0	0	2	4
Serine/threonine-protein phosphatase OS=Homo sapiens GN=PPP1CC PE=3 SV=1	A0A024RBP2_HUMAN (+3)	37 kDa	0.042	BRAC1 high, Naive low	0	2	5	15	11	10
Serine/threonine-protein phosphatase PP1-alpha catalytic subunit OS=Homo sapiens GN=PPP1CA PE=1 SV=1	PP1A_HUMAN	38 kDa	0.034	BRAC1 high, Naive low	2	4	5	15	13	11
Serine/threonine-protein phosphatase PP1-beta catalytic subunit OS=Homo sapiens GN=PPP1CB PE=1 SV=3	PP1B_HUMAN (+1)	37 kDa	0.059	[]	1	4	6	15	12	11
Serpin B6 OS=Homo sapiens GN=SERPINB6 PE=1 SV=3	SPB6_HUMAN (+3)	43 kDa	0.12	[]	0	0	0	0	2	2
Serpin peptidase inhibitor, clade H (Heat shock protein 47), member 1, (Collagen binding protein 1), isoform CRA_a OS=Homo sapiens	A0A024R5K8_HUMAN (+1)	46 kDa	0.027	BRAC1 low, Naive high	42	37	29	8	13	14
Serum deprivation-response protein OS=Homo sapiens GN=SDPR PE=1 SV=3	SDPR_HUMAN (+1)	47 kDa	0.12	[]	1	0	2	0	0	0
Sickle tail protein homolog OS=Homo sapiens GN=KIAA1217 PE=1 SV=2	SKT_HUMAN	214 kDa	0.37	[]	0	0	0	0	2	0
Sideroflexin OS=Homo sapiens GN=SFXN3 PE=1 SV=1	A0A0A0MS41_HUMAN	36 kDa	0.13	[]	3	2	9	2	2	4
Sideroflexin-1 OS=Homo sapiens GN=SFXN1 PE=1 SV=4	SFXN1_HUMAN	36 kDa	0.58	[]	2	1	3	1	7	6
Signal recognition particle receptor beta subunit OS=Homo sapiens GN=SRPRB PE=2 SV=1	Q549N5_HUMAN (+1)	30 kDa	0.37	[]	2	2	2	3	4	6
Signal recognition particle receptor subunit alpha OS=Homo sapiens GN=SRPR PE=1 SV=2	SRPR_HUMAN	70 kDa	0.15	[]	0	2	1	3	3	9

Signal recognition particle subunit SRP68 OS=Homo sapiens GN=SRP68 PE=1 SV=2	SRP68_HUMAN	71 kDa	0.37	[]	0	0	0	0	0	4
Signal recognition particle subunit SRP72 OS=Homo sapiens GN=SRP72 PE=1 SV=3	SRP72_HUMAN (+1)	75 kDa	0.87	[]	0	0	2	0	0	4
Signal transducer and activator of transcription 1-alpha/beta OS=Homo sapiens GN=STAT1 PE=1 SV=2	STAT1_HUMAN	87 kDa	0.57	[]	4	3	17	6	4	17
Signal transducer and activator of transcription 3 OS=Homo sapiens GN=STAT3 PE=1 SV=2	STAT3_HUMAN (+5)	88 kDa	0.69	[]	0	0	1	0	0	3
Signal-induced proliferation-associated 1 like 3 OS=Homo sapiens GN=SIPA1L3 PE=2 SV=1	B2RWP0_HUMAN (+1)	195 kDa	0.16	[]	0	0	0	2	1	0
Single Ig IL-1-related receptor OS=Homo sapiens GN=SIGIRR PE=1 SV=3	SIGIR_HUMAN	46 kDa	0.00058	BRAC1 high, Naive low	0	0	0	4	3	4
Single-stranded DNA-binding protein OS=Homo sapiens GN=SSBP1 PE=2 SV=1	A4D1U3_HUMAN (+2)	17 kDa	0.23	[]	5	4	2	4	4	0
Sister chromatid cohesion protein PDS5 homolog A OS=Homo sapiens GN=PDS5A PE=1 SV=1	PDS5A_HUMAN (+1)	151 kDa	0.37	[]	0	0	0	0	0	2
Sister chromatid cohesion protein PDS5 homolog B OS=Homo sapiens GN=PDS5B PE=1 SV=1	PDS5B_HUMAN (+1)	165 kDa	0.37	[]	0	0	0	0	0	2
Slingshot homolog 3 (Drosophila), isoform CRA_f OS=Homo sapiens GN=SSH3 PE=4 SV=1	A0A024R5J4_HUMAN (+1)	73 kDa	0.13	[]	0	0	0	1	0	2
Small nuclear ribonucleoprotein Sm D1 OS=Homo sapiens GN=SNRPD1 PE=1 SV=1	SMD1_HUMAN (+2)	13 kDa	0.37	[]	0	0	0	0	0	2
Sodium/calcium exchanger 1 OS=Homo sapiens GN=SLC8A1 PE=1 SV=3	NAC1_HUMAN (+1)	109 kDa	0.37	[]	0	0	3	0	0	0
Potassium-transporting ATPase alpha chain 2 OS=Homo sapiens GN=ATP12A PE=1 SV=3	AT12A_HUMAN	116 kDa	0.13	[]	0	0	0	0	2	4
Sodium/potassium-transporting ATPase subunit alpha OS=Homo sapiens PE=2 SV=1	B3KW93_HUMAN	101 kDa	0.086	[]	5	5	7	39	35	14
Sodium/potassium-transporting ATPase subunit alpha OS=Homo sapiens PE=2 SV=1	B7Z3F8_HUMAN	108 kDa	0.036	BRAC1 high, Naive low	0	0	0	40	39	14
Sodium/potassium-transporting ATPase subunit alpha-1 OS=Homo sapiens GN=ATP1A1 PE=1 SV=1	AT1A1_HUMAN	113 kDa	0.12	[]	21	26	31	121	108	53
Sodium/potassium-transporting ATPase subunit beta OS=Homo sapiens GN=ATP1B1 PE=2 SV=1	A3KLL5_HUMAN (+4)	35 kDa	0.029	BRAC1 high, Naive low	0	0	2	15	15	7
Sodium/potassium-transporting ATPase subunit beta-3 OS=Homo sapiens GN=ATP1B3 PE=1 SV=1	AT1B3_HUMAN	32 kDa	0.18	[]	4	4	5	6	6	5
Solute carrier family 12 member 4 OS=Homo sapiens GN=SLC12A4 PE=1 SV=2	S12A4_HUMAN (+2)	121 kDa	0.37	[]	0	0	2	0	0	0
Solute carrier family 12 member 7 OS=Homo sapiens GN=SLC12A7 PE=1 SV=1	A0A0G2JNW7_HUMAN (+1)	119 kDa	0.0037	BRAC1 high, Naive low	0	0	0	6	7	5
Solute carrier family 16 (Monocarboxylic acid transporters), member 1, isoform CRA_b OS=Homo sapiens GN=SLC16A1 PE=4 SV=1	A0A024R0H1_HUMAN (+2)	54 kDa	0.12	[]	0	1	4	9	6	5
cDNA FLJ53399, highly similar to Monocarboxylate transporter 1 OS=Homo sapiens PE=2 SV=1	B4E106_HUMAN	52 kDa	0.16	[]	0	1	4	10	5	5
Solute carrier family 16 (Monocarboxylic acid transporters), member 3, isoform CRA_a OS=Homo sapiens GN=SLC16A3 PE=4 SV=1	A0A024R8U1_HUMAN (+1)	49 kDa	0.58	[]	2	4	6	3	6	8
Solute carrier family 22 (Organic cation transporter), member 18, isoform CRA_a OS=Homo sapiens GN=SLC22A18 PE=1 SV=1	A0A024RCG2_HUMAN (+3)	45 kDa	0.19	[]	0	0	0	2	0	1
Solute carrier family 25 (Mitochondrial carrier phosphate carrier), member 3, isoform CRA_a OS=Homo sapiens GN=SLC25A3 PE=3 SV	A0A024RBE8_HUMAN (+3)	40 kDa	0.011	BRAC1 low, Naive high	4	3	5	2	1	3
Solute carrier family 27 (Fatty acid transporter), member 2 variant (Fragment) OS=Homo sapiens PE=2 SV=1	Q53GS2_HUMAN (+1)	70 kDa	0.14	[]	0	0	0	1	1	6
Solute carrier family 39 (Zinc transporter), member 7, isoform CRA_a OS=Homo sapiens GN=SLC39A7 PE=4 SV=1	A0A024RCX7_HUMAN (+4)	50 kDa	0.74	[]	1	1	1	1	1	5
Solute carrier family 44, member 1, isoform CRA_a OS=Homo sapiens GN=SLC44A1 PE=4 SV=1	A0A024R151_HUMAN (+1)	73 kDa	0.018	BRAC1 high, Naive low	0	0	3	6	7	7
Solute carrier family 44, member 2, isoform CRA_a OS=Homo sapiens GN=SLC44A2 PE=4 SV=1	A0A024R7D2_HUMAN	80 kDa	0.14	[]	1	0	3	4	5	4
Sorbitol dehydrogenase OS=Homo sapiens GN=SORD PE=1 SV=4	DHSO_HUMAN (+1)	38 kDa	0.026	BRAC1 high, Naive low	0	0	0	2	3	7
Sorting nexin OS=Homo sapiens GN=SNX9 PE=1 SV=1	A0A087WYU1_HUMAN (+4)	66 kDa	0.74	[]	1	2	0	2	2	0
Sorting nexin-5 OS=Homo sapiens GN=SNX5 PE=1 SV=1	SNX5_HUMAN	47 kDa	0.13	[]	0	0	0	0	1	2
Specifically androgen-regulated gene protein OS=Homo sapiens GN=SARG PE=1 SV=2	SARG_HUMAN	64 kDa	0.04	BRAC1 high, Naive low	0	0	0	2	3	1
Spectrin alpha chain, non-erythrocytic 1 OS=Homo sapiens GN=SPTAN1 PE=1 SV=3	SPTN1_HUMAN	285 kDa	0.031	BRAC1 high, Naive low	7	13	14	66	67	42
Spectrin beta chain, non-erythrocytic 1 OS=Homo sapiens GN=SPTBN1 PE=1 SV=1	A0A087WUZ3_HUMAN (+2)	275 kDa	0.02	BRAC1 high, Naive low	4	9	11	40	48	33
Spectrin beta chain, non-erythrocytic 2 OS=Homo sapiens GN=SPTBN2 PE=1 SV=3	SPTN2_HUMAN	271 kDa	0.12	[]	0	0	0	3	4	0
Spermatogenesis-associated protein 5-like protein 1 OS=Homo sapiens GN=SPATA5L1 PE=1 SV=2	SPA5L_HUMAN (+1)	81 kDa	0.37	[]	0	0	0	0	0	2
Sphingosine-1-phosphate lyase 1 OS=Homo sapiens GN=SGPL1 PE=1 SV=3	SGPL1_HUMAN	64 kDa	0.081	[]	0	0	0	1	4	9
Splicing factor 3B subunit 1 OS=Homo sapiens GN=SF3B1 PE=1 SV=3	SF3B1_HUMAN	146 kDa	0.37	[]	0	0	0	0	0	2
Splicing factor 3B subunit 3 OS=Homo sapiens GN=SF3B3 PE=1 SV=4	SF3B3_HUMAN (+2)	136 kDa	0.37	[]	0	0	0	0	0	2
Splicing factor U2AF 35 kDa subunit OS=Homo sapiens GN=U2AF1 PE=1 SV=3	U2AF1_HUMAN (+1)	28 kDa	0.13	[]	0	0	0	0	1	2
Splicing factor U2AF 65 kDa subunit OS=Homo sapiens GN=U2AF2 PE=1 SV=4	U2AF2_HUMAN (+1)	54 kDa	0.37	[]	0	0	0	0	0	2
Splicing factor proline/glutamine-rich (Polypyrimidine tract binding protein associated) OS=Homo sapiens GN=SFPQ PE=2 SV=1	Q86VG2_HUMAN (+2)	76 kDa	0.27	[]	1	2	7	1	0	5
Squalene monooxygenase OS=Homo sapiens GN=SQLE PE=1 SV=3	ERG1_HUMAN (+1)	64 kDa	0.25	[]	0	0	0	1	0	6
Squalene synthase OS=Homo sapiens GN=FDFT1 PE=1 SV=1	FDFT_HUMAN (+5)	48 kDa	0.13	[]	0	0	0	0	1	2
Staphylococcal nuclease domain-containing protein 1 OS=Homo sapiens GN=SND1 PE=1 SV=1	SND1_HUMAN	102 kDa	0.24	[]	15	16	15	18	19	28
Sterile alpha motif domain-containing protein 9 OS=Homo sapiens GN=SAMD9 PE=1 SV=1	SAMD9_HUMAN	184 kDa	0.21	[]	0	0	0	1	1	9
Sterol-4-alpha-carboxylate 3-dehydrogenase, decarboxylating OS=Homo sapiens GN=NSDHL PE=1 SV=2	NSDHL_HUMAN	42 kDa	0.2	[]	0	0	3	2	4	5
Stimulator of interferon genes protein OS=Homo sapiens GN=TMEM173 PE=1 SV=1	STING_HUMAN	42 kDa	0.0051	BRAC1 low, Naive high	2	2	2	0	0	1
Stomatin-like protein 2, mitochondrial OS=Homo sapiens GN=STOML2 PE=1 SV=1	STML2_HUMAN	39 kDa	0.98	[]	4	3	5	4	7	11
Stromal cell-derived factor 2 OS=Homo sapiens GN=SDF2 PE=1 SV=2	SDF2_HUMAN (+1)	23 kDa	0.37	[]	0	0	0	0	0	2
Stromal interaction molecule 1 OS=Homo sapiens GN=STIM1 PE=1 SV=3	STIM1_HUMAN (+2)	77 kDa	0.97	[]	0	2	1	0	0	6
Structural maintenance of chromosomes protein 3 OS=Homo sapiens GN=SMC3 PE=1 SV=2	SMC3_HUMAN (+2)	142 kDa	0.27	[]	0	0	0	0	1	7
Succinate dehydrogenase [ubiquinone] flavoprotein subunit, mitochondrial OS=Homo sapiens GN=SDHA PE=3 SV=1	A0A024QZ30_HUMAN (+1)	73 kDa	0.025	BRAC1 high, Naive low	3	2	3	8	7	10
cDNA FLJ37560 fis, clone BRCOC2000333, highly similar to Succinate dehydrogenase (ubiquinone) flavoprotein subunit, mitochondria	B3KT34_HUMAN	48 kDa	0.086	[]	3	2	1	7	7	8
Succinate dehydrogenase [ubiquinone] iron-sulfur subunit, mitochondrial OS=Homo sapiens GN=SDHB PE=1 SV=3	SDHB_HUMAN	32 kDa	0.86	[]	1	0	0	0	0	2
Succinyl-CoA ligase subunit beta OS=Homo sapiens GN=SUCLG2 PE=3 SV=1	A0A024R325_HUMAN (+1)	47 kDa	0.12	[]	3	2	2	2	2	0
Succinyl-CoA:3-ketoacid coenzyme A transferase 1, mitochondrial OS=Homo sapiens GN=OXCT1 PE=1 SV=1	SCOT1_HUMAN (+2)	56 kDa	0.17	[]	0	1	3	0	0	0
Sulfatase-modifying factor 2 OS=Homo sapiens GN=SUMF2 PE=1 SV=2	SUMF2_HUMAN (+3)	34 kDa	0.89	[]	0	1	1	0	1	2
Sulfhydryl oxidase OS=Homo sapiens GN=QSOX2 PE=1 SV=1	A0A087X176_HUMAN (+1)	77 kDa	0.036	BRAC1 high, Naive low	0	0	0	3	1	5
Sulfide:quinone oxidoreductase, mitochondrial OS=Homo sapiens GN=SQRDL PE=1 SV=1	SQRD_HUMAN (+4)	50 kDa	0.00091	BRAC1 low, Naive high	2	2	4	0	0	0
Sulfotransferase family cytosolic 2B member 1 OS=Homo sapiens GN=SULT2B1 PE=1 SV=2	ST2B1_HUMAN	41 kDa	0.15	[]	0	0	0	0	2	5

Sulfurtransferase OS=Homo sapiens PE=2 SV=1	B4DVZ4_HUMAN (+2)	28 kDa	0.1	[]	1	0	0	2	2	3
Superoxide dismutase [Mn], mitochondrial OS=Homo sapiens GN=SOD2 PE=1 SV=2	SODM_HUMAN (+2)	25 kDa	0.021	BRAC1 low, Naive high	7	4	6	0	0	1
Suppressor of tumorigenicity 14 protein homolog OS=Homo sapiens PE=2 SV=1	B2RAF9_HUMAN (+1)	95 kDa	0.0092	BRAC1 high, Naive low	0	0	0	10	9	6
Surfeit locus protein 4 OS=Homo sapiens GN=SURF4 PE=1 SV=3	SURF4_HUMAN (+2)	30 kDa	0.63	[]	1	1	8	0	1	9
tr|B7Z1G8|B7Z1G8_HUMAN Surfeit 4, isoform CRA_b OS=Homo sapiens GN=SURF4 PE=1 SV=1	B7Z1G8_HUMAN	?	0.37	[]	0	0	0	0	0	2
Sushi domain-containing protein 2 OS=Homo sapiens GN=SUSD2 PE=1 SV=1	SUSD2_HUMAN	90 kDa	0.37	[]	0	0	2	0	0	0
Synaptogyrin OS=Homo sapiens GN=SYNGR2 PE=3 SV=1	A0A024R8T9_HUMAN (+1)	25 kDa	0.89	[]	0	1	0	0	2	0
Synaptophysin-like protein 1 OS=Homo sapiens GN=SYPL1 PE=1 SV=1	C9JYN0_HUMAN	25 kDa	0.5	[]	1	2	2	1	2	4
Synaptosomal-associated protein OS=Homo sapiens GN=SNAP23 PE=1 SV=1	A8K287_HUMAN (+1)	23 kDa	0.052	[]	0	0	1	8	5	3
Syndecan binding protein (Syntenin), isoform CRA_a OS=Homo sapiens GN=SDCBP PE=1 SV=1	G5EA09_HUMAN	35 kDa	0.0049	BRAC1 high, Naive low	0	0	0	14	14	10
Syndecan binding protein (Syntenin), isoform CRA_c OS=Homo sapiens GN=SDCBP PE=4 SV=1	A0A024R7Z5_HUMAN (+1)	32 kDa	0.01	BRAC1 high, Naive low	0	0	0	16	17	10
Syndecan-1 OS=Homo sapiens GN=SDC1 PE=1 SV=3	SDC1_HUMAN	32 kDa	0.14	[]	0	0	0	3	5	0
Synembryn-A OS=Homo sapiens GN=RIC8A PE=1 SV=3	RIC8A_HUMAN	60 kDa	0.23	[]	0	0	0	0	1	5
Syntaxin 4A (Placental), isoform CRA_a OS=Homo sapiens GN=STX4A PE=2 SV=1	B7Z425_HUMAN (+1)	25 kDa	0.77	[]	0	1	3	1	4	2
Syntaxin-3 OS=Homo sapiens GN=STX3 PE=1 SV=3	STX3_HUMAN (+5)	33 kDa	0.37	[]	0	0	0	0	0	2
Syntaxin-7 OS=Homo sapiens GN=STX7 PE=1 SV=4	STX7_HUMAN	30 kDa	0.14	[]	3	4	2	3	3	1
Syntaxin-binding protein 1 OS=Homo sapiens GN=STXBP1 PE=1 SV=1	STXB1_HUMAN (+4)	68 kDa	0.12	[]	1	0	2	0	0	0
Syntaxin-binding protein 2 OS=Homo sapiens GN=STXBP2 PE=1 SV=1	A0A087WWS7_HUMAN (+4)	65 kDa	0.077	[]	0	1	1	2	2	4
Syntaxin-binding protein 3 OS=Homo sapiens GN=STXBP3 PE=1 SV=2	STXB3_HUMAN	68 kDa	0.65	[]	0	1	1	2	2	0
T-complex protein 1 subunit alpha OS=Homo sapiens GN=TCP1 PE=1 SV=1	TCPA_HUMAN	60 kDa	0.029	BRAC1 high, Naive low	3	3	6	10	9	17
T-complex protein 1 subunit beta OS=Homo sapiens GN=CCT2 PE=1 SV=4	TCPB_HUMAN (+1)	57 kDa	0.093	[]	6	4	11	15	16	19
T-complex protein 1 subunit delta OS=Homo sapiens PE=2 SV=1	B7Z9L0_HUMAN (+1)	52 kDa	0.23	[]	4	2	9	10	10	15
T-complex protein 1 subunit epsilon OS=Homo sapiens GN=CCT5 PE=1 SV=1	TCPE_HUMAN (+1)	60 kDa	0.12	[]	0	2	8	14	9	12
T-complex protein 1 subunit gamma OS=Homo sapiens PE=2 SV=1	B3KX11_HUMAN (+2)	58 kDa	0.33	[]	2	2	7	7	7	9
TAR DNA-binding protein 43 OS=Homo sapiens GN=TARDBP PE=1 SV=1	TADBP_HUMAN (+3)	45 kDa	0.87	[]	0	0	2	0	0	4
TBC1 domain family member 4 OS=Homo sapiens GN=TBC1D4 PE=1 SV=2	TBCD4_HUMAN (+1)	147 kDa	0.37	[]	0	0	0	0	0	2
TBC1 domain family member 10B OS=Homo sapiens GN=TBC1D10B PE=1 SV=3	TB10B_HUMAN	87 kDa	0.053	[]	0	0	0	4	4	1
TELO2-interacting protein 1 homolog OS=Homo sapiens GN=TTI1 PE=1 SV=3	TTI1_HUMAN (+1)	122 kDa	0.37	[]	0	0	0	0	0	2
TJP3 protein (Fragment) OS=Homo sapiens GN=TJP3 PE=2 SV=2	Q2VPE5_HUMAN (+2)	101 kDa	0.073	[]	0	0	0	2	4	1
TNF receptor-associated protein 1 variant (Fragment) OS=Homo sapiens PE=2 SV=1	Q53G55_HUMAN	80 kDa	0.24	[]	3	3	4	3	4	7
TRAP1 protein (Fragment) OS=Homo sapiens GN=TRAP1 PE=2 SV=2	Q9BV61_HUMAN	79 kDa	0.49	[]	3	3	4	4	5	7
TRIO and F-actin-binding protein OS=Homo sapiens GN=TRIOBP PE=1 SV=3	TARA_HUMAN	261 kDa	0.0036	BRAC1 low, Naive high	1	2	3	0	0	0
Talin-1 OS=Homo sapiens GN=TLN1 PE=1 SV=3	TLN1_HUMAN	270 kDa	0.004	BRAC1 low, Naive high	26	39	49	26	28	15
Tankyrase 1 binding protein 1, 182kDa, isoform CRA_a OS=Homo sapiens GN=TNKS1BP1 PE=4 SV=1	A0A024R542_HUMAN (+1)	182 kDa	0.076	[]	0	1	0	17	22	4
Tapasin OS=Homo sapiens GN=TAPBP PE=1 SV=1	TPSN_HUMAN (+7)	48 kDa	0.86	[]	0	1	1	0	0	3
Tenascin OS=Homo sapiens GN=TNC PE=1 SV=3	TENA_HUMAN (+5)	241 kDa	0.12	[]	4	5	0	0	0	0
Tensin-1 OS=Homo sapiens GN=TNS1 PE=1 SV=2	TENS1_HUMAN (+6)	186 kDa	0.12	[]	2	2	0	0	0	0
Testis derived transcript (3 LIM domains) OS=Homo sapiens GN=TES PE=4 SV=1	A4D0U5_HUMAN (+4)	48 kDa	0.07	[]	0	0	0	1	1	4
Tetraspanin (Fragment) OS=Homo sapiens GN=TSPAN14 PE=1 SV=2	A6NEP9_HUMAN (+1)	25 kDa	0.055	[]	0	0	0	4	3	1
Tetraspanin (Fragment) OS=Homo sapiens PE=2 SV=1	Q53GA9_HUMAN (+1)	26 kDa	0.0011	BRAC1 high, Naive low	0	0	0	9	7	8
Tetraspanin OS=Homo sapiens GN=CD9 PE=1 SV=1	A6NNI4_HUMAN (+3)	18 kDa	0.65	[]	1	1	2	4	4	0
Tetraspanin OS=Homo sapiens GN=CD81 PE=3 SV=1	A0A024RCB7_HUMAN (+7)	26 kDa	0.15	[]	5	4	5	5	6	2
Tetraspanin OS=Homo sapiens GN=CD151 PE=3 SV=1	A0A024RCB3_HUMAN (+1)	28 kDa	0.13	[]	0	0	0	0	1	2
Tetraspanin-15 (Fragment) OS=Homo sapiens GN=TSPAN15 PE=1 SV=1	H7C285_HUMAN (+1)	18 kDa	0.0068	BRAC1 high, Naive low	0	0	0	3	3	2
Tetratricopeptide repeat protein 37 OS=Homo sapiens GN=TTC37 PE=1 SV=1	TTC37_HUMAN	175 kDa	0.37	[]	0	0	0	0	0	5
Thioredoxin domain-containing protein 5 OS=Homo sapiens GN=TXNDC5 PE=1 SV=2	TXND5_HUMAN	48 kDa	0.0094	BRAC1 low, Naive high	4	5	6	2	1	6
Thioredoxin interacting protein, isoform CRA_a OS=Homo sapiens GN=TXNIP PE=4 SV=1	A0A024R4B7_HUMAN (+3)	44 kDa	0.37	[]	0	0	0	2	0	0
Thioredoxin reductase 1, cytoplasmic OS=Homo sapiens GN=TXNRD1 PE=1 SV=3	TRXR1_HUMAN (+8)	71 kDa	0.19	[]	0	3	7	0	0	2
Thioredoxin-dependent peroxide reductase, mitochondrial OS=Homo sapiens GN=PRDX3 PE=1 SV=3	PRDX3_HUMAN	28 kDa	0.85	[]	1	2	4	3	3	6
Thioredoxin-related transmembrane protein 1 OS=Homo sapiens GN=TMX1 PE=1 SV=1	TMX1_HUMAN	32 kDa	0.082	[]	1	1	2	0	1	2
Thioredoxin-related transmembrane protein 2 OS=Homo sapiens GN=TMX2 PE=1 SV=1	TMX2_HUMAN	34 kDa	0.0003	BRAC1 high, Naive low	0	0	0	3	3	5
Thrombospondin-1 OS=Homo sapiens GN=THBS1 PE=1 SV=2	TSP1_HUMAN (+1)	129 kDa	0.0008	BRAC1 low, Naive high	10	16	15	0	0	0
Thy-1 membrane glycoprotein OS=Homo sapiens GN=THY1 PE=1 SV=2	THY1_HUMAN (+4)	18 kDa	0.0013	BRAC1 low, Naive high	9	11	10	0	0	0
Tight junction protein 2 (Zona occludens 2), isoform CRA_a OS=Homo sapiens GN=TJP2 PE=4 SV=1	A0A024R233_HUMAN	134 kDa	0.0028	BRAC1 high, Naive low	0	0	0	13	14	11
Tight junction protein ZO-1 (Fragment) OS=Homo sapiens GN=TJP1 PE=2 SV=1	A9CQZ8_HUMAN (+2)	197 kDa	0.076	[]	1	1	6	19	17	8
Tight junction protein ZO-1 OS=Homo sapiens GN=TJP1 PE=1 SV=1	A0A087X0K9_HUMAN (+1)	188 kDa	0.076	[]	1	1	6	19	17	8
Tissue alpha-L-fucosidase OS=Homo sapiens GN=FUCA1 PE=1 SV=4	FUCO_HUMAN	54 kDa	0.23	[]	0	0	0	0	1	5
Tissue factor OS=Homo sapiens GN=F3 PE=1 SV=1	TF_HUMAN (+2)	33 kDa	0.025	BRAC1 low, Naive high	1	3	2	0	0	0
Toll-interacting protein OS=Homo sapiens GN=TOLLIP PE=1 SV=1	F2Z2Y8_HUMAN (+4)	23 kDa	0.018	BRAC1 high, Naive low	0	0	0	2	2	1
Torsin-1A-interacting protein 1 OS=Homo sapiens GN=TOR1AIP1 PE=1 SV=1	A0A0A0MSK5_HUMAN (+2)	52 kDa	0.82	[]	0	1	2	1	0	3
Torsin-1A-interacting protein 2 OS=Homo sapiens GN=TOR1AIP2 PE=1 SV=1	TOIP2_HUMAN (+1)	51 kDa	0.12	[]	1	0	2	0	0	0
Trans-Golgi network integral membrane protein 2 OS=Homo sapiens GN=TGOLN2 PE=1 SV=2	F8W8W7_HUMAN (+2)	51 kDa	0.05	[]	0	2	2	5	4	7

Transaldolase OS=Homo sapiens GN=TALDO1 PE=1 SV=1	F2Z393_HUMAN (+1)	35 kDa	0.086	[]	0	0	1	1	2	2
Transcription factor p65 OS=Homo sapiens GN=RELA PE=1 SV=2	TF65_HUMAN (+4)	60 kDa	0.13	[]	0	0	0	0	1	2
Transcriptional activator protein Pur-alpha OS=Homo sapiens GN=PURA PE=1 SV=2	PURA_HUMAN (+4)	35 kDa	0.072	[]	3	2	2	1	1	2
Transducin beta-like protein 2 OS=Homo sapiens GN=TBL2 PE=1 SV=1	TBL2_HUMAN (+4)	50 kDa	0.67	[]	1	3	1	0	1	5
Transgelin OS=Homo sapiens GN=TAGLN PE=1 SV=4	TAGL_HUMAN (+1)	23 kDa	0.034	BRAC1 low, Naive high	1	2	5	0	0	0
Transgelin-2 OS=Homo sapiens GN=TAGLN2 PE=1 SV=3	TAGL2_HUMAN (+1)	22 kDa	0.063	[]	2	4	4	15	16	9
Transient receptor potential cation channel subfamily M member 4 OS=Homo sapiens GN=TRPM4 PE=1 SV=1	TRPM4_HUMAN	134 kDa	0.0058	BRAC1 high, Naive low	0	0	0	1	2	2
Transitional endoplasmic reticulum ATPase OS=Homo sapiens GN=VCP PE=1 SV=4	TERA_HUMAN (+1)	89 kDa	0.0023	BRAC1 low, Naive high	9	11	17	11	13	15
Translation initiation factor eIF-2B subunit delta OS=Homo sapiens GN=EIF2B4 PE=1 SV=2	EI2BD_HUMAN (+4)	58 kDa	0.37	[]	0	0	0	0	0	2
Translocase of inner mitochondrial membrane 50 homolog (Yeast), isoform CRA_b OS=Homo sapiens GN=TIMM50 PE=4 SV=1	A0A024R0M6_HUMAN (+2)	50 kDa	0.017	BRAC1 high, Naive low	0	0	0	4	3	2
Translocation protein SEC62 OS=Homo sapiens GN=SEC62 PE=1 SV=1	SEC62_HUMAN	46 kDa	0.37	[]	0	0	0	0	0	5
Translocon-associated protein subunit delta OS=Homo sapiens GN=SSR4 PE=1 SV=1	SSRD_HUMAN	19 kDa	0.041	BRAC1 low, Naive high	2	2	3	2	3	2
Transmembrane BAX inhibitor motif containing 1, isoform CRA_a OS=Homo sapiens GN=TMBIM1 PE=4 SV=1	A0A024R472_HUMAN (+9)	35 kDa	0.67	[]	1	0	2	3	2	1
Transmembrane and coiled-coil domains 1, isoform CRA_a OS=Homo sapiens GN=TMCO1 PE=2 SV=1	B7Z591_HUMAN (+3)	20 kDa	0.031	BRAC1 high, Naive low	0	0	0	2	1	1
Transmembrane emp24 domain-containing protein 7 OS=Homo sapiens GN=TMED7 PE=1 SV=2	TMED7_HUMAN	25 kDa	0.72	[]	0	2	2	0	1	4
Transmembrane emp24-like trafficking protein 10 (Yeast), isoform CRA_a OS=Homo sapiens GN=TMED10 PE=4 SV=1	A0A024R6I3_HUMAN (+2)	25 kDa	0.58	[]	0	2	1	1	0	2
Transmembrane protein 2, isoform CRA_b OS=Homo sapiens GN=TMEM2 PE=4 SV=1	A0A024R229_HUMAN (+1)	154 kDa	0.12	[]	0	0	0	4	4	0
Transmembrane protein 43, isoform CRA_a OS=Homo sapiens GN=TMEM43 PE=4 SV=1	A0A024R2F9_HUMAN (+1)	45 kDa	0.01	BRAC1 low, Naive high	6	6	5	1	1	2
Transmembrane protein 51, isoform CRA_b OS=Homo sapiens GN=TMEM51 PE=4 SV=1	A0A024QZ97_HUMAN (+1)	28 kDa	0.0014	BRAC1 high, Naive low	0	0	0	3	2	3
Transmembrane protein 119 OS=Homo sapiens GN=TMEM119 PE=1 SV=1	TM119_HUMAN (+1)	29 kDa	0.014	BRAC1 low, Naive high	1	2	4	0	0	0
Transmembrane protein 165 OS=Homo sapiens GN=TMEM165 PE=1 SV=1	TM165_HUMAN	35 kDa	0.038	BRAC1 low, Naive high	2	3	2	2	1	1
Transmembrane protein 214 OS=Homo sapiens GN=TMEM214 PE=1 SV=2	TM214_HUMAN (+1)	77 kDa	0.5	[]	0	2	0	0	0	1
Transmembrane protein 238 OS=Homo sapiens GN=TMEM238 PE=1 SV=1	TM238_HUMAN	18 kDa	0.19	[]	0	0	0	2	0	1
Transporter 2, ATP-binding cassette, sub-family B isoform 1 variant (Fragment) OS=Homo sapiens PE=2 SV=1	Q59H06_HUMAN (+1)	78 kDa	0.0078	BRAC1 low, Naive high	1	2	3	0	0	1
Transporter OS=Homo sapiens GN=SLC6A6 PE=3 SV=1	A0A024R2N0_HUMAN (+5)	70 kDa	0.16	[]	0	0	0	1	2	0
Transportin 3, isoform CRA_b OS=Homo sapiens GN=TNPO3 PE=4 SV=1	A0A024R794_HUMAN (+2)	104 kDa	0.23	[]	0	0	0	2	0	10
Transportin-1 OS=Homo sapiens GN=TNPO1 PE=1 SV=2	TNPO1_HUMAN (+1)	102 kDa	0.27	[]	1	1	2	0	1	3
tr|A0A024R7F7|A0A024R7F7_HUMAN Transportin 2 (Importin 3, karyopherin beta 2b), isoform...	A0A024R7F7_HUMAN (+2)	?	0.37	[]	0	0	0	0	0	2
Trifunctional enzyme subunit beta, mitochondrial OS=Homo sapiens GN=HADHB PE=1 SV=3	ECHB_HUMAN	51 kDa	0.24	[]	1	1	7	0	0	3
Trifunctional purine biosynthetic protein adenosine-3 OS=Homo sapiens GN=GART PE=1 SV=1	PUR2_HUMAN (+2)	108 kDa	0.31	[]	0	0	1	1	1	16
Trinucleotide repeat containing 5, isoform CRA_g OS=Homo sapiens GN=TNRC5 PE=4 SV=1	A0A024RCZ2_HUMAN (+2)	17 kDa	0.13	[]	0	0	0	1	0	2
Triosephosphate isomerase (Fragment) OS=Homo sapiens PE=2 SV=1	Q53HE2_HUMAN (+2)	27 kDa	0.2	[]	4	2	8	11	9	13
Tripartite motif-containing 29, isoform CRA_a OS=Homo sapiens GN=TRIM29 PE=4 SV=1	A0A024R3J1_HUMAN (+2)	66 kDa	0.042	BRAC1 high, Naive low	0	0	0	3	2	1
Tripeptidyl-peptidase 1 OS=Homo sapiens GN=TPP1 PE=1 SV=1	A0A0C4DGZ9_HUMAN (+5)	60 kDa	0.011	BRAC1 low, Naive high	6	5	6	1	1	4
Tripeptidyl-peptidase 2 OS=Homo sapiens GN=TPP2 PE=1 SV=4	TPP2_HUMAN (+2)	138 kDa	0.55	[]	0	0	2	0	0	1
Trophoblast glycoprotein OS=Homo sapiens GN=TPBG PE=1 SV=1	TPBG_HUMAN	46 kDa	0.018	BRAC1 low, Naive high	3	5	3	1	1	1
Epididymis secretory protein Li 265 OS=Homo sapiens GN=HEL-S-265 PE=2 SV=1	A0A0K0K1I0_HUMAN (+3)	33 kDa	0.00054	BRAC1 low, Naive high	11	12	13	0	0	0
Epididymis secretory protein Li 273 OS=Homo sapiens GN=HEL-S-273 PE=2 SV=1	V9HW25_HUMAN	33 kDa	0.0036	BRAC1 low, Naive high	14	14	13	0	0	0
Tropomyosin 1 (Alpha), isoform CRA_a OS=Homo sapiens GN=TPM1 PE=3 SV=1	A0A024R5W6_HUMAN	33 kDa	0.0008	BRAC1 low, Naive high	7	8	8	0	0	0
Tropomyosin alpha-1 chain OS=Homo sapiens GN=TPM1 PE=1 SV=1	B7Z596_HUMAN (+6)	32 kDa	0.072	[]	7	8	4	5	3	0
cDNA FLJ57036, highly similar to Homo sapiens tropomyosin 2 (beta) (TPM2), transcript variant 2, mRNA OS=Homo sapiens PE=2 SV=	B4E3P1_HUMAN	34 kDa	< 0.00010	BRAC1 low, Naive high	6	8	9	0	0	0
Tropomyosin alpha-4 chain OS=Homo sapiens GN=TPM4 PE=1 SV=3	TPM4_HUMAN (+1)	29 kDa	0.014	BRAC1 low, Naive high	13	13	11	5	5	2
Truncated nucleolar phosphoprotein B23 OS=Homo sapiens GN=NPM1 PE=2 SV=1	A4ZU86_HUMAN (+1)	30 kDa	0.35	[]	0	0	2	3	1	2
Trypsin-2 OS=Homo sapiens GN=PRSS2 PE=1 SV=1	TRY2_HUMAN (+10)	26 kDa	0.37	[]	0	0	0	0	0	2
Tryptophan--tRNA ligase, cytoplasmic OS=Homo sapiens GN=WARS PE=1 SV=2	SYWC_HUMAN (+1)	53 kDa	0.38	[]	0	0	1	0	1	5
Tubulin alpha-1A chain OS=Homo sapiens GN=TUBA1A PE=1 SV=1	TBA1A_HUMAN	50 kDa	0.69	[]	26	28	36	38	33	75
Tubulin alpha-4A chain OS=Homo sapiens GN=TUBA4A PE=1 SV=1	TBA4A_HUMAN	50 kDa	0.17	[]	33	36	32	67	62	117
cDNA FLJ55956, highly similar to Tubulin alpha-6 chain OS=Homo sapiens PE=2 SV=1	B7Z1K5_HUMAN (+3)	58 kDa	0.19	[]	37	40	36	71	70	128
Tubulin gamma-1 chain OS=Homo sapiens GN=TUBG1 PE=1 SV=2	TBG1_HUMAN (+1)	51 kDa	0.37	[]	0	0	0	0	0	2
Tubulin polymerization-promoting protein family member 3 OS=Homo sapiens GN=TPPP3 PE=1 SV=1	TPPP3_HUMAN (+1)	19 kDa	0.37	[]	0	0	0	0	2	0
Tubulin--tyrosine ligase-like protein 12 OS=Homo sapiens GN=TTLL12 PE=1 SV=2	TTL12_HUMAN (+1)	74 kDa	0.37	[]	0	0	0	0	0	5
Tubulin-specific chaperone D OS=Homo sapiens GN=TBCD PE=1 SV=2	TBCD_HUMAN (+1)	133 kDa	0.37	[]	0	0	0	0	0	3
Tumor necrosis factor alpha-induced protein 2 OS=Homo sapiens GN=TNFAIP2 PE=2 SV=2	TNAP2_HUMAN	73 kDa	0.0038	BRAC1 high, Naive low	0	0	0	3	4	7
Tumor protein D54 OS=Homo sapiens GN=TPD52L2 PE=1 SV=1	A0A087WYR3_HUMAN (+2)	24 kDa	0.35	[]	1	1	1	1	3	4
Tumor susceptibility gene 101 protein OS=Homo sapiens GN=TSG101 PE=1 SV=1	F5H442_HUMAN (+1)	41 kDa	0.19	[]	0	0	1	1	2	1
Tumor-associated calcium signal transducer 2 OS=Homo sapiens GN=TACSTD2 PE=1 SV=3	TACD2_HUMAN	36 kDa	0.16	[]	0	0	0	2	1	0
Twinfilin-1 OS=Homo sapiens GN=TWF1 PE=1 SV=3	TWF1_HUMAN	40 kDa	0.37	[]	0	0	2	0	0	0
Twinfilin-2 OS=Homo sapiens GN=TWF2 PE=1 SV=1	D6RG15_HUMAN (+1)	29 kDa	0.21	[]	0	0	2	1	3	4
Type I inositol 1,4,5-trisphosphate 5-phosphatase OS=Homo sapiens GN=INPP5A PE=1 SV=1	I5P1_HUMAN (+2)	48 kDa	0.48	[]	0	0	3	0	0	1
Tyrosine--tRNA ligase, cytoplasmic OS=Homo sapiens GN=YARS PE=1 SV=4	SYYC_HUMAN	59 kDa	0.094	[]	0	0	0	1	4	10
Tyrosine--tRNA ligase, mitochondrial OS=Homo sapiens GN=YARS2 PE=1 SV=2	SYYM_HUMAN (+1)	53 kDa	0.37	[]	0	0	0	0	0	2
Tyrosine-protein kinase CSK OS=Homo sapiens GN=CSK PE=1 SV=1	CSK_HUMAN (+5)	51 kDa	0.13	[]	0	0	0	0	1	2

Tyrosine-protein kinase OS=Homo sapiens GN=JAK1 PE=2 SV=1	A0A0A0N0M2_HUMAN (+1)	133 kDa	0.16	[]	0	0	0	1	2	0
Tyrosine-protein kinase OS=Homo sapiens PE=2 SV=1	B2RA70_HUMAN (+2)	61 kDa	0.056	[]	0	2	0	4	4	5
Tyrosine-protein kinase transmembrane receptor OS=Homo sapiens GN=ROR1 PE=1 SV=1	A0A0A0MTJ5_HUMAN (+2)	98 kDa	0.13	[]	0	0	0	3	3	0
Tyrosine-protein phosphatase non-receptor type 6 OS=Homo sapiens GN=PTPN6 PE=1 SV=1	PTN6_HUMAN (+8)	68 kDa	0.37	[]	0	0	0	0	0	2
Tyrosine-protein phosphatase non-receptor type substrate 1 OS=Homo sapiens GN=SIRPA PE=1 SV=2	SHPS1_HUMAN (+2)	55 kDa	0.13	[]	0	1	2	0	0	0
UBX domain-containing protein 6 OS=Homo sapiens GN=UBXN6 PE=1 SV=1	UBXN6_HUMAN (+1)	50 kDa	0.37	[]	0	0	0	0	0	2
UDP-N-acetylglucosamine--peptide N-acetylglucosaminyltransferase 110 kDa subunit OS=Homo sapiens GN=OGT PE=1 SV=3	OGT1_HUMAN	117 kDa	0.37	[]	0	0	0	0	0	2
UDP-glucose 4-epimerase OS=Homo sapiens GN=GALE PE=1 SV=2	GALE_HUMAN	38 kDa	0.1	[]	0	0	0	2	1	7
UDP-glucose 6-dehydrogenase OS=Homo sapiens GN=UGDH PE=1 SV=1	UGDH_HUMAN	55 kDa	0.56	[]	2	3	6	5	4	16
UDP-glucose:glycoprotein glucosyltransferase 1 OS=Homo sapiens GN=UGGT1 PE=1 SV=3	UGGG1_HUMAN	177 kDa	0.93	[]	2	5	7	1	7	15
UDP-glucuronosyltransferase OS=Homo sapiens GN=UGT1A8 PE=2 SV=1	Q5DSZ6_HUMAN (+1)	60 kDa	0.19	[]	0	0	0	0	5	17
UDP-glucuronosyltransferase OS=Homo sapiens GN=UGT1A9 PE=2 SV=1	Q5DSZ5_HUMAN (+1)	60 kDa	0.15	[]	0	0	0	0	5	12
UDP-glucuronosyltransferase OS=Homo sapiens GN=UGT1A10 PE=2 SV=1	Q5DT02_HUMAN (+1)	60 kDa	0.078	[]	0	0	0	5	6	23
UMP-CMP kinase OS=Homo sapiens GN=CMPK1 PE=1 SV=3	KCY_HUMAN (+2)	22 kDa	0.21	[]	0	0	0	0	1	4
UPF1 regulator of nonsense transcripts homolog (Yeast), isoform CRA_b OS=Homo sapiens GN=UPF1 PE=4 SV=1	A0A024R7L5_HUMAN (+2)	123 kDa	0.3	[]	0	0	0	1	0	10
UPF0568 protein C14orf166 OS=Homo sapiens GN=C14orf166 PE=1 SV=1	CN166_HUMAN (+2)	28 kDa	0.31	[]	1	0	3	0	0	2
UTP--glucose-1-phosphate uridylyltransferase OS=Homo sapiens GN=UGP2 PE=1 SV=1	A0A087WYS1_HUMAN (+4)	57 kDa	0.44	[]	0	1	8	0	0	4
Ubiquitin carboxyl-terminal hydrolase 5 OS=Homo sapiens GN=USP5 PE=1 SV=2	UBP5_HUMAN	96 kDa	0.1	[]	1	1	1	4	3	11
Ubiquitin carboxyl-terminal hydrolase 7 OS=Homo sapiens GN=USP7 PE=1 SV=2	UBP7_HUMAN (+3)	128 kDa	0.37	[]	0	0	0	0	0	2
Ubiquitin carboxyl-terminal hydrolase 8 OS=Homo sapiens GN=USP8 PE=1 SV=1	UBP8_HUMAN (+3)	128 kDa	0.37	[]	0	0	0	0	0	2
Ubiquitin carboxyl-terminal hydrolase 46 OS=Homo sapiens GN=USP46 PE=1 SV=1	H7BZK6_HUMAN (+2)	40 kDa	0.16	[]	0	0	0	1	2	0
Ubiquitin carboxyl-terminal hydrolase isozyme L3 OS=Homo sapiens GN=UCHL3 PE=1 SV=1	UCHL3_HUMAN	26 kDa	0.11	[]	0	0	0	1	1	5
Ubiquitin carboxyl-terminal hydrolase isozyme L5 OS=Homo sapiens GN=UCHL5 PE=1 SV=3	UCHL5_HUMAN (+3)	38 kDa	0.37	[]	0	0	0	0	0	2
Ubiquitin conjugation factor E4 A OS=Homo sapiens GN=UBE4A PE=1 SV=2	UBE4A_HUMAN (+1)	123 kDa	0.37	[]	0	0	0	0	0	3
Ubiquitin specific peptidase 14 (TRNA-guanine transglycosylase), isoform CRA_a OS=Homo sapiens GN=USP14 PE=4 SV=1	D3DUG9_HUMAN (+1)	53 kDa	0.19	[]	0	0	2	2	2	9
Ubiquitin-activating enzyme E1 (A1S9T and BN75 temperature sensitivity complementing), isoform CRA_a OS=Homo sapiens GN=UBE	A0A024R1A3_HUMAN (+1)	118 kDa	0.31	[]	3	6	12	11	12	33
Ubiquitin-conjugating enzyme E2 N OS=Homo sapiens GN=UBE2N PE=1 SV=1	UBE2N_HUMAN (+2)	17 kDa	0.12	[]	0	0	0	2	2	0
Ubiquitin-like 3, isoform CRA_a OS=Homo sapiens GN=UBL3 PE=4 SV=1	A0A024RDP0_HUMAN (+1)	13 kDa	0.16	[]	0	0	0	2	1	0
Ubiquitin-like modifier-activating enzyme 6 OS=Homo sapiens GN=UBA6 PE=1 SV=1	UBA6_HUMAN (+2)	118 kDa	0.37	[]	0	0	0	0	0	7
Ubiquitin-like protein ISG15 (Fragment) OS=Homo sapiens GN=ISG15 PE=1 SV=4	A0A096LNZ9_HUMAN (+2)	16 kDa	0.96	[]	1	0	5	3	3	3
Ufm1-specific protease 2 OS=Homo sapiens GN=UFSP2 PE=1 SV=3	UFSP2_HUMAN (+1)	53 kDa	0.37	[]	0	0	0	0	2	0
Uncharacterized protein (Fragment) OS=Homo sapiens PE=1 SV=1	F8W031_HUMAN	29 kDa	0.94	[]	1	1	0	2	1	1
Uncharacterized protein KIAA1522 OS=Homo sapiens GN=KIAA1522 PE=1 SV=2	K1522_HUMAN	107 kDa	0.02	BRAC1 high, Naive low	0	0	0	9	11	5
Uncharacterized protein OS=Homo sapiens PE=2 SV=1	B4DLN1_HUMAN	48 kDa	0.18	[]	1	0	0	3	1	3
Uncharacterized protein OS=Homo sapiens PE=2 SV=1	B7ZL84_HUMAN (+3)	69 kDa	0.13	[]	0	0	0	3	2	0
Myosin IC, isoform CRA_a OS=Homo sapiens GN=MYO1C PE=4 SV=1	D3DTH7_HUMAN	99 kDa	0.13	[]	20	25	26	62	68	49
Unconventional myosin-Ic OS=Homo sapiens GN=MYO1C PE=1 SV=4	MYO1C_HUMAN	122 kDa	0.074	[]	29	37	37	108	110	81
Unconventional myosin-Id OS=Homo sapiens GN=MYO1D PE=1 SV=2	MYO1D_HUMAN	116 kDa	0.049	BRAC1 high, Naive low	1	1	2	26	24	9
Unconventional myosin-Ie OS=Homo sapiens GN=MYO1E PE=1 SV=2	MYO1E_HUMAN (+1)	127 kDa	0.0027	BRAC1 high, Naive low	0	0	0	1	1	2
Unconventional myosin-VI OS=Homo sapiens GN=MYO6 PE=1 SV=1	A0A0A0MRM8_HUMAN	145 kDa	0.002	BRAC1 high, Naive low	0	0	2	19	16	32
Uridine 5'-monophosphate synthase OS=Homo sapiens GN=UMPS PE=1 SV=1	UMPS_HUMAN (+2)	52 kDa	0.37	[]	0	0	0	0	0	4
Utrophin OS=Homo sapiens GN=UTRN PE=1 SV=2	UTRO_HUMAN	394 kDa	0.35	[]	7	9	1	32	26	4
Uveal autoantigen with coiled-coil domains and ankyrin repeats OS=Homo sapiens GN=UACA PE=2 SV=1	B7ZKM6_HUMAN (+2)	163 kDa	0.49	[]	1	1	1	2	1	1
V-ral simian leukemia viral oncogene homolog B (Ras related GTP binding protein), isoform CRA_a OS=Homo sapiens GN=RALB PE=4 S	A0A024RAG3_HUMAN (+2)	23 kDa	0.027	BRAC1 high, Naive low	0	1	4	10	11	8
V-set and immunoglobulin domain-containing protein 2 OS=Homo sapiens GN=VSIG2 PE=1 SV=1	VSIG2_HUMAN	34 kDa	0.14	[]	0	0	0	3	5	0
V-set and immunoglobulin domain-containing protein 10-like OS=Homo sapiens GN=VSIG10L PE=2 SV=2	VS10L_HUMAN	92 kDa	0.0003	BRAC1 high, Naive low	0	0	0	3	3	5
V-type proton ATPase catalytic subunit A OS=Homo sapiens GN=ATP6V1A PE=1 SV=2	VATA_HUMAN	68 kDa	0.51	[]	0	2	4	0	0	5
V-type proton ATPase subunit B, brain isoform OS=Homo sapiens GN=ATP6V1B2 PE=1 SV=3	VATB2_HUMAN	57 kDa	0.092	[]	2	1	1	0	0	1
V-type proton ATPase subunit E 1 (Fragment) OS=Homo sapiens GN=ATP6V1E1 PE=1 SV=1	C9J8H1_HUMAN (+2)	24 kDa	0.46	[]	2	0	2	1	1	2
V-type proton ATPase subunit H OS=Homo sapiens GN=ATP6V1H PE=1 SV=1	VATH_HUMAN (+5)	56 kDa	0.35	[]	0	1	3	0	0	2
V-type proton ATPase subunit a OS=Homo sapiens GN=TCIRG1 PE=3 SV=1	A0A024R5E5_HUMAN (+1)	93 kDa	0.0038	BRAC1 low, Naive high	3	4	3	0	0	0
VAMP3 protein OS=Homo sapiens GN=VAMP3 PE=2 SV=1	Q6FGG2_HUMAN (+1)	11 kDa	0.13	[]	3	4	1	2	1	1
VAMP (Vesicle-associated membrane protein)-associated protein B and C OS=Homo sapiens GN=VAPB PE=1 SV=1	Q53XM7_HUMAN (+1)	27 kDa	0.62	[]	0	1	2	1	2	3
Vacuolar protein sorting 4A (Yeast), isoform CRA_c OS=Homo sapiens GN=VPS4A PE=3 SV=1	A0A024R705_HUMAN (+1)	49 kDa	0.17	[]	0	0	0	1	0	3
Vacuolar protein sorting 4B (Yeast), isoform CRA_a OS=Homo sapiens GN=VPS4B PE=3 SV=1	A0A024R2C5_HUMAN (+3)	49 kDa	0.17	[]	0	0	0	1	0	3
Vacuolar protein sorting-associated protein 26A OS=Homo sapiens GN=VPS26A PE=1 SV=2	VP26A_HUMAN	38 kDa	0.67	[]	0	0	2	1	1	2
Vacuolar protein sorting-associated protein 35 OS=Homo sapiens GN=VPS35 PE=1 SV=2	VPS35_HUMAN	92 kDa	0.62	[]	0	3	7	3	4	16
Vacuolar protein sorting-associated protein 37B OS=Homo sapiens GN=VPS37B PE=1 SV=1	VP37B_HUMAN (+2)	31 kDa	0.19	[]	0	0	0	0	4	2
Vacuolar protein sorting-associated protein 45 OS=Homo sapiens GN=VPS45 PE=1 SV=1	A0A087WU65_HUMAN (+3)	51 kDa	0.0027	BRAC1 high, Naive low	0	0	0	1	1	2
Vacuolar protein sorting-associated protein 51 homolog OS=Homo sapiens GN=VPS51 PE=1 SV=2	VPS51_HUMAN	86 kDa	0.13	[]	0	0	0	0	1	2
Valyl-tRNA synthetase, isoform CRA_a OS=Homo sapiens GN=VARS PE=3 SV=1	A0A024RCN6_HUMAN (+1)	140 kDa	0.059	[]	0	1	0	5	4	15
Vasodilator-stimulated phosphoprotein, isoform CRA_a OS=Homo sapiens GN=VASP PE=4 SV=1	A0A024R0V4_HUMAN (+1)	40 kDa	0.038	BRAC1 high, Naive low	3	3	7	24	26	15

Vasorin OS=Homo sapiens GN=VASN PE=1 SV=1	VASN_HUMAN	72 kDa	0.12	[]	1	0	2	0	0	0
Very long-chain specific acyl-CoA dehydrogenase, mitochondrial OS=Homo sapiens GN=ACADVL PE=1 SV=1	ACADV_HUMAN (+1)	70 kDa	0.032	BRAC1 low, Naive high	10	12	11	8	5	15
Very-long-chain (3R)-3-hydroxyacyl-CoA dehydratase 3 OS=Homo sapiens GN=HACD3 PE=1 SV=2	HACD3_HUMAN	43 kDa	0.18	[]	0	1	3	3	5	4
Very-long-chain enoyl-CoA reductase OS=Homo sapiens GN=TECR PE=1 SV=1	TECR_HUMAN (+2)	36 kDa	0.5	[]	0	0	1	0	2	1
Vesicle amine transport protein 1 homolog (T californica), isoform CRA_a OS=Homo sapiens GN=VAT1 PE=4 SV=1	A0A024R1Z6_HUMAN (+1)	42 kDa	0.021	BRAC1 low, Naive high	6	8	5	2	1	4
Vesicle docking protein p115, isoform CRA_a OS=Homo sapiens GN=VDP PE=4 SV=1	A0A024RDG1_HUMAN (+1)	108 kDa	0.76	[]	1	1	2	1	0	5
Vesicle transport protein GOT1B OS=Homo sapiens GN=GOLT1B PE=1 SV=1	GOT1B_HUMAN (+2)	15 kDa	0.19	[]	0	0	0	0	2	1
Vesicle-trafficking protein SEC22b OS=Homo sapiens GN=SEC22B PE=1 SV=4	SC22B_HUMAN	25 kDa	0.046	BRAC1 low, Naive high	1	1	2	1	1	2
Villin 1 variant (Fragment) OS=Homo sapiens PE=2 SV=1	Q53F91_HUMAN (+1)	93 kDa	0.046	BRAC1 high, Naive low	0	0	0	27	23	8
Vinculin, isoform CRA_c OS=Homo sapiens GN=VCL PE=4 SV=1	A0A024QZN4_HUMAN (+2)	117 kDa	0.0074	BRAC1 low, Naive high	15	12	18	8	6	4
Voltage-dependent anion channel 2, isoform CRA_a OS=Homo sapiens GN=VDAC2 PE=4 SV=1	A0A024QZN9_HUMAN (+3)	34 kDa	0.0016	BRAC1 low, Naive high	8	10	11	6	6	10
Voltage-dependent anion-selective channel protein 1 OS=Homo sapiens GN=VDAC1 PE=1 SV=2	VDAC1_HUMAN	31 kDa	0.0088	BRAC1 low, Naive high	11	21	17	3	8	8
Voltage-dependent anion-selective channel protein 3 OS=Homo sapiens GN=VDAC3 PE=1 SV=1	VDAC3_HUMAN	31 kDa	0.12	[]	1	0	2	0	0	0
Voltage-dependent calcium channel subunit alpha-2/delta-1 OS=Homo sapiens GN=CACNA2D1 PE=1 SV=3	CA2D1_HUMAN	125 kDa	0.37	[]	0	0	6	0	0	0
WASH complex subunit strumpellin OS=Homo sapiens GN=KIAA0196 PE=1 SV=1	STRUM_HUMAN (+1)	134 kDa	0.37	[]	0	0	0	0	0	4
Epididymis secretory protein Li 52 OS=Homo sapiens GN=HEL-S-52 PE=2 SV=1	V9HWG7_HUMAN (+1)	66 kDa	0.63	[]	3	3	17	9	17	18
WD repeat domain 1, isoform CRA_a (Fragment) OS=Homo sapiens GN=WDR1 PE=2 SV=1	Q53GN4_HUMAN (+2)	66 kDa	0.73	[]	3	3	18	9	17	17
WD repeat-containing protein 11 OS=Homo sapiens GN=WDR11 PE=1 SV=1	WDR11_HUMAN (+2)	137 kDa	0.37	[]	0	0	0	0	0	2
WD repeat-containing protein 61 OS=Homo sapiens GN=WDR61 PE=1 SV=1	WDR61_HUMAN (+1)	34 kDa	0.37	[]	0	0	0	0	0	4
Wiskott-Aldrich syndrome protein family member 2 OS=Homo sapiens GN=WASF2 PE=1 SV=3	WASF2_HUMAN	54 kDa	0.027	BRAC1 high, Naive low	0	0	1	6	5	3
Wolframin OS=Homo sapiens GN=WFS1 PE=1 SV=2	WFS1_HUMAN	100 kDa	0.19	[]	1	2	1	1	1	2
X-ray repair complementing defective repair in Chinese hamster cells 6 (Ku autoantigen, 70kDa), isoform CRA_a OS=Homo sapiens GN	A0A024R1N4_HUMAN (+1)	70 kDa	0.2	[]	0	1	4	3	5	13
X-ray repair cross-complementing protein 5 OS=Homo sapiens GN=XRCC5 PE=1 SV=3	XRCC5_HUMAN	83 kDa	0.3	[]	0	0	3	2	2	9
Xaa-Pro dipeptidase OS=Homo sapiens GN=PEPD PE=1 SV=3	PEPD_HUMAN (+1)	55 kDa	0.69	[]	0	0	1	0	0	3
Zinc finger CCCH domain-containing protein 15 OS=Homo sapiens GN=ZC3H15 PE=1 SV=1	ZC3HF_HUMAN (+1)	49 kDa	0.37	[]	0	0	0	0	0	2
Zinc finger protein 185 (Fragment) OS=Homo sapiens GN=ZNF185 PE=1 SV=2	H0Y704_HUMAN	48 kDa	0.12	[]	0	0	0	7	7	0
Zinc finger protein 185 OS=Homo sapiens GN=ZNF185 PE=1 SV=3	ZN185_HUMAN	74 kDa	0.12	[]	0	0	0	7	7	0
Zinc transporter 1 OS=Homo sapiens GN=SLC30A1 PE=1 SV=3	ZNT1_HUMAN	55 kDa	0.3	[]	0	0	1	2	2	0
Zinc transporter ZIP14 OS=Homo sapiens GN=SLC39A14 PE=1 SV=3	S39AE_HUMAN (+1)	54 kDa	0.12	[]	0	2	2	0	0	0
Zinc-alpha-2-glycoprotein OS=Homo sapiens GN=AZGP1 PE=1 SV=2	ZA2G_HUMAN (+1)	34 kDa	0.37	[]	0	0	0	0	0	3
Zyxin OS=Homo sapiens GN=ZYX PE=1 SV=1	ZYX_HUMAN	61 kDa	0.47	[]	3	6	5	7	7	5
cAMP-dependent protein kinase catalytic subunit beta OS=Homo sapiens GN=PRKACB PE=1 SV=2	KAPCB_HUMAN (+5)	41 kDa	0.13	[]	0	1	2	0	0	0
cAMP-dependent protein kinase type I-alpha regulatory subunit OS=Homo sapiens GN=PRKAR1A PE=1 SV=1	KAP0_HUMAN (+3)	43 kDa	0.12	[]	1	1	1	0	0	2
cDNA FLJ12766 fis, clone NT2RP2001520, highly similar to Calcium-binding mitochondrial carrier protein Aralar1 OS=Homo sapiens PE	B3KMV8_HUMAN (+1)	75 kDa	0.68	[]	1	1	0	0	2	4
cDNA FLJ14916 fis, clone PLACE1006958, highly similar to Heat shock 70 kDa protein 4L (Fragment) OS=Homo sapiens PE=2 SV=1	B3KNM0_HUMAN (+4)	64 kDa	0.13	[]	0	0	0	2	0	4
cDNA FLJ16785 fis, clone NT2RI2015342, highly similar to Solute carrier family 2, facilitated glucose transporter member 1 OS=Homo s	B3KVN0_HUMAN (+3)	46 kDa	0.013	BRAC1 high, Naive low	0	1	1	5	8	6
cDNA FLJ31446 fis, clone NT2NE2000909, highly similar to Guanine nucleotide-binding protein G(o) subunit alpha 1 OS=Homo sapiens	B3KP89_HUMAN (+1)	40 kDa	0.17	[]	0	2	6	0	0	0
cDNA FLJ32798 fis, clone TESTI2002498, highly similar to Homo sapiens membrane protein, palmitoylated 7 (MAGUK p55 subfamily m	B3KQ45_HUMAN (+1)	66 kDa	0.076	[]	0	0	0	1	4	2
cDNA FLJ34794 fis, clone NT2NE2005676, highly similar to Gamma-tubulin complex component 2 OS=Homo sapiens PE=2 SV=1	B3KRS1_HUMAN (+3)	83 kDa	0.19	[]	0	0	0	0	2	1
cDNA FLJ36069 fis, clone TESTI2019406, highly similar to HEME OXYGENASE 2 (EC 1.14.99.3) OS=Homo sapiens PE=2 SV=1	B3KSE0_HUMAN (+3)	36 kDa	0.37	[]	0	0	1	1	0	5
cDNA FLJ41755 fis, clone HSYRA2009102, highly similar to Adenosine 3'-phospho 5'-phosphosulfate transporter 1 OS=Homo sapiens P	B3KVY9_HUMAN (+1)	45 kDa	0.21	[]	1	1	1	1	0	2
cDNA FLJ44733 fis, clone BRACE3026290, highly similar to Lethal(2) giant larvae protein homolog 2 OS=Homo sapiens PE=2 SV=1	B3KX47_HUMAN (+2)	111 kDa	0.11	[]	0	0	0	3	2	1
cDNA FLJ44920 fis, clone BRAMY3011501, highly similar to Heterogeneous nuclear ribonucleoprotein U OS=Homo sapiens PE=2 SV=1	B3KX72_HUMAN (+1)	83 kDa	0.48	[]	2	1	6	3	3	4
cDNA FLJ50039, highly similar to Homo sapiens solute carrier family 25, member 24, transcript variant 1, mRNA OS=Homo sapiens PE	B4E290_HUMAN (+2)	53 kDa	0.25	[]	2	0	4	1	0	2
cDNA FLJ50778, highly similar to Protein flightless-1 homolog OS=Homo sapiens PE=2 SV=1	B4DIX0_HUMAN (+1)	141 kDa	0.11	[]	0	0	0	1	1	5
cDNA FLJ51365, highly similar to Flap endonuclease 1 (EC 3.1.-.-) OS=Homo sapiens PE=2 SV=1	B4DWZ4_HUMAN (+2)	39 kDa	0.028	BRAC1 high, Naive low	0	0	0	1	2	1
cDNA FLJ51671, highly similar to Prenylcysteine oxidase (EC 1.8.3.5) OS=Homo sapiens PE=2 SV=1	B7Z8A2_HUMAN (+1)	55 kDa	0.18	[]	4	2	7	1	3	7
cDNA FLJ53290, highly similar to Cytoplasmic dynein 1 intermediate chain 2 OS=Homo sapiens PE=2 SV=1	B4DPZ3_HUMAN (+1)	70 kDa	0.18	[]	4	0	6	1	0	2
cDNA FLJ53321, highly similar to Homo sapiens pitrilysin metallopeptidase 1 (PITRM1), mRNA OS=Homo sapiens PE=2 SV=1	B4DH07_HUMAN (+2)	116 kDa	0.27	[]	0	0	2	2	2	2
cDNA FLJ53478, highly similar to Galectin-3-binding protein OS=Homo sapiens PE=2 SV=1	B4DVE1_HUMAN (+1)	64 kDa	0.0011	BRAC1 high, Naive low	0	0	1	13	11	21
Heat shock protein 90kDa alpha (Cytosolic), class B member 1, isoform CRA_a OS=Homo sapiens GN=HSP90AB1 PE=3 SV=1	A0A024RD80_HUMAN (+1)	83 kDa	0.14	[]	10	8	14	26	23	66
cDNA FLJ53619, highly similar to Heat shock protein HSP 90-beta OS=Homo sapiens PE=2 SV=1	B4DGL0_HUMAN	82 kDa	0.19	[]	10	8	12	23	20	63
cDNA FLJ53327, highly similar to Gelsolin OS=Homo sapiens PE=2 SV=1	B7Z2X4_HUMAN	78 kDa	0.027	BRAC1 low, Naive high	7	12	19	5	7	15
cDNA FLJ53698, highly similar to Gelsolin OS=Homo sapiens PE=2 SV=1	B7Z992_HUMAN	79 kDa	0.028	BRAC1 low, Naive high	7	13	20	5	7	15
cDNA FLJ53963, highly similar to Leukocyte elastase inhibitor OS=Homo sapiens PE=2 SV=1	B4E3A8_HUMAN (+2)	39 kDa	0.03	BRAC1 high, Naive low	0	0	0	1	1	3
cDNA FLJ54014, highly similar to All-trans-retinol 13,14-reductase(EC 1.3.99.23) OS=Homo sapiens PE=2 SV=1	B4DKE1_HUMAN (+2)	61 kDa	0.88	[]	0	0	2	1	1	1
cDNA FLJ54117, highly similar to CD97 antigen OS=Homo sapiens PE=2 SV=1	B4DTS6_HUMAN (+2)	84 kDa	0.45	[]	0	0	2	2	1	2
cDNA FLJ54170, highly similar to Cytosolic nonspecific dipeptidase OS=Homo sapiens PE=2 SV=1	B4DV28_HUMAN (+2)	52 kDa	0.11	[]	0	0	1	2	2	1
cDNA FLJ54290, highly similar to Mitochondrial inner membrane protein OS=Homo sapiens PE=2 SV=1	B4DS66_HUMAN (+1)	73 kDa	0.68	[]	0	1	7	1	5	11
cDNA FLJ54303, highly similar to Heat shock 70 kDa protein 1 OS=Homo sapiens PE=2 SV=1	B4DFN9_HUMAN	62 kDa	0.043	BRAC1 high, Naive low	8	6	14	22	22	39
cDNA FLJ54370, highly similar to Heat shock 70 kDa protein 1 OS=Homo sapiens PE=2 SV=1	B4DNT8_HUMAN	67 kDa	0.016	BRAC1 high, Naive low	6	5	13	23	22	40
cDNA FLJ54723, highly similar to Poly (ADP-ribose) polymerase 9 (EC 2.4.2.30) OS=Homo sapiens PE=2 SV=1	B7Z5L6_HUMAN (+1)	88 kDa	0.03	BRAC1 high, Naive low	0	0	0	1	1	3

cDNA FLJ54827, highly similar to Alpha-parvin OS=Homo sapiens PE=2 SV=1	B7Z952_HUMAN (+3)	33 kDa	0.75	[]	2	0	3	2	2	3
cDNA FLJ55671, highly similar to Ubiquitin-associated protein 2-like OS=Homo sapiens PE=2 SV=1	B4DYY5_HUMAN (+2)	114 kDa	0.26	[]	0	1	0	1	1	3
cDNA FLJ55697, highly similar to Protein transport protein Sec23B OS=Homo sapiens PE=2 SV=1	B4DJW8_HUMAN (+2)	84 kDa	0.2	[]	0	0	0	1	2	13
cDNA FLJ55809 OS=Homo sapiens PE=2 SV=1	B4DN31_HUMAN (+2)	51 kDa	0.26	[]	0	0	1	2	0	4
cDNA FLJ56108, highly similar to Puromycin-sensitive aminopeptidase (EC 3.4.11.-) OS=Homo sapiens PE=2 SV=1	B7Z4B2_HUMAN (+3)	93 kDa	0.39	[]	0	0	2	1	1	6
cDNA FLJ56274, highly similar to Transketolase (EC 2.2.1.1) OS=Homo sapiens PE=2 SV=1	B4E022_HUMAN (+3)	63 kDa	0.49	[]	7	5	15	11	11	15
cDNA FLJ56280, highly similar to Endoplasmic reticulum-Golgi intermediate compartment protein 1 OS=Homo sapiens PE=2 SV=1	B4E0N6_HUMAN (+1)	26 kDa	0.32	[]	0	0	2	0	3	7
cDNA FLJ56307, highly similar to Ubiquitin thioesterase protein OTUB1 (EC 3.4.-.-) OS=Homo sapiens PE=2 SV=1	B4DPD5_HUMAN (+3)	35 kDa	0.011	BRAC1 high, Naive low	0	0	0	1	2	3
cDNA FLJ56381, highly similar to Dynamin-1-like protein (EC 3.6.5.5) OS=Homo sapiens PE=2 SV=1	B4DYR6_HUMAN (+6)	85 kDa	0.15	[]	0	0	2	2	2	4
cDNA FLJ56822, highly similar to Alpha-2-HS-glycoprotein OS=Homo sapiens PE=2 SV=1	B7Z556_HUMAN (+2)	27 kDa	0.0046	BRAC1 low, Naive high	2	3	3	1	2	2
cDNA FLJ57632, highly similar to Vacuolar ATP synthase subunit B, kidney isoform (EC 3.6.3.14) OS=Homo sapiens PE=2 SV=1	B4DWH7_HUMAN (+2)	54 kDa	0.7	[]	0	1	0	0	0	4
cDNA FLJ58042, highly similar to Protein NipSnap1 OS=Homo sapiens PE=2 SV=1	B4DQI7_HUMAN (+1)	31 kDa	0.42	[]	0	1	1	1	1	4
cDNA FLJ58258, highly similar to Cyclin G-associated kinase (EC 2.7.11.1) OS=Homo sapiens PE=2 SV=1	B4DS37_HUMAN (+1)	132 kDa	0.11	[]	0	0	0	1	1	5
cDNA FLJ58285, highly similar to Homo sapiens pre-B-cell leukemia transcription factor interacting protein 1 (PBXIP1), mRNA OS=Hom	B4DV94_HUMAN (+3)	58 kDa	0.22	[]	1	1	3	1	0	3
cDNA FLJ58682, highly similar to Vesicle-fusing ATPase (EC 3.6.4.6) OS=Homo sapiens PE=2 SV=1	B7Z5J7_HUMAN (+2)	82 kDa	0.23	[]	0	1	3	2	4	11
cDNA FLJ59571, highly similar to Eukaryotic translation initiation factor 4gamma 2 OS=Homo sapiens PE=2 SV=1	B4DZF2_HUMAN (+3)	110 kDa	0.14	[]	0	0	0	1	1	6
cDNA FLJ52902, highly similar to Rab GDP dissociation inhibitor alpha OS=Homo sapiens PE=2 SV=1	B4DHX4_HUMAN (+1)	47 kDa	0.00013	BRAC1 high, Naive low	0	0	0	5	4	6
cDNA FLJ60299, highly similar to Rab GDP dissociation inhibitor beta OS=Homo sapiens PE=2 SV=1	B4DLV7_HUMAN (+2)	51 kDa	0.029	BRAC1 high, Naive low	1	1	4	6	6	9
cDNA FLJ60385, highly similar to Integrin alpha-3 OS=Homo sapiens PE=2 SV=1	B4E0H8_HUMAN (+1)	115 kDa	0.8	[]	3	3	8	8	7	5
cDNA FLJ60461, highly similar to Peroxiredoxin-2 (EC 1.11.1.15) OS=Homo sapiens PE=2 SV=1	B4DF70_HUMAN (+2)	20 kDa	0.7	[]	6	5	3	19	9	3
cDNA FLJ60960, highly similar to Phosphatidylinositol 4-kinase alpha (EC 2.7.1.67) OS=Homo sapiens PE=2 SV=1	B4DYG5_HUMAN (+3)	237 kDa	0.17	[]	0	0	0	0	1	3
cDNA FLJ52516, moderately similar to Basigin OS=Homo sapiens PE=2 SV=3	A8MZ77_HUMAN	17 kDa	0.9	[]	7	5	9	17	12	7
cDNA FLJ61188, highly similar to Basigin OS=Homo sapiens PE=2 SV=1	B4DY23_HUMAN (+1)	28 kDa	0.8	[]	10	8	11	19	16	10
cDNA FLJ61189, highly similar to Cysteine and glycine-rich protein 1 OS=Homo sapiens PE=2 SV=1	B4DY28_HUMAN (+1)	20 kDa	0.29	[]	2	3	12	5	4	2
Neutral alpha-glucosidase AB OS=Homo sapiens GN=GANAB PE=1 SV=1	E9PKU7_HUMAN	97 kDa	0.81	[]	8	12	17	11	17	41
cDNA FLJ61290, highly similar to Neutral alpha-glucosidase AB OS=Homo sapiens PE=2 SV=1	B4DJ30_HUMAN (+2)	113 kDa	0.6	[]	13	14	20	18	26	49
cDNA FLJ61587, highly similar to Integrin alpha-1 (Fragment) OS=Homo sapiens PE=2 SV=1	B4DTY8_HUMAN (+1)	130 kDa	0.083	[]	0	1	4	10	9	5
cDNA FLJ75299, highly similar to Xenopus laevis proteasome (prosome, macropain) 26S subunit, ATPase 3, mRNA OS=Homo sapiens P	A8K781_HUMAN (+1)	47 kDa	0.82	[]	0	0	4	1	2	4
cDNA FLJ75516, highly similar to Xenopus tropicalis ubiquitin C, mRNA OS=Homo sapiens PE=2 SV=1	A8K674_HUMAN (+36)	68 kDa	0.41	[]	13	19	6	33	30	29
cDNA FLJ75699, highly similar to Homo sapiens osteoclast stimulating factor 1 (OSTF1), mRNA OS=Homo sapiens PE=2 SV=1	A8K646_HUMAN (+1)	24 kDa	0.13	[]	0	0	0	1	0	2
Complement component 1 Q subcomponent-binding protein, mitochondrial OS=Homo sapiens GN=C1QBP PE=1 SV=1	I3L3B0_HUMAN (+1)	20 kDa	0.39	[]	1	1	2	0	0	5
cDNA FLJ75700, highly similar to Homo sapiens complement component 1, q subcomponent binding protein (C1QBP), nuclear gene e	A8K651_HUMAN (+1)	31 kDa	0.19	[]	1	2	5	1	0	6
cDNA FLJ75774, highly similar to Homo sapiens lectin, mannose-binding 2 (LMAN2), mRNA OS=Homo sapiens PE=2 SV=1	A8K7T4_HUMAN (+1)	40 kDa	0.97	[]	0	4	3	2	0	10
Transferrin receptor protein 1 OS=Homo sapiens GN=TFRC PE=1 SV=2	TFR1_HUMAN	85 kDa	0.036	BRAC1 high, Naive low	7	7	6	22	19	21
cDNA FLJ75881, highly similar to Homo sapiens transferrin receptor (p90, CD71) (TFRC), mRNA OS=Homo sapiens PE=2 SV=1	A8K6Q8_HUMAN	85 kDa	0.031	BRAC1 high, Naive low	7	7	7	22	19	21
cDNA FLJ75930, highly similar to Homo sapiens NADH dehydrogenase (ubiquinone) 1 alpha subcomplex, 9, 39kDa (NDUFA9), mRNA O	A8K4V2_HUMAN (+2)	42 kDa	0.37	[]	0	0	0	0	0	2
cDNA FLJ76064 OS=Homo sapiens PE=2 SV=1	A8K9V9_HUMAN (+1)	35 kDa	0.077	[]	0	0	0	5	3	1
cDNA FLJ76121, highly similar to Homo sapiens zinc finger CCCH-type, antiviral 1 (ZC3HAV1), transcript variant 1, mRNA OS=Homo sap	A8K9U6_HUMAN (+1)	101 kDa	0.86	[]	3	2	2	4	3	7
cDNA FLJ76169 OS=Homo sapiens PE=2 SV=1	A8K4K9_HUMAN (+2)	35 kDa	0.5	[]	0	1	2	1	2	4
cDNA FLJ76245, highly similar to Homo sapiens desmocollin 2 (DSC2), transcript variant Dsc2a, mRNA OS=Homo sapiens PE=2 SV=1	A8K2P8_HUMAN (+6)	100 kDa	0.12	[]	0	0	0	2	2	0
cDNA FLJ76605, highly similar to Homo sapiens secretory carrier membrane protein 1 (SCAMP1), transcript variant 1, mRNA OS=Hom	A8K2G0_HUMAN (+1)	38 kDa	0.11	[]	1	1	3	1	0	2
cDNA FLJ76654, highly similar to Homo sapiens CD44 antigen (homing function and Indian blood group system) (CD44), transcript var	A8K309_HUMAN (+2)	39 kDa	0.07	[]	12	13	21	16	19	9
cDNA FLJ76789, highly similar to Homo sapiens methionine-tRNA synthetase (MARS), mRNA OS=Homo sapiens PE=2 SV=1	A8K492_HUMAN (+1)	101 kDa	0.18	[]	0	0	0	5	1	22
cDNA FLJ76863, highly similar to Homo sapiens stress-induced-phosphoprotein 1 (Hsp70/Hsp90-organizing protein) (STIP1), mRNA OS	A8K690_HUMAN (+2)	63 kDa	0.14	[]	0	0	1	2	1	6
cDNA FLJ77464, highly similar to Homo sapiens protocadherin 1 (cadherin-like 1) (PCDH1), transcript variant 1, mRNA OS=Homo sapie	A8K0E7_HUMAN (+3)	115 kDa	0.2	[]	0	0	0	1	3	0
cDNA FLJ77590, highly similar to Homo sapiens leucine rich repeat containing 1, mRNA OS=Homo sapiens PE=2 SV=1	A8K883_HUMAN (+1)	59 kDa	0.0049	BRAC1 high, Naive low	0	0	0	21	19	15
cDNA FLJ77721, highly similar to Homo sapiens secretory carrier membrane protein 2 (SCAMP2), mRNA OS=Homo sapiens PE=2 SV=1	A8K769_HUMAN (+2)	37 kDa	0.079	[]	1	1	2	3	3	3
cDNA FLJ77762, highly similar to Homo sapiens cullin-associated and neddylation-dissociated 1 (CAND1), mRNA OS=Homo sapiens PE	A8K8U1_HUMAN (+1)	136 kDa	0.13	[]	0	2	7	8	11	10
cDNA FLJ77770, highly similar to Homo sapiens nucleobindin 1 (NUCB1), mRNA OS=Homo sapiens PE=2 SV=1	A8K7Q1_HUMAN (+2)	54 kDa	0.67	[]	2	3	2	2	2	7
Eukaryotic initiation factor 4A-II OS=Homo sapiens GN=EIF4A2 PE=1 SV=1	E7EQG2_HUMAN (+1)	41 kDa	0.51	[]	3	0	4	4	3	13
cDNA FLJ78244, highly similar to Homo sapiens eukaryotic translation initiation factor 4A, isoform 1 (EIF4A1), mRNA OS=Homo sapien	A8K7F6_HUMAN (+1)	46 kDa	0.45	[]	3	1	4	4	3	23
cDNA FLJ78449 OS=Homo sapiens PE=2 SV=1	A8K5S3_HUMAN (+1)	40 kDa	0.026	BRAC1 high, Naive low	0	0	0	2	3	7
cDNA FLJ78458, highly similar to Homo sapiens septin 10 (SEPT10), transcript variant 1, mRNA OS=Homo sapiens PE=2 SV=1	A8K7M3_HUMAN (+4)	53 kDa	0.16	[]	0	0	0	0	3	2
cDNA FLJ78497 OS=Homo sapiens PE=2 SV=1	A8K1E9_HUMAN (+4)	54 kDa	0.37	[]	0	0	0	0	0	2
cDNA FLJ78586, highly similar to Homo sapiens VAMP (vesicle-associated membrane protein)-associated protein A, 33kDa (VAPA), m	A8KA83_HUMAN (+1)	27 kDa	0.41	[]	0	2	3	2	4	6
cDNA FLJ78635, highly similar to Homo sapiens ATP synthase, H+ transporting, mitochondrial F0 complex, subunit b, isoform 1 (ATP5F	A8K4W2_HUMAN (+3)	29 kDa	0.93	[]	0	0	4	1	1	3
cDNA FLJ90354 fis, clone NT2RP2003390, highly similar to Translocation protein SEC63 homolog OS=Homo sapiens PE=2 SV=1	B3KQF0_HUMAN (+1)	88 kDa	0.82	[]	2	1	1	1	0	6
cDNA, FLJ93042, highly similar to Homo sapiens signal sequence receptor, alpha (translocon-associated protein alpha) (SSR1), mRNA	B2R6N9_HUMAN (+5)	32 kDa	0.3	[]	3	2	1	1	2	3
cDNA, FLJ93299, highly similar to Homo sapiens sterol carrier protein 2 (SCP2), mRNA OS=Homo sapiens PE=2 SV=1	B2R761_HUMAN (+1)	59 kDa	0.04	BRAC1 low, Naive high	2	2	2	1	2	0
cDNA, FLJ93507, highly similar to Homo sapiens ATPase, H+ transporting, lysosomal 38kDa, V0 subunit d isoform 1 (ATP6V0D1), mRN	B2R7M1_HUMAN (+2)	40 kDa	0.39	[]	0	2	4	2	0	2
cDNA, FLJ93750, Homo sapiens DEAD (Asp-Glu-Ala-Asp) box polypeptide 6 (DDX6), mRNA OS=Homo sapiens PE=2 SV=1	B2R858_HUMAN (+1)	53 kDa	0.13	[]	0	0	0	1	0	2
cDNA, FLJ94230, highly similar to Homo sapiens thioredoxin-like 1 (TXNL1), mRNA OS=Homo sapiens PE=2 SV=1	B2R960_HUMAN (+2)	32 kDa	0.96	[]	0	0	2	1	0	2

cDNA, FLJ94267, highly similar to Homo sapiens glutathione S-transferase omega 1 (GSTO1), mRNA OS=Homo sapiens PE=2 SV=1	B2R983_HUMAN (+2)	28 kDa	0.69	[]	0	1	3	1	2	1
cDNA, FLJ94534, highly similar to Homo sapiens capping protein (actin filament), gelsolin-like(CAPG), mRNA OS=Homo sapiens PE=2 S	B2R9S4_HUMAN (+3)	38 kDa	0.017	BRAC1 high, Naive low	0	0	2	4	4	5
cDNA, FLJ94599, highly similar to Homo sapiens GDP-mannose 4,6-dehydratase (GMDS), mRNA OS=Homo sapiens PE=2 SV=1	B2R9X3_HUMAN (+1)	42 kDa	0.17	[]	0	0	0	1	0	3
cDNA, FLJ94965, highly similar to Homo sapiens leucyl/cystinyl aminopeptidase (LNPEP), mRNA OS=Homo sapiens PE=2 SV=1	B2RAK1_HUMAN (+1)	117 kDa	0.077	[]	0	0	2	3	3	3
cDNA, FLJ95288, highly similar to Homo sapiens feline leukemia virus subgroup C cellular receptor (FLVCR), mRNA OS=Homo sapiens I	B2RB38_HUMAN (+1)	60 kDa	0.19	[]	0	0	0	0	2	1
cDNA, FLJ95416, highly similar to Homo sapiens phosphatidylinositol glycan, class S (PIGS), mRNA OS=Homo sapiens PE=2 SV=1	B2RBB2_HUMAN (+2)	62 kDa	0.15	[]	0	0	2	2	2	4
cDNA, FLJ95650, highly similar to Homo sapiens karyopherin (importin) beta 1 (KPNB1), mRNA OS=Homo sapiens PE=2 SV=1	B2RBR9_HUMAN (+1)	97 kDa	0.062	[]	1	3	8	12	10	20
cDNA, FLJ95704, highly similar to Homo sapiens serine protease inhibitor, Kunitz type 1 (SPINT1), mRNA OS=Homo sapiens PE=2 SV=1	B2RBU9_HUMAN (+3)	57 kDa	0.031	BRAC1 high, Naive low	0	0	0	4	2	2
cDNA, FLJ96156, highly similar to Homo sapiens leucyl-tRNA synthetase (LARS), mRNA OS=Homo sapiens PE=2 SV=1	B2RCM2_HUMAN (+3)	134 kDa	0.14	[]	0	1	3	4	4	14
cDNA, FLJ96428, highly similar to Homo sapiens proteasome (prosome, macropain) 26S subunit, non-ATPase, 7 (Mov34 homolog) (PS	B2RD27_HUMAN (+1)	37 kDa	0.13	[]	0	0	0	1	0	2
cDNA, FLJ96568, highly similar to Homo sapiens tropomyosin 3 (TPM3), mRNA OS=Homo sapiens PE=2 SV=1	B2RDE1_HUMAN	29 kDa	0.21	[]	12	13	10	14	16	9
Erlin-2 (Fragment) OS=Homo sapiens GN=ERLIN2 PE=1 SV=1	E5RHW4_HUMAN (+1)	38 kDa	0.052	[]	1	2	3	2	1	1
cDNA, FLJ96656, highly similar to Homo sapiens SPFH domain family, member 1 (SPFH1), mRNA OS=Homo sapiens PE=2 SV=1	B2RDK6_HUMAN (+2)	39 kDa	0.15	[]	2	2	2	3	1	2
cDNA, FLJ96792, highly similar to Homo sapiens calmodulin 2 (phosphorylase kinase, delta) (CALM2), mRNA OS=Homo sapiens PE=2 S	B2RDW0_HUMAN (+4)	17 kDa	0.72	[]	10	8	5	16	11	8
cDNA, FLJ96812, highly similar to Homo sapiens threonyl-tRNA synthetase (TARS), mRNA OS=Homo sapiens PE=1 SV=1	B2RDX5_HUMAN	82 kDa	0.31	[]	0	0	4	4	2	7
tRNA (cytosine(34)-C(5))-methyltransferase OS=Homo sapiens GN=NSUN2 PE=1 SV=2	NSUN2_HUMAN	86 kDa	0.13	[]	0	0	0	0	1	2
tRNA-splicing ligase RtcB homolog OS=Homo sapiens GN=RTCB PE=1 SV=1	RTCB_HUMAN	55 kDa	0.39	[]	0	0	1	0	1	4
tr|A0A024R4E5|A0A024R4E5_HUMAN High density lipoprotein binding protein (Vigilin),...	A0A024R4E5_HUMAN	?	0.56	[]	3	7	6	0	2	18
tr|A0A024R5C4|A0A024R5C4_HUMAN Reticulon OS=Homo sapiens GN=RTN3 PE=4 SV=1	A0A024R5C4_HUMAN	?	0.37	[]	0	0	0	0	0	3
tr|A0A024R152|A0A024R152_HUMAN HCG28765, isoform CRA_b OS=Homo sapiens GN=hCG_28765...	A0A024R152_HUMAN	?	0.018	BRAC1 low, Naive high	3	4	2	0	0	0
tr|A0A024R201|A0A024R201_HUMAN Proteasome (Prosome, macropain) 26S subunit, non-ATPase,...	A0A024R201_HUMAN	?	0.55	[]	0	0	2	0	0	10
tr|A0A024R462|A0A024R462_HUMAN Fibronectin 1, isoform CRA_n OS=Homo sapiens GN=FN1...	A0A024R462_HUMAN	?	0.0046	BRAC1 low, Naive high	30	56	43	0	0	0
tr|A0A024RDY9|A0A024RDY9_HUMAN Rho guanine nucleotide exchange factor (GEF) 7, isoform...	A0A024RDY9_HUMAN (+2)	?	0.42	[]	0	0	1	0	1	3
tr|B2RCM3|B2RCM3_HUMAN cDNA, FLJ96158, highly similar to Homo sapiens calpain 2,...	B2RCM3_HUMAN (+2)	?	0.17	[]	0	1	3	0	0	0
tr|B4DPY0|B4DPY0_HUMAN Glutathione peroxidase OS=Homo sapiens PE=2 SV=1	B4DPY0_HUMAN	?	0.12	[]	0	2	2	0	0	0
tr|B4DTS2|B4DTS2_HUMAN Serine/threonine-protein kinase OS=Homo sapiens GN=PRKD2 PE=1...	B4DTS2_HUMAN	?	0.37	[]	0	0	0	0	0	6
tr|B4DV84|B4DV84_HUMAN cDNA FLJ56159, highly similar to 26S proteasome non-ATPase...	B4DV84_HUMAN	?	0.47	[]	0	0	1	0	0	8
tr|B4DVS4|B4DVS4_HUMAN cDNA FLJ57619, highly similar to Delta 3,5-delta 2,4-dienoyl-CoA...	B4DVS4_HUMAN	?	0.41	[]	0	0	2	0	4	3
tr|B7Z6U8|B7Z6U8_HUMAN cDNA FLJ53665, highly similar to Four and a half LIM domains...	B7Z6U8_HUMAN (+1)	?	0.25	[]	0	1	6	0	0	0
tr|B7ZKT9|B7ZKT9_HUMAN KIAA1033 protein OS=Homo sapiens GN=KIAA1033 PE=2 SV=1	B7ZKT9_HUMAN	?	0.37	[]	0	0	0	0	0	4
tr|B9EGI2|B9EGI2_HUMAN Myosin phosphatase Rho interacting protein OS=Homo sapiens...	B9EGI2_HUMAN	?	0.014	BRAC1 low, Naive high	1	3	4	0	0	0
tr|D7PBN3|D7PBN3_HUMAN ESRP1/RAF1 fusion protein OS=Homo sapiens PE=2 SV=1	D7PBN3_HUMAN	?	0.37	[]	0	0	0	0	0	5
tr|E7ESL9|E7ESL9_HUMAN Arf-GAP with GTPase, ANK repeat and PH domain-containing protein...	E7ESL9_HUMAN	?	0.37	[]	0	0	0	0	0	2
tr|B7Z6G4|B7Z6G4_HUMAN cDNA FLJ53193, highly similar to Homo sapiens caldesmon 1...	B7Z6G4_HUMAN	?	< 0.00010	BRAC1 low, Naive high	26	28	39	0	0	0
tr|E7EX44|E7EX44_HUMAN Caldesmon OS=Homo sapiens GN=CALD1 PE=1 SV=1	E7EX44_HUMAN	?	< 0.00010	BRAC1 low, Naive high	26	30	42	0	0	0
tr|Q6ICT6|Q6ICT6_HUMAN TNFRSF6 protein (Fragment) OS=Homo sapiens GN=TNFRSF6 PE=2...	Q6ICT6_HUMAN	?	0.28	[]	0	1	4	0	1	0
