# Supplementary material for: Exosomes isolated from cancer patients’ sera transfer malignant traits and confer the same phenotype of primary tumors to oncosuppressor-mutated cells
Source: J Exp Clin Cancer Res. 2017 Aug 30;36:113. doi: 10.1186/s13046-017-0587-0 (PMC5577828; doi:10.1186/s13046-017-0587-0)
Supplement: Supplementary file 3 — Plasma membrane proteins involved in microvesicles uptake*. (PDF 239 kb) [file 13046_2017_587_MOESM3_ESM.pdf]

Supplementary Table 3. Plasma membrane proteins involved in microvesicles uptake*.

			T-Test		Control			BRCA1-KO		
**Identified Proteins**	**Alternate Name**	**MW**	**(p-value)**	**Quantitative Profile**	**1**	**2**	**3**	**1**	**2**	**3**
Dynamin-2 OS=Homo sapiens GN=DNM2 PE=1 SV=2	DYN2_HUMAN	98 kDa	0.00022	BRAC1 high, Naive low	0	0	1	**13**	**11**	**18**
Dynamin-1 OS=Homo sapiens GN=DNM1 PE=1 SV=1	A0A0D9SFB1_HUMAN (+3)	94 kDa	0.01	BRAC1 high, Naive low	0	0	0	**2**	**4**	**3**
cDNA FLJ56381, highly similar to Dynamin-1-like protein (EC 3.6.5.5) OS=Homo sapiens PE=2 SV=1	B4DYR6_HUMAN (+6)	85 kDa	0.15	[]	0	0	2	2	2	4
Dynamin-like 120 kDa protein, mitochondrial OS=Homo sapiens GN=OPA1 PE=1 SV=1	E5KLJ5_HUMAN (+11)	118 kDa	0.5	[]	0	0	1	2	0	1
Flotillin-1 OS=Homo sapiens GN=FLOT1 PE=1 SV=3	FLOT1_HUMAN (+2)	47 kDa	0.023	BRAC1 high, Naive low	4	4	8	**13**	**13**	**14**
Integrin alpha-2 OS=Homo sapiens GN=ITGA2 PE=1 SV=1	ITA2_HUMAN	129 kDa	0.2	[]	10	12	14	41	34	20
Integrin alpha-5 OS=Homo sapiens GN=ITGA5 PE=1 SV=2	ITA5_HUMAN (+1)	115 kDa	0.00086	BRAC1 low, Naive high	**7**	**7**	**8**	0	0	0
Integrin alpha-6 OS=Homo sapiens GN=ITGA6 PE=1 SV=5	ITA6_HUMAN	127 kDa	0.0036	BRAC1 high, Naive low	0	0	0	**41**	**36**	**30**
Integrin beta-1 OS=Homo sapiens GN=ITGB1 PE=1 SV=2	ITB1_HUMAN	88 kDa	0.77	[]	14	18	21	38	39	17
Integrin beta-5 OS=Homo sapiens GN=ITGB5 PE=1 SV=1	ITB5_HUMAN (+1)	88 kDa	0.061	[]	1	1	2	3	5	9
Integrin beta OS=Homo sapiens GN=ITGB4 PE=3 SV=1	A0A024R8K7_HUMAN (+2)	195 kDa	0.0049	BRAC1 high, Naive low	0	0	0	**59**	**54**	**41**
Integrin beta OS=Homo sapiens PE=2 SV=1	A8K2N5_HUMAN (+3)	86 kDa	0.013	BRAC1 high, Naive low	0	0	0	**2**	**3**	**6**
Integrin alpha-2 OS=Homo sapiens GN=ITGA2 PE=1 SV=1	E7ESP4_HUMAN	103 kDa	0.18	[]	8	12	13	40	33	19
Galectin OS=Homo sapiens GN=hCG_22119 PE=4 SV=1	A0A024R693_HUMAN (+3)	26 kDa	0.53	[]	2	2	2	7	6	1
Galectin-1 OS=Homo sapiens GN=LGALS1 PE=1 SV=2	LEG1_HUMAN	15 kDa	0.0096	BRAC1 low, Naive high	**3**	**6**	**4**	0	0	0
Galectin-4 OS=Homo sapiens GN=LGALS4 PE=1 SV=1	LEG4_HUMAN (+1)	36 kDa	0.057	[]	0	0	2	**104**	**113**	**26**
cDNA FLJ53478, highly similar to Galectin-3-binding protein OS=Homo sapiens PE=2 SV=1	B4DVE1_HUMAN (+1)	64 kDa	0.0011	BRAC1 high, Naive low	0	0	1	**13**	**11**	**21**
Ras-related C3 botulinum toxin substrate 1 (Rho family, small GTP binding protein Rac1) OS=Homo sapiens GN=RAC1 PE=2 SV=	A4D2P0_HUMAN	23 kDa	0.13	[]	1	1	0	6	9	2
Ras-related C3 botulinum toxin substrate 1 (Rho family, small GTP binding protein Rac1) OS=Homo sapiens GN=RAC1 PE=2 SV=	A4D2P1_HUMAN (+1)	21 kDa	0.086	[]	0	0	0	2	6	2
Ras-related C3 botulinum toxin substrate 2 (Rho family, small GTP binding protein Rac2), isoform CRA_a OS=Homo sapiens GN=I	A0A024R1P2_HUMAN (+2)	21 kDa	0.5	[]	1	1	0	1	3	2
Rho guanine nucleotide exchange factor (GEF) 1, isoform CRA_e OS=Homo sapiens GN=ARHGEF1 PE=4 SV=1	A0A024R0R1_HUMAN (+2)	102 kDa	0.095	[]	0	0	0	1	6	13
Ras homolog gene family, member A, isoform CRA_a OS=Homo sapiens GN=RHOA PE=3 SV=1	A0A024R324_HUMAN (+3)	22 kDa	0.29	[]	3	4	3	11	8	6
Rho GTPase activating protein OS=Homo sapiens PE=2 SV=1	A9UK01_HUMAN (+1)	75 kDa	0.27	[]	0	0	0	0	1	7
Rho guanine nucleotide exchange factor 16 OS=Homo sapiens GN=ARHGEF16 PE=1 SV=1	ARHGG_HUMAN (+1)	80 kDa	0.14	[]	0	0	0	1	1	6
CDC42 effector protein (Rho GTPase binding) 4, isoform CRA_a OS=Homo sapiens GN=CDC42EP4 PE=2 SV=1	B2R6D8_HUMAN (+1)	38 kDa	0.028	BRAC1 high, Naive low	0	0	0	**1**	**2**	**1**
tr|B9EGI2|B9EGI2_HUMAN Myosin phosphatase Rho interacting protein OS=Homo sapiens...	B9EGI2_HUMAN	?	0.014	BRAC1 low, Naive high	**1**	**3**	**4**	0	0	0
FERM, RhoGEF and pleckstrin domain-containing protein 1 OS=Homo sapiens GN=FARP1 PE=1 SV=1	FARP1_HUMAN	119 kDa	0.095	[]	4	3	5	4	4	6
FERM, RhoGEF and pleckstrin domain-containing protein 2 OS=Homo sapiens GN=FARP2 PE=1 SV=3	FARP2_HUMAN	120 kDa	0.12	[]	0	0	0	4	5	0
Rho GDP-dissociation inhibitor 1 OS=Homo sapiens GN=ARHGDIA PE=1 SV=3	GDIR1_HUMAN (+3)	23 kDa	0.48	[]	1	1	4	0	3	3
Rho GTPase-activating protein 1 OS=Homo sapiens GN=ARHGAP1 PE=1 SV=1	RHG01_HUMAN	50 kDa	0.57	[]	1	1	5	0	0	7
Clathrin heavy chain OS=Homo sapiens GN=CLTC PE=1 SV=1	A0A087WVQ6_HUMAN (+1)	192 kDa	0.14	[]	19	29	51	21	31	55
Clathrin interactor 1 OS=Homo sapiens GN=CLINT1 PE=1 SV=1	EPN4_HUMAN	68 kDa	0.21	[]	0	0	0	0	1	4
RAB5C, member RAS oncogene family, isoform CRA_a OS=Homo sapiens GN=RAB5C PE=3 SV=1	A0A024R1U4_HUMAN (+1)	23 kDa	0.13	[]	6	4	8	4	8	6
RAB5A, member RAS oncogene family, isoform CRA_a OS=Homo sapiens GN=RAB5A PE=3 SV=1	A0A024R2K1_HUMAN (+1)	24 kDa	0.82	[]	1	1	2	3	0	3
RAB6A, member RAS oncogene family, isoform CRA_b OS=Homo sapiens GN=RAB6A PE=3 SV=1	A0A024R5H8_HUMAN (+2)	24 kDa	0.35	[]	1	1	1	5	3	1
RAB11A, member RAS oncogene family, isoform CRA_a OS=Homo sapiens GN=RAB11A PE=3 SV=1	A0A024R5Z8_HUMAN (+1)	24 kDa	0.46	[]	6	3	3	4	7	3
RAB8A, member RAS oncogene family, isoform CRA_a OS=Homo sapiens GN=RAB8A PE=3 SV=1	A0A024R7I3_HUMAN (+1)	24 kDa	0.0022	BRAC1 high, Naive low	0	0	1	**5**	**4**	**5**
RAB2, member RAS oncogene family, isoform CRA_a OS=Homo sapiens GN=RAB2 PE=3 SV=1	A0A024R7V6_HUMAN (+1)	24 kDa	0.66	[]	3	2	1	5	3	6
RAB14, member RAS oncogene family, isoform CRA_a OS=Homo sapiens GN=RAB14 PE=3 SV=1	A0A024R845_HUMAN (+1)	24 kDa	0.18	[]	3	2	5	8	8	7
Heparan sulfate proteoglycan 2 (Perlecan), isoform CRA_b OS=Homo sapiens GN=HSPG2 PE=4 SV=1	A0A024RAB6_HUMAN (+1)	464 kDa	0.25	[]	9	6	0	2	4	0
RAB5B, member RAS oncogene family, isoform CRA_a OS=Homo sapiens GN=RAB5B PE=3 SV=1	A0A024RB09_HUMAN (+1)	24 kDa	0.6	[]	3	2	0	1	2	4
RAB21, member RAS oncogene family, isoform CRA_a OS=Homo sapiens GN=RAB21 PE=3 SV=1	A0A024RBA9_HUMAN (+1)	24 kDa	0.49	[]	1	2	1	3	4	2
Ras-related protein Rab-35 (Fragment) OS=Homo sapiens GN=RAB35 PE=1 SV=1	F5H157_HUMAN (+1)	21 kDa	0.14	[]	1	0	0	7	5	1
Ras-related protein Rab-10 OS=Homo sapiens GN=RAB10 PE=1 SV=1	RAB10_HUMAN	23 kDa	0.072	[]	2	1	3	14	13	6
Ras-related protein Rab-25 OS=Homo sapiens GN=RAB25 PE=1 SV=2	RAB25_HUMAN	23 kDa	0.04	BRAC1 high, Naive low	0	0	0	**2**	**3**	**1**
Ras-related protein Rab-34 OS=Homo sapiens GN=RAB34 PE=1 SV=1	RAB34_HUMAN (+3)	29 kDa	0.00091	BRAC1 low, Naive high	**1**	**1**	**2**	0	0	0
Ras-related protein Rab-7a OS=Homo sapiens GN=RAB7A PE=1 SV=1	RAB7A_HUMAN	23 kDa	0.55	[]	6	5	6	6	10	10
Ras-related protein Rab-11B OS=Homo sapiens GN=RAB11B PE=1 SV=4	RB11B_HUMAN	24 kDa	0.57	[]	5	2	2	3	6	2
Ras-related protein Ral-A (Fragment) OS=Homo sapiens GN=RALA PE=1 SV=1	H7C3P7_HUMAN (+1)	18 kDa	0.032	BRAC1 high, Naive low	0	1	2	**9**	**8**	**5**
AP-1 complex subunit beta-1 OS=Homo sapiens GN=AP1B1 PE=1 SV=2	AP1B1_HUMAN	105 kDa	0.032	BRAC1 high, Naive low	3	2	3	**9**	**15**	**12**
AP-2 complex subunit alpha-2 OS=Homo sapiens GN=AP2A2 PE=1 SV=2	AP2A2_HUMAN (+1)	104 kDa	0.86	[]	3	4	7	9	7	5
AP-1 complex subunit gamma-1 OS=Homo sapiens GN=AP1G1 PE=1 SV=5	AP1G1_HUMAN (+2)	91 kDa	0.07	[]	0	0	0	1	1	4
AP-1 complex subunit mu-2 OS=Homo sapiens GN=AP1M2 PE=1 SV=4	AP1M2_HUMAN (+2)	48 kDa	0.00029	BRAC1 high, Naive low	0	0	0	**2**	**2**	**2**
AP-2 complex subunit alpha-1 OS=Homo sapiens GN=AP2A1 PE=1 SV=3	AP2A1_HUMAN	108 kDa	0.034	BRAC1 low, Naive high	**6**	**6**	**10**	8	8	6
Basal cell adhesion molecule (Lutheran blood group) OS=Homo sapiens GN=BCAM PE=4 SV=1	A0A068W6H0_HUMAN (+6)	67 kDa	0.00019	BRAC1 high, Naive low	0	0	0	**16**	**13**	**17**
Carcinoembryonic antigen-related cell adhesion molecule 1 OS=Homo sapiens GN=CEACAM1 PE=1 SV=2	CEAM1_HUMAN (+1)	58 kDa	0.0042	BRAC1 high, Naive low	0	0	0	**5**	**7**	**12**
Epithelial cell adhesion molecule OS=Homo sapiens GN=EPCAM PE=1 SV=2	EPCAM_HUMAN	35 kDa	0.019	BRAC1 high, Naive low	0	0	1	**30**	**36**	**16**
Junctional adhesion molecule A OS=Homo sapiens GN=F11R PE=1 SV=1	A0A087WY82_HUMAN (+4)	30 kDa	0.0015	BRAC1 high, Naive low	0	0	0	**12**	**11**	**10**

Junction adhesion molecule OS=Homo sapiens PE=2 SV=1	Q9Y5B2_HUMAN	28 kDa	0.0046	BRAC1 high, Naive low	0	0	0	**10**	**9**	**7**
Cadherin-1 OS=Homo sapiens GN=CDH1 PE=1 SV=1	A0A087WXI5_HUMAN (+4)	100 kDa	0.048	BRAC1 high, Naive low	0	0	0	**31**	**30**	**9**
Cadherin-11 OS=Homo sapiens GN=CDH11 PE=2 SV=2	CAD11_HUMAN (+3)	88 kDa	0.37	[]	0	2	0	0	0	0
Cadherin-13 OS=Homo sapiens GN=CDH13 PE=1 SV=1	CAD13_HUMAN (+2)	78 kDa	0.069	[]	3	1	2	0	0	0
Cadherin-2 OS=Homo sapiens GN=CDH2 PE=1 SV=4	CADH2_HUMAN (+1)	100 kDa	0.005	BRAC1 low, Naive high	**2**	**3**	**6**	0	0	0
Caveolin-1 OS=Homo sapiens GN=CAV1 PE=1 SV=4	CAV1_HUMAN (+1)	20 kDa	0.003	BRAC1 low, Naive high	**9**	**7**	**10**	0	0	0
Intercellular adhesion molecule 1 OS=Homo sapiens GN=ICAM1 PE=1 SV=2	ICAM1_HUMAN (+1)	58 kDa	0.003	BRAC1 low, Naive high	**7**	**11**	**10**	3	4	2

*Based on:

Kelly J. McKelvey et al., 2015. Exosomes: Mechanisms of Uptake. J Circ Biomark, 4:7.

Laura Ann Mulcahy et al., 2014. Routes and mechanisms of extracellular vesicle uptake. Journal of Extracellular Vesicles 3: 24641
