# Supplementary material for: Exosomes isolated from cancer patients’ sera transfer malignant traits and confer the same phenotype of primary tumors to oncosuppressor-mutated cells
Source: J Exp Clin Cancer Res. 2017 Aug 30;36:113. doi: 10.1186/s13046-017-0587-0 (PMC5577828; doi:10.1186/s13046-017-0587-0)
Supplement: Supplementary file 4 — List of top 100 proteins that are often identified in exosomes (ExoCarta). (PDF 158 kb) [file 13046_2017_587_MOESM4_ESM.pdf]

Supplementary Table 4. List of top 100 proteins that are often identified in exosomes (ExoCarta)

		Exocarta.org	Fibroblasts	
	Gene Symbol	# of times identified	Control	BRCA1-KO
1	CD9	98	Y	Y
2	HSPA8	97	N	N
3	PDCD6IP	96	N	N
4	GAPDH	95	Y	Y
5	ACTB	93	N	N
6	ANXA2	83	Y	Y
7	CD63	82	Y	Y
8	SDCBP	78	Y	Y
9	ENO1	78	Y	Y
10	HSP90AA1	77	N	N
11	TSG101	76	Y	Y
12	PKM	72	N	N
13	LDHA	72	N	N
14	EEF1A1	71	Y	Y
15	YWHAZ	69	Y	Y
16	PGK1	69	N	N
17	EEF2	69	Y	Y
18	ALDOA	69	N	N
19	HSP90AB1	67	Y	Y
20	ANXA5	67	N	N
21	FASN	66	Y	Y
22	YWHAE	65	N	N
23	CLTC	64	Y	Y
24	CD81	64	Y	Y
25	ALB	64	Y	Y
26	VCP	62	N	N
27	TPI1	62	N	N
28	PPIA	62	N	N
29	MSN	62	N	N
30	CFL1	62	N	N
31	PRDX1	61	Y	Y
32	PFN1	61	Y	Y
33	RAP1B	60	Y	Y
34	ITGB1	60	Y	Y
35	HSPA5	58	N	N
36	SLC3A2	57	Y	Y
37	HIST1H4A	57	N	N
38	GNB2	57	Y	Y
39	ATP1A1	57	N	N
40	YWHAQ	56	Y	Y
41	FLOT1	56	Y	Y
42	FLNA	56	Y	Y
43	CLIC1	56	N	N
44	CDC42	56	Y	Y
45	CCT2	56	N	N
46	A2M	55	N	N
47	YWHAG	54	N	N
48	TUBA1B	53	N	N
49	RAC1	53	Y	Y
50	LGALS3BP	53	Y	Y
51	HSPA1A	53	N	N
52	GNAI2	53	N	N
53	ANXA1	53	Y	Y
54	RHOA	52	Y	Y
55	MFGE8	52	Y	Y
56	PRDX2	51	N	N
57	GDI2	51	N	N
58	EHD4	51	Y	Y
59	ACTN4	51	Y	Y
60	YWHAB	50	N	N
61	RAB7A	50	Y	Y
62	LDHB	50	Y	Y
63	GNAS	50	Y	Y
64	TFRC	49	Y	Y
65	RAB5C	49	Y	Y
66	ARF1	49	Y	Y
67	ANXA6	49	Y	Y
68	ANXA11	49	N	N
69	ACTG1	49	N	N
70	KPNB1	48	Y	Y
71	EZR	48	Y	Y
72	ANXA4	48	Y	Y
73	ACLY	48	Y	Y
74	TUBA1C	47	N	N
75	RAB14	47	Y	Y
76	HIST2H4A	47	N	N
77	GNB1	47	Y	Y
78	UBA1	46	N	N
79	THBS1	46	Y	Y
80	RAN	46	Y	Y
81	RAB5A	46	Y	Y
82	PTGFRN	46	Y	Y
83	CCT5	46	N	N
84	CCT3	46	N	N
85	BSG	46	N	N
86	AHCY	46	Y	Y
87	RAB5B	45	N	N
88	RAB1A	45	N	N
89	LAMP2	45	N	N
90	ITGA6	45	Y	Y
91	HIST1H4B	45	N	N
92	GSN	45	Y	Y
93	FN1	45	Y	Y
94	YWHAH	44	Y	Y
95	TUBA1A	44	N	N
96	TKT	44	N	N
97	TCP1	44	Y	Y
98	STOM	44	Y	Y
99	SLC16A1	44	Y	Y
100	RAB8A	44	Y	Y

Y: Present in our MS analyses. N: Absent in our MS analyses
