# Supplementary material for: Exosomes isolated from cancer patients’ sera transfer malignant traits and confer the same phenotype of primary tumors to oncosuppressor-mutated cells
Source: J Exp Clin Cancer Res. 2017 Aug 30;36:113. doi: 10.1186/s13046-017-0587-0 (PMC5577828; doi:10.1186/s13046-017-0587-0)
Supplement: Supplementary file 5 — MS data from the exosomes as visualized on Scaffold Q+. (PDF 1267 kb) [file 13046_2017_587_MOESM5_ESM.pdf]

Supplementary Table 5. MS data from the exosomes as visualized on Scaffold Q+.

Identified Proteins	Alternate Name	MW	T-Test (p-value)	Fold Change	Quantitative Profile	Control		BRCA1-KO	
						1	2	1	2
Major vault protein isoform A (Fragment) OS=Homo sapiens GN=MVP PE=2 SV=1	MVP	99 kDa	0.098	0.4	[]	363	592	169	180
sp|ALBU_BOVIN|		69 kDa	0.0061	3.6	Fibro-BKO high, Fibro-Naive low	101	124	367	355
Annexin OS=Homo sapiens GN=ANXA2 PE=3 SV=1	ANXA2	39 kDa	0.025	0.6	Fibro-BKO low, Fibro-Naive high	226	273	134	137
Pyruvate kinase OS=Homo sapiens GN=HEL-S-30 PE=1 SV=1	HEL-S-30	58 kDa	0.014	0.3	Fibro-BKO low, Fibro-Naive high	242	297	77	59
Tubulin alpha chain OS=Homo sapiens PE=2 SV=1		46 kDa	0.87	1	[]	162	135	133	124
Glyceraldehyde-3-phosphate dehydrogenase OS=Homo sapiens GN=HEL-S-162eP PE=2 SV=1	HEL-S-162eP	36 kDa	0.023	0.4	Fibro-BKO low, Fibro-Naive high	112	141	39	52
Histone H4 OS=Homo sapiens GN=HIST1H4H PE=2 SV=1	HIST1H4H	11 kDa	0.22	0.2	[]	173	68	21	19
Fatty acid synthase OS=Homo sapiens GN=FASN PE=1 SV=1	FASN	273 kDa	0.12	1.9	[]	55	111	141	132
Heparan sulfate proteoglycan 2 (Perlecan), isoform CRA_b OS=Homo sapiens GN=HSPG2 PE=4 SV=1	HSPG2	464 kDa	0.015	9.2	Fibro-BKO high, Fibro-Naive low	1	76	283	329
Clathrin heavy chain OS=Homo sapiens GN=CLTC PE=1 SV=1	CLTC	192 kDa	0.35	0.8	[]	60	91	56	50
Thrombospondin 1, isoform CRA_a OS=Homo sapiens GN=THBS1 PE=4 SV=1	THBS1	129 kDa	0.0076	21	Fibro-BKO high, Fibro-Naive low	4	10	130	119
Integrin beta OS=Homo sapiens GN=ITGB4 PE=3 SV=1	ITGB4	195 kDa	0.94	1	[]	52	64	46	58
Junction plakoglobin, isoform CRA_a OS=Homo sapiens GN=JUP PE=4 SV=1	JUP	82 kDa	0.81	1.1	[]	48	74	58	55
Catenin delta-1 OS=Homo sapiens GN=CTNND1 PE=1 SV=2	CTNND1	105 kDa	0.066	1.8	[]	27	34	52	47
Chaperonin containing TCP1, subunit 2 (Beta), isoform CRA_b OS=Homo sapiens GN=HEL-S-100n PE=2 SV=1	HEL-S-100n	57 kDa	0.073	0.3	[]	52	84	18	16
Polyubiquitin-C (Fragment) OS=Homo sapiens GN=UBC PE=1 SV=1	UBC	17 kDa	0.6	1.2	[]	35	38	28	48
Enolase 1, (Alpha), isoform CRA_a OS=Homo sapiens GN=ENO1 PE=2 SV=1	ENO1	47 kDa	0.0014	0.3	Fibro-BKO low, Fibro-Naive high	63	66	15	19
Epididymis luminal protein 220 OS=Homo sapiens GN=HEL-S-70 PE=2 SV=1	HEL-S-70	89 kDa	0.012	1.3	Fibro-BKO high, Fibro-Naive low	32	33	36	40
Histone H3 OS=Homo sapiens GN=H3F3A PE=2 SV=1	H3F3A	15 kDa	0.0084	0.5	Fibro-BKO low, Fibro-Naive high	51	55	27	25
cDNA FLJ54370, highly similar to Heat shock 70 kDa protein 1 OS=Homo sapiens PE=2 SV=1		67 kDa	0.0042	0.6	Fibro-BKO low, Fibro-Naive high	47	48	25	26
Annexin (Fragment) OS=Homo sapiens GN=ANXA4 PE=2 SV=1	ANXA4	36 kDa	0.029	0.3	Fibro-BKO low, Fibro-Naive high	55	47	14	17
cDNA FLJ53573, highly similar to Myosin Ic OS=Homo sapiens PE=2 SV=1		119 kDa	0.31	0.9	[]	30	39	25	28
Annexin OS=Homo sapiens GN=HEL-S-7 PE=2 SV=1	HEL-S-7	36 kDa	0.1	0.4	[]	52	38	16	21
Agrin OS=Homo sapiens GN=AGRN PE=1 SV=1	AGRN	202 kDa	0.36	2.1	[]	3	42	46	36
Actinin alpha 4 isoform 1 (Fragment) OS=Homo sapiens GN=ACTN4 PE=2 SV=1	ACTN4	105 kDa	0.024	0.6	Fibro-BKO low, Fibro-Naive high	51	61	28	32
Elongation factor 1-alpha (Fragment) OS=Homo sapiens PE=2 SV=1		50 kDa	0.16	0.5	[]	80	52	27	25
Tetraspanin (Fragment) OS=Homo sapiens PE=2 SV=1		26 kDa	0.044	0.4	Fibro-BKO low, Fibro-Naive high	31	31	10	14
Annexin OS=Homo sapiens GN=ANXA1 PE=2 SV=1	ANXA1	39 kDa	0.76	0.9	[]	24	45	26	29
HCG1991735, isoform CRA_a OS=Homo sapiens GN=hCG_1991735 PE=4 SV=1	hCG_1991735	189 kDa	0.28	0.8	[]	29	36	17	27
Myosin, heavy polypeptide 9, non-muscle, isoform CRA_a OS=Homo sapiens GN=MYH9 PE=3 SV=1	MYH9	227 kDa	0.33	1.1	[]	38	42	35	44
Poly [ADP-ribose] polymerase OS=Homo sapiens PE=2 SV=1		113 kDa	0.083	0.4	[]	31	47	15	9
Epithelial cell adhesion molecule OS=Homo sapiens GN=EPCAM PE=1 SV=1	EPCAM	38 kDa	0.61	0.9	[]	34	28	21	25
Catenin (Cadherin-associated protein), beta 1, 88kDa, isoform CRA_a OS=Homo sapiens GN=CTNNB1 PE=4 SV=1	CTNNB1	85 kDa	0.15	1.3	[]	25	35	33	36
4F2 cell-surface antigen heavy chain OS=Homo sapiens GN=SLC3A2 PE=1 SV=1	SLC3A2	65 kDa	0.48	1.1	[]	25	30	30	25
cDNA, FLJ94213, highly similar to Homo sapiens pregnancy-zone protein (PZP), mRNA OS=Homo sapiens PE=2 SV=1		164 kDa	0.0038	4	Fibro-BKO high, Fibro-Naive low	9	12	37	37
Testicular secretory protein Li 63 OS=Homo sapiens GN=UBE1 PE=2 SV=1	UBE1	118 kDa	0.0094	0.5	Fibro-BKO low, Fibro-Naive high	35	41	14	17
Integrin alpha-2 OS=Homo sapiens GN=ITGA2 PE=1 SV=1	ITGA2	103 kDa	0.98	1	[]	18	35	22	25
Lectin galactoside-binding soluble 3 binding protein isoform 1 (Fragment) OS=Homo sapiens GN=LGALS3BP PE=2 SV=1	LGALS3BP	65 kDa	0.16	1.2	[]	89	90	94	89
EEF2 protein (Fragment) OS=Homo sapiens GN=EEF2 PE=2 SV=1	EEF2	65 kDa	0.028	0.6	Fibro-BKO low, Fibro-Naive high	29	30	12	17
Aldehyde dehydrogenase 1 family, member A1, isoform CRA_a OS=Homo sapiens GN=HEL-S-53e PE=2 SV=1	HEL-S-53e	55 kDa	0.049	0.6	Fibro-BKO low, Fibro-Naive high	32	29	17	17
Heat shock protein 90kDa alpha (Cytosolic), class B member 1, isoform CRA_a OS=Homo sapiens GN=HSP90AB1 PE=3 SV	HSP90AB1	83 kDa	0.018	0.7	Fibro-BKO low, Fibro-Naive high	42	42	23	27
Galectin OS=Homo sapiens GN=LGALS4 PE=2 SV=1	LGALS4	36 kDa	0.038	0.5	Fibro-BKO low, Fibro-Naive high	25	31	11	13
Collagen alpha-1(VI) chain OS=Homo sapiens GN=COL6A1 PE=1 SV=1	COL6A1	108 kDa	0.015	14	Fibro-BKO high, Fibro-Naive low	3	3	41	35
Sodium/potassium-transporting ATPase subunit alpha (Fragment) OS=Homo sapiens PE=2 SV=1		112 kDa	0.11	0.7	[]	41	54	28	32
Chaperonin containing TCP1, subunit 8 (Theta) variant (Fragment) OS=Homo sapiens PE=2 SV=1		60 kDa	0.29	0.8	[]	20	23	17	12
Protocadherin Fat 1 OS=Homo sapiens GN=FAT1 PE=1 SV=1	FAT1	506 kDa	0.0067	9.4	Fibro-BKO high, Fibro-Naive low	2	6	30	33
SNRNP200 protein (Fragment) OS=Homo sapiens GN=SNRNP200 PE=2 SV=1	SNRNP200	216 kDa	0.4	0.7	[]	16	28	16	11
Chaperonin containing TCP1, subunit 7 (Eta) variant (Fragment) OS=Homo sapiens PE=2 SV=1		59 kDa	0.13	0.6	[]	25	21	14	11
Cofilin 1 (Non-muscle), isoform CRA_b OS=Homo sapiens GN=HEL-S-15 PE=2 SV=1	HEL-S-15	19 kDa	0.16	0.6	[]	31	23	14	15
T-complex protein 1 subunit delta OS=Homo sapiens PE=2 SV=1		58 kDa	0.14	0.5	[]	32	23	12	14
Isoleucyl-tRNA synthetase, cytoplasmic variant (Fragment) OS=Homo sapiens PE=2 SV=1		146 kDa	0.39	0.9	[]	18	17	14	15
Peptidyl-prolyl cis-trans isomerase OS=Homo sapiens PE=2 SV=1		18 kDa	0.11	0.4	[]	34	23	9	10
Histone H2A OS=Homo sapiens GN=H2AFY PE=1 SV=1	H2AFY	18 kDa	0.033	0.2	Fibro-BKO low, Fibro-Naive high	71	59	17	11
Keratin 18, isoform CRA_a OS=Homo sapiens GN=KRT18 PE=3 SV=1	KRT18	48 kDa	0.13	0.3	[]	30	20	7	9
Tetraspanin OS=Homo sapiens GN=CD81 PE=3 SV=1	CD81	26 kDa	0.3	0.7	[]	28	19	16	13
Chaperonin containing TCP1, subunit 6A (Zeta 1), isoform CRA_a OS=Homo sapiens GN=CCT6A PE=3 SV=1	CCT6A	58 kDa	0.33	0.6	[]	19	29	8	19

Bumetanide-sensitive Na-K-Cl cotransporter OS=Homo sapiens GN=SLC12A2 PE=2 SV=1	SLC12A2	131 kDa	0.26	1.6	[]	12	18	24	17
GNAS complex locus isoform 1 (Fragment) OS=Homo sapiens GN=GNAS PE=2 SV=1	GNAS	46 kDa	0.14	1.7	[]	16	14	25	20
Cell division cycle 42 (GTP binding protein, 25kDa), isoform CRA_a OS=Homo sapiens GN=CDC42 PE=4 SV=1	CDC42	21 kDa	0.044	0.4	Fibro-BKO low, Fibro-Naive high	24	21	6	10
Suppressor of tumorigenicity 14 protein homolog OS=Homo sapiens PE=2 SV=1		95 kDa	0.33	1.3	[]	12	22	20	20
T-complex protein 1 subunit gamma OS=Homo sapiens PE=2 SV=1		58 kDa	0.087	0.7	[]	20	18	11	13
Barrier to autointegration factor 1, isoform CRA_a OS=Homo sapiens GN=BANF1 PE=4 SV=1	BANF1	10 kDa	0.19	0.05	[]	33	12	0	2
Collagen alpha-1(XII) chain OS=Homo sapiens GN=COL12A1 PE=1 SV=1	COL12A1	333 kDa	0.00087	INF	Fibro-BKO high, Fibro-Naive low	0	0	84	96
ADAM metallopeptidase domain 10, isoform CRA_b OS=Homo sapiens GN=ADAM10 PE=4 SV=1	ADAM10	84 kDa	0.3	1.2	[]	13	19	18	17
Epididymis secretory protein Li 45 OS=Homo sapiens GN=HEL-S-45 PE=2 SV=1	HEL-S-45	77 kDa	0.16	0.7	[]	18	24	14	13
IgGFc-binding protein OS=Homo sapiens GN=FCGBP PE=1 SV=1	FCGBP	445 kDa	0.0026	INF	Fibro-BKO high, Fibro-Naive low	0	0	36	35
Epididymis tissue sperm binding protein Li 3a OS=Homo sapiens PE=2 SV=1		35 kDa	0.048	0.4	Fibro-BKO low, Fibro-Naive high	31	25	9	10
Myosin-14 (Fragment) OS=Homo sapiens GN=MYH14 PE=1 SV=8	MYH14	114 kDa	0.017	0.5	Fibro-BKO low, Fibro-Naive high	30	35	15	13
Epididymis luminal protein 33 OS=Homo sapiens GN=HEL-S-72p PE=2 SV=1	HEL-S-72p	71 kDa	0.26	0.8	[]	67	60	48	48
60S ribosomal protein L6 OS=Homo sapiens GN=RPL6 PE=3 SV=1	RPL6	33 kDa	0.037	0.3	Fibro-BKO low, Fibro-Naive high	25	21	5	8
Ezrin OS=Homo sapiens GN=EZR PE=1 SV=3	EZR	69 kDa	0.95	1	[]	22	24	15	27
PTGFRN protein (Fragment) OS=Homo sapiens GN=PTGFRN PE=2 SV=1	PTGFRN	88 kDa	0.088	2.1	[]	7	15	21	20
Proteasome 26S non-ATPase subunit 2 variant (Fragment) OS=Homo sapiens PE=2 SV=1		101 kDa	0.37	1.2	[]	15	17	14	19
L-lactate dehydrogenase OS=Homo sapiens GN=HEL-S-133P PE=2 SV=1	HEL-S-133P	37 kDa	0.003	0.3	Fibro-BKO low, Fibro-Naive high	22	24	7	6
Phosphoglycerate kinase OS=Homo sapiens GN=HEL-S-68p PE=2 SV=1	HEL-S-68p	45 kDa	0.35	0.9	[]	20	19	16	15
sp|K1C10_HUMAN|		59 kDa	0.65	1.5	[]	12	13	6	28
Epididymis luminal protein 4 OS=Homo sapiens GN=YWHAZ PE=2 SV=1	YWHAZ	28 kDa	0.031	0.5	Fibro-BKO low, Fibro-Naive high	22	20	10	10
Fructose-bisphosphate aldolase OS=Homo sapiens GN=HEL-S-87p PE=2 SV=1	HEL-S-87p	39 kDa	0.16	0.7	[]	23	21	11	14
Tetraspanin OS=Homo sapiens GN=CD9 PE=1 SV=1	CD9	18 kDa	0.037	1.8	Fibro-BKO high, Fibro-Naive low	13	11	20	19
Collagen alpha-2(I) chain OS=Homo sapiens GN=COL1A2 PE=1 SV=1	COL1A2	129 kDa	0.0016	INF	Fibro-BKO high, Fibro-Naive low	0	0	30	35
Histone 1, H1e OS=Homo sapiens GN=HIST1H1E PE=2 SV=1	HIST1H1E	22 kDa	0.052	0.1	[]	28	20	2	4
T-complex protein 1 subunit alpha OS=Homo sapiens GN=TCP1 PE=1 SV=1	TCP1	44 kDa	0.017	0.4	Fibro-BKO low, Fibro-Naive high	26	24	8	10
Flotillin 1 variant (Fragment) OS=Homo sapiens PE=2 SV=1		47 kDa	0.21	1.6	[]	10	15	20	15
ITGAV protein OS=Homo sapiens GN=ITGAV PE=2 SV=1	ITGAV	116 kDa	0.13	1.5	[]	11	14	14	19
CD133 isoform H OS=Homo sapiens GN=CD133 PE=2 SV=1	CD133	96 kDa	0.09	2.9	[]	3	13	19	21
Fibronectin 1, isoform CRA_n OS=Homo sapiens GN=FN1 PE=4 SV=1	FN1	259 kDa	0.001	44	Fibro-BKO high, Fibro-Naive low	11	25	676	688
Glucosaminyl (N-acetyl) transferase 3, mucin type, isoform CRA_a OS=Homo sapiens GN=GCNT3 PE=4 SV=1	GCNT3	51 kDa	0.87	1	[]	16	15	13	15
Chromosome 9 open reading frame 88, isoform CRA_a OS=Homo sapiens GN=C9orf88 PE=4 SV=1	C9orf88	83 kDa	0.48	1.2	[]	10	15	14	12
Immunoblobulin light chain (Fragment) OS=Homo sapiens PE=1 SV=1		24 kDa	0.19	64	[]	1	0	14	46
ATP-citrate synthase OS=Homo sapiens GN=ACLY PE=3 SV=1	ACLY	121 kDa	0.16	1.4	[]	13	11	13	17
Glucose-6-phosphate 1-dehydrogenase OS=Homo sapiens PE=2 SV=1		59 kDa	0.13	0.4	[]	25	16	5	8
Tetraspanin (Fragment) OS=Homo sapiens GN=KAI1 PE=2 SV=1	KAI1	30 kDa	0.6	0.9	[]	18	12	11	12
Collagen, type VI, alpha 3 OS=Homo sapiens GN=COL6A3 PE=4 SV=1	COL6A3	344 kDa	0.00011	97	Fibro-BKO high, Fibro-Naive low	0	5	200	213
cDNA FLJ44754 fis, clone BRACE3030748, highly similar to Guanine nucleotide-binding protein G(i), alpha-2 subunit OS=Homo sapiens PE=2		35 kDa	0.48	1.2	[]	14	15	17	13
Dopamine receptor interacting protein 4 OS=Homo sapiens GN=DRIP4 PE=2 SV=1	DRIP4	96 kDa	0.05	1.3	[]	48	59	62	62
Laminin, gamma 1 (Formerly LAMB2), isoform CRA_a OS=Homo sapiens GN=LAMC1 PE=4 SV=1	LAMC1	174 kDa	0.0047	67	Fibro-BKO high, Fibro-Naive low	0	1	26	32
Triosephosphate isomerase OS=Homo sapiens GN=HEL-S-49 PE=2 SV=1	HEL-S-49	27 kDa	0.18	0.5	[]	23	14	7	8
RuvB-like helicase (Fragment) OS=Homo sapiens GN=RUVBL1 PE=2 SV=1	RUVBL1	50 kDa	0.016	0.3	Fibro-BKO low, Fibro-Naive high	21	19	6	6
Glucose-6-phosphate isomerase (Fragment) OS=Homo sapiens GN=GPI PE=1 SV=1	GPI	65 kDa	0.081	0.4	[]	21	16	5	8
cDNA FLJ78483, highly similar to Homo sapiens elongation factor Tu GTP binding domain containing 2 (EFTUD2), mRNA OS=Homo sapiens P		109 kDa	0.0062	0.3	Fibro-BKO low, Fibro-Naive high	20	20	4	6
Annexin OS=Homo sapiens PE=2 SV=1		46 kDa	0.046	0.9	Fibro-BKO low, Fibro-Naive high	14	15	11	11
Protein arginine N-methyltransferase 1 OS=Homo sapiens GN=PRMT1 PE=1 SV=1	PRMT1	38 kDa	0.11	0.5	[]	19	15	9	7
EPRS protein (Fragment) OS=Homo sapiens GN=EPRS PE=2 SV=1	EPRS	110 kDa	0.86	1	[]	10	17	11	14
Keratin 1 OS=Homo sapiens GN=KRT1 PE=3 SV=1	KRT1	66 kDa	0.92	0.9	[]	12	19	5	21
Vacuolar protein sorting 37B (Yeast), isoform CRA_b OS=Homo sapiens GN=VPS37B PE=4 SV=1	VPS37B	31 kDa	0.14	1.2	[]	11	12	13	12
Proteasome subunit alpha type OS=Homo sapiens GN=HEL-S-276 PE=2 SV=1	HEL-S-276	28 kDa	0.1	1.6	[]	10	8	11	14
Programmed cell death 6 variant (Fragment) OS=Homo sapiens PE=2 SV=1		22 kDa	0.093	0.5	[]	28	20	11	11
Epididymis secretory protein Li 1 OS=Homo sapiens GN=HEL-S-1 PE=2 SV=1	HEL-S-1	28 kDa	0.16	0.8	[]	13	14	9	12
SF3B1 protein (Fragment) OS=Homo sapiens GN=SF3B1 PE=2 SV=1	SF3B1	87 kDa	0.018	0.3	Fibro-BKO low, Fibro-Naive high	15	19	5	4
EH domain-containing protein 1 OS=Homo sapiens GN=EHD1 PE=1 SV=1	EHD1	62 kDa	0.013	1.7	Fibro-BKO high, Fibro-Naive low	10	12	17	17
EH-domain containing 4, isoform CRA_a OS=Homo sapiens GN=EHD4 PE=3 SV=1	EHD4	61 kDa	0.054	1.5	[]	9	12	14	14
RAB7, member RAS oncogene family, isoform CRA_a OS=Homo sapiens GN=RAB7A PE=2 SV=1	RAB7A	23 kDa	0.51	0.8	[]	14	13	6	13
cDNA, FLJ93545, highly similar to Homo sapiens 5-aminoimidazole-4-carboxamide ribonucleotide formyltransferase/IMP cyclohydrolase (AT		65 kDa	0.0037	0.8	Fibro-BKO low, Fibro-Naive high	13	14	9	10
cDNA FLJ54622, highly similar to Prothrombin (EC 3.4.21.5) OS=Homo sapiens PE=2 SV=1		53 kDa	0.0036	3	Fibro-BKO high, Fibro-Naive low	6	7	17	17
Cytoplasmic FMR1 interacting protein 1 isoform A (Fragment) OS=Homo sapiens GN=CYFIP1 PE=2 SV=1	CYFIP1	145 kDa	0.18	1.4	[]	10	9	10	14

Putative uncharacterized protein XRCC5 (Fragment) OS=Homo sapiens GN=XRCC5 PE=4 SV=1	XRCC5	64 kDa	0.063	0.3	[]	17	15	6	2
cDNA, FLJ95508, highly similar to Homo sapiens 5'-nucleotidase, ecto (CD73) (NT5E), mRNA OS=Homo sapiens PE=2 SV=1		63 kDa	0.48	0.9	[]	11	12	10	8
Lipolysis-stimulated lipoprotein receptor OS=Homo sapiens GN=LSR PE=1 SV=2	LSR	66 kDa	0.3	1.5	[]	9	13	11	19
Apolipoprotein B (Including Ag(X) antigen) OS=Homo sapiens GN=APOB PE=4 SV=1	APOB	516 kDa	0.062	2.8	[]	3	6	12	10
cDNA FLJ61188, highly similar to Basigin OS=Homo sapiens PE=2 SV=1		28 kDa	0.88	1	[]	12	12	9	12
Integrin alpha-6 (Fragment) OS=Homo sapiens GN=ITGA6 PE=1 SV=1	ITGA6	26 kDa	0.25	2	[]	6	9	9	18
40S ribosomal protein S8 OS=Homo sapiens GN=RPS8 PE=2 SV=1	RPS8	24 kDa	0.29	0.5	[]	17	11	8	4
Proteasome subunit alpha type OS=Homo sapiens GN=PSMA2 PE=1 SV=1	PSMA2	26 kDa	0.29	1.1	[]	11	12	12	11
Coagulation factor V OS=Homo sapiens GN=F5 PE=1 SV=1	F5	252 kDa	0.16	2.7	[]	7	4	16	10
cDNA FLJ76654, highly similar to Homo sapiens CD44 antigen (homing function and Indian blood group system) (CD44), transcript variant 4,		39 kDa	0.83	1	[]	10	15	12	11
cDNA FLJ78677, highly similar to Homo sapiens splicing factor 3b, subunit 3, 130kDa (SF3B3), mRNA OS=Homo sapiens PE=2 SV=1		136 kDa	0.0043	0.3	Fibro-BKO low, Fibro-Naive high	17	19	5	4
Epididymis luminal secretory protein 52 OS=Homo sapiens GN=EL52 PE=2 SV=1	EL52	85 kDa	0.23	0.6	[]	17	28	11	14
Epididymis luminal protein 2 OS=Homo sapiens GN=HEL2 PE=1 SV=1	HEL2	29 kDa	0.054	0.4	[]	16	13	5	6
Epididymis secretory protein Li 22 OS=Homo sapiens GN=HEL-S-22 PE=2 SV=1	HEL-S-22	23 kDa	0.0002	0.3	Fibro-BKO low, Fibro-Naive high	26	28	7	8
Protein tweety homolog OS=Homo sapiens GN=TTYH3 PE=3 SV=1	TTYH3	58 kDa	0.014	1.5	Fibro-BKO high, Fibro-Naive low	9	10	12	14
Chloride intracellular channel protein (Fragment) OS=Homo sapiens PE=2 SV=1		27 kDa	0.23	0.6	[]	15	10	7	6
Transgelin-2 (Fragment) OS=Homo sapiens GN=TAGLN2 PE=1 SV=1	TAGLN2	21 kDa	0.2	0.6	[]	16	11	7	7
ARP3 actin-related protein 3 homolog (Yeast), isoform CRA_a OS=Homo sapiens GN=ACTR3 PE=3 SV=1	ACTR3	47 kDa	0.0011	0.2	Fibro-BKO low, Fibro-Naive high	16	17	3	4
Ferritin OS=Homo sapiens GN=FTH1 PE=3 SV=1	FTH1	21 kDa	0.0061	1.5	Fibro-BKO high, Fibro-Naive low	8	8	10	11
cDNA FLJ76789, highly similar to Homo sapiens methionine-tRNA synthetase (MARS), mRNA OS=Homo sapiens PE=2 SV=1		101 kDa	0.15	0.7	[]	15	13	7	10
Adenylyl cyclase-associated protein OS=Homo sapiens PE=2 SV=1		52 kDa	0.0018	0.3	Fibro-BKO low, Fibro-Naive high	15	17	4	4
WD repeat domain 1, isoform CRA_a (Fragment) OS=Homo sapiens GN=WDR1 PE=2 SV=1	WDR1	66 kDa	0.49	0.9	[]	10	15	9	10
Gelsolin OS=Homo sapiens GN=GSN PE=1 SV=1	GSN	83 kDa	0.41	1.1	[]	10	11	9	12
Testicular tissue protein Li 192 OS=Homo sapiens PE=2 SV=1		57 kDa	0.55	0.9	[]	10	9	6	9
MARCKS protein (Fragment) OS=Homo sapiens GN=MARCKS PE=2 SV=1	MARCKS	15 kDa	0.39	1.5	[]	7	10	8	15
Histone H2A OS=Homo sapiens GN=H2AFY PE=1 SV=1	H2AFY	22 kDa	0.055	0	[]	20	13	0	0
cDNA FLJ77762, highly similar to Homo sapiens cullin-associated and neddylation-dissociated 1 (CAND1), mRNA OS=Homo sapiens PE=2 SV=		136 kDa	0.2	0.7	[]	10	15	7	8
cDNA, FLJ95650, highly similar to Homo sapiens karyopherin (importin) beta 1 (KPNB1), mRNA OS=Homo sapiens PE=2 SV=1		97 kDa	0.11	0.7	[]	12	12	8	6
COPG protein (Fragment) OS=Homo sapiens GN=COPG PE=2 SV=2	COPG	78 kDa	0.13	0.5	[]	19	16	10	6
Solute carrier family 44, member 1, isoform CRA_a OS=Homo sapiens GN=SLC44A1 PE=3 SV=1	SLC44A1	73 kDa	0.068	4.1	[]	4	4	17	12
Testicular tissue protein Li 96 OS=Homo sapiens PE=2 SV=1		117 kDa	0.29	1.6	[]	6	6	6	11
Ras-related C3 botulinum toxin substrate 1 (Rho family, small GTP binding protein Rac1) OS=Homo sapiens GN=RAC1 PE	RAC1	23 kDa	0.54	1.1	[]	10	9	8	11
40S ribosomal protein S4 OS=Homo sapiens GN=RPS4X PE=2 SV=1	RPS4X	30 kDa	0.35	0.6	[]	12	7	5	6
cDNA FLJ39263 fis, clone OCBBF2009571, highly similar to ATP-dependent RNA helicase A (EC 3.6.1.-) OS=Homo sapiens PE=2 SV=1		60 kDa	0.27	0.6	[]	15	9	5	7
Laminin subunit beta-1 OS=Homo sapiens GN=LAMB1 PE=1 SV=1	LAMB1	200 kDa	0.0083	INF	Fibro-BKO high, Fibro-Naive low	0	0	17	22
Vinculin, isoform CRA_c OS=Homo sapiens GN=VCL PE=4 SV=1	VCL	117 kDa	0.07	2.5	[]	4	8	14	12
cDNA FLJ56274, highly similar to Transketolase (EC 2.2.1.1) OS=Homo sapiens PE=2 SV=1		63 kDa	0.19	0.6	[]	10	15	7	7
Epididymis secretory sperm binding protein Li 62p OS=Homo sapiens GN=HEL-S-62p PE=2 SV=1	HEL-S-62p	187 kDa	0.076	2.4	[]	7	4	13	11
Epididymis luminal protein 70 OS=Homo sapiens GN=HEL70 PE=2 SV=1	HEL70	68 kDa	0.86	1	[]	15	17	11	19
Ribosomal protein L7, isoform CRA_a OS=Homo sapiens GN=RPL7 PE=4 SV=1	RPL7	30 kDa	0.01	0.2	Fibro-BKO low, Fibro-Naive high	15	14	3	2
Valyl-tRNA synthetase, isoform CRA_a OS=Homo sapiens GN=VARS PE=3 SV=1	VARS	140 kDa	0.17	0.7	[]	12	11	8	6
Glycyl-tRNA synthetase OS=Homo sapiens GN=GARS PE=4 SV=1	GARS	78 kDa	0.057	0.9	[]	10	11	8	8
EEF1G protein OS=Homo sapiens GN=EEF1G PE=2 SV=1	EEF1G	50 kDa	0.15	0.7	[]	12	10	7	7
L-lactate dehydrogenase OS=Homo sapiens GN=LDHB PE=2 SV=1	LDHB	37 kDa	0.037	0.5	Fibro-BKO low, Fibro-Naive high	13	17	6	7
sp|TRYP_PIG|		24 kDa	0.54	0.8	[]	12	8	5	9
NAD(P)H dehydrogenase [quinone] 1 OS=Homo sapiens GN=NQO1 PE=1 SV=1	NQO1	23 kDa	0.087	0.3	[]	19	13	3	5
GTP-binding nuclear protein Ran OS=Homo sapiens GN=RAN PE=1 SV=1	RAN	26 kDa	0.028	0.3	Fibro-BKO low, Fibro-Naive high	14	12	4	4
2',3'-cyclic-nucleotide 3'-phosphodiesterase OS=Homo sapiens GN=CNP PE=3 SV=1	CNP	45 kDa	0.95	1	[]	10	9	9	8
GNB2 protein OS=Homo sapiens GN=GNB2 PE=2 SV=1	GNB2	37 kDa	0.029	1.3	Fibro-BKO high, Fibro-Naive low	11	12	13	13
Mutant hemoglobin alpha 2 globin chain OS=Homo sapiens GN=HBA2 PE=3 SV=1	HBA2	15 kDa	0.15	1.8	[]	6	5	7	11
cDNA FLJ44920 fis, clone BRAMY3011501, highly similar to Heterogeneous nuclear ribonucleoprotein U OS=Homo sapiens PE=2 SV=1		83 kDa	0.0074	0.1	Fibro-BKO low, Fibro-Naive high	15	14	1	2
cDNA FLJ61290, highly similar to Neutral alpha-glucosidase AB OS=Homo sapiens PE=2 SV=1		113 kDa	0.74	0.9	[]	42	23	27	25
Rab GDP dissociation inhibitor OS=Homo sapiens PE=2 SV=1		51 kDa	0.019	0.6	Fibro-BKO low, Fibro-Naive high	24	23	12	12
Adenosylhomocysteinase OS=Homo sapiens GN=AHCY PE=2 SV=1	AHCY	34 kDa	0.39	0.7	[]	14	9	7	8
DHX9 protein (Fragment) OS=Homo sapiens GN=DHX9 PE=2 SV=1	DHX9	67 kDa	0.043	0.2	Fibro-BKO low, Fibro-Naive high	17	13	3	3
sp|K1C9_HUMAN|		62 kDa	0.93	1.1	[]	10	5	2	13
X-ray repair complementing defective repair in Chinese hamster cells 6 (Ku autoantigen, 70kDa), isoform CRA_a OS=Hor	XRCC6	70 kDa	0.022	0.1	Fibro-BKO low, Fibro-Naive high	17	14	1	2
Guanine nucleotide binding protein (G protein), beta polypeptide 1, isoform CRA_a OS=Homo sapiens GN=GNB1 PE=2 S	GNB1	37 kDa	0.037	1.2	Fibro-BKO high, Fibro-Naive low	12	12	12	13
cDNA FLJ75881, highly similar to Homo sapiens transferrin receptor (p90, CD71) (TFRC), mRNA OS=Homo sapiens PE=2 SV=1		85 kDa	0.26	0.8	[]	11	10	8	6

Protein phosphatase 2 (Formerly 2A), regulatory subunit A (PR 65), alpha isoform OS=Homo sapiens GN=PPP2R1A PE=1	PPP2R1A	65 kDa	0.059	0.5	[]	11	10	4	6
Arrestin domain-containing protein 1 (Fragment) OS=Homo sapiens GN=ARRDC1 PE=1 SV=1	ARRDC1	29 kDa	0.26	1.2	[]	9	12	11	11
Complement C4-B OS=Homo sapiens GN=C4B_2 PE=1 SV=1	C4B_2	188 kDa	0.076	4.3	[]	3	4	10	15
Copine-1 OS=Homo sapiens GN=CPNE1 PE=1 SV=1	CPNE1	53 kDa	0.012	0.4	Fibro-BKO low, Fibro-Naive high	13	13	5	4
T-complex protein 1 subunit alpha OS=Homo sapiens GN=TCP1 PE=1 SV=1	TCP1	47 kDa	0.19	0.6	[]	17	13	7	10
40S ribosomal protein SA OS=Homo sapiens GN=RPSA PE=3 SV=1	RPSA	33 kDa	0.073	0.3	[]	16	12	2	5
HCG1998851, isoform CRA_c OS=Homo sapiens GN=hCG_1998851 PE=4 SV=1	hCG_1998851	47 kDa	0.45	0.9	[]	7	7	6	5
EPH receptor A2, isoform CRA_a OS=Homo sapiens GN=EPHA2 PE=3 SV=1	EPHA2	108 kDa	0.026	2.6	Fibro-BKO high, Fibro-Naive low	5	4	11	10
Cadherin-1 OS=Homo sapiens GN=CDH1 PE=1 SV=1	CDH1	100 kDa	0.77	0.9	[]	10	8	6	9
AP complex subunit beta OS=Homo sapiens GN=AP2B1 PE=2 SV=1	AP2B1	106 kDa	0.12	0.7	[]	19	17	11	13
Proteasome subunit beta type OS=Homo sapiens PE=2 SV=1		26 kDa	0.0033	1.8	Fibro-BKO high, Fibro-Naive low	6	7	10	11
Integrin beta-1 (Fragment) OS=Homo sapiens GN=ITGB1 PE=1 SV=1	ITGB1	11 kDa	0.3	1.6	[]	5	9	11	8
Ribosomal protein L15 OS=Homo sapiens GN=RPL15 PE=3 SV=1	RPL15	24 kDa	0.23	0.4	[]	12	6	3	3
H3L-like histone (Fragment) OS=Homo sapiens PE=1 SV=1		12 kDa	0.099	0	[]	18	9	0	0
Phosphoribosylaminoimidazole carboxylase, phosphoribosylaminoimidazole succinocarboxamide synthetase, isoform C	PAICS	47 kDa	0.093	0.5	[]	11	10	6	4
Tetraspanin (Fragment) OS=Homo sapiens GN=TSPAN14 PE=1 SV=2	TSPAN14	25 kDa	0.66	1.2	[]	10	5	8	8
cDNA, FLJ96156, highly similar to Homo sapiens leucyl-tRNA synthetase (LARS), mRNA OS=Homo sapiens PE=2 SV=1		134 kDa	0.52	0.8	[]	10	7	7	5
Eukaryotic translation initiation factor 3 subunit A OS=Homo sapiens GN=eIF3a PE=2 SV=1	eIF3a	166 kDa	0.099	0.5	[]	11	9	5	3
cDNA, FLJ94599, highly similar to Homo sapiens GDP-mannose 4,6-dehydratase (GMDS), mRNA OS=Homo sapiens PE=2 SV=1		42 kDa	0.041	0.2	Fibro-BKO low, Fibro-Naive high	14	11	3	2
Heat shock 105kDa/110kDa protein 1, isoform CRA_a OS=Homo sapiens GN=HSPH1 PE=3 SV=1	HSPH1	92 kDa	0.024	0.4	Fibro-BKO low, Fibro-Naive high	11	11	3	5
Serpin peptidase inhibitor, clade E (Nexin, plasminogen activator inhibitor type 1), member 2, isoform CRA_b OS=Homo	SERPINE2	44 kDa	0.0028	12	Fibro-BKO high, Fibro-Naive low	2	1	16	16
Solute carrier family 44, member 2, isoform CRA_a OS=Homo sapiens GN=SLC44A2 PE=3 SV=1	SLC44A2	80 kDa	0.14	0.9	[]	6	7	5	5
Milk fat globule-EGF factor 8 protein, isoform CRA_a OS=Homo sapiens GN=MFGE8 PE=4 SV=1	MFGE8	43 kDa	0.00013	6.5	Fibro-BKO high, Fibro-Naive low	12	13	67	74
Immunoglobulin superfamily member 8 (Fragment) OS=Homo sapiens GN=IGSF8 PE=1 SV=1	IGSF8	32 kDa	0.011	1.8	Fibro-BKO high, Fibro-Naive low	5	6	9	9
DIP2B protein (Fragment) OS=Homo sapiens GN=DIP2B PE=2 SV=2	DIP2B	102 kDa	0.036	4.8	Fibro-BKO high, Fibro-Naive low	1	5	13	12
RuvB-like helicase OS=Homo sapiens GN=RUVBL2 PE=2 SV=1	RUVBL2	47 kDa	0.22	0.5	[]	14	8	4	5
cDNA FLJ54047, highly similar to Alpha-1 catenin (Cadherin-associated protein) OS=Homo sapiens PE=2 SV=1		98 kDa	0.042	1.4	Fibro-BKO high, Fibro-Naive low	51	61	65	76
Tubulointerstitial nephritis antigen-like OS=Homo sapiens GN=TINAGL1 PE=1 SV=1	TINAGL1	41 kDa	0.75	1.2	[]	23	10	15	19
Collagen, type I, alpha 1, isoform CRA_a OS=Homo sapiens GN=COL1A1 PE=4 SV=1	COL1A1	85 kDa	0.0029	INF	Fibro-BKO high, Fibro-Naive low	0	0	25	30
Collagen alpha-1(I) chain preproprotein OS=Homo sapiens GN=COL1A1 PE=2 SV=1	COL1A1	100 kDa	0.021	INF	Fibro-BKO high, Fibro-Naive low	0	0	22	32
Sodium/potassium-transporting ATPase subunit beta OS=Homo sapiens GN=ATP1B1 PE=2 SV=1	ATP1B1	35 kDa	0.044	0.6	Fibro-BKO low, Fibro-Naive high	7	9	4	4
cDNA FLJ78449 OS=Homo sapiens PE=2 SV=1		40 kDa	0.3	1.3	[]	7	7	9	7
H/ACA ribonucleoprotein complex subunit 4 (Fragment) OS=Homo sapiens GN=DKC1 PE=1 SV=8	DKC1	30 kDa	0.14	0.1	[]	16	8	1	2
Splicing factor 3B subunit 1 (Fragment) OS=Homo sapiens GN=SF3B1 PE=1 SV=1	SF3B1	27 kDa	0.018	0.3	Fibro-BKO low, Fibro-Naive high	11	13	2	4
Staphylococcal nuclease domain containing 1, isoform CRA_b OS=Homo sapiens GN=SND1 PE=2 SV=1	SND1	102 kDa	0.12	0.3	[]	8	13	1	3
Low density lipoprotein-related protein 1 variant (Fragment) OS=Homo sapiens PE=2 SV=1		262 kDa	0.015	30	Fibro-BKO high, Fibro-Naive low	0	1	14	12
Proteasome subunit beta type OS=Homo sapiens PE=2 SV=1		23 kDa	0.64	1.2	[]	8	4	7	6
cDNA FLJ50510, highly similar to Heat shock 70 kDa protein 4 OS=Homo sapiens PE=2 SV=1		94 kDa	0.28	0.7	[]	7	12	5	6
Epididymis secretory protein Li 69 OS=Homo sapiens GN=HEL-S-69 PE=1 SV=1	HEL-S-69	60 kDa	0.0022	0.5	Fibro-BKO low, Fibro-Naive high	34	34	16	17
Proteasome (Prosome, macropain) 26S subunit, non-ATPase, 6, isoform CRA_c OS=Homo sapiens GN=PSMD6 PE=2 SV=1	PSMD6	40 kDa	0.047	0.4	Fibro-BKO low, Fibro-Naive high	10	12	5	3
Unconventional myosin-Id OS=Homo sapiens GN=MYO1D PE=1 SV=1	MYO1D	111 kDa	0.044	2.5	Fibro-BKO high, Fibro-Naive low	2	4	6	7
Ribosomal protein S2 OS=Homo sapiens GN=RPS2 PE=2 SV=1	RPS2	31 kDa	0.14	0.5	[]	10	7	4	3
cDNA FLJ61587, highly similar to Integrin alpha-1 (Fragment) OS=Homo sapiens PE=2 SV=1		130 kDa	0.16	4.7	[]	1	4	13	7
Casein kinase II subunit alpha OS=Homo sapiens GN=CSNK2A1 PE=1 SV=1	CSNK2A1	45 kDa	0.05	0.05	Fibro-BKO low, Fibro-Naive high	13	9	0	1
COL6A2 protein OS=Homo sapiens PE=2 SV=1		47 kDa	0.0025	33	Fibro-BKO high, Fibro-Naive low	0	1	14	14
N-ethylmaleimide-sensitive factor attachment protein, alpha, isoform CRA_c OS=Homo sapiens GN=NAPA PE=4 SV=1	NAPA	33 kDa	0.26	0.5	[]	12	7	5	4
RAB2, member RAS oncogene family, isoform CRA_a OS=Homo sapiens GN=RAB2 PE=4 SV=1	RAB2	24 kDa	0.25	0.6	[]	7	12	4	6
Epididymis secretory sperm binding protein Li 128m OS=Homo sapiens GN=HEL-S-128m PE=2 SV=1	HEL-S-128m	25 kDa	0.24	1.4	[]	7	5	8	7
Solute carrier family 16 (Monocarboxylic acid transporters), member 1, isoform CRA_b OS=Homo sapiens GN=SLC16A1 P	SLC16A1	54 kDa	0.34	1.8	[]	2	9	8	9
cDNA FLJ75066, highly similar to Homo sapiens complement component 1, r subcomponent (C1R), mRNA OS=Homo sapiens PE=2 SV=1		80 kDa	0.16	INF	[]	0	0	7	19
Unconventional myosin-VI OS=Homo sapiens GN=MYO6 PE=1 SV=1	MYO6	145 kDa	0.082	0.4	[]	7	11	3	3
Band 4.1-like protein 1 OS=Homo sapiens GN=EPB41L1 PE=1 SV=1	EPB41L1	98 kDa	0.13	0.7	[]	9	9	5	7
Putative uncharacterized protein Nbla00445 (Fragment) OS=Homo sapiens GN=Nbla00445 PE=2 SV=1	Nbla00445	103 kDa	0.2	1.5	[]	9	12	15	12
ADP-ribosylation factor 1, isoform CRA_a OS=Homo sapiens GN=ARF1 PE=3 SV=1	ARF1	21 kDa	0.12	0.6	[]	13	11	7	5
EPH receptor B4, isoform CRA_a OS=Homo sapiens GN=EPHB4 PE=3 SV=1	EPHB4	108 kDa	0.46	1.3	[]	5	11	8	11
Epididymis secretory sperm binding protein Li 66p OS=Homo sapiens GN=HEL-S-66p PE=2 SV=1	HEL-S-66p	33 kDa	0.026	0.4	Fibro-BKO low, Fibro-Naive high	9	10	2	4
von Willebrand factor A domain containing 1, isoform CRA_c OS=Homo sapiens GN=VWA1 PE=4 SV=1	VWA1	43 kDa	0.58	1.2	[]	7	5	5	8
Heterogeneous nuclear ribonucleoprotein K, isoform CRA_d OS=Homo sapiens GN=HNRPK PE=4 SV=1	HNRPK	51 kDa	0.18	0.6	[]	6	8	3	5
RAN binding protein 5, isoform CRA_d OS=Homo sapiens GN=RANBP5 PE=4 SV=1	RANBP5	124 kDa	0.12	0.5	[]	7	11	4	4

Basal cell adhesion molecule (Lutheran blood group) OS=Homo sapiens GN=BCAM PE=4 SV=1	BCAM	67 kDa	0.078	2.1	[]	5	4	7	10
cDNA FLJ77590, highly similar to Homo sapiens leucine rich repeat containing 1, mRNA OS=Homo sapiens PE=2 SV=1		59 kDa	0.2	1.5	[]	5	9	10	9
cDNA FLJ30049 fis, clone ADRGL1000033, highly similar to 26S proteasome non-ATPase regulatory subunit 3 OS=Homo sapiens PE=2 SV=1		57 kDa	0.087	0.8	[]	7	8	5	6
Acyl-CoA synthetase 4 OS=Homo sapiens GN=FACL4 PE=2 SV=1	FACL4	74 kDa	0.29	1.8	[]	6	4	10	6
Epididymis secretory sperm binding protein Li 89n OS=Homo sapiens GN=HEL-S-89n PE=2 SV=1	HEL-S-89n	72 kDa	0.75	1	[]	11	10	8	10
cDNA FLJ14168 fis, clone NT2RP2001440, highly similar to 14-3-3 protein gamma OS=Homo sapiens PE=2 SV=1		28 kDa	0.57	0.9	[]	6	8	6	5
S100A10 protein (Fragment) OS=Homo sapiens GN=S100A10 PE=2 SV=1	S100A10	11 kDa	0.0024	0.5	Fibro-BKO low, Fibro-Naive high	9	10	4	4
Integrin beta OS=Homo sapiens GN=ITGB5 PE=2 SV=1	ITGB5	88 kDa	0.015	4.9	Fibro-BKO high, Fibro-Naive low	1	3	8	9
Apolipoprotein A-I, isoform CRA_a OS=Homo sapiens GN=APOA1 PE=3 SV=1	APOA1	31 kDa	0.39	2.4	[]	4	2	3	10
cDNA FLJ51525, highly similar to Homo sapiens aldehyde dehydrogenase 16 family, member A1 (ALDH16A1), mRNA OS=Homo sapiens PE=2		67 kDa	0.028	0.2	Fibro-BKO low, Fibro-Naive high	9	10	3	1
Testicular tissue protein Li 164 OS=Homo sapiens PE=2 SV=1		50 kDa	0.0082	0.6	Fibro-BKO low, Fibro-Naive high	8	8	4	4
cDNA, FLJ96428, highly similar to Homo sapiens proteasome (prosome, macropain) 26S subunit, non-ATPase, 7 (Mov34 homolog) (PSMD7),		37 kDa	0.26	0.4	[]	12	6	3	4
Phospholipid scramblase OS=Homo sapiens GN=PLSCR1 PE=1 SV=1	PLSCR1	34 kDa	0.35	0.6	[]	5	10	3	5
Kinesin-like protein (Fragment) OS=Homo sapiens PE=2 SV=1		101 kDa	0.76	1.1	[]	4	7	7	4
Proteasome (Prosome, macropain) 26S subunit, ATPase, 4, isoform CRA_b OS=Homo sapiens GN=PSMC4 PE=2 SV=1	PSMC4	47 kDa	0.85	1.1	[]	5	4	7	2
26S protease regulatory subunit 10B OS=Homo sapiens GN=PSMC6 PE=1 SV=1	PSMC6	46 kDa	0.79	1.2	[]	7	3	3	6
Proteasome (Prosome, macropain) 26S subunit, non-ATPase, 13, isoform CRA_a OS=Homo sapiens GN=PSMD13 PE=4 S	PSMD13	43 kDa	0.023	0.2	Fibro-BKO low, Fibro-Naive high	7	9	2	1
Putative uncharacterized protein DKFZp686F17268 (Fragment) OS=Homo sapiens GN=DKFZp686F17268 PE=2 SV=1	DKFZp686F17268	50 kDa	0.09	0.3	[]	8	9	4	1
HCG2043275 OS=Homo sapiens GN=EEF1E1-BLOC1S5 PE=4 SV=2	EEF1E1-BLOC1S5	17 kDa	0.9	0.9	[]	6	3	4	3
Myosin light polypeptide 6 OS=Homo sapiens GN=MYL6 PE=1 SV=1	MYL6	16 kDa	0.11	0.4	[]	8	7	1	4
Proteasome (Prosome, macropain) 26S subunit, non-ATPase, 12, isoform CRA_a (Fragment) OS=Homo sapiens GN=PSM	PSMD12	53 kDa	0.11	0.7	[]	7	6	4	4
Vasodilator-stimulated phosphoprotein isoform 1 OS=Homo sapiens GN=VASP PE=2 SV=1	VASP	40 kDa	0.71	0.9	[]	6	6	4	6
Coatomer subunit beta (Fragment) OS=Homo sapiens GN=COPB1 PE=1 SV=8	COPB1	55 kDa	0.026	0.2	Fibro-BKO low, Fibro-Naive high	8	11	2	2
cDNA FLJ56822, highly similar to Alpha-2-HS-glycoprotein OS=Homo sapiens PE=2 SV=1		27 kDa	0.18	1.1	[]	6	7	6	7
sp|PRDX1_HUMAN|		22 kDa	1	1	[]	5	5	3	6
GCN1 general control of amino-acid synthesis 1-like 1 (Yeast), isoform CRA_b OS=Homo sapiens GN=GCN1L1 PE=4 SV=1	GCN1L1	267 kDa	0.18	3.1	[]	0	7	8	11
Hypoxia up-regulated protein 1 OS=Homo sapiens GN=HYOU1 PE=1 SV=1	HYOU1	105 kDa	0.051	2.3	[]	3	5	7	9
Proteasome subunit alpha type OS=Homo sapiens PE=2 SV=1		27 kDa	0.65	0.8	[]	7	5	3	6
cDNA FLJ57283, highly similar to Actin, cytoplasmic 2 OS=Homo sapiens PE=2 SV=1		40 kDa	0.24	1.2	[]	48	42	44	50
Ribosomal protein L18, isoform CRA_c OS=Homo sapiens GN=RPL18 PE=4 SV=1	RPL18	22 kDa	0.4	0.6	[]	9	5	3	5
Family with sequence similarity 62 (C2 domain containing), member A, isoform CRA_a OS=Homo sapiens GN=FAM62A P	FAM62A	124 kDa	0.0042	0.4	Fibro-BKO low, Fibro-Naive high	8	8	3	3
Reticulon OS=Homo sapiens GN=RTN3 PE=4 SV=1	RTN3	26 kDa	0.0096	0.5	Fibro-BKO low, Fibro-Naive high	6	7	3	3
cDNA FLJ75085, highly similar to Homo sapiens glutaminyl-tRNA synthetase (QARS), mRNA OS=Homo sapiens PE=2 SV=1		88 kDa	0.053	0.3	[]	6	9	2	2
Inactive tyrosine-protein kinase transmembrane receptor ROR1 OS=Homo sapiens GN=ROR1 PE=1 SV=1	ROR1	98 kDa	0.17	3.2	[]	0	5	6	8
Coatomer protein complex, subunit epsilon, isoform CRA_g OS=Homo sapiens GN=COPE PE=1 SV=1	COPE	37 kDa	0.057	0	[]	14	9	0	0
High mobility group AT-hook 1, isoform CRA_b OS=Homo sapiens GN=HMGA1 PE=4 SV=1	HMGA1	34 kDa	0.0011	0	Fibro-BKO low, Fibro-Naive high	12	12	0	0
RAB11A, member RAS oncogene family, isoform CRA_a OS=Homo sapiens GN=RAB11A PE=4 SV=1	RAB11A	24 kDa	0.52	0.8	[]	7	5	4	5
Prominin 2, isoform CRA_a OS=Homo sapiens GN=PROM2 PE=4 SV=1	PROM2	92 kDa	0.81	1.1	[]	5	6	8	3
cDNA, FLJ92896, highly similar to Homo sapiens proteasome (prosome, macropain) 26S subunit, non-ATPase, 1 (PSMD1), mRNA OS=Homo s		106 kDa	0.1	1.3	[]	4	5	5	5
Putative uncharacterized protein RAB10 (Fragment) OS=Homo sapiens GN=RAB10 PE=4 SV=1	RAB10	18 kDa	0.1	0.7	[]	8	7	4	5
Aconitate hydratase OS=Homo sapiens GN=HEL60 PE=2 SV=1	HEL60	98 kDa	0.11	1.6	[]	4	4	6	5
Actin related protein 2/3 complex, subunit 1B, 41kDa OS=Homo sapiens GN=ARPC1B PE=2 SV=1	ARPC1B	41 kDa	0.24	0.5	[]	9	5	3	3
Epoxide hydrolase OS=Homo sapiens GN=EPHX1 PE=2 SV=1	EPHX1	53 kDa	0.025	0.3	Fibro-BKO low, Fibro-Naive high	6	8	2	2
Actinin, alpha 1, isoform CRA_a OS=Homo sapiens GN=ACTN1 PE=4 SV=1	ACTN1	103 kDa	0.61	0.9	[]	20	29	15	23
Actin related protein 2/3 complex, subunit 2, 34kDa, isoform CRA_a OS=Homo sapiens GN=ARPC2 PE=2 SV=1	ARPC2	34 kDa	0.16	0.3	[]	5	9	3	1
Actin-like protein (Fragment) OS=Homo sapiens GN=ACT PE=4 SV=1	ACT	11 kDa	0.69	0.9	[]	15	12	9	13
Zinc finger, UBR1 type 1, isoform CRA_c OS=Homo sapiens GN=ZUBR1 PE=4 SV=1	ZUBR1	290 kDa	0.09	4.7	[]	0	5	11	9
Collagen, type XVIII, alpha 1, isoform CRA_d OS=Homo sapiens GN=COL18A1 PE=4 SV=1	COL18A1	136 kDa	0.29	3.1	[]	0	4	5	11
Alanyl (Membrane) aminopeptidase (Aminopeptidase N, aminopeptidase M, microsomal aminopeptidase, CD13, p150),	ANPEP	110 kDa	0.017	INF	Fibro-BKO high, Fibro-Naive low	0	0	20	28
cDNA FLJ56405, highly similar to Epidermal growth factor receptor kinase substrate 8 OS=Homo sapiens PE=2 SV=1		77 kDa	0.89	0.9	[]	3	8	4	5
CD59 glycoprotein OS=Homo sapiens GN=CD59 PE=1 SV=1	CD59	12 kDa	0.34	0.7	[]	7	5	4	4
Proteasome 26S ATPase subunit 1 variant (Fragment) OS=Homo sapiens PE=1 SV=1		49 kDa	0.34	0.9	[]	6	6	5	5
Vacuolar protein sorting 28 (Yeast), isoform CRA_a OS=Homo sapiens GN=VPS28 PE=2 SV=1	VPS28	25 kDa	0.037	2.1	Fibro-BKO high, Fibro-Naive low	4	4	8	7
cDNA FLJ56016, highly similar to C-1-tetrahydrofolate synthase, cytoplasmic OS=Homo sapiens PE=2 SV=1		110 kDa	0.19	0.7	[]	5	6	4	3
Protein disulfide-isomerase OS=Homo sapiens PE=2 SV=1		54 kDa	0.061	0.3	[]	9	7	2	3
Testicular tissue protein Li 149 OS=Homo sapiens PE=2 SV=1		46 kDa	0.64	0.7	[]	8	3	2	5
Pre-mRNA-processing-splicing factor 8 (Fragment) OS=Homo sapiens GN=PRPF8 PE=1 SV=8	PRPF8	120 kDa	0.2	0.5	[]	4	7	2	3
cDNA FLJ76962, highly similar to Homo sapiens nucleolar protein 5A (56kDa with KKE/D repeat) (NOL5A), mRNA OS=Homo sapiens PE=2 SV		66 kDa	0.0013	0.3	Fibro-BKO low, Fibro-Naive high	8	9	2	2
Nucleoside diphosphate kinase OS=Homo sapiens GN=NME1-NME2 PE=1 SV=1	NME1-NME2	33 kDa	0.081	0.5	[]	6	5	2	3

Transforming growth factor beta-induced 68kDa isoform 1 (Fragment) OS=Homo sapiens GN=TGFBI PE=2 SV=1	TGFBI	75 kDa	0.19	2.5	[]	2	3	7	4
cDNA FLJ35376 fis, clone SKMUS2004044, highly similar to Homo sapiens ribosomal protein L3 (RPL3), transcript variant 2, mRNA OS=Homo		40 kDa	0.38	0.5	[]	9	4	2	4
cDNA FLJ11291 fis, clone PLACE1009659, highly similar to Nck-associated protein 1 OS=Homo sapiens PE=2 SV=1		60 kDa	0.072	4	[]	1	3	8	6
Histone H2A OS=Homo sapiens GN=H2AFJ PE=3 SV=1	H2AFJ	14 kDa	0.073	0.2	[]	52	33	6	6
sp|K22E_HUMAN|		65 kDa	0.73	0.7	[]	9	7	1	10
Prelamin-A/C OS=Homo sapiens GN=LMNA PE=1 SV=1	LMNA	56 kDa	0.061	0.1	[]	11	8	2	0
EH-domain containing 2, isoform CRA_a OS=Homo sapiens GN=EHD2 PE=3 SV=1	EHD2	61 kDa	0.036	11	Fibro-BKO high, Fibro-Naive low	0	2	10	8
Stomatin, isoform CRA_a OS=Homo sapiens GN=STOM PE=4 SV=1	STOM	32 kDa	0.014	23	Fibro-BKO high, Fibro-Naive low	1	0	9	12
Proteasome activator complex subunit 2 OS=Homo sapiens GN=PSME2 PE=1 SV=1	PSME2	29 kDa	0.013	0.06	Fibro-BKO low, Fibro-Naive high	10	9	1	0
Gamma filamin variant (Fragment) OS=Homo sapiens PE=2 SV=1		143 kDa	0.0015	27	Fibro-BKO high, Fibro-Naive low	1	0	12	13
Syndecan binding protein (Syntenin), isoform CRA_c OS=Homo sapiens GN=SDCBP PE=4 SV=1	SDCBP	32 kDa	0.0062	1.3	Fibro-BKO high, Fibro-Naive low	86	90	100	103
Histone H2B OS=Homo sapiens GN=HIST1H2BK PE=3 SV=1	HIST1H2BK	14 kDa	0.14	0.4	[]	76	47	15	24
Testicular tissue protein Li 153 OS=Homo sapiens PE=2 SV=1		18 kDa	0.31	1.2	[]	4	6	5	6
Calpain 5, isoform CRA_c OS=Homo sapiens GN=CAPN5 PE=2 SV=1	CAPN5	73 kDa	0.84	0.9	[]	3	7	3	5
ARHG protein (Fragment) OS=Homo sapiens GN=ARHG PE=2 SV=1	ARHG	21 kDa	0.29	0.8	[]	6	5	4	4
Proteasome subunit alpha type OS=Homo sapiens PE=2 SV=1		26 kDa	0.29	1.3	[]	4	4	5	4
cDNA FLJ55002, highly similar to Alpha-centractin OS=Homo sapiens PE=2 SV=1		38 kDa	0.096	0.3	[]	10	7	3	2
Proteasome subunit alpha type (Fragment) OS=Homo sapiens GN=PSMA4 PE=1 SV=1	PSMA4	26 kDa	0.04	1.9	Fibro-BKO high, Fibro-Naive low	4	3	6	6
Isocitrate dehydrogenase [NADP] OS=Homo sapiens PE=2 SV=1		47 kDa	0.046	0.4	Fibro-BKO low, Fibro-Naive high	8	8	2	4
Phosphoglycerate mutase (Fragment) OS=Homo sapiens PE=2 SV=1		29 kDa	0.047	0.2	Fibro-BKO low, Fibro-Naive high	9	7	2	1
Pre-mRNA-processing factor 19 (Fragment) OS=Homo sapiens GN=PRPF19 PE=1 SV=1	PRPF19	28 kDa	0.016	0.3	Fibro-BKO low, Fibro-Naive high	9	6	2	2
60S ribosomal protein L7a OS=Homo sapiens GN=RPL7A PE=1 SV=1	RPL7A	17 kDa	0.14	0.3	[]	12	7	2	3
CAD protein OS=Homo sapiens GN=CAD PE=1 SV=1	CAD	236 kDa	0.025	5	Fibro-BKO high, Fibro-Naive low	1	2	7	6
Ubiquitinyl hydrolase 1 OS=Homo sapiens PE=2 SV=1		96 kDa	0.24	0.5	[]	7	8	5	1
Small nuclear ribonucleoprotein Sm D1 OS=Homo sapiens GN=SNRPD1 PE=1 SV=1	SNRPD1	8 kDa	0.044	0.3	Fibro-BKO low, Fibro-Naive high	8	8	3	1
Sorcin OS=Homo sapiens GN=SRI PE=1 SV=1	SRI	18 kDa	0.017	0.2	Fibro-BKO low, Fibro-Naive high	7	6	1	1
Villin 1 variant (Fragment) OS=Homo sapiens PE=2 SV=1		93 kDa	0.039	0.3	Fibro-BKO low, Fibro-Naive high	7	8	1	3
DNA-directed RNA polymerase subunit OS=Homo sapiens GN=POLR2A PE=1 SV=1	POLR2A	218 kDa	0.027	0.06	Fibro-BKO low, Fibro-Naive high	7	10	0	1
Epididymis secretory sperm binding protein Li 129m OS=Homo sapiens GN=HEL-S-129m PE=2 SV=1	HEL-S-129m	29 kDa	0.011	0.06	Fibro-BKO low, Fibro-Naive high	8	10	0	1
Eukaryotic translation initiation factor 3 subunit L OS=Homo sapiens GN=EIF3L PE=1 SV=1	EIF3L	71 kDa	0.036	0.6	Fibro-BKO low, Fibro-Naive high	6	7	3	4
cDNA FLJ61359, highly similar to Adapter-relatedprotein complex 2 alpha- 2 subunit OS=Homo sapiens PE=2 SV=1		103 kDa	0.23	0.6	[]	7	11	4	6
CEACAM1 protein (Fragment) OS=Homo sapiens GN=CEACAM1 PE=2 SV=1	CEACAM1	53 kDa	0.99	1	[]	4	5	3	5
Testis secretory sperm-binding protein Li 197a OS=Homo sapiens PE=2 SV=1		49 kDa	0.64	1.1	[]	4	3	3	4
cDNA FLJ31086 fis, clone IMR321000044, highly similar to Tyrosine-protein kinase-like 7 OS=Homo sapiens PE=2 SV=1		46 kDa	0.62	1.1	[]	5	4	5	4
Tyrosine-protein kinase OS=Homo sapiens PE=2 SV=1		61 kDa	0.49	1.4	[]	3	6	4	7
IQ motif containing GTPase activating protein 2, isoform CRA_b OS=Homo sapiens GN=IQGAP2 PE=4 SV=1	IQGAP2	181 kDa	0.021	0.4	Fibro-BKO low, Fibro-Naive high	6	7	2	3
Proteasome subunit alpha type OS=Homo sapiens PE=2 SV=1		28 kDa	1	1	[]	3	5	4	3
Histone H2B OS=Homo sapiens GN=ABCF2 PE=3 SV=1	ABCF2	7 kDa	0.12	0.5	[]	7	6	2	4
cDNA FLJ77464, highly similar to Homo sapiens protocadherin 1 (cadherin-like 1) (PCDH1), transcript variant 1, mRNA OS=Homo sapiens PE=		115 kDa	0.042	2.9	Fibro-BKO high, Fibro-Naive low	3	2	7	6
Claudin OS=Homo sapiens GN=CLDN3 PE=2 SV=1	CLDN3	23 kDa	0.088	0.5	[]	12	11	4	7
cDNA FLJ57825, highly similar to DNA-dependent protein kinase catalytic subunit (EC 2.7.11.1) OS=Homo sapiens PE=2 SV=1		128 kDa	0.45	0.5	[]	2	7	2	2
Tubulin tyrosine ligase-like family, member 12, isoform CRA_a OS=Homo sapiens GN=TTLL12 PE=4 SV=1	TTLL12	74 kDa	0.014	0.1	Fibro-BKO low, Fibro-Naive high	8	7	1	1
Epididymis secretory protein Li 55 OS=Homo sapiens GN=HEL-S-55 PE=2 SV=1	HEL-S-55	17 kDa	0.05	0.06	[]	10	7	0	1
NID2 protein OS=Homo sapiens GN=NID2 PE=2 SV=1	NID2	107 kDa	< 0.00010	INF	Fibro-BKO high, Fibro-Naive low	0	0	22	24
Epididymis secretory protein Li 102 OS=Homo sapiens GN=HEL-S-102 PE=2 SV=1	HEL-S-102	23 kDa	0.52	1.1	[]	3	4	3	4
Receptor-type tyrosine-protein phosphatase F (Fragment) OS=Homo sapiens GN=PTPRF PE=1 SV=1	PTPRF	146 kDa	0.6	1.1	[]	3	5	4	4
Epididymis secretory protein Li 34 OS=Homo sapiens GN=PEBP1 PE=2 SV=1	PEBP1	21 kDa	0.39	0.7	[]	5	6	5	2
Tyrosine 3-monooxygenase/tryptophan 5-monooxygenase activation protein, eta polypeptide, isoform CRA_b OS=Hom	YWHAH	28 kDa	0.29	0.8	[]	5	5	4	3
ATP-binding cassette sub-family E member 1 OS=Homo sapiens GN=ABCE1 PE=1 SV=1	ABCE1	47 kDa	0.37	0.6	[]	3	6	2	3
Carbonic anhydrase 12 OS=Homo sapiens GN=CA12 PE=1 SV=1	CA12	32 kDa	0.011	2.9	Fibro-BKO high, Fibro-Naive low	2	3	6	7
Tubulin beta chain OS=Homo sapiens GN=TUBB PE=2 SV=1	TUBB	50 kDa	0.46	0.9	[]	83	70	52	67
Midkine OS=Homo sapiens GN=MDK PE=1 SV=1	MDK	17 kDa	0.21	0.6	[]	6	5	2	4
cDNA FLJ50285, highly similar to Arginyl-tRNA synthetase (EC 6.1.1.19) OS=Homo sapiens PE=2 SV=1		52 kDa	0.062	0.2	[]	8	6	2	1
Armadillo repeat gene deletes in velocardiofacial syndrome OS=Homo sapiens GN=ARVCF PE=2 SV=1	ARVCF	105 kDa	0.25	2.1	[]	1	5	6	5
RAB14, member RAS oncogene family, isoform CRA_a OS=Homo sapiens GN=RAB14 PE=4 SV=1	RAB14	24 kDa	0.27	0.5	[]	7	9	1	6
cDNA FLJ16785 fis, clone NT2RI2015342, highly similar to Solute carrier family 2, facilitated glucose transporter member 1 OS=Homo sapien		46 kDa	0.074	3.2	[]	1	3	6	5
cDNA FLJ52352, highly similar to DnaJ homolog subfamily A member 1 OS=Homo sapiens PE=2 SV=1		28 kDa	0.33	0.5	[]	6	6	1	5
Testicular secretory protein Li 9 OS=Homo sapiens PE=2 SV=1		22 kDa	0.014	0.1	Fibro-BKO low, Fibro-Naive high	8	7	1	1
HECT, UBA and WWE domain containing 1, isoform CRA_c OS=Homo sapiens GN=HUWE1 PE=4 SV=1	HUWE1	481 kDa	0.062	0.2	[]	8	6	2	1

HCG27198, isoform CRA_c OS=Homo sapiens GN=hCG_27198 PE=3 SV=1	hCG_27198	231 kDa	0.16	0.3	[]	5	9	3	1
Epididymis secretory protein Li 106 OS=Homo sapiens GN=HEL-S-106 PE=2 SV=1	HEL-S-106	56 kDa	0.16	0.3	[]	7	5	0	3
Integrin alpha-6 (Fragment) OS=Homo sapiens GN=ITGA6 PE=1 SV=1	ITGA6	30 kDa	0.085	0.2	[]	6	5	2	0
Complement decay-accelerating factor OS=Homo sapiens GN=CD55 PE=4 SV=1	CD55	41 kDa	0.079	0.8	[]	4	4	3	3
Inter-alpha (Globulin) inhibitor H2 OS=Homo sapiens GN=ITIH2 PE=2 SV=1	ITIH2	106 kDa	0.066	3.6	[]	2	2	5	8
Ribosomal protein S5, isoform CRA_a OS=Homo sapiens GN=RPS5 PE=3 SV=1	RPS5	23 kDa	0.28	0.6	[]	6	5	4	2
Protein arginine N-methyltransferase 5 OS=Homo sapiens PE=2 SV=1		73 kDa	0.086	0.3	[]	7	5	2	1
AP-1 complex subunit beta-1 (Fragment) OS=Homo sapiens GN=AP1B1 PE=1 SV=1	AP1B1	65 kDa	0.085	0.4	[]	19	14	6	6
S-formylglutathione hydrolase OS=Homo sapiens GN=ESD PE=2 SV=1	ESD	31 kDa	0.088	0.2	[]	8	5	1	1
cDNA, FLJ96587, highly similar to Homo sapiens SUMO-1 activating enzyme subunit 2 (UBA2), mRNA OS=Homo sapiens PE=2 SV=1		71 kDa	0.18	0.3	[]	5	10	3	1
Putative uncharacterized protein XRCC5 (Fragment) OS=Homo sapiens GN=XRCC5 PE=4 SV=1	XRCC5	18 kDa	0.08	0.3	[]	5	8	2	1
cDNA, FLJ96792, highly similar to Homo sapiens calmodulin 2 (phosphorylase kinase, delta) (CALM2), mRNA OS=Homo sapiens PE=2 SV=1		17 kDa	0.082	0.2	[]	8	7	0	3
Topoisomerase (DNA) I OS=Homo sapiens GN=TOP1 PE=2 SV=1	TOP1	91 kDa	0.019	0.1	Fibro-BKO low, Fibro-Naive high	12	10	1	1
LRP1 protein OS=Homo sapiens GN=LRP1 PE=1 SV=1	LRP1	48 kDa	0.0014	INF	Fibro-BKO high, Fibro-Naive low	0	0	7	7
Capping protein (Actin filament) muscle Z-line, alpha 1, isoform CRA_a OS=Homo sapiens GN=CAPZA1 PE=3 SV=1	CAPZA1	33 kDa	0.38	1.1	[]	4	5	4	5
cDNA FLJ76282, highly similar to Homo sapiens proteasome (prosome, macropain) 26S subunit, non-ATPase, 5 (PSMD5), mRNA OS=Homo s		56 kDa	0.63	0.9	[]	5	4	4	3
Alpha-1,4 glucan phosphorylase (Fragment) OS=Homo sapiens PE=2 SV=1		99 kDa	0.054	0.4	[]	64	52	22	23
WASF2 protein (Fragment) OS=Homo sapiens GN=WASF2 PE=2 SV=1	WASF2	21 kDa	0.18	0.9	[]	5	5	4	4
Tubulin alpha chain (Fragment) OS=Homo sapiens GN=TUBA4A PE=1 SV=1	TUBA4A	20 kDa	0.44	1.2	[]	11	9	9	12
cDNA FLJ52118, highly similar to 14-3-3 protein theta OS=Homo sapiens PE=2 SV=1		24 kDa	0.083	0.4	[]	12	9	4	4
cDNA FLJ60461, highly similar to Peroxiredoxin-2 (EC 1.11.1.15) OS=Homo sapiens PE=2 SV=1		20 kDa	0.14	0.7	[]	7	7	5	4
SFPQ protein (Fragment) OS=Homo sapiens GN=SFPQ PE=2 SV=1	SFPQ	55 kDa	0.089	0.4	[]	7	5	2	2
Spectrin, alpha, non-erythrocytic 1 (Alpha-fodrin), isoform CRA_g OS=Homo sapiens GN=SPTAN1 PE=4 SV=1	SPTAN1	282 kDa	0.28	0.5	[]	3	7	2	2
Cleavage and polyadenylation specific factor 1, 160kDa, isoform CRA_a OS=Homo sapiens GN=CPSF1 PE=4 SV=1	CPSF1	152 kDa	0.088	0.6	[]	5	5	3	2
E3 ubiquitin-protein ligase UBR4 (Fragment) OS=Homo sapiens GN=UBR4 PE=1 SV=1	UBR4	225 kDa	0.025	2.8	Fibro-BKO high, Fibro-Naive low	3	3	8	7
Epididymis secretory sperm binding protein Li 95n OS=Homo sapiens GN=HEL-S-95n PE=2 SV=1	HEL-S-95n	38 kDa	0.14	0.4	[]	7	5	1	3
40S ribosomal protein S16 OS=Homo sapiens GN=RPS16 PE=1 SV=1	RPS16	14 kDa	0.037	0.3	Fibro-BKO low, Fibro-Naive high	5	5	2	1
cDNA FLJ78217 OS=Homo sapiens PE=2 SV=1		40 kDa	0.55	1.5	[]	1	5	3	5
Profilin (Fragment) OS=Homo sapiens GN=PFN1 PE=1 SV=1	PFN1	18 kDa	0.43	0.8	[]	7	7	6	4
60S ribosomal protein L7a (Fragment) OS=Homo sapiens GN=RPL7A PE=1 SV=1	RPL7A	22 kDa	0.061	0.3	[]	9	7	2	3
Proteasome subunit beta type-3 (Fragment) OS=Homo sapiens GN=PSMB3 PE=1 SV=1	PSMB3	16 kDa	0.42	1.6	[]	1	4	3	4
Brain-specific angiogenesis inhibitor 1-associated protein 2 OS=Homo sapiens GN=BAIAP2 PE=1 SV=1	BAIAP2	61 kDa	0.34	0.6	[]	4	6	1	4
Albumin (Fragment) OS=Homo sapiens PE=2 SV=1		67 kDa	0.021	2.5	Fibro-BKO high, Fibro-Naive low	8	7	15	19
Proteasome endopeptidase complex OS=Homo sapiens GN=HEL-S-275 PE=2 SV=1	HEL-S-275	30 kDa	0.2	1.6	[]	4	5	5	8
cDNA FLJ52684, highly similar to Dynein heavy chain, cytosolic OS=Homo sapiens PE=2 SV=1		28 kDa	0.021	2.3	Fibro-BKO high, Fibro-Naive low	2	3	5	5
cDNA PSEC0048 fis, clone NT2RP2000028, highly similar to Serine protease 23 OS=Homo sapiens PE=2 SV=1		43 kDa	0.0037	2.8	Fibro-BKO high, Fibro-Naive low	2	2	5	5
Plectin (Fragment) OS=Homo sapiens GN=PLEC PE=1 SV=1	PLEC	81 kDa	0.38	0.8	[]	4	6	3	4
Vacuolar protein sorting 4B (Yeast), isoform CRA_a OS=Homo sapiens GN=VPS4B PE=3 SV=1	VPS4B	49 kDa	0.093	2.1	[]	2	3	5	4
cDNA FLJ55534, highly similar to 4-trimethylaminobutyraldehyde dehydrogenase (EC 1.2.1.47) OS=Homo sapiens PE=2 SV=1		53 kDa	0.075	0.5	[]	5	4	2	2
RPL14 protein (Fragment) OS=Homo sapiens GN=RPL14 PE=2 SV=1	RPL14	15 kDa	0.13	0.3	[]	6	4	2	1
Guanine nucleotide-binding protein G, alpha subunit variant (Fragment) OS=Homo sapiens PE=2 SV=1		42 kDa	0.48	1.6	[]	2	6	7	4
40S ribosomal protein S13 OS=Homo sapiens GN=RPS13 PE=1 SV=1	RPS13	17 kDa	0.45	0.7	[]	3	2	2	1
Histone H2A OS=Homo sapiens GN=H2AFV PE=1 SV=1	H2AFV	13 kDa	0.0018	0.2	Fibro-BKO low, Fibro-Naive high	6	6	1	1
Interferon induced transmembrane protein 1 (9-27), isoform CRA_a OS=Homo sapiens GN=IFITM1 PE=4 SV=1	IFITM1	14 kDa	0.045	2	Fibro-BKO high, Fibro-Naive low	4	4	6	8
Calpain 1, (Mu/I) large subunit, isoform CRA_a OS=Homo sapiens GN=CAPN1 PE=3 SV=1	CAPN1	82 kDa	0.6	0.7	[]	4	4	4	1
Dipeptidase OS=Homo sapiens PE=2 SV=1		46 kDa	0.31	1.3	[]	3	3	4	3
Septin 9, isoform CRA_a OS=Homo sapiens GN=SEPT9 PE=3 SV=1	Sep-09	64 kDa	0.32	0.5	[]	4	7	1	4
Proteasome subunit beta type OS=Homo sapiens GN=PSMB6 PE=2 SV=1	PSMB6	25 kDa	0.62	1.8	[]	2	3	1	5
HCG2044799 OS=Homo sapiens GN=HNRNPUL2-BSCL2 PE=4 SV=1	HNRNPUL2-BSCL2	85 kDa	0.017	0.2	Fibro-BKO low, Fibro-Naive high	6	8	1	1
cDNA FLJ57882, highly similar to Pre-mRNA-processing-splicing factor 8 OS=Homo sapiens PE=2 SV=1		123 kDa	0.21	0.4	[]	3	6	2	1
Myoferlin (Fragment) OS=Homo sapiens GN=MYOF PE=1 SV=1	MYOF	135 kDa	0.074	7.8	[]	1	1	5	9
HCG30204, isoform CRA_a OS=Homo sapiens GN=hCG_30204 PE=4 SV=1	hCG_30204	257 kDa	0.4	2.1	[]	0	5	5	4
Spectrin beta chain, non-erythrocytic 1 OS=Homo sapiens GN=SPTBN1 PE=1 SV=1	SPTBN1	275 kDa	0.24	0.2	[]	3	8	2	0
Epididymis luminal protein 113 OS=Homo sapiens GN=HEL113 PE=2 SV=1	HEL113	54 kDa	0.011	INF	Fibro-BKO high, Fibro-Naive low	0	0	8	7
RAB5C, member RAS oncogene family, isoform CRA_a OS=Homo sapiens GN=RAB5C PE=4 SV=1	RAB5C	23 kDa	0.017	0.7	Fibro-BKO low, Fibro-Naive high	5	5	3	3
Vesicle amine transport protein 1 homolog (T californica), isoform CRA_a OS=Homo sapiens GN=VAT1 PE=4 SV=1	VAT1	42 kDa	0.58	0.8	[]	4	4	2	4
V-ral simian leukemia viral oncogene homolog B (Ras related GTP binding protein), isoform CRA_a OS=Homo sapiens GN	RALB	23 kDa	0.79	1.1	[]	5	4	3	6
Nucleophosmin isoform 2 (Fragment) OS=Homo sapiens GN=NPM1 PE=2 SV=1	NPM1	33 kDa	0.97	1	[]	4	4	4	3
Dihydropyrimidinase-related protein 2 OS=Homo sapiens GN=DPYSL2 PE=1 SV=1	DPYSL2	74 kDa	0.0099	0.4	Fibro-BKO low, Fibro-Naive high	5	6	2	2

Putative uncharacterized protein DKFZp547A0616 (Fragment) OS=Homo sapiens GN=DKFZp547A0616 PE=2 SV=1	DKFZp547A0616	20 kDa	0.11	3.1	[]	2	2	4	7
Arrestin domain containing 1 variant (Fragment) OS=Homo sapiens PE=2 SV=1		35 kDa	0.091	1.8	[]	4	7	8	9
cDNA FLJ56126, highly similar to Programmed cell death 6-interacting protein OS=Homo sapiens PE=2 SV=1		72 kDa	0.32	1.1	[]	41	51	47	45
ATP-binding cassette, sub-family C, member 3 isoform MRP3 variant (Fragment) OS=Homo sapiens PE=2 SV=1		170 kDa	0.55	1.6	[]	1	5	5	3
DnaJ (Hsp40) homolog, subfamily A, member 2, isoform CRA_a OS=Homo sapiens GN=DNAJA2 PE=3 SV=1	DNAJA2	46 kDa	0.1	0.5	[]	3	4	1	2
cDNA FLJ78448, highly similar to Homo sapiens argininosuccinate synthetase (ASS), transcript variant 1, mRNA OS=Homo sapiens PE=2 SV=		47 kDa	0.04	0.5	Fibro-BKO low, Fibro-Naive high	5	6	2	3
60S ribosomal protein L10 (Fragment) OS=Homo sapiens GN=RPL10 PE=1 SV=7	RPL10	23 kDa	0.0034	0.4	Fibro-BKO low, Fibro-Naive high	3	3	1	1
Phospholipid scramblase OS=Homo sapiens GN=hCG_1987383 PE=3 SV=1	hCG_1987383	38 kDa	0.25	1.4	[]	4	3	5	4
Protein S100 OS=Homo sapiens PE=2 SV=1		10 kDa	0.0099	0.4	Fibro-BKO low, Fibro-Naive high	5	6	2	2
cDNA FLJ58882, highly similar to 26S proteasome non-ATPase regulatory subunit 11 OS=Homo sapiens PE=2 SV=1		35 kDa	0.043	0.1	Fibro-BKO low, Fibro-Naive high	5	4	0	1
Nuclear cap binding protein subunit 1, 80kDa, isoform CRA_a OS=Homo sapiens GN=NCBP1 PE=4 SV=1	NCBP1	92 kDa	0.03	0.09	Fibro-BKO low, Fibro-Naive high	5	7	0	1
SARS protein OS=Homo sapiens GN=SARS PE=2 SV=1	SARS	58 kDa	0.068	0.2	[]	5	7	0	2
THO complex subunit 2 OS=Homo sapiens GN=THOC2 PE=1 SV=1	THOC2	170 kDa	0.05	0	Fibro-BKO low, Fibro-Naive high	6	4	0	0
Putative uncharacterized protein DKFZp686C11235 OS=Homo sapiens GN=DKFZp686C11235 PE=2 SV=1	DKFZp686C11235	52 kDa	0.2	INF	[]	0	0	4	14
Thyroglobulin OS=Homo sapiens GN=TG PE=2 SV=1	TG	305 kDa	0.11	INF	[]	0	0	8	4
High mobility group AT-hook 1 OS=Homo sapiens GN=HMGA1 PE=2 SV=1	HMGA1	11 kDa	0.0019	0	Fibro-BKO low, Fibro-Naive high	6	7	0	0
Calcium/calmodulin-dependent protein kinase (CaM kinase) II delta, isoform CRA_a OS=Homo sapiens GN=CAMK2D PE=	CAMK2D	56 kDa	0.89	0.9	[]	4	3	2	4
Uridine diphospho-glucose dehydrogenase (Fragment) OS=Homo sapiens GN=GDH PE=4 SV=1	GDH	18 kDa	0.093	2.1	[]	2	3	5	4
Secretory carrier-associated membrane protein (Fragment) OS=Homo sapiens GN=SCAMP3 PE=2 SV=1	SCAMP3	38 kDa	0.14	0.4	[]	6	4	2	2
Polyadenylate-binding protein OS=Homo sapiens GN=PABPC1 PE=3 SV=1	PABPC1	71 kDa	0.47	1.8	[]	3	2	2	6
Amino acid transporter (Fragment) OS=Homo sapiens PE=2 SV=1		57 kDa	0.24	0.9	[]	27	34	21	25
Ras homolog gene family, member A, isoform CRA_a OS=Homo sapiens GN=RHOA PE=4 SV=1	RHOA	22 kDa	0.51	0.8	[]	12	7	6	7
Guanine nucleotide binding protein (G protein), alpha 13, isoform CRA_a OS=Homo sapiens GN=GNA13 PE=4 SV=1	GNA13	44 kDa	0.87	1.1	[]	1	5	2	4
cDNA FLJ76863, highly similar to Homo sapiens stress-induced-phosphoprotein 1 (Hsp70/Hsp90-organizing protein) (STIP1), mRNA OS=Hom		63 kDa	0.1	0.2	[]	4	5	0	2
cDNA, FLJ96812, highly similar to Homo sapiens threonyl-tRNA synthetase (TARS), mRNA OS=Homo sapiens PE=1 SV=1		82 kDa	0.0034	0.4	Fibro-BKO low, Fibro-Naive high	3	3	1	1
Serpin peptidase inhibitor, clade C (Antithrombin), member 1, isoform CRA_a OS=Homo sapiens GN=SERPINC1 PE=3 SV=	SERPINC1	53 kDa	0.035	3	Fibro-BKO high, Fibro-Naive low	2	1	4	4
Integrin beta OS=Homo sapiens GN=ITGB3 PE=2 SV=1	ITGB3	87 kDa	0.073	5.7	[]	1	1	6	4
Tetraspanin OS=Homo sapiens GN=CD63 PE=3 SV=1	CD63	26 kDa	0.5	1.5	[]	3	2	2	5
60S acidic ribosomal protein P0 OS=Homo sapiens GN=RPLP0 PE=3 SV=1	RPLP0	34 kDa	0.03	0.1	Fibro-BKO low, Fibro-Naive high	6	5	0	1
Eukaryotic translation initiation factor 3 subunit E OS=Homo sapiens GN=EIF3E PE=2 SV=1	EIF3E	52 kDa	0.2	0	[]	3	1	0	0
Pre-B-cell colony enhancing factor 1, isoform CRA_a OS=Homo sapiens GN=PBEF1 PE=4 SV=1	PBEF1	56 kDa	0.026	0.1	Fibro-BKO low, Fibro-Naive high	4	5	0	1
Testicular tissue protein Li 82 OS=Homo sapiens PE=2 SV=1		77 kDa	0.0059	0	Fibro-BKO low, Fibro-Naive high	4	5	0	0
Aspartate aminotransferase OS=Homo sapiens PE=2 SV=1		46 kDa	0.017	0	Fibro-BKO low, Fibro-Naive high	5	7	0	0
DNA-directed RNA polymerase subunit beta OS=Homo sapiens PE=2 SV=1		133 kDa	0.063	0	[]	8	5	0	0
cDNA FLJ46846 fis, clone UTERU3004635, moderately similar to Neuroblast differentiation associated protein AHNAK (Fragment) OS=Homo		181 kDa	0.52	1.1	[]	3	4	3	4
Disks large homolog 1 OS=Homo sapiens GN=DLG1 PE=1 SV=1	DLG1	100 kDa	0.7	1.1	[]	2	3	3	2
Abl interactor 1 OS=Homo sapiens GN=ABI1 PE=1 SV=1	ABI1	52 kDa	0.23	1.5	[]	2	4	4	4
Amino acid transporter OS=Homo sapiens GN=SLC1A5 PE=2 SV=1	SLC1A5	57 kDa	0.1	0.9	[]	27	31	21	24
Proteasome subunit beta type (Fragment) OS=Homo sapiens GN=PSMB8 PE=2 SV=1	PSMB8	30 kDa	0.77	1.1	[]	3	3	4	2
Transportin 3, isoform CRA_b OS=Homo sapiens GN=TNPO3 PE=4 SV=1	TNPO3	104 kDa	0.48	0.7	[]	2	5	2	2
cDNA FLJ78244, highly similar to Homo sapiens eukaryotic translation initiation factor 4A, isoform 1 (EIF4A1), mRNA OS=Homo sapiens PE=		46 kDa	0.11	1.9	[]	3	5	7	6
Dihydrolipoamide S-succinyltransferase (E2 component of 2-oxo-glutarate complex), isoform CRA_a OS=Homo sapiens (	DLST	49 kDa	0.18	0.6	[]	4	3	2	2
KLK10 protein (Fragment) OS=Homo sapiens GN=KLK10 PE=2 SV=1	KLK10	30 kDa	0.17	0.4	[]	3	5	2	1
ADP-ribosylation factor 6 OS=Homo sapiens PE=2 SV=1		20 kDa	0.86	1.1	[]	2	1	1	2
RAB21, member RAS oncogene family, isoform CRA_a OS=Homo sapiens GN=RAB21 PE=4 SV=1	RAB21	24 kDa	0.17	0.4	[]	3	5	2	1
Tropomyosin 3 isoform 1 (Fragment) OS=Homo sapiens GN=TPM3 PE=2 SV=1	TPM3	29 kDa	0.0099	0.4	Fibro-BKO low, Fibro-Naive high	5	6	2	2
ST3 beta-galactoside alpha-2,3-sialyltransferase 1, isoform CRA_a OS=Homo sapiens GN=ST3GAL1 PE=3 SV=1	ST3GAL1	39 kDa	0.011	1.7	Fibro-BKO high, Fibro-Naive low	2	2	3	3
Uridine diphospho-glucose dehydrogenase (Fragment) OS=Homo sapiens GN=GDH PE=4 SV=1	GDH	17 kDa	0.7	1.1	[]	2	3	3	2
Calcium binding protein 39, isoform CRA_a OS=Homo sapiens GN=CAB39 PE=4 SV=1	CAB39	40 kDa	0.68	0.7	[]	1	4	2	1
cDNA FLJ10153 fis, clone HEMBA1003417, highly similar to BAG family molecular chaperone regulator 2 OS=Homo sapiens PE=2 SV=1		24 kDa	0.0024	0.3	Fibro-BKO low, Fibro-Naive high	4	4	1	1
Annexin OS=Homo sapiens PE=2 SV=1		53 kDa	0.39	0.7	[]	3	2	1	2
Septin-2 OS=Homo sapiens GN=SEPT2 PE=1 SV=1	Sep-02	37 kDa	0.019	0.3	Fibro-BKO low, Fibro-Naive high	11	11	2	4
Eukaryotic translation initiation factor 3 subunit C OS=Homo sapiens GN=EIF3S8 PE=3 SV=1	EIF3S8	105 kDa	0.23	0.3	[]	2	5	1	1
Epididymis secretory sperm binding protein Li 8a OS=Homo sapiens GN=HEL-S-8a PE=2 SV=1	HEL-S-8a	31 kDa	0.023	0.3	Fibro-BKO low, Fibro-Naive high	5	6	1	2
Obg-like ATPase 1 OS=Homo sapiens GN=OLA1 PE=1 SV=1	OLA1	47 kDa	0.46	0.7	[]	4	3	3	1
Copine III, isoform CRA_a OS=Homo sapiens GN=CPNE3 PE=4 SV=1	CPNE3	60 kDa	0.0082	0.6	Fibro-BKO low, Fibro-Naive high	4	4	2	2
cDNA FLJ53963, highly similar to Leukocyte elastase inhibitor OS=Homo sapiens PE=2 SV=1		39 kDa	0.27	0.3	[]	2	6	1	1
cDNA FLJ61545, highly similar to Coatomer subunit beta OS=Homo sapiens PE=2 SV=1		31 kDa	0.055	0.3	[]	6	5	2	1
Ribosomal protein L5 OS=Homo sapiens GN=RPL5 PE=2 SV=1	RPL5	34 kDa	0.045	0.3	Fibro-BKO low, Fibro-Naive high	6	5	1	2

Scavenger receptor class B, member 2, isoform CRA_a OS=Homo sapiens GN=SCARB2 PE=3 SV=1	SCARB2	54 kDa	0.0033	0	Fibro-BKO low, Fibro-Naive high	5	6	0	0
Adenine phosphoribosyltransferase (Fragment) OS=Homo sapiens GN=APRT PE=1 SV=8	APRT	16 kDa	0.33	0.3	[]	7	3	0	3
cDNA FLJ58269, highly similar to Bifunctional aminoacyl-tRNA synthetase OS=Homo sapiens PE=2 SV=1		54 kDa	0.02	0	Fibro-BKO low, Fibro-Naive high	5	4	0	0
2'-5'-oligoadenylate synthetase 3, 100kDa, isoform CRA_a OS=Homo sapiens GN=OAS3 PE=4 SV=1	OAS3	121 kDa	0.15	0.1	[]	6	3	0	1
cDNA FLJ13913 fis, clone Y79AA1000231, highly similar to Nucleolar protein NOP5 OS=Homo sapiens PE=2 SV=1		52 kDa	0.015	0	Fibro-BKO low, Fibro-Naive high	6	5	0	0
Laminin, beta 2 (Laminin S), isoform CRA_a OS=Homo sapiens GN=LAMB2 PE=4 SV=1	LAMB2	196 kDa	0.0014	INF	Fibro-BKO high, Fibro-Naive low	0	0	5	6
Heat shock 60kDa protein 1 (Chaperonin), isoform CRA_a OS=Homo sapiens GN=HSPD1 PE=2 SV=1	HSPD1	61 kDa	0.081	0	[]	7	4	0	0
cDNA FLJ42779 fis, clone BRAWH3005300, highly similar to Exportin-1 OS=Homo sapiens PE=2 SV=1		82 kDa	0.05	0	Fibro-BKO low, Fibro-Naive high	6	4	0	0
Epididymis secretory sperm binding protein Li 71p OS=Homo sapiens GN=HEL-S-71p PE=2 SV=1	HEL-S-71p	77 kDa	0.42	INF	[]	0	0	12	0
RAP1B, member of RAS oncogene family, isoform CRA_a OS=Homo sapiens GN=RAP1B PE=4 SV=1	RAP1B	21 kDa	0.6	1.1	[]	9	9	9	8
cDNA FLJ36998 fis, clone BRACE2007295, highly similar to ALPHA-ADAPTIN A OS=Homo sapiens PE=2 SV=1		52 kDa	0.034	1.7	Fibro-BKO high, Fibro-Naive low	3	3	4	5
Serine/threonine-protein phosphatase OS=Homo sapiens GN=HEL-S-80p PE=2 SV=1	HEL-S-80p	37 kDa	0.12	0.4	[]	17	13	4	8
Signal transducer and activator of transcription OS=Homo sapiens GN=STAT1 PE=1 SV=1	STAT1	83 kDa	0.21	1.7	[]	2	4	5	4
Telomerase protein component 1 OS=Homo sapiens GN=TEP1 PE=1 SV=1	TEP1	278 kDa	0.41	0.6	[]	4	10	4	3
Family with sequence similarity 49, member B, isoform CRA_a OS=Homo sapiens GN=FAM49B PE=4 SV=1	FAM49B	37 kDa	0.0082	0.6	Fibro-BKO low, Fibro-Naive high	4	4	2	2
Ras-related protein Ral-A (Fragment) OS=Homo sapiens GN=RALA PE=1 SV=1	RALA	18 kDa	0.64	1.1	[]	4	3	3	4
Septin-11 OS=Homo sapiens GN=SEPT11 PE=1 SV=1	Sep-11	50 kDa	0.23	0.7	[]	4	4	3	2
Rho GDP-dissociation inhibitor 1 OS=Homo sapiens GN=ARHGDIA PE=1 SV=1	ARHGDIA	15 kDa	0.03	0.8	Fibro-BKO low, Fibro-Naive high	3	3	2	2
Aspartyl aminopeptidase, isoform CRA_b OS=Homo sapiens GN=DNPEP PE=3 SV=1	DNPEP	52 kDa	0.62	0.8	[]	3	3	3	1
Tetraspanin OS=Homo sapiens GN=CD151 PE=3 SV=1	CD151	28 kDa	0.25	2.3	[]	1	3	5	3
CSTB protein OS=Homo sapiens GN=CSTB PE=2 SV=1	CSTB	11 kDa	0.046	0.4	Fibro-BKO low, Fibro-Naive high	4	4	1	2
Testicular tissue protein Li 42 OS=Homo sapiens PE=2 SV=1		30 kDa	0.58	1.4	[]	3	1	2	3
Nucleosome assembly protein 1-like 1, isoform CRA_a OS=Homo sapiens GN=NAP1L1 PE=3 SV=1	NAP1L1	45 kDa	0.83	0.9	[]	3	3	3	2
Drug-sensitive protein 1 OS=Homo sapiens GN=YA61 PE=2 SV=1	YA61	15 kDa	0.83	0.9	[]	2	3	1	3
cDNA FLJ59343, highly similar to Exportin-2 OS=Homo sapiens PE=2 SV=1		69 kDa	0.12	0.3	[]	5	9	2	2
Protein scribble homolog OS=Homo sapiens GN=SCRIB PE=1 SV=1	SCRIB	175 kDa	0.33	1.4	[]	3	5	4	6
UTP--glucose-1-phosphate uridylyltransferase OS=Homo sapiens GN=UGP2 PE=1 SV=1	UGP2	57 kDa	0.76	0.8	[]	1	5	2	2
Eukaryotic translation initiation factor 3 subunit F OS=Homo sapiens GN=EIF3F PE=2 SV=1	EIF3F	39 kDa	0.98	1	[]	4	3	5	1
Fructose-bisphosphate aldolase OS=Homo sapiens GN=ALDOC PE=3 SV=1	ALDOC	39 kDa	0.92	0.9	[]	6	9	7	2
Platelet-activating factor acetylhydrolase, isoform Ib, gamma subunit 29kDa, isoform CRA_a OS=Homo sapiens GN=PAF	PAFAH1B3	26 kDa	0.029	0.1	Fibro-BKO low, Fibro-Naive high	4	5	1	0
FARSLA protein OS=Homo sapiens GN=FARSLA PE=2 SV=1	FARSLA	58 kDa	0.061	0.3	[]	4	3	1	1
cDNA FLJ53750, highly similar to 60S ribosomal protein L8 OS=Homo sapiens PE=2 SV=1		21 kDa	0.85	1	[]	4	3	3	3
Ribosomal protein L13a variant (Fragment) OS=Homo sapiens PE=2 SV=1		24 kDa	0.03	0.8	Fibro-BKO low, Fibro-Naive high	3	3	2	2
cDNA FLJ54022, highly similar to Exportin-2 OS=Homo sapiens PE=2 SV=1		86 kDa	0.15	0.2	[]	4	9	1	1
Core histone macro-H2A OS=Homo sapiens GN=H2AFY2 PE=4 SV=1	H2AFY2	40 kDa	0.064	0.1	[]	15	11	3	0
Coatomer subunit gamma OS=Homo sapiens PE=2 SV=1		98 kDa	0.08	0.2	[]	10	8	3	0
Periostin, osteoblast specific factor, isoform CRA_c OS=Homo sapiens GN=POSTN PE=4 SV=1	POSTN	93 kDa	0.036	11	Fibro-BKO high, Fibro-Naive low	0	1	5	4
40S ribosomal protein S7 OS=Homo sapiens GN=RPS7 PE=1 SV=1	RPS7	21 kDa	0.073	0.1	[]	6	4	0	1
Heterogeneous nuclear ribonucleoprotein A1, isoform CRA_b OS=Homo sapiens GN=HNRPA1 PE=4 SV=1	HNRPA1	39 kDa	0.027	0	Fibro-BKO low, Fibro-Naive high	4	6	0	0
RSL1D1 protein (Fragment) OS=Homo sapiens GN=RSL1D1 PE=2 SV=1	RSL1D1	49 kDa	0.015	0	Fibro-BKO low, Fibro-Naive high	6	5	0	0
cDNA FLJ54170, highly similar to Cytosolic nonspecific dipeptidase OS=Homo sapiens PE=2 SV=1		52 kDa	0.037	0	Fibro-BKO low, Fibro-Naive high	5	8	0	0
Ig lambda-2 chain C regions (Fragment) OS=Homo sapiens GN=IGLC2 PE=4 SV=1	IGLC2	11 kDa	0.36	INF	[]	0	0	2	26
cDNA FLJ55253, highly similar to Actin, cytoplasmic 1 OS=Homo sapiens PE=2 SV=1		39 kDa	0.34	1.2	[]	55	42	48	59
C-type lysozyme OS=Homo sapiens GN=LYZ PE=2 SV=1	LYZ	17 kDa	0.75	1.1	[]	3	2	3	2
Calcium-transporting ATPase OS=Homo sapiens GN=ATP2B1 PE=3 SV=1	ATP2B1	135 kDa	0.77	1.1	[]	3	8	6	5
Golgi apparatus protein 1 OS=Homo sapiens GN=GLG1 PE=1 SV=1	GLG1	79 kDa	0.37	0.8	[]	3	4	2	3
Profilin OS=Homo sapiens GN=PFN1 PE=1 SV=1	PFN1	11 kDa	0.88	1	[]	6	5	5	5
Uncharacterized protein OS=Homo sapiens GN=LOC93343 PE=4 SV=1	LOC93343	29 kDa	0.77	1.1	[]	3	3	2	4
Ubiquitin-like protein ISG15 (Fragment) OS=Homo sapiens GN=ISG15 PE=1 SV=6	ISG15	16 kDa	0.53	0.8	[]	4	3	3	2
UMP-CMP kinase OS=Homo sapiens GN=CMPK PE=2 SV=1	CMPK	26 kDa	0.51	1.4	[]	1	3	2	3
Tubulin alpha chain OS=Homo sapiens PE=2 SV=1		43 kDa	0.3	0.8	[]	44	36	24	32
Synaptosomal-associated protein OS=Homo sapiens GN=SNAP23 PE=2 SV=1	SNAP23	23 kDa	0.31	1.7	[]	2	2	2	4
Guanine nucleotide-binding protein G(i) subunit alpha-1 (Fragment) OS=Homo sapiens GN=GNAI1 PE=1 SV=1	GNAI1	16 kDa	0.23	1.9	[]	2	4	6	4
N-myc downstream regulated gene 1, isoform CRA_b OS=Homo sapiens GN=NDRG1 PE=4 SV=1	NDRG1	39 kDa	0.55	0.7	[]	4	2	2	2
TOM1-like protein 1 OS=Homo sapiens GN=TOM1L1 PE=1 SV=1	TOM1L1	52 kDa	0.081	2.2	[]	2	1	3	3
Annexin OS=Homo sapiens GN=ANXA3 PE=1 SV=1	ANXA3	32 kDa	0.041	0.4	Fibro-BKO low, Fibro-Naive high	9	10	4	2
Epididymis secretory protein Li 303 OS=Homo sapiens GN=HEL-S-303 PE=2 SV=1	HEL-S-303	26 kDa	0.18	0.6	[]	3	3	2	1
Alpha-1,4 glucan phosphorylase OS=Homo sapiens PE=2 SV=1		97 kDa	0.065	0.3	[]	12	19	4	3
Ubiquitin specific peptidase 9, X-linked, isoform CRA_b OS=Homo sapiens GN=USP9X PE=3 SV=1	USP9X	271 kDa	0.34	0.7	[]	2	3	1	2

Related RAS viral (R-ras) oncogene homolog, isoform CRA_a OS=Homo sapiens GN=RRAS PE=4 SV=1	RRAS	23 kDa	0.42	2.3	[]	1	3	6	2
Equilibrative nucleoside transporter 1 variant ata (Fragment) OS=Homo sapiens GN=SLC29A1 PE=4 SV=1	SLC29A1	8 kDa	0.11	2	[]	2	2	4	3
Testis tissue sperm-binding protein Li 69n OS=Homo sapiens PE=2 SV=1		35 kDa	0.39	0.7	[]	3	2	1	2
Fructose-1,6-bisphosphatase 1 OS=Homo sapiens GN=FBP1 PE=2 SV=1	FBP1	37 kDa	0.044	0.4	Fibro-BKO low, Fibro-Naive high	4	5	1	2
Tryptophanyl-tRNA synthetase, isoform CRA_a OS=Homo sapiens GN=WARS PE=3 SV=1	WARS	53 kDa	0.14	0.5	[]	3	3	2	1
Protein diaphanous homolog 1 OS=Homo sapiens GN=DIAPH1 PE=1 SV=1	DIAPH1	141 kDa	0.082	0.3	[]	3	5	1	1
cDNA, FLJ93507, highly similar to Homo sapiens ATPase, H+ transporting, lysosomal 38kDa, V0 subunit d isoform 1 (ATP6V0D1), mRNA OS=		40 kDa	0.22	0.3	[]	4	3	2	0
MHC class I antigen (Fragment) OS=Homo sapiens GN=HLA-B PE=3 SV=1	HLA-B	21 kDa	0.24	0.7	[]	5	5	2	4
CD276 antigen OS=Homo sapiens GN=CD276 PE=1 SV=1	CD276	42 kDa	0.45	1.7	[]	1	3	4	2
FLJ00095 protein (Fragment) OS=Homo sapiens GN=FLJ00095 PE=2 SV=1	FLJ00095	16 kDa	0.0082	0.6	Fibro-BKO low, Fibro-Naive high	4	4	2	2
Proteasome subunit beta type-5 (Fragment) OS=Homo sapiens GN=PSMB5 PE=1 SV=1	PSMB5	13 kDa	0.2	3.5	[]	1	2	6	3
cDNA FLJ57801, highly similar to Homo sapiens AHNAK nucleoprotein (desmoyokin) (AHNAK), transcript variant 1, mRNA (Fragment) OS=Ho		114 kDa	0.032	5.1	Fibro-BKO high, Fibro-Naive low	1	1	5	4
Cellular retinoic acid-binding protein 2 (Fragment) OS=Homo sapiens GN=CRABP2 PE=1 SV=1	CRABP2	9 kDa	0.23	0.6	[]	4	4	1	3
cDNA, FLJ94534, highly similar to Homo sapiens capping protein (actin filament), gelsolin-like(CAPG), mRNA OS=Homo sapiens PE=2 SV=1		38 kDa	0.002	0.2	Fibro-BKO low, Fibro-Naive high	5	5	1	1
cDNA, FLJ94267, highly similar to Homo sapiens glutathione S-transferase omega 1 (GSTO1), mRNA OS=Homo sapiens PE=2 SV=1		28 kDa	0.029	0.1	Fibro-BKO low, Fibro-Naive high	4	4	1	0
Histone H3 OS=Homo sapiens GN=HIST2H3PS2 PE=1 SV=1	HIST2H3PS2	15 kDa	0.14	0.3	[]	9	7	0	4
cDNA FLJ56108, highly similar to Puromycin-sensitive aminopeptidase (EC 3.4.11.-) OS=Homo sapiens PE=2 SV=1		93 kDa	0.046	0.1	Fibro-BKO low, Fibro-Naive high	5	4	1	0
FBL protein (Fragment) OS=Homo sapiens GN=FBL PE=2 SV=2	FBL	28 kDa	0.043	0.1	Fibro-BKO low, Fibro-Naive high	5	4	0	1
40S ribosomal protein S9 OS=Homo sapiens GN=RPS9 PE=1 SV=1	RPS9	23 kDa	0.0011	0	Fibro-BKO low, Fibro-Naive high	4	4	0	0
Poly [ADP-ribose] polymerase OS=Homo sapiens GN=PARP1 PE=4 SV=1	PARP1	113 kDa	0.0033	0	Fibro-BKO low, Fibro-Naive high	5	6	0	0
Procollagen C-endopeptidase enhancer OS=Homo sapiens GN=PCOLCE PE=4 SV=1	PCOLCE	48 kDa	0.026	INF	Fibro-BKO high, Fibro-Naive low	0	0	4	6
cDNA FLJ78586, highly similar to Homo sapiens VAMP (vesicle-associated membrane protein)-associated protein A, 33kDa (VAPA), mRNA O		27 kDa	0.015	0	Fibro-BKO low, Fibro-Naive high	6	5	0	0
Interferon-induced GTP-binding protein Mx1 OS=Homo sapiens GN=MX1 PE=1 SV=1	MX1	73 kDa	0.013	INF	Fibro-BKO high, Fibro-Naive low	0	0	7	6
Peroxidasin homolog (Fragment) OS=Homo sapiens GN=PXDN PE=1 SV=1	PXDN	80 kDa	0.017	INF	Fibro-BKO high, Fibro-Naive low	0	0	5	7
Myosin regulatory light chain 12A OS=Homo sapiens GN=MYL12A PE=1 SV=1	MYL12A	20 kDa	0.071	0	[]	5	3	0	0
Proliferating cell nuclear antigen (Fragment) OS=Homo sapiens GN=PCNA PE=2 SV=1	PCNA	29 kDa	0.015	0	Fibro-BKO low, Fibro-Naive high	6	5	0	0
Glycylpeptide N-tetradecanoyltransferase (Fragment) OS=Homo sapiens GN=NMT1 PE=2 SV=2	NMT1	57 kDa	0.02	0	Fibro-BKO low, Fibro-Naive high	5	4	0	0
Serine/arginine-rich-splicing factor 1 OS=Homo sapiens GN=SRSF1 PE=1 SV=1	SRSF1	28 kDa	0.071	0	[]	5	3	0	0
Actin-related protein 2/3 complex subunit 3 OS=Homo sapiens PE=2 SV=1		21 kDa	0.0011	0	Fibro-BKO low, Fibro-Naive high	4	4	0	0
cDNA FLJ35987 fis, clone TESTI2014269, highly similar to D-3-phosphoglycerate dehydrogenase (EC 1.1.1.95) OS=Homo sapiens PE=2 SV=1		53 kDa	0.74	1.1	[]	3	2	2	3
Proteasome subunit beta type OS=Homo sapiens PE=2 SV=1		29 kDa	0.74	1.1	[]	3	2	2	3
ATP-dependent RNA helicase DDX3X OS=Homo sapiens GN=DDX3X PE=1 SV=1	DDX3X	81 kDa	0.34	1.4	[]	2	2	3	2
Truncated plakophilin-2 OS=Homo sapiens GN=PKP2 PE=1 SV=1	PKP2	91 kDa	0.55	1.3	[]	3	2	2	3
Ubiquitin-conjugating enzyme E2D 3 (UBC4/5 homolog, yeast), isoform CRA_a OS=Homo sapiens GN=UBE2D3 PE=3 SV=	UBE2D3	17 kDa	0.7	1.1	[]	2	3	3	2
ATP-dependent 6-phosphofructokinase, platelet type (Fragment) OS=Homo sapiens GN=PFKP PE=1 SV=1	PFKP	23 kDa	0.75	1.1	[]	3	2	3	2
Annexin (Fragment) OS=Homo sapiens GN=ANXA6 PE=2 SV=1	ANXA6	75 kDa	0.00056	0.3	Fibro-BKO low, Fibro-Naive high	59	63	15	18
Junctional adhesion molecule A OS=Homo sapiens GN=F11R PE=1 SV=1	F11R	30 kDa	0.16	0.7	[]	8	11	6	6
cDNA FLJ51818, highly similar to Phosphoglucomutase-1 (EC 5.4.2.2) OS=Homo sapiens PE=2 SV=1		59 kDa	0.83	0.9	[]	2	3	1	3
Serine/threonine-protein phosphatase OS=Homo sapiens PE=2 SV=1		36 kDa	0.32	0.6	[]	3	5	1	3
Aldo-keto reductase family 1 member C3 OS=Homo sapiens GN=AKR1C3 PE=1 SV=1	AKR1C3	37 kDa	0.18	0.4	[]	2	4	1	1
Tumor rejection antigen (Gp96) 1 OS=Homo sapiens GN=TRA1 PE=2 SV=1	TRA1	92 kDa	0.33	0.6	[]	2	4	1	2
Calpain small subunit 1 OS=Homo sapiens GN=CAPNS1 PE=1 SV=1	CAPNS1	34 kDa	0.011	1.7	Fibro-BKO high, Fibro-Naive low	2	2	3	3
Amino acid transporter OS=Homo sapiens PE=2 SV=1		56 kDa	0.53	1.9	[]	1	2	4	1
Interferon-induced GTP-binding protein Mx1 (Fragment) OS=Homo sapiens GN=MX1 PE=1 SV=1	MX1	21 kDa	0.11	2.8	[]	2	2	6	4
Heterogeneous nuclear ribonucleoprotein U-like 1, isoform CRA_a OS=Homo sapiens GN=HNRPUL1 PE=4 SV=1	HNRPUL1	85 kDa	0.18	0.5	[]	4	3	2	1
cDNA FLJ90427 fis, clone NT2RP3000481, highly similar to Importin-7 OS=Homo sapiens PE=2 SV=1		83 kDa	0.7	1.1	[]	2	3	3	2
cDNA FLJ44621 fis, clone BRACE2016896, highly similar to Lysyl-tRNA synthetase (EC 6.1.1.6) OS=Homo sapiens PE=2 SV=1		48 kDa	0.12	0.3	[]	5	3	1	1
Pro-cathepsin H OS=Homo sapiens GN=CTSH PE=1 SV=1	CTSH	36 kDa	0.45	0.4	[]	5	1	1	1
CUB domain containing protein 1 (Fragment) OS=Homo sapiens PE=2 SV=1		72 kDa	0.66	0.7	[]	1	4	1	2
S-methyl-5'-thioadenosine phosphorylase OS=Homo sapiens GN=MTAP-ANRIL fusion PE=2 SV=1	MTAP-ANRIL	30 kDa	0.23	0.4	[]	4	2	1	1
Cytoplasmic dynein 1 heavy chain 1 (Fragment) OS=Homo sapiens GN=DYNC1H1 PE=1 SV=1	DYNC1H1	21 kDa	0.13	4.1	[]	0	2	4	3
Solute carrier family 38, member 2, isoform CRA_b OS=Homo sapiens GN=SLC38A2 PE=4 SV=1	SLC38A2	56 kDa	0.033	8.1	Fibro-BKO high, Fibro-Naive low	0	1	3	4
cDNA FLJ76855, highly similar to Homo sapiens exportin 7 (XPO7), mRNA OS=Homo sapiens PE=2 SV=1		124 kDa	0.15	0.1	[]	6	3	1	0
FARSB protein (Fragment) OS=Homo sapiens GN=FARSB PE=2 SV=2	FARSB	66 kDa	0.075	0.2	[]	4	3	1	0
Actin-like 6A isoform 1 variant (Fragment) OS=Homo sapiens PE=2 SV=1		47 kDa	0.2	0.2	[]	2	5	1	0
Putative uncharacterized protein DKFZp686M1483 (Fragment) OS=Homo sapiens GN=DKFZp686M1483 PE=2 SV=1	DKFZp686M1483	33 kDa	0.17	12	[]	0	1	6	3
F-actin-capping protein subunit beta OS=Homo sapiens GN=CAPZB PE=1 SV=1	CAPZB	29 kDa	0.19	0.2	[]	2	5	0	1
cDNA FLJ75877, highly similar to Homo sapiens 5'-nucleotidase, cytosolic II (NT5C2), mRNA OS=Homo sapiens PE=2 SV=1		65 kDa	0.11	0.1	[]	3	6	0	1
Nicotinate phosphoribosyltransferase OS=Homo sapiens GN=NAPRT PE=1 SV=1	NAPRT	62 kDa	0.03	0.1	Fibro-BKO low, Fibro-Naive high	6	5	0	1

Testis tissue sperm-binding protein Li 44a OS=Homo sapiens GN=WDR77 PE=2 SV=1	WDR77	37 kDa	0.11	0	[]	6	3	0	0
Ribosomal protein, large, P2, isoform CRA_a OS=Homo sapiens GN=RPLP2 PE=3 SV=1	RPLP2	12 kDa	0.05	0	Fibro-BKO low, Fibro-Naive high	6	4	0	0
Putative uncharacterized protein DKFZp686D12108 (Fragment) OS=Homo sapiens GN=DKFZp686D12108 PE=2 SV=1	DKFZp686D12108	130 kDa	0.0014	INF	Fibro-BKO high, Fibro-Naive low	0	0	17	17
HCG2025883, isoform CRA_b OS=Homo sapiens GN=POLR2C PE=2 SV=1	POLR2C	31 kDa	0.081	0	[]	7	4	0	0
Complement C1s subcomponent OS=Homo sapiens GN=C1S PE=1 SV=1	C1S	76 kDa	0.31	INF	[]	0	0	1	7
Annexin OS=Homo sapiens PE=2 SV=1		40 kDa	0.12	1.8	[]	8	5	11	10
Tubulin beta chain OS=Homo sapiens PE=2 SV=1		50 kDa	0.28	0.8	[]	71	55	38	47
Alpha-1,4 glucan phosphorylase OS=Homo sapiens PE=2 SV=1		86 kDa	0.0043	0.4	Fibro-BKO low, Fibro-Naive high	67	66	21	22
UDP-glucose 6-dehydrogenase (Fragment) OS=Homo sapiens GN=UGDH PE=1 SV=1	UGDH	14 kDa	0.7	1.1	[]	2	3	3	2
Transmembrane BAX inhibitor motif containing 1, isoform CRA_a OS=Homo sapiens GN=TMBIM1 PE=3 SV=1	TMBIM1	35 kDa	0.31	1.7	[]	2	2	2	4
Secretory carrier-associated membrane protein OS=Homo sapiens PE=2 SV=1		37 kDa	0.03	0.8	Fibro-BKO low, Fibro-Naive high	3	3	2	2
Solute carrier family 7 (Cationic amino acid transporter, y+ system), member 1, isoform CRA_a OS=Homo sapiens GN=SL	SLC7A1	68 kDa	0.34	1.4	[]	2	2	3	2
Syndecan binding protein (Syntenin), isoform CRA_a OS=Homo sapiens GN=SDCBP PE=1 SV=1	SDCBP	35 kDa	0.006	1.3	Fibro-BKO high, Fibro-Naive low	84	88	97	100
6-phosphogluconate dehydrogenase, decarboxylating OS=Homo sapiens PE=2 SV=1		51 kDa	0.27	0.4	[]	4	9	3	2
6-phosphogluconate dehydrogenase, decarboxylating OS=Homo sapiens PE=2 SV=1		47 kDa	0.073	0.4	[]	6	9	2	3
Beta globin (Fragment) OS=Homo sapiens PE=3 SV=1		11 kDa	0.58	0.7	[]	5	3	1	4
cDNA FLJ58636, moderately similar to Atlastin OS=Homo sapiens PE=2 SV=1		59 kDa	0.53	1.4	[]	1	3	3	2
Solute carrier family 16 (Monocarboxylic acid transporters), member 3, isoform CRA_a OS=Homo sapiens GN=SLC16A3 F	SLC16A3	49 kDa	0.83	0.9	[]	2	3	1	3
Coronin OS=Homo sapiens GN=CORO1B PE=3 SV=1	CORO1B	54 kDa	0.66	0.7	[]	1	4	1	2
Sodium/potassium-transporting ATPase subunit beta-3 (Fragment) OS=Homo sapiens GN=ATP1B3 PE=1 SV=1	ATP1B3	15 kDa	0.23	0.4	[]	4	2	1	1
Vesicle docking protein p115, isoform CRA_a OS=Homo sapiens GN=VDP PE=4 SV=1	VDP	108 kDa	0.92	1.1	[]	3	1	1	3
SEC31-like 1 (S. cerevisiae), isoform CRA_d OS=Homo sapiens GN=SEC31L1 PE=4 SV=1	SEC31L1	133 kDa	0.89	1.1	[]	3	1	3	1
cDNA FLJ56381, highly similar to Dynamin-1-like protein (EC 3.6.5.5) OS=Homo sapiens PE=2 SV=1		85 kDa	0.58	0.8	[]	2	2	1	2
Beta-2-microglobulin OS=Homo sapiens PE=2 SV=1		14 kDa	0.67	0.7	[]	4	1	2	1
Integrin-linked protein kinase OS=Homo sapiens GN=ILK PE=1 SV=1	ILK	55 kDa	0.39	3.3	[]	1	1	1	5
40S ribosomal protein S3a OS=Homo sapiens GN=RPS3A PE=2 SV=1	RPS3A	30 kDa	0.082	0.3	[]	3	5	1	1
Coatomer subunit beta' (Fragment) OS=Homo sapiens GN=COPB2 PE=1 SV=1	COPB2	20 kDa	0.18	0.5	[]	3	3	2	1
cDNA FLJ52703, highly similar to Asparaginyl-tRNA synthetase, cytoplasmic (EC6.1.1.22) OS=Homo sapiens PE=2 SV=1		35 kDa	0.1	0.5	[]	3	4	1	2
Apolipoprotein E isoform 4 (Fragment) OS=Homo sapiens GN=APOE PE=2 SV=1	APOE	15 kDa	0.14	3.3	[]	1	1	2	4
Pyridoxal kinase OS=Homo sapiens GN=PDXK PE=1 SV=1	PDXK	31 kDa	0.38	0.6	[]	4	2	1	2
Adenylosuccinate synthetase isozyme 2 OS=Homo sapiens GN=ADSS PE=3 SV=1	ADSS	50 kDa	0.025	0.3	Fibro-BKO low, Fibro-Naive high	3	4	1	1
Lactadherin OS=Homo sapiens GN=MFGE8 PE=1 SV=1	MFGE8	38 kDa	0.0028	6.9	Fibro-BKO high, Fibro-Naive low	10	11	64	64
Carbonyl reductase [NADPH] 1 OS=Homo sapiens GN=CBR1 PE=1 SV=1	CBR1	24 kDa	0.22	0.3	[]	4	3	2	0
Large neutral amino acids transporter 1 (Fragment) OS=Homo sapiens GN=SLC7A5 PE=4 SV=1	SLC7A5	7 kDa	0.38	0.5	[]	3	4	0	3
Alpha-2-antiplasmin OS=Homo sapiens GN=SERPINF2 PE=1 SV=1	SERPINF2	48 kDa	0.036	7.6	Fibro-BKO high, Fibro-Naive low	1	0	3	4
Neurofibromin (Fragment) OS=Homo sapiens GN=NF1 PE=1 SV=1	NF1	281 kDa	0.26	3.5	[]	0	2	4	2
Tight junction protein 2 (Zona occludens 2), isoform CRA_a OS=Homo sapiens GN=TJP2 PE=4 SV=1	TJP2	134 kDa	0.94	1.1	[]	1	2	0	3
cDNA FLJ55635, highly similar to pre-mRNA-splicing factorATP-dependent RNA helicase DHX15 (EC 3.6.1.-) OS=Homo sapiens PE=2 SV=1		90 kDa	0.13	0.2	[]	3	2	0	1
Aflatoxin B1 aldehyde reductase member 2 (Fragment) OS=Homo sapiens GN=AKR7A2 PE=1 SV=1	AKR7A2	35 kDa	0.029	0.1	Fibro-BKO low, Fibro-Naive high	4	4	1	0
cDNA, FLJ79540, highly similar to Serine-threonine kinase receptor-associatedprotein OS=Homo sapiens PE=2 SV=1		29 kDa	0.025	0.1	Fibro-BKO low, Fibro-Naive high	4	4	0	1
Plexin-B2 (Fragment) OS=Homo sapiens GN=PLXNB2 PE=1 SV=1	PLXNB2	52 kDa	0.48	2.4	[]	0	2	3	1
N-alpha-acetyltransferase 15, NatA auxiliary subunit OS=Homo sapiens GN=NAA15 PE=1 SV=1	NAA15	101 kDa	0.12	0.2	[]	2	3	1	0
Casein kinase II subunit beta OS=Homo sapiens GN=CSNK2B PE=2 SV=1	CSNK2B	25 kDa	0.046	0.1	Fibro-BKO low, Fibro-Naive high	5	4	1	0
Collagen alpha-2(VI) chain (Fragment) OS=Homo sapiens GN=COL6A2 PE=1 SV=1	COL6A2	20 kDa	0.021	INF	Fibro-BKO high, Fibro-Naive low	0	0	5	4
Quinone oxidoreductase (Fragment) OS=Homo sapiens GN=CRYZ PE=1 SV=1	CRYZ	26 kDa	0.071	0	[]	5	3	0	0
MHC class I antigen OS=Homo sapiens GN=HLA-A PE=3 SV=1	HLA-A	41 kDa	0.1	0.6	[]	11	14	5	8
Tumor susceptibility gene 101 protein OS=Homo sapiens GN=TSG101 PE=1 SV=1	TSG101	41 kDa	0.47	0.9	[]	5	6	4	5
Afadin OS=Homo sapiens GN=AFDN PE=1 SV=2	AFDN	202 kDa	0.23	1.4	[]	2	2	2	3
HCG2043376, isoform CRA_b OS=Homo sapiens GN=hCG_2043376 PE=4 SV=1	hCG_2043376	22 kDa	0.42	0.7	[]	11	6	4	6
Plectin 1, intermediate filament binding protein 500kDa, isoform CRA_b OS=Homo sapiens GN=PLEC1 PE=4 SV=1	PLEC1	234 kDa	0.14	1.1	[]	2	2	2	2
Heterogeneous nuclear ribonucleoprotein H OS=Homo sapiens GN=HNRNPH1 PE=1 SV=1	HNRNPH1	47 kDa	0.51	0.9	[]	5	7	5	4
cDNA FLJ61453, highly similar to Adapter-relatedprotein complex 2 alpha- 1 subunit OS=Homo sapiens PE=2 SV=1		55 kDa	0.39	0.7	[]	3	2	1	2
Putative uncharacterized protein DKFZp434M052 (Fragment) OS=Homo sapiens GN=DKFZp434M052 PE=2 SV=1	DKFZp434M052	16 kDa	0.43	0.7	[]	5	4	2	4
FASN protein (Fragment) OS=Homo sapiens GN=FASN PE=2 SV=1	FASN	84 kDa	0.35	1.3	[]	22	37	34	34
RAB8A, member RAS oncogene family, isoform CRA_a OS=Homo sapiens GN=RAB8A PE=4 SV=1	RAB8A	24 kDa	0.13	0.7	[]	4	4	2	3
Lymphocyte antigen 75 variant (Fragment) OS=Homo sapiens PE=2 SV=1		155 kDa	0.33	0.6	[]	2	4	1	2
Alanine--tRNA ligase, cytoplasmic (Fragment) OS=Homo sapiens GN=AARS PE=1 SV=3	AARS	30 kDa	0.12	0.6	[]	3	3	1	2
Eukaryotic translation initiation factor 3 subunit B OS=Homo sapiens GN=EIF3S9 PE=3 SV=1	EIF3S9	92 kDa	0.23	0.4	[]	4	2	1	1
Nectin-2 (Fragment) OS=Homo sapiens GN=NECTIN2 PE=1 SV=1	NECTIN2	23 kDa	0.081	2.2	[]	2	1	3	3

KRT8 protein (Fragment) OS=Homo sapiens GN=KRT8 PE=2 SV=2	KRT8	41 kDa	0.059	0.3	[]	13	10	3	4
cDNA FLJ76813, highly similar to Homo sapiens acetyl-Coenzyme A acetyltransferase 2 (acetoacetyl Coenzyme A thiolase), mRNA OS=Homo		41 kDa	0.32	1.5	[]	2	1	2	2
Hunc18b2 OS=Homo sapiens PE=2 SV=1		65 kDa	0.18	0.6	[]	3	3	2	1
Tight junction protein ZO-1 OS=Homo sapiens GN=TJP1 PE=1 SV=1	TJP1	188 kDa	0.88	1.1	[]	1	3	1	3
GIPC PDZ domain containing family, member 1, isoform CRA_a OS=Homo sapiens GN=GIPC1 PE=4 SV=1	GIPC1	36 kDa	0.053	4	[]	1	1	4	3
Transmembrane 9 superfamily member OS=Homo sapiens GN=TM9SF2 PE=2 SV=1	TM9SF2	76 kDa	0.86	1.1	[]	3	1	2	2
Epiplakin OS=Homo sapiens GN=EPPK1 PE=1 SV=2	EPPK1	553 kDa	0.43	2	[]	1	3	5	2
Leucine rich repeat containing 54, isoform CRA_a OS=Homo sapiens GN=LRRC54 PE=4 SV=1	LRRC54	38 kDa	0.061	0.3	[]	4	3	1	1
cDNA, FLJ94921, highly similar to Homo sapiens prolyl endopeptidase (PREP), mRNA OS=Homo sapiens PE=2 SV=1		81 kDa	0.18	0.4	[]	2	4	1	1
Polypyrimidine tract-binding protein 1 OS=Homo sapiens GN=PTBP1 PE=1 SV=1	PTBP1	62 kDa	0.061	0.3	[]	4	3	1	1
Testicular tissue protein Li 183 OS=Homo sapiens PE=2 SV=1		22 kDa	0.18	0.6	[]	3	3	2	1
Ribosomal protein L23, isoform CRA_b OS=Homo sapiens GN=RPL23 PE=3 SV=1	RPL23	15 kDa	0.41	0.6	[]	4	2	2	1
Synaptogyrin OS=Homo sapiens GN=SYNGR2 PE=3 SV=1	SYNGR2	25 kDa	0.0082	0.6	Fibro-BKO low, Fibro-Naive high	2	2	1	1
Transmembrane 9 superfamily member OS=Homo sapiens PE=2 SV=1		75 kDa	0.23	1.5	[]	1	2	2	2
cDNA FLJ58258, highly similar to Cyclin G-associated kinase (EC 2.7.11.1) OS=Homo sapiens PE=2 SV=1		132 kDa	0.7	0.9	[]	3	2	2	2
Toll interacting protein variant (Fragment) OS=Homo sapiens PE=2 SV=1		39 kDa	0.14	3.3	[]	1	1	2	4
cDNA FLJ76064 OS=Homo sapiens PE=2 SV=1		35 kDa	0.0034	0.4	Fibro-BKO low, Fibro-Naive high	3	3	1	1
U2 small nuclear RNA auxillary factor 1 isoform a OS=Homo sapiens GN=U2AF1 PE=2 SV=1	U2AF1	28 kDa	0.46	0.4	[]	4	1	1	1
Nucleosome assembly protein 1-like 4, isoform CRA_b OS=Homo sapiens GN=NAP1L4 PE=3 SV=1	NAP1L4	43 kDa	0.55	0.6	[]	2	5	1	3
Elongation factor 1-delta OS=Homo sapiens GN=EEF1D PE=1 SV=1	EEF1D	69 kDa	0.22	0.3	[]	4	3	2	0
Epididymis secretory protein Li 71 OS=Homo sapiens GN=HEL-S-71 PE=2 SV=1	HEL-S-71	17 kDa	0.37	0.4	[]	3	2	0	2
Transmembrane protein 2, isoform CRA_b OS=Homo sapiens GN=TMEM2 PE=4 SV=1	TMEM2	154 kDa	0.23	2.9	[]	0	2	2	3
Na(+)/H(+) exchange regulatory cofactor NHE-RF OS=Homo sapiens PE=2 SV=1		39 kDa	0.17	0.3	[]	3	4	0	2
cDNA, FLJ94229, highly similar to Homo sapiens heterogeneous nuclear ribonucleoprotein L (HNRPL),mRNA OS=Homo sapiens PE=2 SV=1		60 kDa	0.22	0.4	[]	3	3	0	2
Ubiquitin carboxyl-terminal hydrolase OS=Homo sapiens PE=2 SV=1		26 kDa	0.19	0.3	[]	4	3	0	2
NPD011 OS=Homo sapiens GN=NPD011 PE=2 SV=1	NPD011	24 kDa	0.34	0.3	[]	1	3	1	0
Vitamin D-binding protein OS=Homo sapiens GN=GC PE=1 SV=1	GC	39 kDa	0.018	8.7	Fibro-BKO high, Fibro-Naive low	1	0	4	4
Epidermal growth factor receptor kinase substrate 8-like protein 1 OS=Homo sapiens GN=EPS8L1 PE=1 SV=1	EPS8L1	73 kDa	0.51	0.4	[]	1	5	2	0
Uncharacterized protein (Fragment) OS=Homo sapiens PE=4 SV=2		27 kDa	0.053	0.2	[]	3	4	1	0
Transaldolase OS=Homo sapiens PE=2 SV=1		38 kDa	0.025	0.1	Fibro-BKO low, Fibro-Naive high	4	4	0	1
Heterogeneous nuclear ribonucleoprotein D (AU-rich element RNA binding protein 1, 37kDa), isoform CRA_c OS=Homo	HNRPD	36 kDa	0.14	0.2	[]	3	2	1	0
DNA-directed RNA polymerases I, II, and III subunit RPABC3 (Fragment) OS=Homo sapiens GN=POLR2H PE=1 SV=8	POLR2H	17 kDa	0.069	0.2	[]	4	3	0	1
Eukaryotic translation initiation factor 5A (Fragment) OS=Homo sapiens GN=EIF5A PE=1 SV=8	EIF5A	16 kDa	0.081	0	[]	2	4	0	0
Actin-like protein (Fragment) OS=Homo sapiens GN=ACT PE=4 SV=1	ACT	11 kDa	0.35	0.5	[]	6	6	0	5
Tenascin C (Hexabrachion), isoform CRA_a OS=Homo sapiens GN=TNC PE=4 SV=1	TNC	241 kDa	0.00028	INF	Fibro-BKO high, Fibro-Naive low	0	0	44	49
Tubulin gamma chain OS=Homo sapiens GN=TUBG2 PE=2 SV=1	TUBG2	52 kDa	0.03	0	Fibro-BKO low, Fibro-Naive high	4	3	0	0
ATP synthase subunit alpha OS=Homo sapiens GN=HEL-S-123m PE=2 SV=1	HEL-S-123m	60 kDa	0.0011	0	Fibro-BKO low, Fibro-Naive high	4	4	0	0
Tenascin OS=Homo sapiens GN=TNC PE=1 SV=1	TNC	221 kDa	0.00024	INF	Fibro-BKO high, Fibro-Naive low	0	0	45	50
Malate dehydrogenase OS=Homo sapiens GN=HEL-S-32 PE=2 SV=1	HEL-S-32	36 kDa	0.012	0	Fibro-BKO low, Fibro-Naive high	3	4	0	0
cDNA, FLJ93750, Homo sapiens DEAD (Asp-Glu-Ala-Asp) box polypeptide 6 (DDX6), mRNA OS=Homo sapiens PE=2 SV=1		53 kDa	0.17	0	[]	2	6	0	0
Serpin peptidase inhibitor, clade B (Ovalbumin), member 6, isoform CRA_a OS=Homo sapiens GN=SERPINB6 PE=3 SV=1	SERPINB6	43 kDa	0.0011	0	Fibro-BKO low, Fibro-Naive high	3	3	0	0
Complement component 1, q subcomponent, B chain, isoform CRA_a OS=Homo sapiens GN=C1QB PE=4 SV=1	C1QB	27 kDa	0.13	INF	[]	0	0	2	5
Proliferation-associated 2G4, 38kDa, isoform CRA_a OS=Homo sapiens GN=PA2G4 PE=4 SV=1	PA2G4	44 kDa	0.11	0	[]	4	2	0	0
Ribosomal protein L4 variant (Fragment) OS=Homo sapiens PE=2 SV=1		49 kDa	0.037	0.4	Fibro-BKO low, Fibro-Naive high	16	15	7	5
Filamin B, beta (Actin binding protein 278), isoform CRA_a OS=Homo sapiens GN=FLNB PE=4 SV=1	FLNB	280 kDa	0.23	0.3	[]	12	29	6	6
Neutral alpha-glucosidase AB OS=Homo sapiens GN=GANAB PE=1 SV=1	GANAB	97 kDa	0.7	0.8	[]	35	14	20	16
Histone H2B OS=Homo sapiens GN=HIST1H2BJ PE=3 SV=1	HIST1H2BJ	14 kDa	0.17	0.4	[]	71	42	13	24
cDNA FLJ51655, highly similar to Actin-like protein 2 OS=Homo sapiens PE=2 SV=1		35 kDa	0.0034	0.4	Fibro-BKO low, Fibro-Naive high	6	6	2	2
MYH14 protein OS=Homo sapiens GN=MYH14 PE=1 SV=1	MYH14	73 kDa	0.088	0.6	[]	3	4	2	2
Reticulon OS=Homo sapiens GN=RTN4 PE=1 SV=1	RTN4	37 kDa	0.63	0.8	[]	9	4	4	5
Non-specific serine/threonine protein kinase OS=Homo sapiens PE=2 SV=1		81 kDa	0.18	1.9	[]	1	2	2	3
Guanine nucleotide binding protein (G protein), q polypeptide, isoform CRA_c OS=Homo sapiens GN=GNAQ PE=4 SV=1	GNAQ	42 kDa	0.79	1.1	[]	1	3	2	2
Trifunctional purine biosynthetic protein adenosine-3 OS=Homo sapiens GN=GART PE=2 SV=1	GART	108 kDa	0.69	0.9	[]	2	2	2	1
CTP synthase OS=Homo sapiens PE=2 SV=1		50 kDa	0.45	0.7	[]	3	2	2	1
Ribosomal protein S23, isoform CRA_a OS=Homo sapiens GN=RPS23 PE=2 SV=1	RPS23	16 kDa	0.81	0.8	[]	1	3	1	2
cDNA FLJ42082 fis, clone TCERX2000171, highly similar to CLAUDIN-4 OS=Homo sapiens PE=2 SV=1		21 kDa	0.73	0.9	[]	8	5	6	4
Lactoferrin OS=Homo sapiens GN=LTF PE=2 SV=1	LTF	78 kDa	0.18	1.9	[]	1	2	2	3
Epididymis secretory sperm binding protein Li 67p OS=Homo sapiens GN=HEL-S-67p PE=2 SV=1	HEL-S-67p	20 kDa	0.14	0.4	[]	3	2	1	1
Small nuclear ribonucleoprotein 70kDa polypeptide (RNP antigen), isoform CRA_b OS=Homo sapiens GN=SNRP70 PE=4 !	SNRP70	52 kDa	0.0034	0.4	Fibro-BKO low, Fibro-Naive high	3	3	1	1

Glycogen [starch] synthase OS=Homo sapiens PE=2 SV=1		84 kDa	0.46	0.6	[]	1	3	1	1
G protein-coupled receptor 56 isoform 1 OS=Homo sapiens GN=GPR56 PE=2 SV=1	GPR56	78 kDa	0.45	0.7	[]	3	2	2	1
Protein S100 OS=Homo sapiens GN=HEL-S-43 PE=2 SV=1	HEL-S-43	12 kDa	0.23	0.4	[]	4	2	1	1
Ribosomal protein L11, isoform CRA_b OS=Homo sapiens GN=RPL11 PE=2 SV=1	RPL11	20 kDa	0.64	1.6	[]	2	0	1	2
Ribosomal protein L30, isoform CRA_b OS=Homo sapiens GN=RPL30 PE=3 SV=1	RPL30	13 kDa	0.0034	0.4	Fibro-BKO low, Fibro-Naive high	3	3	1	1
Ribosomal protein L35, isoform CRA_a OS=Homo sapiens GN=RPL35 PE=3 SV=1	RPL35	18 kDa	0.55	0.7	[]	2	1	1	1
Growth factor receptor-bound protein 2 OS=Homo sapiens GN=GRB2 PE=2 SV=1	GRB2	25 kDa	0.45	0.5	[]	1	2	1	1
Acid phosphatase 1 isoform c variant (Fragment) OS=Homo sapiens PE=2 SV=1		19 kDa	0.14	0.4	[]	3	2	1	1
Neural cell expressed, developmentally down-regulated 4-like, isoform CRA_b OS=Homo sapiens GN=NEDD4L PE=4 SV=	NEDD4L	96 kDa	0.32	0.3	[]	1	3	0	1
Ubiquitin-activating enzyme E1-like 2, isoform CRA_a OS=Homo sapiens GN=UBE1L2 PE=4 SV=1	UBE1L2	118 kDa	0.45	3	[]	0	2	4	1
DERP12 (Dermal papilla derived protein 12) OS=Homo sapiens GN=derp12 PE=2 SV=1	derp12	38 kDa	0.14	0.2	[]	3	2	1	0
Glutathione S-transferase pi (Fragment) OS=Homo sapiens GN=GSTP1 PE=2 SV=1	GSTP1	17 kDa	0.074	0.4	[]	16	12	5	4
Lin-7 homolog C variant (Fragment) OS=Homo sapiens PE=2 SV=1		22 kDa	0.84	0.8	[]	2	2	0	3
Syndecan OS=Homo sapiens GN=SDC4 PE=2 SV=1	SDC4	14 kDa	0.64	1.6	[]	0	3	2	2
Eukaryotic translation initiation factor 6 (Fragment) OS=Homo sapiens GN=EIF6 PE=1 SV=1	EIF6	12 kDa	0.35	0.4	[]	2	3	0	2
SEC24 related gene family, member C (S. cerevisiae), isoform CRA_a OS=Homo sapiens GN=SEC24C PE=4 SV=1	SEC24C	118 kDa	0.12	0.2	[]	2	3	1	0
Macrophage migration inhibitory factor (Fragment) OS=Homo sapiens GN=MIF PE=1 SV=1	MIF	12 kDa	0.12	0.2	[]	2	3	1	0
Adaptor-related protein complex 3 beta 1 subunit isoform 2 OS=Homo sapiens GN=AP3B1 PE=2 SV=1	AP3B1	96 kDa	0.14	0.3	[]	2	2	1	0
Ubiquitin thioesterase OS=Homo sapiens PE=2 SV=1		35 kDa	0.89	0.8	[]	0	3	2	0
Prosaposin (Variant Gaucher disease and variant metachromatic leukodystrophy), isoform CRA_b OS=Homo sapiens GN	PSAP	58 kDa	0.36	0.3	[]	3	1	1	0
PGRMC1 protein OS=Homo sapiens GN=PGRMC1 PE=2 SV=1	PGRMC1	22 kDa	0.047	0.2	Fibro-BKO low, Fibro-Naive high	3	3	0	1
Trans-Golgi network integral membrane protein 2 OS=Homo sapiens GN=TGOLN2 PE=1 SV=2	TGOLN2	51 kDa	0.15	0.2	[]	2	4	0	1
cDNA FLJ75831, highly similar to Homo sapiens exportin, tRNA (nuclear export receptor for tRNAs) (XPOT), mRNA OS=Homo sapiens PE=2 S		110 kDa	0.047	0.2	Fibro-BKO low, Fibro-Naive high	3	3	0	1
Epididymis secretory protein Li 85 OS=Homo sapiens GN=PCBP1 PE=2 SV=1	PCBP1	37 kDa	0.11	0.1	[]	5	3	0	1
Putative uncharacterized protein (Fragment) OS=Homo sapiens PE=2 SV=1		20 kDa	0.024	0	Fibro-BKO low, Fibro-Naive high	9	7	0	0
Integrin, alpha 5 (Fibronectin receptor, alpha polypeptide), isoform CRA_b OS=Homo sapiens GN=ITGA5 PE=3 SV=1	ITGA5	119 kDa	0.0014	INF	Fibro-BKO high, Fibro-Naive low	0	0	4	4
cDNA FLJ34625 fis, clone KIDNE2015244, highly similar to Serine protease HTRA1 (EC 3.4.21.-) OS=Homo sapiens PE=2 SV=1		48 kDa	0.031	INF	Fibro-BKO high, Fibro-Naive low	0	0	4	3
Complement component 1, q subcomponent, C chain, isoform CRA_a OS=Homo sapiens GN=C1QC PE=4 SV=1	C1QC	26 kDa	0.043	INF	Fibro-BKO high, Fibro-Naive low	0	0	3	5
Ribosomal protein L12 variant (Fragment) OS=Homo sapiens PE=2 SV=1		21 kDa	0.012	0	Fibro-BKO low, Fibro-Naive high	3	4	0	0
Actin-related protein 2 OS=Homo sapiens GN=ACTR2 PE=1 SV=1	ACTR2	6 kDa	0.0011	0	Fibro-BKO low, Fibro-Naive high	4	4	0	0
Cytochrome b5 type B OS=Homo sapiens GN=CYB5B PE=1 SV=1	CYB5B	16 kDa	0.0011	0	Fibro-BKO low, Fibro-Naive high	3	3	0	0
Uncharacterized protein OS=Homo sapiens PE=1 SV=1		63 kDa	0.0011	0	Fibro-BKO low, Fibro-Naive high	2	2	0	0
Cell migration-inducing and hyaluronan-binding protein (Fragment) OS=Homo sapiens GN=CEMIP PE=1 SV=1	CEMIP	17 kDa	0.0014	INF	Fibro-BKO high, Fibro-Naive low	0	0	4	4
Annexin (Fragment) OS=Homo sapiens GN=ANXA6 PE=2 SV=1	ANXA6	76 kDa	0.00011	0.3	Fibro-BKO low, Fibro-Naive high	57	61	16	18
cDNA FLJ78096, highly similar to Homo sapiens actin, alpha, cardiac muscle (ACTC), mRNA OS=Homo sapiens PE=2 SV=1		42 kDa	0.68	1.1	[]	20	16	19	16
HLA class I histocompatibility antigen, A-3 alpha chain OS=Homo sapiens GN=HLA-A PE=1 SV=1	HLA-A	41 kDa	0.72	0.8	[]	13	15	5	16
cDNA, FLJ94136, highly similar to Homo sapiens synaptotagmin binding, cytoplasmic RNA interacting protein (SYNCRIP), mRNA OS=Homo sa		70 kDa	0.083	0.5	[]	2	3	1	1
Amine oxidase OS=Homo sapiens PE=2 SV=1		71 kDa	0.23	1.5	[]	1	2	2	2
cDNA FLJ54369, highly similar to Itchy homolog E3 ubiquitin protein ligase (EC 6.3.2.-) OS=Homo sapiens PE=2 SV=1		93 kDa	0.23	1.5	[]	1	2	2	2
S-adenosylmethionine synthase OS=Homo sapiens PE=2 SV=1		44 kDa	0.0082	0.6	Fibro-BKO low, Fibro-Naive high	2	2	1	1
VAMP3 protein OS=Homo sapiens GN=VAMP3 PE=2 SV=1	VAMP3	11 kDa	0.69	0.9	[]	2	2	2	1
Interferon-induced transmembrane protein 3 OS=Homo sapiens GN=IFITM3 PE=1 SV=1	IFITM3	12 kDa	0.82	1	[]	3	4	3	3
HCG24487, isoform CRA_c OS=Homo sapiens GN=hCG_24487 PE=3 SV=1	hCG_24487	21 kDa	0.31	1.7	[]	1	1	1	2
cDNA, FLJ93141, highly similar to Homo sapiens coagulation factor XIII, A1 polypeptide (F13A1), mRNA OS=Homo sapiens PE=2 SV=1		83 kDa	0.37	1.7	[]	1	1	2	1
Ras-related protein R-Ras2 (Fragment) OS=Homo sapiens GN=RRAS2 PE=1 SV=1	RRAS2	20 kDa	0.22	1.8	[]	2	1	2	3
Receptor-type tyrosine-protein phosphatase OS=Homo sapiens PE=2 SV=1		92 kDa	0.0082	0.6	Fibro-BKO low, Fibro-Naive high	2	2	1	1
Glutamine-fructose-6-phosphate transaminase 2 isoform 2 (Fragment) OS=Homo sapiens GN=GFPT2 PE=2 SV=1	GFPT2	71 kDa	0.083	0.5	[]	2	3	1	1
Chloride intracellular channel protein OS=Homo sapiens PE=2 SV=1		28 kDa	0.69	0.9	[]	2	2	2	1
Formyltetrahydrofolate dehydrogenase isoform a variant (Fragment) OS=Homo sapiens PE=2 SV=1		99 kDa	0.62	1.5	[]	2	1	3	1
cDNA FLJ50778, highly similar to Protein flightless-1 homolog OS=Homo sapiens PE=2 SV=1		141 kDa	0.35	0.4	[]	1	2	1	0
Discoidin domain receptor family, member 1, isoform CRA_b OS=Homo sapiens GN=DDR1 PE=4 SV=1	DDR1	102 kDa	0.37	1.7	[]	1	1	2	1
cDNA FLJ60424, highly similar to Junction plakoglobin OS=Homo sapiens PE=2 SV=1		63 kDa	0.23	1.3	[]	12	13	12	17
Ras-related protein Rab-35 (Fragment) OS=Homo sapiens GN=RAB35 PE=1 SV=1	RAB35	21 kDa	0.58	1.4	[]	1	4	3	3
Misshapen/NIK-related kinase isoform 2 variant (Fragment) OS=Homo sapiens PE=2 SV=1		93 kDa	0.59	1.5	[]	1	2	1	3
60S ribosomal protein L21 OS=Homo sapiens GN=RPL21 PE=1 SV=1	RPL21	10 kDa	0.58	1.8	[]	0	2	2	1
RAB1B protein OS=Homo sapiens GN=RAB1B PE=2 SV=1	RAB1B	22 kDa	0.38	0.5	[]	7	5	1	5
DEAD (Asp-Glu-Ala-Asp) box polypeptide 48, isoform CRA_a OS=Homo sapiens GN=DDX48 PE=3 SV=1	DDX48	47 kDa	0.11	2.6	[]	2	1	4	3
POLR1C protein (Fragment) OS=Homo sapiens GN=POLR1C PE=2 SV=1	POLR1C	38 kDa	0.14	0.4	[]	3	2	1	1
Dynactin subunit 1 OS=Homo sapiens GN=DCTN1 PE=1 SV=1	DCTN1	139 kDa	0.23	1.5	[]	1	2	2	2

EPH receptor B3, isoform CRA_a OS=Homo sapiens GN=EPHB3 PE=3 SV=1	EPHB3	106 kDa	0.92	1	[]	4	6	5	4
Tetraspanin-15 (Fragment) OS=Homo sapiens GN=TSPAN15 PE=1 SV=1	TSPAN15	18 kDa	0.37	1.7	[]	1	1	2	1
Fibronectin (Fragment) OS=Homo sapiens GN=FN1 PE=1 SV=1	FN1	121 kDa	0.0012	52	Fibro-BKO high, Fibro-Naive low	7	14	480	484
Sequestosome-1 OS=Homo sapiens GN=SQSTM1 PE=1 SV=1	SQSTM1	41 kDa	0.49	0.5	[]	2	2	0	2
cDNA FLJ61687, highly similar to Homo sapiens UEV and lactate/malate dehyrogenase domains (UEVLD), transcript variant 1, mRNA OS=Ho		37 kDa	0.58	1.7	[]	0	2	1	2
cDNA FLJ10273 fis, clone HEMBB1001137, highly similar to SEC23-interacting protein OS=Homo sapiens PE=2 SV=1		111 kDa	0.14	0.3	[]	2	2	1	0
NADH-cytochrome b5 reductase OS=Homo sapiens GN=CYB5R3 PE=3 SV=1	CYB5R3	33 kDa	0.32	0.3	[]	1	3	0	1
Receptor protein-tyrosine kinase OS=Homo sapiens GN=ERBB2 PE=1 SV=1	ERBB2	108 kDa	0.033	6.5	Fibro-BKO high, Fibro-Naive low	1	0	3	3
cDNA FLJ56531, highly similar to UV excision repair protein RAD23 homolog B OS=Homo sapiens PE=2 SV=1		42 kDa	0.033	6.5	Fibro-BKO high, Fibro-Naive low	1	0	3	3
cDNA FLJ75154, highly similar to Homo sapiens heterogeneous nuclear ribonucleoprotein C (C1/C2), mRNA OS=Homo sapiens PE=2 SV=1		34 kDa	0.14	0.2	[]	3	2	1	0
M6PR protein OS=Homo sapiens GN=M6PR PE=2 SV=1	M6PR	31 kDa	0.1	0.2	[]	2	3	0	1
Hydroxysteroid (17-beta) dehydrogenase 4, isoform CRA_b (Fragment) OS=Homo sapiens GN=HSD17B4 PE=2 SV=1	HSD17B4	80 kDa	0.19	0.2	[]	4	2	1	0
Extended synaptotagmin-2 OS=Homo sapiens GN=ESYT2 PE=1 SV=1	ESYT2	102 kDa	0.14	0.3	[]	2	2	1	0
cDNA FLJ60782, highly similar to Rho-GTPase-activating protein 1 OS=Homo sapiens PE=2 SV=1		53 kDa	0.64	0.6	[]	3	1	2	0
Epididymis secretory protein Li 10 OS=Homo sapiens GN=HEL-S-10 PE=2 SV=1	HEL-S-10	22 kDa	0.047	0.2	Fibro-BKO low, Fibro-Naive high	3	3	0	1
Ribosomal protein (Fragment) OS=Homo sapiens GN=RPL10A PE=2 SV=1	RPL10A	24 kDa	0.13	0.2	[]	3	2	0	1
Epididymis luminal protein 32 OS=Homo sapiens GN=HEL32 PE=2 SV=1	HEL32	19 kDa	0.047	0.2	Fibro-BKO low, Fibro-Naive high	3	3	0	1
cDNA FLJ51641, highly similar to Nicastrin OS=Homo sapiens PE=2 SV=1		50 kDa	0.71	1.8	[]	1	1	3	0
ATP-dependent RNA helicase DDX1 OS=Homo sapiens GN=DDX1 PE=1 SV=1	DDX1	74 kDa	0.055	0.2	[]	3	3	1	0
Capping protein (Actin filament) muscle Z-line, alpha 2 OS=Homo sapiens GN=CAPZA2 PE=2 SV=1	CAPZA2	33 kDa	0.1	0.2	[]	2	3	0	1
Probable ATP-dependent RNA helicase DDX17 OS=Homo sapiens GN=DDX17 PE=1 SV=1	DDX17	80 kDa	0.15	0.1	[]	6	3	1	0
Talin-2 OS=Homo sapiens GN=TLN2 PE=1 SV=1	TLN2	165 kDa	0.069	4.7	[]	0	1	2	2
Myelin protein zero-like 1, isoform CRA_b OS=Homo sapiens GN=MPZL1 PE=2 SV=1	MPZL1	29 kDa	0.3	4.8	[]	0	1	3	1
Purine nucleoside phosphorylase OS=Homo sapiens PE=2 SV=1		33 kDa	0.36	0.3	[]	3	1	1	0
eIF4G1 protein OS=Homo sapiens GN=EIF4G1 PE=2 SV=1	EIF4G1	176 kDa	0.081	0	[]	1	2	0	0
Testicular tissue protein Li 89 OS=Homo sapiens PE=2 SV=1		25 kDa	0.03	0	Fibro-BKO low, Fibro-Naive high	4	3	0	0
60S ribosomal protein L9 (Fragment) OS=Homo sapiens GN=RPL9 PE=1 SV=8	RPL9	21 kDa	0.012	0	Fibro-BKO low, Fibro-Naive high	3	4	0	0
SET translocation (Myeloid leukemia-associated), isoform CRA_b OS=Homo sapiens GN=SET PE=3 SV=1	SET	32 kDa	0.027	0	Fibro-BKO low, Fibro-Naive high	2	3	0	0
Protein DEK OS=Homo sapiens GN=DEK PE=1 SV=1	DEK	40 kDa	0.03	0	Fibro-BKO low, Fibro-Naive high	4	3	0	0
Gamma-tubulin complex component OS=Homo sapiens PE=2 SV=1		83 kDa	0.0011	0	Fibro-BKO low, Fibro-Naive high	2	2	0	0
Caldesmon 1, isoform CRA_i OS=Homo sapiens GN=CALD1 PE=2 SV=1	CALD1	63 kDa	0.079	INF	[]	0	0	2	4
Translation initiation factor eIF-2B subunit delta OS=Homo sapiens GN=EIF2B4 PE=1 SV=1	EIF2B4	57 kDa	0.11	0	[]	4	2	0	0
Uncharacterized protein OS=Homo sapiens GN=DKFZp686E23209 PE=1 SV=2	DKFZp686E23209	52 kDa	0.026	INF	Fibro-BKO high, Fibro-Naive low	0	0	4	6
Plakophilin-4 OS=Homo sapiens GN=PKP4 PE=1 SV=1	PKP4	134 kDa	0.86	0.7	[]	0	3	0	2
cDNA FLJ56912, highly similar to Fibulin-2 OS=Homo sapiens PE=2 SV=1		129 kDa	0.026	INF	Fibro-BKO high, Fibro-Naive low	0	0	2	3
cDNA FLJ76823, highly similar to Homo sapiens splicing factor, arginine/serine-rich 6 (SFRS6), mRNA OS=Homo sapiens PE=2 SV=1		39 kDa	0.16	0	[]	5	2	0	0
Transmembrane protein 113, isoform CRA_a OS=Homo sapiens GN=TMEM113 PE=4 SV=1	TMEM113	35 kDa	0.05	0	Fibro-BKO low, Fibro-Naive high	3	2	0	0
Putative uncharacterized protein DKFZp686J11235 (Fragment) OS=Homo sapiens GN=DKFZp686J11235 PE=1 SV=1	DKFZp686J11235	54 kDa	0.22	INF	[]	0	0	1	4
Receptor protein-tyrosine kinase (Fragment) OS=Homo sapiens PE=2 SV=1		120 kDa	0.043	INF	Fibro-BKO high, Fibro-Naive low	0	0	3	5
Ngg1 interacting factor 3 like 1 binding protein 1, isoform CRA_a OS=Homo sapiens GN=NIF3L1BP1 PE=4 SV=1	NIF3L1BP1	24 kDa	0.42	0	[]	5	0	0	0
Catenin alpha-2 (Fragment) OS=Homo sapiens GN=CTNNA2 PE=1 SV=1	CTNNA2	13 kDa	0.52	1.9	[]	1	2	1	4
Ribosomal protein S3 variant (Fragment) OS=Homo sapiens PE=2 SV=1		27 kDa	0.4	0.6	[]	4	6	4	1
Proteasome subunit beta type OS=Homo sapiens PE=2 SV=1		30 kDa	0.31	1.7	[]	1	1	1	2
STX12 protein (Fragment) OS=Homo sapiens GN=STX12 PE=2 SV=1	STX12	31 kDa	0.0052	2.3	Fibro-BKO high, Fibro-Naive low	1	1	2	2
Profilin OS=Homo sapiens PE=2 SV=1		21 kDa	0.0082	0.6	Fibro-BKO low, Fibro-Naive high	2	2	1	1
cDNA FLJ56734, moderately similar to Sepiapterin reductase (EC 1.1.1.153) OS=Homo sapiens PE=2 SV=1		13 kDa	0.46	0.6	[]	1	3	1	1
Membrane cofactor protein (Fragment) OS=Homo sapiens GN=CD46 PE=1 SV=1	CD46	9 kDa	0.51	0.8	[]	1	2	1	1
Guanine nucleotide-binding protein subunit gamma (Fragment) OS=Homo sapiens PE=2 SV=1		8 kDa	0.49	0.6	[]	3	1	1	1
Serine/threonine-protein phosphatase OS=Homo sapiens PE=2 SV=1		39 kDa	0.072	0.3	[]	16	13	2	6
Monoglyceride lipase OS=Homo sapiens GN=MGLL PE=1 SV=1	MGLL	34 kDa	0.0082	0.6	Fibro-BKO low, Fibro-Naive high	2	2	1	1
Ribonucleoside-diphosphate reductase OS=Homo sapiens PE=2 SV=1		86 kDa	0.46	0.6	[]	1	3	1	1
Mitogen-activated protein kinase kinase 1 OS=Homo sapiens GN=MAP2K1 PE=2 SV=1	MAP2K1	43 kDa	0.55	0.7	[]	2	1	1	1
Tubulin beta chain OS=Homo sapiens PE=2 SV=1		47 kDa	0.6	1.1	[]	22	15	17	21
40S ribosomal protein S3 OS=Homo sapiens GN=RPS3 PE=1 SV=1	RPS3	17 kDa	0.19	0.4	[]	6	7	4	1
Arginine--tRNA ligase, cytoplasmic OS=Homo sapiens GN=RARS PE=1 SV=1	RARS	14 kDa	0.55	0.7	[]	2	1	1	1
MHC class I antigen (Fragment) OS=Homo sapiens GN=HLA-A PE=3 SV=1	HLA-A	38 kDa	0.63	0.8	[]	9	11	3	12
YBX1 protein (Fragment) OS=Homo sapiens GN=YBX1 PE=2 SV=1	YBX1	29 kDa	0.055	0.4	[]	6	8	3	2
cDNA FLJ50123 OS=Homo sapiens PE=2 SV=1		43 kDa	0.069	4.5	[]	2	2	6	9
N-acetyl-D-glucosamine kinase OS=Homo sapiens GN=NAGK PE=1 SV=3	NAGK	32 kDa	0.33	0.4	[]	2	1	0	1

cDNA FLJ56798, highly similar to Acid sphingomyelinase-like phosphodiesterase 3b (EC 3.1.4.-) OS=Homo sapiens PE=2 SV=1		51 kDa	0.81	0.8	[]	2	1	2	0
Keratin 14 (Epidermolysis bullosa simplex, Dowling-Meara, Koebner), isoform CRA_a OS=Homo sapiens GN=KRT14 PE=3	KRT14	30 kDa	0.76	0.7	[]	2	1	0	2
Cullin 3, isoform CRA_a OS=Homo sapiens GN=CUL3 PE=3 SV=1	CUL3	89 kDa	0.33	0.4	[]	2	1	0	1
GDP-mannose pyrophosphorylase A, isoform CRA_a OS=Homo sapiens GN=GMPPA PE=4 SV=1	GMPPA	46 kDa	0.08	4.4	[]	1	0	2	2
Metastasis associated 1 family, member 2, isoform CRA_a OS=Homo sapiens GN=MTA2 PE=4 SV=1	MTA2	75 kDa	0.26	0.4	[]	3	3	2	0
Hedgehog protein OS=Homo sapiens GN=SHH PE=3 SV=1	SHH	50 kDa	0.28	4.3	[]	1	0	1	3
SMARCD2 protein OS=Homo sapiens GN=SMARCD2 PE=1 SV=1	SMARCD2	56 kDa	0.1	0.2	[]	2	3	0	1
cDNA FLJ55486, highly similar to Semaphorin-3C OS=Homo sapiens PE=2 SV=1		87 kDa	0.12	0.3	[]	2	2	0	1
Rho-associated protein kinase 2 OS=Homo sapiens GN=ROCK2 PE=1 SV=1	ROCK2	133 kDa	0.37	0.4	[]	2	1	1	0
DENN/MADD domain containing 2D, isoform CRA_d OS=Homo sapiens GN=DENND2D PE=4 SV=1	DENND2D	53 kDa	0.31	5.3	[]	1	0	1	4
cDNA FLJ39945 fis, clone SPLEN2023977, highly similar to Homo sapiens CD97 antigen (CD97), transcript variant 2, mRNA OS=Homo sapiens		78 kDa	0.94	1.1	[]	1	2	0	3
Lysine--tRNA ligase (Fragment) OS=Homo sapiens GN=KARS PE=1 SV=1	KARS	29 kDa	0.77	0.7	[]	1	2	0	2
Serine/threonine-protein phosphatase 2A 55 kDa regulatory subunit B OS=Homo sapiens PE=2 SV=1		52 kDa	0.12	0.3	[]	2	2	0	1
Prefoldin subunit 5 (Fragment) OS=Homo sapiens GN=PFDN5 PE=1 SV=1	PFDN5	17 kDa	0.1	0.2	[]	2	3	0	1
Tetraspanin 6, isoform CRA_a OS=Homo sapiens GN=TSPAN6 PE=3 SV=1	TSPAN6	18 kDa	0.3	4.8	[]	0	1	3	1
Testis derived transcript (3 LIM domains) OS=Homo sapiens GN=TES PE=4 SV=1	TES	48 kDa	0.12	0.3	[]	2	2	0	1
MHC class I antigen (Fragment) OS=Homo sapiens GN=HLA-A PE=3 SV=1	HLA-A	32 kDa	0.29	0.4	[]	9	13	0	8
cDNA FLJ55697, highly similar to Protein transport protein Sec23B OS=Homo sapiens PE=2 SV=1		84 kDa	0.1	0.2	[]	2	3	0	1
Rho guanine nucleotide exchange factor (GEF) 1, isoform CRA_e OS=Homo sapiens GN=ARHGEF1 PE=4 SV=1	ARHGEF1	102 kDa	0.12	0.3	[]	2	2	0	1
Protein phosphatase 1, regulatory subunit 7, isoform CRA_b OS=Homo sapiens GN=PPP1R7 PE=2 SV=1	PPP1R7	42 kDa	0.12	0.3	[]	2	2	0	1
Fructosamine-3-kinase-related protein, isoform CRA_a OS=Homo sapiens GN=FN3KRP PE=2 SV=1	FN3KRP	34 kDa	0.0011	0	Fibro-BKO low, Fibro-Naive high	3	3	0	0
Epididymis secretory protein Li 6 OS=Homo sapiens GN=HEL-S-165mP PE=2 SV=1	HEL-S-165mP	37 kDa	0.0011	0	Fibro-BKO low, Fibro-Naive high	3	3	0	0
cDNA FLJ55495, highly similar to Ethanolamine-phosphate cytidylyltransferase (EC 2.7.7.14) OS=Homo sapiens PE=2 SV=1		41 kDa	0.05	0	Fibro-BKO low, Fibro-Naive high	3	2	0	0
Regulator of chromosome condensation 2, isoform CRA_a OS=Homo sapiens GN=RCC2 PE=4 SV=1	RCC2	56 kDa	0.027	0	Fibro-BKO low, Fibro-Naive high	2	3	0	0
Dimethylarginine dimethylaminohydrolase 2, isoform CRA_a OS=Homo sapiens GN=HEL-S-277 PE=1 SV=1	HEL-S-277	30 kDa	0.0011	0	Fibro-BKO low, Fibro-Naive high	3	3	0	0
cDNA FLJ13801 fis, clone THYRO1000173, highly similar to AP-1 complex subunit mu-2 OS=Homo sapiens PE=2 SV=1		48 kDa	0.0011	0	Fibro-BKO low, Fibro-Naive high	2	2	0	0
Heterogeneous nuclear ribonucleoprotein A2/B1, isoform CRA_d OS=Homo sapiens GN=HNRPA2B1 PE=4 SV=1	HNRPA2B1	31 kDa	0.11	0	[]	4	2	0	0
Deoxyribonuclease-1-like 1 (Fragment) OS=Homo sapiens GN=DNASE1L1 PE=1 SV=1	DNASE1L1	24 kDa	0.11	0	[]	4	2	0	0
L-xylulose reductase (Fragment) OS=Homo sapiens GN=DCXR PE=1 SV=8	DCXR	24 kDa	0.05	0	Fibro-BKO low, Fibro-Naive high	3	2	0	0
EMILIN-1 OS=Homo sapiens GN=EMILIN1 PE=1 SV=1	EMILIN1	107 kDa	0.026	INF	Fibro-BKO high, Fibro-Naive low	0	0	2	3
Citrate synthase OS=Homo sapiens GN=CS PE=3 SV=1	CS	52 kDa	0.0011	0	Fibro-BKO low, Fibro-Naive high	2	2	0	0
Prelamin-A/C (Fragment) OS=Homo sapiens GN=LMNA PE=1 SV=1	LMNA	28 kDa	0.05	0	Fibro-BKO low, Fibro-Naive high	3	2	0	0
Ribosomal protein, large, P1, isoform CRA_a OS=Homo sapiens GN=RPLP1 PE=3 SV=1	RPLP1	12 kDa	0.11	0	[]	4	2	0	0
Eukaryotic translation initiation factor 4E OS=Homo sapiens GN=EIF4E PE=1 SV=1	EIF4E	29 kDa	0.0011	0	Fibro-BKO low, Fibro-Naive high	3	3	0	0
HCG2016482, isoform CRA_b OS=Homo sapiens GN=hCG_2016482 PE=4 SV=1	hCG_2016482	23 kDa	0.027	0	Fibro-BKO low, Fibro-Naive high	2	3	0	0
Testis tissue sperm-binding protein Li 45a OS=Homo sapiens PE=2 SV=1		36 kDa	0.081	0	[]	1	2	0	0
Amidophosphoribosyltransferase OS=Homo sapiens GN=PPAT PE=2 SV=1	PPAT	57 kDa	0.05	0	Fibro-BKO low, Fibro-Naive high	3	2	0	0
Peroxiredoxin 3 isoform a variant (Fragment) OS=Homo sapiens PE=2 SV=1		28 kDa	0.25	0	[]	4	1	0	0
cDNA FLJ61699, highly similar to Homo sapiens androgen-induced proliferation inhibitor (APRIN), transcript variant 1, mRNA OS=Homo sapi		149 kDa	0.13	0	[]	2	5	0	0
Lipopolysaccharide-responsive and beige-like anchor protein OS=Homo sapiens GN=LRBA PE=1 SV=1	LRBA	287 kDa	0.23	0	[]	1	4	0	0
sp|CO5_HUMAN|		188 kDa	0.25	INF	[]	0	0	4	1
Translin OS=Homo sapiens GN=TSN PE=1 SV=1	TSN	26 kDa	0.2	0	[]	3	1	0	0
cDNA, FLJ96114, highly similar to Homo sapiens bromodomain and WD repeat domain containing 2 (BRWD2), mRNA OS=Homo sapiens PE=		137 kDa	0.17	0	[]	1	3	0	0
Thy-1 cell surface antigen, isoform CRA_a OS=Homo sapiens GN=THY1 PE=2 SV=1	THY1	18 kDa	0.051	INF	[]	0	0	3	2
Queuine tRNA-ribosyltransferase catalytic subunit 1 OS=Homo sapiens GN=QTRT1 PE=2 SV=1	QTRT1	44 kDa	0.2	0	[]	3	1	0	0
Polypeptide N-acetylgalactosaminyltransferase OS=Homo sapiens PE=2 SV=1		73 kDa	0.05	0	Fibro-BKO low, Fibro-Naive high	3	2	0	0
cDNA FLJ75774, highly similar to Homo sapiens lectin, mannose-binding 2 (LMAN2), mRNA OS=Homo sapiens PE=2 SV=1		40 kDa	0.42	0	[]	4	0	0	0
Transportin 1, isoform CRA_a OS=Homo sapiens GN=TNPO1 PE=4 SV=1	TNPO1	102 kDa	0.25	0	[]	4	1	0	0
Collagen alpha-2(VI) chain (Fragment) OS=Homo sapiens GN=COL6A2 PE=1 SV=1	COL6A2	26 kDa	0.051	INF	[]	0	0	3	2
Structure specific recognition protein 1, isoform CRA_a OS=Homo sapiens GN=SSRP1 PE=4 SV=1	SSRP1	81 kDa	0.25	0	[]	4	1	0	0
Epididymis secretory sperm binding protein Li 134P OS=Homo sapiens GN=HEL-S-134P PE=2 SV=1	HEL-S-134P	52 kDa	0.42	0	[]	0	3	0	0
Tubulin beta chain (Fragment) OS=Homo sapiens PE=2 SV=1		50 kDa	0.19	0.8	[]	38	35	21	29
Ferritin (Fragment) OS=Homo sapiens GN=FTL PE=3 SV=1	FTL	10 kDa	0.025	2.8	Fibro-BKO high, Fibro-Naive low	3	3	8	7
Chromosome 6 open reading frame 55, isoform CRA_b OS=Homo sapiens GN=VTA1 PE=1 SV=1	VTA1	31 kDa	0.26	0.6	[]	5	3	2	2
cDNA FLJ53758 OS=Homo sapiens PE=2 SV=1		38 kDa	0.068	4.1	[]	3	1	6	8
Myosin-14 OS=Homo sapiens GN=MYH14 PE=1 SV=1	MYH14	58 kDa	0.88	0.9	[]	2	4	2	3
Proteasome subunit alpha type OS=Homo sapiens PE=2 SV=1		18 kDa	0.26	2	[]	3	2	3	6
Vacuolar protein sorting 35 variant (Fragment) OS=Homo sapiens PE=2 SV=1		92 kDa	0.11	0.4	[]	7	11	4	3
Annexin (Fragment) OS=Homo sapiens GN=ANXA3 PE=1 SV=1	ANXA3	17 kDa	0.23	0.6	[]	5	7	4	2

Tubulin beta chain OS=Homo sapiens PE=2 SV=1		50 kDa	0.35	0.8	[]	74	63	42	58
Tubulin beta chain OS=Homo sapiens PE=2 SV=1		46 kDa	0.86	1	[]	53	40	35	45
Cysteine-rich protein 2 (Fragment) OS=Homo sapiens GN=CRIP2 PE=1 SV=1	CRIP2	21 kDa	0.51	0.8	[]	1	2	1	1
Histone-binding protein RBBP7 OS=Homo sapiens GN=RBBP7 PE=1 SV=1	RBBP7	47 kDa	0.51	0.8	[]	1	2	1	1
DYNC1H1 protein OS=Homo sapiens GN=DYNC1H1 PE=2 SV=1	DYNC1H1	22 kDa	0.37	1.7	[]	1	1	2	1
Cortactin, isoform CRA_c OS=Homo sapiens GN=CTTN PE=4 SV=1	CTTN	57 kDa	0.55	0.7	[]	2	1	1	1
6-phosphogluconolactonase, isoform CRA_b OS=Homo sapiens GN=HEL-S-304 PE=2 SV=1	HEL-S-304	28 kDa	0.55	0.7	[]	2	1	1	1
CMP-N-acetylneuraminate-beta-galactosamide-alpha-2,3-sialyltransferase 4 (Fragment) OS=Homo sapiens GN=ST3GAL4	ST3GAL4	24 kDa	0.55	0.7	[]	2	1	1	1
Peptidylprolyl isomerase OS=Homo sapiens PE=2 SV=1		52 kDa	0.55	0.7	[]	2	1	1	1
FERM, RhoGEF and pleckstrin domain-containing protein 1 OS=Homo sapiens GN=FARP1 PE=1 SV=2	FARP1	122 kDa	0.078	3	[]	1	4	6	7
Keratin, type II cytoskeletal 8 (Fragment) OS=Homo sapiens GN=KRT8 PE=1 SV=1	KRT8	31 kDa	0.096	0.3	[]	11	8	2	4
Heterogeneous nuclear ribonucleoprotein F, isoform CRA_a OS=Homo sapiens GN=HNRPF PE=4 SV=1	HNRPF	46 kDa	0.14	0.8	[]	5	5	3	4
ADP-ribosylation factor 4 (Fragment) OS=Homo sapiens GN=ARF4 PE=1 SV=1	ARF4	15 kDa	0.096	0.3	[]	10	7	3	2
cDNA FLJ54827, highly similar to Alpha-parvin OS=Homo sapiens PE=2 SV=1		33 kDa	0.51	0.8	[]	1	2	1	1
Receptor protein-tyrosine kinase OS=Homo sapiens PE=2 SV=1		134 kDa	0.026	3.4	Fibro-BKO high, Fibro-Naive low	1	2	4	5
Calcium-transporting ATPase OS=Homo sapiens GN=ATP2B4 PE=3 SV=1	ATP2B4	134 kDa	0.48	1.7	[]	1	5	3	6
cDNA FLJ51656, highly similar to Actin-like protein 2 OS=Homo sapiens PE=2 SV=1		39 kDa	0.12	0.3	[]	4	4	0	2
Phosphoinositide phospholipase C OS=Homo sapiens GN=PLCD3 PE=4 SV=1	PLCD3	60 kDa	0.08	4.4	[]	1	0	2	2
Transmembrane channel-like protein OS=Homo sapiens PE=2 SV=1		87 kDa	0.58	1.7	[]	0	2	1	2
CDW4/GRWD1 OS=Homo sapiens GN=GRWD1 PE=2 SV=1	GRWD1	49 kDa	0.14	0.3	[]	2	2	1	0
Protein kinase C and casein kinase substrate in neurons 2, isoform CRA_a OS=Homo sapiens GN=PACSIN2 PE=4 SV=1	PACSIN2	50 kDa	0.33	0.4	[]	2	1	0	1
Glyceraldehyde-3-phosphate dehydrogenase OS=Homo sapiens GN=HEL-S-278 PE=2 SV=1	HEL-S-278	45 kDa	0.12	0.3	[]	2	2	0	1
cDNA, FLJ93029, highly similar to Homo sapiens COP9 constitutive photomorphogenic homolog subunit 2 (Arabidopsis) (COPS2), mRNA OS=		52 kDa	0.33	0.4	[]	2	1	0	1
Signal recognition particle 14 kDa protein OS=Homo sapiens GN=SRP14 PE=1 SV=1	SRP14	13 kDa	0.35	0.4	[]	1	2	1	0
Epithelial protein lost in neoplasm beta variant (Fragment) OS=Homo sapiens PE=2 SV=1		85 kDa	0.34	0.3	[]	1	3	1	0
Protein Wnt OS=Homo sapiens PE=2 SV=1		39 kDa	0.069	4.7	[]	0	1	2	2
SRP72 protein (Fragment) OS=Homo sapiens GN=SRP72 PE=2 SV=1	SRP72	63 kDa	0.14	0.3	[]	2	2	1	0
Testicular tissue protein Li 75 OS=Homo sapiens PE=2 SV=1		37 kDa	0.34	0.3	[]	3	1	0	1
Syntaxin 4A (Placental), isoform CRA_a OS=Homo sapiens GN=STX4A PE=2 SV=1	STX4A	25 kDa	0.34	0.3	[]	1	3	1	0
cDNA FLJ57995, moderately similar to Ubiquitin-conjugating enzyme E2-25 kDa (EC 6.3.2.19) OS=Homo sapiens PE=2 SV=1		16 kDa	0.14	0.3	[]	2	2	1	0
Testis tissue sperm-binding protein Li 66n OS=Homo sapiens PE=2 SV=1		71 kDa	0.31	0.4	[]	1	2	0	1
Sodium/potassium-transporting ATPase subunit alpha (Fragment) OS=Homo sapiens GN=ATP1A2 PE=2 SV=1	ATP1A2	112 kDa	0.78	1	[]	18	22	18	16
HCG1640809, isoform CRA_b OS=Homo sapiens GN=hCG_1640809 PE=4 SV=1	hCG_1640809	35 kDa	0.27	4.6	[]	0	1	1	3
cDNA FLJ77640, highly similar to Homo sapiens copine II (CPNE2), mRNA OS=Homo sapiens PE=2 SV=1		61 kDa	0.34	0.3	[]	1	3	1	0
Beta-hexosaminidase OS=Homo sapiens GN=HEXB PE=3 SV=1	HEXB	63 kDa	0.31	0.4	[]	1	2	0	1
cDNA FLJ75699, highly similar to Homo sapiens osteoclast stimulating factor 1 (OSTF1), mRNA OS=Homo sapiens PE=2 SV=1		24 kDa	0.31	0.4	[]	1	2	0	1
UPF1 regulator of nonsense transcripts homolog (Yeast), isoform CRA_b OS=Homo sapiens GN=UPF1 PE=4 SV=1	UPF1	123 kDa	0.66	2.2	[]	0	1	0	2
AP3D1 protein (Fragment) OS=Homo sapiens GN=AP3D1 PE=2 SV=1	AP3D1	99 kDa	0.62	0.4	[]	0	3	0	1
Serpin peptidase inhibitor, clade H (Heat shock protein 47), member 1, (Collagen binding protein 1), isoform CRA_a OS=	SERPINH1	46 kDa	0.11	0	[]	2	1	0	0
eIF2C2 protein (Fragment) OS=Homo sapiens GN=EIF2C2 PE=2 SV=1	EIF2C2	91 kDa	0.0011	0	Fibro-BKO low, Fibro-Naive high	2	2	0	0
U2 small nuclear RNA auxiliary factor 2 isoform b OS=Homo sapiens GN=U2AF2 PE=2 SV=1	U2AF2	53 kDa	0.35	0.4	[]	1	2	1	0
Prefoldin subunit 2 OS=Homo sapiens GN=PFDN2 PE=2 SV=1	PFDN2	17 kDa	0.0011	0	Fibro-BKO low, Fibro-Naive high	2	2	0	0
Plastin-3 OS=Homo sapiens GN=PLS3 PE=1 SV=1	PLS3	69 kDa	0.081	0	[]	1	2	0	0
Beta-galactosidase OS=Homo sapiens GN=LOC89944 PE=3 SV=1	LOC89944	72 kDa	0.05	0	Fibro-BKO low, Fibro-Naive high	3	2	0	0
Inter-alpha-trypsin inhibitor heavy chain H3 OS=Homo sapiens GN=ITIH3 PE=1 SV=1	ITIH3	75 kDa	0.026	INF	Fibro-BKO high, Fibro-Naive low	0	0	2	3
Single-stranded DNA-binding protein, mitochondrial (Fragment) OS=Homo sapiens GN=SSBP1 PE=1 SV=1	SSBP1	16 kDa	0.05	0	Fibro-BKO low, Fibro-Naive high	3	2	0	0
DNA helicase (Fragment) OS=Homo sapiens GN=MCM7 PE=2 SV=1	MCM7	81 kDa	0.05	0	Fibro-BKO low, Fibro-Naive high	3	2	0	0
Nuclear protein localization 4 homolog (S. cerevisiae), isoform CRA_a OS=Homo sapiens GN=NPLOC4 PE=4 SV=1	NPLOC4	68 kDa	0.0011	0	Fibro-BKO low, Fibro-Naive high	2	2	0	0
Lysyl oxidase-like 2 protein isoform b (Fragment) OS=Homo sapiens GN=LOXL2 PE=2 SV=1	LOXL2	84 kDa	0.0091	INF	Fibro-BKO high, Fibro-Naive low	0	0	18	16
cDNA FLJ54595, highly similar to Golgi reassembly-stacking protein 2 OS=Homo sapiens PE=2 SV=1		42 kDa	0.027	0	Fibro-BKO low, Fibro-Naive high	2	2	0	0
DNA topoisomerase 2 (Fragment) OS=Homo sapiens PE=2 SV=1		134 kDa	0.0011	0	Fibro-BKO low, Fibro-Naive high	2	2	0	0
NADPH--cytochrome P450 reductase OS=Homo sapiens GN=POR PE=2 SV=1	POR	77 kDa	0.0011	0	Fibro-BKO low, Fibro-Naive high	2	2	0	0
MAM domain-containing protein 2 (Fragment) OS=Homo sapiens GN=MAMDC2 PE=1 SV=1	MAMDC2	62 kDa	0.051	INF	[]	0	0	3	2
Eukaryotic translation initiation factor 3 subunit I OS=Homo sapiens GN=EIF3S2 PE=2 SV=1	EIF3S2	37 kDa	0.0011	0	Fibro-BKO low, Fibro-Naive high	2	2	0	0
cDNA, FLJ96222, highly similar to Homo sapiens betaine-homocysteine methyltransferase (BHMT), mRNA OS=Homo sapiens PE=2 SV=1		45 kDa	0.051	INF	[]	0	0	3	2
Metastasis associated 1, isoform CRA_c OS=Homo sapiens GN=MTA1 PE=4 SV=1	MTA1	71 kDa	0.0011	0	Fibro-BKO low, Fibro-Naive high	3	3	0	0
Inositol monophosphatase domain containing 1, isoform CRA_a OS=Homo sapiens GN=IMPAD1 PE=4 SV=1	IMPAD1	39 kDa	0.05	0	Fibro-BKO low, Fibro-Naive high	3	2	0	0
Tyrosine-protein kinase receptor OS=Homo sapiens PE=2 SV=1		146 kDa	0.05	0	Fibro-BKO low, Fibro-Naive high	3	2	0	0
Fascin OS=Homo sapiens PE=2 SV=1		52 kDa	0.0014	INF	Fibro-BKO high, Fibro-Naive low	0	0	2	2

Procollagen-lysine 1, 2-oxoglutarate 5-dioxygenase 1, isoform CRA_a OS=Homo sapiens GN=PLOD1 PE=4 SV=1	PLOD1	84 kDa	0.57	3.4	[]	1	0	3	0
Cysteinyl-tRNA synthetase, isoform CRA_a OS=Homo sapiens GN=CARS PE=3 SV=1	CARS	85 kDa	0.17	0	[]	1	3	0	0
Cell division cycle 2, G1 to S and G2 to M, isoform CRA_c OS=Homo sapiens GN=CDC2 PE=4 SV=1	CDC2	28 kDa	0.2	0	[]	3	1	0	0
cDNA FLJ36374 fis, clone THYMU2008185, highly similar to Xaa-Pro aminopeptidase 1 (EC 3.4.11.9) OS=Homo sapiens PE=2 SV=1		62 kDa	0.11	0	[]	2	1	0	0
Replication protein A 70 kDa DNA-binding subunit (Fragment) OS=Homo sapiens GN=RPA1 PE=1 SV=1	RPA1	35 kDa	0.0011	0	Fibro-BKO low, Fibro-Naive high	2	2	0	0
Malate dehydrogenase OS=Homo sapiens GN=MDH2 PE=2 SV=1	MDH2	36 kDa	0.2	0	[]	3	1	0	0
CDW11/WDR57 OS=Homo sapiens GN=WDR57 PE=2 SV=1	WDR57	39 kDa	0.081	0	[]	1	2	0	0
HCG2011153, isoform CRA_b OS=Homo sapiens GN=hCG_2011153 PE=4 SV=1	hCG_2011153	79 kDa	0.17	0	[]	1	3	0	0
Guanine deaminase, isoform CRA_b OS=Homo sapiens GN=GDA PE=4 SV=1	GDA	51 kDa	0.027	0	Fibro-BKO low, Fibro-Naive high	2	3	0	0
Nudix (Nucleoside diphosphate linked moiety X)-type motif 21, isoform CRA_a OS=Homo sapiens GN=NUDT21 PE=4 SV=	NUDT21	26 kDa	0.027	0	Fibro-BKO low, Fibro-Naive high	2	3	0	0
AP-2 complex subunit mu OS=Homo sapiens GN=AP2M1 PE=1 SV=1	AP2M1	50 kDa	0.74	0.6	[]	2	0	1	0
Myosin-reactive immunoglobulin light chain variable region (Fragment) OS=Homo sapiens PE=2 SV=1		12 kDa	0.17	INF	[]	0	0	1	3
HCG2005638, isoform CRA_a OS=Homo sapiens GN=hCG_2005638 PE=4 SV=1	hCG_2005638	49 kDa	0.17	0	[]	1	3	0	0
SEC16A protein (Fragment) OS=Homo sapiens GN=SEC16A PE=2 SV=1	SEC16A	211 kDa	0.25	0	[]	4	1	0	0
cDNA FLJ13106 fis, clone NT2RP3002455, highly similar to DnaJ homolog subfamily C member 13 OS=Homo sapiens PE=2 SV=1		99 kDa	0.42	0	[]	0	4	0	0
MHC class I antigen (Fragment) OS=Homo sapiens GN=HLA-A PE=3 SV=1	HLA-A	38 kDa	0.51	0.7	[]	12	17	4	14
Putative uncharacterized protein DKFZp686O2462 (Fragment) OS=Homo sapiens GN=DKFZp686O2462 PE=4 SV=1	DKFZp686O2462	72 kDa	0.084	0.5	[]	7	10	4	4
Nuclease-sensitive element-binding protein 1 (Fragment) OS=Homo sapiens GN=YBX1 PE=1 SV=1	YBX1	42 kDa	0.025	0.6	Fibro-BKO low, Fibro-Naive high	5	6	3	3
14-3-3 protein theta (Fragment) OS=Homo sapiens GN=YWHAQ PE=1 SV=1	YWHAQ	17 kDa	0.042	0.4	Fibro-BKO low, Fibro-Naive high	6	5	2	2
FERM, RhoGEF and pleckstrin domain-containing protein 1 (Fragment) OS=Homo sapiens GN=FARP1 PE=1 SV=1	FARP1	28 kDa	0.11	2.8	[]	1	1	3	2
MIT domain-containing protein 1 (Fragment) OS=Homo sapiens GN=MITD1 PE=1 SV=1	MITD1	23 kDa	0.69	0.9	[]	2	2	2	1
MIT domain-containing protein 1 (Fragment) OS=Homo sapiens GN=MITD1 PE=1 SV=1	MITD1	28 kDa	0.69	0.9	[]	2	2	2	1
Ferritin OS=Homo sapiens GN=FTL PE=1 SV=1	FTL	21 kDa	0.052	2.1	[]	3	4	7	6
Cytoplasmic FMR1-interacting protein 1 (Fragment) OS=Homo sapiens GN=CYFIP1 PE=1 SV=1	CYFIP1	21 kDa	0.14	1.1	[]	1	1	1	1
cDNA FLJ56447, highly similar to Dynamin-2 (EC 3.6.5.5) OS=Homo sapiens PE=2 SV=1		68 kDa	0.51	0.8	[]	1	2	1	1
Annexin (Fragment) OS=Homo sapiens PE=2 SV=1		35 kDa	0.071	1.7	[]	6	5	9	8
cDNA FLJ57899, highly similar to Mitotic checkpoint protein BUB3 OS=Homo sapiens PE=2 SV=1		28 kDa	0.075	0.5	[]	5	4	2	2
Testicular tissue protein Li 27 OS=Homo sapiens PE=2 SV=1		37 kDa	0.075	0.5	[]	5	4	2	2
Actin-like protein (Fragment) OS=Homo sapiens GN=ACT PE=4 SV=1	ACT	12 kDa	0.066	1.4	[]	12	12	13	16
MHC class I antigen (Fragment) OS=Homo sapiens GN=HLA-C PE=3 SV=1	HLA-C	32 kDa	0.14	0.6	[]	12	13	5	9
Deoxyribose-phosphate aldolase OS=Homo sapiens GN=DERA PE=1 SV=1	DERA	31 kDa	0.044	0.2	Fibro-BKO low, Fibro-Naive high	4	6	1	1
Protein SEC13 homolog OS=Homo sapiens GN=SEC13 PE=1 SV=1	SEC13	36 kDa	0.18	0.4	[]	2	4	1	1
RAB6A, member RAS oncogene family, isoform CRA_b OS=Homo sapiens GN=RAB6A PE=4 SV=1	RAB6A	24 kDa	0.14	0.2	[]	3	2	1	0
Calcium/calmodulin-dependent serine protein kinase (MAGUK family) OS=Homo sapiens GN=CASK PE=2 SV=1	CASK	102 kDa	0.12	6.9	[]	0	1	2	4
Flap endonuclease 1 OS=Homo sapiens GN=FEN1 PE=2 SV=1	FEN1	39 kDa	0.35	0.4	[]	1	2	1	0
Cytochrome b-5 isoform 1 variant (Fragment) OS=Homo sapiens PE=2 SV=1		15 kDa	0.37	0.4	[]	2	1	1	0
Chromosome 1 open reading frame 24, isoform CRA_a OS=Homo sapiens GN=C1orf24 PE=4 SV=1	C1orf24	103 kDa	0.31	0.4	[]	1	2	0	1
Serine/threonine-protein kinase 24 OS=Homo sapiens GN=STK24 PE=1 SV=1	STK24	46 kDa	0.84	0.8	[]	0	3	1	1
cDNA, FLJ96158, highly similar to Homo sapiens calpain 2, (m/II) large subunit (CAPN2), mRNA OS=Homo sapiens PE=2 SV=1		80 kDa	0.94	1.1	[]	2	0	1	1
Renin receptor (Fragment) OS=Homo sapiens GN=ATP6AP2 PE=1 SV=1	ATP6AP2	33 kDa	0.94	1.1	[]	2	0	1	1
Argininosuccinate lyase isoform 1 OS=Homo sapiens GN=ASL PE=2 SV=1	ASL	52 kDa	0.33	0.4	[]	2	1	0	1
Negative elongation factor B OS=Homo sapiens GN=NELFB PE=1 SV=1	NELFB	70 kDa	0.31	0.4	[]	1	2	0	1
Complement component 9, isoform CRA_a OS=Homo sapiens GN=C9 PE=4 SV=1	C9	63 kDa	0.25	3.2	[]	1	0	1	2
Importin-7 (Fragment) OS=Homo sapiens GN=IPO7 PE=1 SV=8	IPO7	15 kDa	0.26	3.5	[]	0	1	2	1
cDNA FLJ60713, highly similar to Homo sapiens heterogeneous nuclear ribonucleoprotein A/B (HNRPAB), transcript variant 1, mRNA OS=Ho		35 kDa	0.33	0.4	[]	2	1	0	1
BM-010 OS=Homo sapiens GN=EIF4A2 PE=2 SV=1	EIF4A2	36 kDa	0.81	0.8	[]	2	1	2	0
Neural cell expressed, developmentally down-regulated 4, isoform CRA_b OS=Homo sapiens GN=NEDD4 PE=4 SV=1	NEDD4	104 kDa	0.94	1.1	[]	1	1	0	2
Pyrroline-5-carboxylate reductase OS=Homo sapiens GN=PYCRL PE=1 SV=1	PYCRL	30 kDa	0.33	0.4	[]	2	1	0	1
40S ribosomal protein S11 OS=Homo sapiens GN=RPS11 PE=1 SV=1	RPS11	14 kDa	0.33	0.4	[]	2	1	0	1
RuvB-like helicase OS=Homo sapiens PE=2 SV=1		27 kDa	0.11	0	[]	4	2	0	0
cDNA FLJ55860, highly similar to Homo sapiens growth hormone regulated TBC protein 1 (GRTP1), mRNA OS=Homo sapiens PE=2 SV=1		39 kDa	0.25	3.2	[]	1	0	1	2
Exportin-1 (Fragment) OS=Homo sapiens GN=XPO1 PE=1 SV=1	XPO1	11 kDa	0.26	3.5	[]	0	1	2	1
Matrix metalloproteinase 7 preproprotein variant (Fragment) OS=Homo sapiens PE=2 SV=1		30 kDa	0.11	INF	[]	0	0	2	1
WD repeat-containing protein 36 OS=Homo sapiens GN=WDR36 PE=1 SV=1	WDR36	99 kDa	0.11	0	[]	2	1	0	0
COL6A3 protein OS=Homo sapiens GN=COL6A3 PE=2 SV=1	COL6A3	278 kDa	0.0003	84	Fibro-BKO high, Fibro-Naive low	0	5	175	184
Urokinase plasminogen activator surface receptor OS=Homo sapiens GN=PLAUR PE=1 SV=1	PLAUR	32 kDa	0.2	3.9	[]	1	1	2	5
Anion exchange protein OS=Homo sapiens GN=SLC4A7 PE=1 SV=1	SLC4A7	135 kDa	0.64	2.4	[]	0	1	2	0
Ig mu chain C region (Fragment) OS=Homo sapiens GN=IGHM PE=1 SV=2	IGHM	49 kDa	0.033	INF	Fibro-BKO high, Fibro-Naive low	0	0	14	22
Testis tissue sperm-binding protein Li 70n OS=Homo sapiens PE=2 SV=1		46 kDa	0.0014	INF	Fibro-BKO high, Fibro-Naive low	0	0	2	2

Eukaryotic translation initiation factor 3 subunit K OS=Homo sapiens GN=EIF3K PE=2 SV=1	EIF3K	25 kDa	0.0011	0	Fibro-BKO low, Fibro-Naive high	2	2	0	0
Adenylate kinase 2, mitochondrial OS=Homo sapiens GN=AK2 PE=2 SV=1	AK2	26 kDa	0.0011	0	Fibro-BKO low, Fibro-Naive high	2	2	0	0
Transmembrane 9 superfamily member OS=Homo sapiens PE=2 SV=1		68 kDa	0.0011	0	Fibro-BKO low, Fibro-Naive high	2	2	0	0
cDNA, FLJ95176, Homo sapiens CGI-48 protein (CGI-48), mRNA OS=Homo sapiens PE=2 SV=1		58 kDa	0.0011	0	Fibro-BKO low, Fibro-Naive high	2	2	0	0
Serine/arginine-rich-splicing factor 7 OS=Homo sapiens GN=SRSF7 PE=1 SV=1	SRSF7	16 kDa	0.05	0	Fibro-BKO low, Fibro-Naive high	3	2	0	0
D-dopachrome tautomerase OS=Homo sapiens GN=DDT PE=2 SV=1	DDT	13 kDa	0.0011	0	Fibro-BKO low, Fibro-Naive high	2	2	0	0
Integrin alpha 4 variant (Fragment) OS=Homo sapiens PE=2 SV=1		95 kDa	0.011	INF	Fibro-BKO high, Fibro-Naive low	0	0	3	4
NAD(P)H-hydrate epimerase OS=Homo sapiens GN=APOA1BP PE=2 SV=1	APOA1BP	34 kDa	0.0011	0	Fibro-BKO low, Fibro-Naive high	2	2	0	0
cDNA FLJ75700, highly similar to Homo sapiens complement component 1, q subcomponent binding protein (C1QBP), nuclear gene encodir		31 kDa	0.11	0	[]	2	1	0	0
cDNA FLJ55318, highly similar to Casein kinase I isoform alpha (EC 2.7.11.1) OS=Homo sapiens PE=2 SV=1		27 kDa	0.0011	0	Fibro-BKO low, Fibro-Naive high	2	2	0	0
Constitutive coactivator of PPAR-gamma-like protein 1 (Fragment) OS=Homo sapiens GN=FAM120A PE=1 SV=1	FAM120A	54 kDa	0.081	0	[]	1	2	0	0
Laminin subunit alpha-2 OS=Homo sapiens GN=LAMA2 PE=1 SV=1	LAMA2	344 kDa	0.079	INF	[]	0	0	1	2
Gamma-tubulin complex component OS=Homo sapiens PE=2 SV=1		103 kDa	0.081	0	[]	1	2	0	0
Serine/threonine-protein phosphatase OS=Homo sapiens GN=PPP5C PE=3 SV=1	PPP5C	57 kDa	0.11	0	[]	2	1	0	0
Protein disulfide-isomerase OS=Homo sapiens GN=P4HB PE=2 SV=1	P4HB	57 kDa	0.74	0.6	[]	0	2	0	1
RecQ protein-like (DNA helicase Q1-like), isoform CRA_a OS=Homo sapiens GN=RECQL PE=4 SV=1	RECQL	73 kDa	0.081	0	[]	1	2	0	0
cDNA FLJ77162, highly similar to Homo sapiens SMC1 structural maintenance of chromosomes 1-like 1 (SMC1L1), mRNA (Fragment) OS=Ho		99 kDa	0.11	0	[]	2	1	0	0
ATPase, H+ transporting, lysosomal 50/57kDa, V1 subunit H, isoform CRA_a OS=Homo sapiens GN=ATP6V1H PE=4 SV=1	ATP6V1H	56 kDa	0.17	0	[]	1	3	0	0
Serine hydroxymethyltransferase OS=Homo sapiens GN=SHMT1 PE=3 SV=1	SHMT1	49 kDa	0.17	0	[]	1	3	0	0
N-acylaminoacyl-peptide hydrolase, isoform CRA_b OS=Homo sapiens GN=APEH PE=4 SV=1	APEH	81 kDa	0.17	0	[]	1	3	0	0
Serine protease HTRA2, mitochondrial (Fragment) OS=Homo sapiens GN=HTRA2 PE=1 SV=1	HTRA2	45 kDa	0.17	0	[]	1	3	0	0
RAP2C, member of RAS oncogene family, isoform CRA_b OS=Homo sapiens GN=RAP2C PE=1 SV=1	RAP2C	14 kDa	0.17	INF	[]	0	0	1	3
Tyrosine-protein kinase receptor OS=Homo sapiens GN=IGF1R PE=1 SV=1	IGF1R	155 kDa	0.77	0.6	[]	0	2	1	0
COP9 constitutive photomorphogenic-like protein subunit 4 isoform 1 (Fragment) OS=Homo sapiens GN=COPS4 PE=2 S\	COPS4	46 kDa	0.2	0	[]	3	1	0	0
Epididymis tissue sperm binding protein Li 14m OS=Homo sapiens PE=2 SV=1		83 kDa	0.2	0	[]	3	1	0	0
Eukaryotic translation elongation factor 1 beta 2, isoform CRA_a OS=Homo sapiens GN=EEF1B2 PE=3 SV=1	EEF1B2	25 kDa	0.2	0	[]	3	1	0	0
cDNA FLJ11140 fis, clone PLACE1006488, highly similar to Signal recognition particle 68 kDa protein OS=Homo sapiens PE=2 SV=1		41 kDa	0.11	0	[]	2	1	0	0
UDP-galactose-4-epimerase, isoform CRA_b OS=Homo sapiens GN=GALE PE=4 SV=1	GALE	26 kDa	0.0011	0	Fibro-BKO low, Fibro-Naive high	2	2	0	0
cDNA FLJ10711 fis, clone NT2RP3000917, highly similar to 5'-3' exoribonuclease 2 (EC 3.1.11.-) OS=Homo sapiens PE=2 SV=1		109 kDa	0.11	0	[]	2	1	0	0
Vacuolar protein sorting-associated protein 26A OS=Homo sapiens GN=VPS26A PE=1 SV=1	VPS26A	26 kDa	0.17	0	[]	1	3	0	0
Dimethylarginine dimethylaminohydrolase 1, isoform CRA_b OS=Homo sapiens GN=DDAH1 PE=2 SV=1	DDAH1	31 kDa	0.2	0	[]	3	1	0	0
Putative uncharacterized protein DKFZp686G02190 (Fragment) OS=Homo sapiens GN=DKFZp686G02190 PE=2 SV=1	DKFZp686G02190	49 kDa	0.011	INF	Fibro-BKO high, Fibro-Naive low	0	0	6	8
MUC5AC (Fragment) OS=Homo sapiens GN=MUC5AC PE=4 SV=1	MUC5AC	522 kDa	0.2	INF	[]	0	0	3	1
Hsp70-binding protein 1 (Fragment) OS=Homo sapiens GN=HSPBP1 PE=1 SV=1	HSPBP1	11 kDa	0.6	0.3	[]	3	0	0	1
IGL@ protein OS=Homo sapiens GN=IGL@ PE=2 SV=1	IGL	25 kDa	0.33	INF	[]	0	0	2	18
CLE7 OS=Homo sapiens GN=C14orf166 PE=2 SV=1	C14orf166	28 kDa	0.74	0.6	[]	2	0	1	0
cDNA FLJ58027 OS=Homo sapiens PE=2 SV=1		47 kDa	0.28	0	[]	5	1	0	0
Chromosome 7 open reading frame 24 OS=Homo sapiens GN=C7orf24 PE=4 SV=1	C7orf24	21 kDa	0.081	0	[]	1	2	0	0
cDNA, FLJ93744, highly similar to Homo sapiens keratin 6E (KRT6E), mRNA OS=Homo sapiens PE=2 SV=1		60 kDa	0.47	1.6	[]	2	3	2	5
Component of oligomeric golgi complex 4, isoform CRA_d OS=Homo sapiens GN=COG4 PE=4 SV=1	COG4	90 kDa	0.17	0	[]	1	3	0	0
cDNA FLJ51241, highly similar to Nidogen-1 OS=Homo sapiens PE=2 SV=1		79 kDa	0.42	INF	[]	0	0	0	2
THO complex subunit 1 OS=Homo sapiens GN=THOC1 PE=1 SV=1	THOC1	76 kDa	0.42	0	[]	0	4	0	0
BXDC2 protein (Fragment) OS=Homo sapiens GN=BXDC2 PE=2 SV=1	BXDC2	39 kDa	0.42	0	[]	5	0	0	0
26S protease regulatory subunit 6A OS=Homo sapiens GN=PSMC3 PE=1 SV=1	PSMC3	47 kDa	0.89	1	[]	10	6	7	8
H.sapiens ras-related Hrab1A protein OS=Homo sapiens GN=RAB1A PE=2 SV=1	RAB1A	23 kDa	0.94	1	[]	5	3	2	5
Junction adhesion molecule OS=Homo sapiens PE=2 SV=1		28 kDa	0.071	0.6	[]	7	9	4	5
Serine/threonine-protein phosphatase OS=Homo sapiens GN=PPP1CC PE=3 SV=1	PPP1CC	37 kDa	0.045	0.3	Fibro-BKO low, Fibro-Naive high	16	13	3	5
MHC class I antigen (Fragment) OS=Homo sapiens GN=HLA-B PE=3 SV=1	HLA-B	32 kDa	0.82	0.9	[]	7	12	4	11
Heat shock 70kDa protein 2, isoform CRA_a OS=Homo sapiens GN=HSPA2 PE=3 SV=1	HSPA2	70 kDa	0.78	1.1	[]	17	14	15	15
HLA class I antigen OS=Homo sapiens GN=HLA-C PE=3 SV=1	HLA-C	41 kDa	0.13	0.5	[]	11	15	4	8
RAP1A, member of RAS oncogene family OS=Homo sapiens GN=RAP1A PE=2 SV=1	RAP1A	21 kDa	0.54	1	[]	6	7	6	6
cDNA FLJ54752, highly similar to Poly(rC)-binding protein 2 OS=Homo sapiens PE=2 SV=1		30 kDa	0.36	0.5	[]	5	3	1	3
40S ribosomal protein S6 OS=Homo sapiens GN=RPS6 PE=1 SV=1	RPS6	25 kDa	0.35	0.4	[]	1	2	1	0
RAB5A, member RAS oncogene family, isoform CRA_a OS=Homo sapiens GN=RAB5A PE=4 SV=1	RAB5A	24 kDa	0.55	0.6	[]	2	2	2	0
cDNA FLJ57871, highly similar to Brix domain-containing protein 2 OS=Homo sapiens PE=2 SV=1		20 kDa	0.33	0.4	[]	2	1	0	1
Putative uncharacterized protein DKFZp686O12165 (Fragment) OS=Homo sapiens GN=DKFZp686O12165 PE=2 SV=1	DKFZp686O12165	241 kDa	0.0016	56	Fibro-BKO high, Fibro-Naive low	9	15	588	587
MHC class I antigen (Fragment) OS=Homo sapiens GN=HLA-C PE=3 SV=1	HLA-C	21 kDa	0.35	0.4	[]	6	9	0	6
Radixin OS=Homo sapiens GN=RDX PE=2 SV=1	RDX	69 kDa	0.75	0.7	[]	5	7	0	8
Histone H2A OS=Homo sapiens GN=HIST1H2AC PE=3 SV=1	HIST1H2AC	14 kDa	0.082	0.2	[]	42	26	6	5

Lipolysis-stimulated lipoprotein receptor (Fragment) OS=Homo sapiens GN=LSR PE=1 SV=1	LSR	12 kDa	0.64	1.6	[]	0	3	2	2
cDNA FLJ53308, highly similar to 2-oxoglutarate dehydrogenase E1 component, mitochondrial (EC 1.2.4.2) OS=Homo sapiens PE=2 SV=1		111 kDa	0.16	0.2	[]	2	4	1	0
Dynamin 2 isoform 4 variant (Fragment) OS=Homo sapiens PE=2 SV=1		55 kDa	0.081	0	[]	1	2	0	0
HCG24991, isoform CRA_a OS=Homo sapiens GN=hCG_24991 PE=4 SV=1	hCG_24991	23 kDa	0.0014	INF	Fibro-BKO high, Fibro-Naive low	0	0	1	1
MHC class I antigen (Fragment) OS=Homo sapiens GN=HLA-B PE=3 SV=1	HLA-B	38 kDa	0.71	1.5	[]	0	9	3	9
cDNA, FLJ93632, highly similar to Homo sapiens heterogeneous nuclear ribonucleoprotein R (HNRPR), mRNA OS=Homo sapiens PE=2 SV=1		71 kDa	0.027	0	Fibro-BKO low, Fibro-Naive high	2	3	0	0
RAB5B, member RAS oncogene family, isoform CRA_a OS=Homo sapiens GN=RAB5B PE=4 SV=1	RAB5B	24 kDa	0.05	0	Fibro-BKO low, Fibro-Naive high	3	2	0	0
Ephrin type-B receptor 2 OS=Homo sapiens GN=EPHB2 PE=1 SV=1	EPHB2	106 kDa	0.027	0	Fibro-BKO low, Fibro-Naive high	2	3	0	0
Laminin subunit alpha-4 OS=Homo sapiens GN=LAMA4 PE=1 SV=1	LAMA4	203 kDa	0.0019	INF	Fibro-BKO high, Fibro-Naive low	0	0	17	20
Laminin, alpha 4, isoform CRA_b OS=Homo sapiens GN=LAMA4 PE=4 SV=1	LAMA4	202 kDa	0.0001	INF	Fibro-BKO high, Fibro-Naive low	0	0	18	19
Cullin-4A OS=Homo sapiens GN=CUL4A PE=1 SV=1	CUL4A	78 kDa	0.05	0	Fibro-BKO low, Fibro-Naive high	3	2	0	0
Dipeptidyl peptidase 3 OS=Homo sapiens GN=DPP3 PE=1 SV=1	DPP3	84 kDa	0.0093	0	Fibro-BKO low, Fibro-Naive high	10	13	0	0
Importin subunit alpha OS=Homo sapiens GN=KPNA1 PE=2 SV=1	KPNA1	60 kDa	0.05	0	Fibro-BKO low, Fibro-Naive high	3	2	0	0
SFRS protein kinase 1, isoform CRA_e OS=Homo sapiens GN=SRPK1 PE=4 SV=1	SRPK1	62 kDa	0.2	0	[]	3	1	0	0
Full-length cDNA clone CS0DD006YL02 of Neuroblastoma of Homo sapiens (human) OS=Homo sapiens PE=2 SV=1		41 kDa	0.026	INF	Fibro-BKO high, Fibro-Naive low	0	0	14	21
Serine/arginine-rich-splicing factor 3 OS=Homo sapiens GN=SRSF3 PE=1 SV=1	SRSF3	10 kDa	0.0011	0	Fibro-BKO low, Fibro-Naive high	2	2	0	0
Septin-8 OS=Homo sapiens GN=SEPT8 PE=1 SV=1	Sep-08	49 kDa	0.2	0	[]	3	1	0	0
Thioredoxin reductase 1 OS=Homo sapiens GN=TXNRD1 PE=3 SV=1	TXNRD1	68 kDa	0.081	0	[]	1	2	0	0
Phosphoenolpyruvate carboxykinase [GTP], mitochondrial OS=Homo sapiens GN=PCK2 PE=1 SV=1	PCK2	48 kDa	0.081	0	[]	1	2	0	0
cDNA FLJ44170 fis, clone THYMU2035319, highly similar to RNA-binding region-containing protein 2 OS=Homo sapiens PE=2 SV=1		57 kDa	0.081	0	[]	1	2	0	0
cDNA FLJ61095, highly similar to Ubiquitin conjugation factor E4 A (Fragment) OS=Homo sapiens PE=2 SV=1		120 kDa	0.081	0	[]	1	2	0	0
cDNA FLJ52497, highly similar to Protein NDRG3 OS=Homo sapiens PE=2 SV=1		31 kDa	0.081	0	[]	1	2	0	0
ATP synthase subunit beta (Fragment) OS=Homo sapiens GN=ATP5B PE=1 SV=1	ATP5B	38 kDa	0.081	0	[]	1	2	0	0
Acidic leucine-rich nuclear phosphoprotein 32 family member A OS=Homo sapiens GN=ANP32A PE=1 SV=1	ANP32A	20 kDa	0.081	0	[]	1	2	0	0
Cytidine 5'-monophosphate N-acetylneuraminic acid synthetase variant (Fragment) OS=Homo sapiens PE=2 SV=1		48 kDa	0.081	0	[]	1	2	0	0
cDNA FLJ51951, highly similar to Squamous cell carcinoma antigen recognized by T-cells 3 (Fragment) OS=Homo sapiens PE=2 SV=1		74 kDa	0.081	0	[]	1	2	0	0
Testicular tissue protein Li 138 OS=Homo sapiens PE=2 SV=1		55 kDa	0.081	0	[]	1	2	0	0
Mal, T-cell differentiation protein 2, isoform CRA_a OS=Homo sapiens GN=MAL2 PE=4 SV=1	MAL2	19 kDa	0.081	0	[]	1	2	0	0
ESRP1/RAF1 fusion protein OS=Homo sapiens PE=2 SV=1		119 kDa	0.081	0	[]	1	2	0	0
SLC9A3R2 protein (Fragment) OS=Homo sapiens GN=SLC9A3R2 PE=2 SV=1	SLC9A3R2	41 kDa	0.66	2.3	[]	1	0	2	0
CKAP5 protein OS=Homo sapiens GN=CKAP5 PE=2 SV=1	CKAP5	107 kDa	0.081	0	[]	1	2	0	0
Anti-(ED-B) scFV (Fragment) OS=Homo sapiens PE=2 SV=2		25 kDa	0.17	INF	[]	0	0	1	3
Alpha-adducin OS=Homo sapiens GN=ADD1 PE=1 SV=1	ADD1	44 kDa	0.77	0.6	[]	0	2	1	0
Ribose-phosphate pyrophosphokinase 3 OS=Homo sapiens GN=PRPS1L1 PE=1 SV=1	PRPS1L1	35 kDa	0.079	INF	[]	0	0	1	2
cDNA, FLJ93928, highly similar to Homo sapiens PDZ domain containing ring finger 1 (PDZRN1), mRNA OS=Homo sapiens PE=2 SV=1		76 kDa	0.079	INF	[]	0	0	1	2
Short-chain dehydrogenase/reductase, isoform CRA_b OS=Homo sapiens GN=MGC4172 PE=3 SV=1	MGC4172	28 kDa	0.11	0	[]	2	1	0	0
F-box and leucine-rich repeat protein 18, isoform CRA_b OS=Homo sapiens GN=FBXL18 PE=4 SV=1	FBXL18	79 kDa	0.11	0	[]	2	1	0	0
Kinesin light chain 4, isoform CRA_a OS=Homo sapiens GN=KLC4 PE=4 SV=1	KLC4	69 kDa	0.11	0	[]	2	1	0	0
Golgi SNAP receptor complex member 1 OS=Homo sapiens PE=2 SV=1		29 kDa	0.11	0	[]	2	1	0	0
Uncharacterized protein OS=Homo sapiens PE=4 SV=1		51 kDa	0.11	0	[]	2	1	0	0
Protein FAM234A (Fragment) OS=Homo sapiens GN=FAM234A PE=1 SV=1	FAM234A	21 kDa	0.66	2.2	[]	0	1	0	2
Small nuclear ribonucleoprotein Sm D2 OS=Homo sapiens GN=SNRPD2 PE=1 SV=1	SNRPD2	9 kDa	0.11	0	[]	2	1	0	0
40S ribosomal protein S27 OS=Homo sapiens GN=RPS27 PE=1 SV=1	RPS27	7 kDa	0.11	0	[]	2	1	0	0
Epididymis secretory protein Li 100 OS=Homo sapiens GN=HEL-S-100 PE=2 SV=1	HEL-S-100	40 kDa	0.11	0	[]	2	1	0	0
Leucine rich repeat containing 57, isoform CRA_c OS=Homo sapiens GN=LRRC57 PE=4 SV=1	LRRC57	27 kDa	0.11	0	[]	2	1	0	0
Methyl-CpG-binding protein 2 OS=Homo sapiens GN=MECP2 PE=1 SV=1	MECP2	19 kDa	0.11	0	[]	2	1	0	0
cDNA FLJ53631, highly similar to Intercellular adhesion molecule 1 OS=Homo sapiens PE=2 SV=1		48 kDa	0.77	0.6	[]	0	2	1	0
Clusterin (Fragment) OS=Homo sapiens GN=CLU PE=1 SV=1	CLU	24 kDa	0.11	INF	[]	0	0	2	1
LIM and senescent cell antigen-like-containing domain protein OS=Homo sapiens GN=LIMS4 PE=1 SV=1	LIMS4	46 kDa	0.11	INF	[]	0	0	2	1
Eukaryotic translation initiation factor 2 beta OS=Homo sapiens GN=EIF2S2 PE=2 SV=1	EIF2S2	38 kDa	0.11	0	[]	2	1	0	0
Protein FAM3C (Fragment) OS=Homo sapiens GN=FAM3C PE=1 SV=1	FAM3C	16 kDa	0.11	0	[]	2	1	0	0
DNA helicase OS=Homo sapiens GN=MCM4 PE=3 SV=1	MCM4	97 kDa	0.11	0	[]	2	1	0	0
Serine/threonine-protein phosphatase OS=Homo sapiens GN=PPP4C PE=3 SV=1	PPP4C	35 kDa	0.2	0	[]	3	1	0	0
Aminoacylase-1 OS=Homo sapiens GN=ACY1 PE=3 SV=1	ACY1	42 kDa	0.081	0	[]	1	2	0	0
Pleckstrin homology domain containing, family C (With FERM domain) member 1, isoform CRA_b OS=Homo sapiens GN	PLEKHC1	78 kDa	0.079	INF	[]	0	0	1	2
Mitogen-activated protein kinase OS=Homo sapiens GN=MAPK13 PE=2 SV=1	MAPK13	42 kDa	0.11	0	[]	2	1	0	0
Processing of 1, ribonuclease P/MRP subunit (S. cerevisiae) OS=Homo sapiens GN=POP1 PE=2 SV=1	POP1	115 kDa	0.74	0.6	[]	0	2	0	1
Fermitin family homolog 3 (Fragment) OS=Homo sapiens GN=FERMT3 PE=1 SV=1	FERMT3	33 kDa	0.42	INF	[]	0	0	0	2
Serpin peptidase inhibitor, clade E (Nexin, plasminogen activator inhibitor type 1), member 1, isoform CRA_b OS=Homo	SERPINE1	45 kDa	0.42	INF	[]	0	0	0	2

Structural maintenance of chromosomes protein OS=Homo sapiens PE=2 SV=1		142 kDa	0.42	0	[]	0	3	0	0
cDNA FLJ13894 fis, clone THYRO1001671, highly similar to 59 kDa 2'-5'-oligoadenylate synthetase-like protein OS=Homo sapiens PE=2 SV=1		59 kDa	0.42	0	[]	0	3	0	0
E3 ubiquitin-protein ligase RNF213 OS=Homo sapiens GN=RNF213 PE=1 SV=1	RNF213	596 kDa	0.42	INF	[]	0	0	2	0
DEAD/H(Asp-Glu-Ala-Asp/His)box polypeptide 21 OS=Homo sapiens GN=OK/SW-cl.65 PE=2 SV=1	OK	16 kDa	0.42	0	[]	2	0	0	0
Rho GTPase activating protein OS=Homo sapiens PE=2 SV=1		75 kDa	0.42	0	[]	3	0	0	0
ADAM metallopeptidase domain 17 OS=Homo sapiens GN=ADAM17 PE=2 SV=1	ADAM17	93 kDa	0.42	INF	[]	0	0	3	0
Perilipin-3 (Fragment) OS=Homo sapiens GN=PLIN3 PE=1 SV=1	PLIN3	32 kDa	0.42	INF	[]	0	0	3	0
Endoglin (Osler-Rendu-Weber syndrome 1), isoform CRA_a OS=Homo sapiens GN=ENG PE=4 SV=1	ENG	71 kDa	0.42	INF	[]	0	0	0	2
Sex hormone-binding globulin OS=Homo sapiens GN=SHBG PE=1 SV=1	SHBG	37 kDa	0.42	0	[]	2	0	0	0
ATP-binding cassette sub-family F member 1 (Fragment) OS=Homo sapiens GN=ABCF1 PE=1 SV=1	ABCF1	68 kDa	0.42	0	[]	2	0	0	0
cDNA FLJ32365 fis, clone PUAEN1000161, highly similar to Homo sapiens CCR4-NOT transcription complex, subunit 1 (CNOT1), transcript va		120 kDa	0.42	0	[]	0	2	0	0
Dedicator of cytokinesis protein 9 OS=Homo sapiens GN=DOCK9 PE=1 SV=2	DOCK9	238 kDa	0.42	0	[]	0	2	0	0
Overexpressed in colon carcinoma 1 protein (Fragment) OS=Homo sapiens GN=C12orf75 PE=1 SV=2	C12orf75	9 kDa	0.42	INF	[]	0	0	0	2
Filamin A OS=Homo sapiens GN=FLNA PE=1 SV=1	FLNA	278 kDa	0.0067	8.3	Fibro-BKO high, Fibro-Naive low	3	10	47	47
cDNA FLJ11352 fis, clone HEMBA1000020, highly similar to Tubulin beta-2C chain OS=Homo sapiens PE=2 SV=1		45 kDa	0.45	0.8	[]	40	31	23	30
Aminopeptidase B OS=Homo sapiens GN=RNPEP PE=4 SV=1	RNPEP	73 kDa	0.038	0.08	Fibro-BKO low, Fibro-Naive high	6	9	1	0
JTV1 gene, isoform CRA_a OS=Homo sapiens GN=JTV1 PE=4 SV=1	JTV1	35 kDa	0.41	0.6	[]	8	4	4	2
Neuroblastoma RAS viral (V-ras) oncogene homolog OS=Homo sapiens GN=NRAS PE=2 SV=1	NRAS	21 kDa	0.0082	0.6	Fibro-BKO low, Fibro-Naive high	4	4	2	2
Actin-like protein (Fragment) OS=Homo sapiens GN=ACT PE=4 SV=1	ACT	11 kDa	0.25	1.9	[]	4	7	11	7
Aminopeptidase B OS=Homo sapiens GN=RNPEP PE=1 SV=1	RNPEP	68 kDa	0.083	0.1	[]	6	10	2	0
Putative uncharacterized protein DKFZp686L11144 (Fragment) OS=Homo sapiens GN=DKFZp686L11144 PE=2 SV=1	DKFZp686L11144	113 kDa	0.0012	48	Fibro-BKO high, Fibro-Naive low	7	16	485	489
FERM, RhoGEF and pleckstrin domain-containing protein 1 OS=Homo sapiens GN=FARP1 PE=1 SV=1	FARP1	88 kDa	0.51	1.8	[]	0	5	3	5
Basement membrane-specific heparan sulfate proteoglycan core protein variant (Fragment) OS=Homo sapiens PE=2 SV=1		246 kDa	0.022	7.9	Fibro-BKO high, Fibro-Naive low	0	48	151	176
Vacuolar protein sorting-associated protein VTA1 homolog OS=Homo sapiens GN=VTA1 PE=1 SV=1	VTA1	27 kDa	0.061	0.3	[]	4	3	1	1
Septin-2 (Fragment) OS=Homo sapiens GN=SEPT2 PE=1 SV=1	Sep-02	17 kDa	0.02	0.3	Fibro-BKO low, Fibro-Naive high	8	7	2	2
40S ribosomal protein S6 OS=Homo sapiens GN=RPS6 PE=2 SV=1	RPS6	29 kDa	0.081	0	[]	1	2	0	0
Ragulator complex protein LAMTOR1 OS=Homo sapiens GN=LAMTOR1 PE=1 SV=1	LAMTOR1	16 kDa	0.76	0.7	[]	2	1	0	2
Rab GDP dissociation inhibitor (Fragment) OS=Homo sapiens GN=GDI1 PE=2 SV=1	GDI1	51 kDa	0.0011	0	Fibro-BKO low, Fibro-Naive high	13	13	0	0
2-deoxyribose-5-phosphate aldolase homolog (C. elegans), isoform CRA_a OS=Homo sapiens GN=DERA PE=1 SV=1	DERA	25 kDa	0.027	0	Fibro-BKO low, Fibro-Naive high	4	6	0	0
cDNA FLJ60208, highly similar to Integrin alpha-4 OS=Homo sapiens PE=2 SV=1		40 kDa	0.011	INF	Fibro-BKO high, Fibro-Naive low	0	0	3	4
Synaptophysin-like protein 1 OS=Homo sapiens GN=SYPL1 PE=1 SV=1	SYPL1	25 kDa	0.081	0	[]	1	2	0	0
SCC-112 protein, isoform CRA_b OS=Homo sapiens GN=PDS5A PE=2 SV=1	PDS5A	151 kDa	0.0011	0	Fibro-BKO low, Fibro-Naive high	2	2	0	0
Lysyl oxidase-like 2 delta e13 OS=Homo sapiens GN=LOXL2 PE=2 SV=1	LOXL2	81 kDa	0.0048	INF	Fibro-BKO high, Fibro-Naive low	0	0	16	15
cDNA FLJ57752 OS=Homo sapiens PE=2 SV=1		19 kDa	0.2	0	[]	3	1	0	0
MS-D3 heavy chain variable region (Fragment) OS=Homo sapiens PE=2 SV=1		15 kDa	0.079	INF	[]	0	0	1	2
cDNA FLJ56668, highly similar to Nucleolar protein NOP5 OS=Homo sapiens PE=2 SV=1		16 kDa	0.081	0	[]	1	2	0	0
MHC class I antigen (Fragment) OS=Homo sapiens GN=HLA-B PE=3 SV=1	HLA-B	21 kDa	0.0014	INF	Fibro-BKO high, Fibro-Naive low	0	0	5	5
PP3856 OS=Homo sapiens PE=2 SV=1		15 kDa	0.0011	0	Fibro-BKO low, Fibro-Naive high	1	1	0	0
cDNA PSEC0016 fis, clone NT2RM1001076, highly similar to Procollagen-lysine,2-oxoglutarate 5-dioxygenase 3 (EC 1.14.11.4) OS=Homo sap		85 kDa	0.051	INF	[]	0	0	3	2
Peripheral plasma membrane protein CASK OS=Homo sapiens GN=CASK PE=1 SV=1	CASK	59 kDa	0.58	1.8	[]	0	2	2	1
cDNA FLJ14219 fis, clone NT2RP3003800, highly similar to Rattus norvegicus tyrosine protein kinase pp60-c-src mRNA OS=Homo sapiens PE		29 kDa	0.74	0.6	[]	0	2	0	1
cDNA FLJ53366, highly similar to Probable ATP-dependent RNA helicase DDX5 (EC 3.6.1.-) OS=Homo sapiens PE=2 SV=1		68 kDa	0.11	0	[]	4	2	0	0
Telomerase protein component 1 OS=Homo sapiens GN=TEP1 PE=1 SV=1	TEP1	167 kDa	0.49	0.4	[]	1	5	0	2
Haptoglobin OS=Homo sapiens GN=HP PE=1 SV=1	HP	38 kDa	0.17	INF	[]	0	0	1	3
cDNA FLJ55960, highly similar to Protein transport protein Sec23A OS=Homo sapiens PE=2 SV=1		74 kDa	0.42	0	[]	2	0	0	0
Ubiquitin-like 3, isoform CRA_a OS=Homo sapiens GN=UBL3 PE=4 SV=1	UBL3	13 kDa	0.42	0	[]	0	2	0	0
Paraspeckle component 1, isoform CRA_b OS=Homo sapiens GN=PSPC1 PE=4 SV=1	PSPC1	46 kDa	0.42	0	[]	2	0	0	0
cDNA, FLJ95208, highly similar to Homo sapiens PYD and CARD domain containing (PYCARD), transcript variant 1, mRNA OS=Homo sapiens		22 kDa	0.42	0	[]	0	2	0	0
Out at first protein homolog OS=Homo sapiens GN=OAF PE=1 SV=1	OAF	18 kDa	0.42	0	[]	2	0	0	0
Myosin-reactive immunoglobulin light chain variable region (Fragment) OS=Homo sapiens PE=2 SV=1		12 kDa	0.42	INF	[]	0	0	0	2
Apolipoprotein L, 1, isoform CRA_b OS=Homo sapiens GN=APOL1 PE=4 SV=1	APOL1	46 kDa	0.42	INF	[]	0	0	0	2
Solute carrier family 39 (Zinc transporter), member 14, isoform CRA_a (Fragment) OS=Homo sapiens GN=SLC39A14 PE=1	SLC39A14	53 kDa	0.42	INF	[]	0	0	2	0
C-type lectin domain family 3 member A OS=Homo sapiens GN=CLEC3A PE=1 SV=1	CLEC3A	23 kDa	0.42	0	[]	2	0	0	0
ATPase, H+ transporting, lysosomal 13kDa, V1 subunit G1, isoform CRA_a OS=Homo sapiens GN=ATP6V1G1 PE=4 SV=1	ATP6V1G1	14 kDa	0.42	0	[]	0	2	0	0
cDNA FLJ57597 OS=Homo sapiens PE=2 SV=1		89 kDa	0.42	0	[]	2	0	0	0
Mitochondrial ribosomal protein L49, isoform CRA_b OS=Homo sapiens GN=MRPL49 PE=4 SV=1	MRPL49	19 kDa	0.42	0	[]	2	0	0	0
Basic leucine zipper and W2 domains 2, isoform CRA_a OS=Homo sapiens GN=BZW2 PE=4 SV=1	BZW2	48 kDa	0.42	0	[]	0	2	0	0
1-phosphatidylinositol 4,5-bisphosphate phosphodiesterase delta-3 (Fragment) OS=Homo sapiens GN=PLCD3 PE=1 SV=1	PLCD3	12 kDa	0.42	INF	[]	0	0	2	0
Ig heavy chain variable region (Fragment) OS=Homo sapiens PE=2 SV=1		13 kDa	0.42	INF	[]	0	0	0	2

EF-hand domain family, member D2, isoform CRA_a OS=Homo sapiens GN=EFHD2 PE=2 SV=1	EFHD2	27 kDa	0.42	0	[]	2	0	0	0
Tyrosine--tRNA ligase (Fragment) OS=Homo sapiens GN=YARS PE=2 SV=1	YARS	59 kDa	0.42	0	[]	2	0	0	0
cDNA FLJ51537, highly similar to Myosin heavy chain, skeletal muscle, adult 2 (Fragment) OS=Homo sapiens PE=2 SV=1		96 kDa	0.42	INF	[]	0	0	2	0
Receptor-type tyrosine-protein phosphatase kappa OS=Homo sapiens GN=PTPRK PE=1 SV=1	PTPRK	166 kDa	0.016	3	Fibro-BKO high, Fibro-Naive low	4	4	11	10
Mutant receptor type protein tyrosine phosphatase K OS=Homo sapiens GN=PTPRK PE=2 SV=1	PTPRK	162 kDa	0.016	3	Fibro-BKO high, Fibro-Naive low	4	4	11	10
Tubulin beta chain OS=Homo sapiens PE=2 SV=1		47 kDa	0.35	0.8	[]	75	63	43	58
Filamin-A OS=Homo sapiens GN=FLNA PE=1 SV=1	FLNA	277 kDa	0.0059	8.2	Fibro-BKO high, Fibro-Naive low	3	10	46	47
Testicular secretory protein Li 42 OS=Homo sapiens PE=2 SV=1		49 kDa	0.75	1.1	[]	9	5	7	7
Catenin (Cadherin-associated protein), alpha 1, 102kDa, isoform CRA_a OS=Homo sapiens GN=CTNNA1 PE=1 SV=1	CTNNA1	93 kDa	0.11	1.3	[]	45	57	55	65
MHC class I antigen (Fragment) OS=Homo sapiens GN=HLA-A PE=3 SV=1	HLA-A	36 kDa	0.43	0.6	[]	14	19	4	15
cDNA FLJ53427, highly similar to Galectin-3-binding protein OS=Homo sapiens PE=2 SV=1		52 kDa	0.27	1.2	[]	66	65	70	64
Filamin-B OS=Homo sapiens GN=FLNB PE=1 SV=1	FLNB	256 kDa	0.23	0.3	[]	10	26	4	5
cDNA FLJ53214, highly similar to Tubulointerstitial nephritis antigen-like OS=Homo sapiens PE=2 SV=1		44 kDa	0.85	1.1	[]	14	6	10	9
cDNA FLJ50996, highly similar to 60S ribosomal protein L4 OS=Homo sapiens PE=2 SV=1		46 kDa	0.041	0.4	Fibro-BKO low, Fibro-Naive high	14	13	6	4
cDNA FLJ58784, highly similar to T-complex protein 1 subunit epsilon OS=Homo sapiens PE=2 SV=1		57 kDa	0.0083	0.5	Fibro-BKO low, Fibro-Naive high	26	27	12	12
Erbin OS=Homo sapiens GN=ERBIN PE=1 SV=1	ERBIN	69 kDa	0.85	1.1	[]	1	6	3	4
Harvey rat sarcoma viral oncoprotein isoform A (Fragment) OS=Homo sapiens GN=HRAS PE=2 SV=1	HRAS	21 kDa	0.0082	0.6	Fibro-BKO low, Fibro-Naive high	4	4	2	2
cDNA FLJ55458, highly similar to Programmed cell death 6-interacting protein OS=Homo sapiens PE=2 SV=1		76 kDa	0.28	1.2	[]	37	48	44	43
Aminoacyl tRNA synthase complex-interacting multifunctional protein 2 OS=Homo sapiens GN=AIMP2 PE=1 SV=2	AIMP2	27 kDa	0.18	0.4	[]	8	5	3	1
V-Ki-ras2 Kirsten rat sarcoma viral oncogene homolog, isoform CRA_b OS=Homo sapiens GN=KRAS PE=1 SV=1	KRAS	21 kDa	0.74	0.7	[]	3	3	0	4
Tubulin beta chain OS=Homo sapiens GN=TUBB6 PE=2 SV=1	TUBB6	50 kDa	0.15	0.7	[]	34	32	17	25
MHC class I antigen (Fragment) OS=Homo sapiens GN=HLA B PE=3 SV=1	HLA	32 kDa	0.37	0.7	[]	6	8	3	6
MHC class I antigen (Fragment) OS=Homo sapiens GN=HLA-A PE=4 SV=2	HLA-A	23 kDa	0.35	0.7	[]	11	12	4	10
HCG1985580, isoform CRA_c OS=Homo sapiens GN=PDCD6 PE=1 SV=1	PDCD6	14 kDa	0.89	1	[]	8	7	7	6
MHC class I antigen (Fragment) OS=Homo sapiens GN=HLA-A PE=3 SV=1	HLA-A	38 kDa	0.74	0.8	[]	11	13	4	14
Elongation factor 1-alpha 1 OS=Homo sapiens GN=PTI-1 PE=2 SV=1	PTI-1	43 kDa	0.12	1.6	[]	10	7	11	12
cDNA FLJ58476, highly similar to Poly(rC)-binding protein 2 OS=Homo sapiens PE=2 SV=1		32 kDa	0.38	0.5	[]	5	2	1	2
UBE2L3/KRAS fusion protein OS=Homo sapiens PE=2 SV=1		34 kDa	0.49	0.5	[]	3	3	0	3
Tubulin beta chain OS=Homo sapiens GN=TUBB8 PE=1 SV=1	TUBB8	46 kDa	0.92	1	[]	19	12	11	16
PLXNB2 protein (Fragment) OS=Homo sapiens GN=PLXNB2 PE=2 SV=1	PLXNB2	32 kDa	0.39	1.3	[]	4	6	6	5
ERBB2IP protein OS=Homo sapiens GN=ERBB2IP PE=2 SV=1	ERBB2IP	154 kDa	0.99	1	[]	1	6	3	3
Sodium/potassium-transporting ATPase subunit alpha OS=Homo sapiens PE=2 SV=1		108 kDa	0.23	0.9	[]	14	16	12	11
Putative uncharacterized protein DKFZp686O1117 OS=Homo sapiens GN=DKFZp686O1117 PE=2 SV=1	DKFZp686O1117	83 kDa	0.0033	0	Fibro-BKO low, Fibro-Naive high	10	12	0	0
MHC class I antigen (Fragment) OS=Homo sapiens GN=HLA-B PE=3 SV=1	HLA-B	32 kDa	0.94	1.1	[]	3	6	0	9
MHC class I antigen (Fragment) OS=Homo sapiens GN=HLA-B PE=3 SV=1	HLA-B	38 kDa	0.82	0.8	[]	5	9	0	10
SEC13-like 1 isoform b variant (Fragment) OS=Homo sapiens PE=2 SV=1		36 kDa	0.77	0.7	[]	1	2	0	2
Tumor susceptibility gene 101, isoform CRA_a OS=Homo sapiens GN=TSG101 PE=4 SV=1	TSG101	29 kDa	0.23	0.6	[]	4	4	1	3
Urokinase plasminogen activator surface receptor (Fragment) OS=Homo sapiens GN=PLAUR PE=1 SV=1	PLAUR	25 kDa	0.37	2.2	[]	1	1	1	3
Heat shock cognate 71 kDa protein OS=Homo sapiens GN=HSPA8 PE=1 SV=1	HSPA8	45 kDa	0.21	0.8	[]	27	33	23	22
Cullin 4B, isoform CRA_e OS=Homo sapiens GN=CUL4B PE=1 SV=1	CUL4B	103 kDa	0.2	0	[]	3	1	0	0
Procollagen-lysine,2-oxoglutarate 5-dioxygenase 3 (Fragment) OS=Homo sapiens GN=PLOD3 PE=1 SV=1	PLOD3	31 kDa	0.051	INF	[]	0	0	3	2
cDNA FLJ51409, highly similar to Thrombospondin-4 OS=Homo sapiens PE=2 SV=1		96 kDa	0.11	INF	[]	0	0	2	1
Synaptophysin-like 1, isoform CRA_a OS=Homo sapiens GN=SYPL PE=4 SV=1	SYPL	29 kDa	0.42	0	[]	0	2	0	0
Reticulon OS=Homo sapiens GN=NOGOC PE=2 SV=1	NOGOC	22 kDa	0.6	1.6	[]	6	0	4	5
Collagen, type XII, alpha 1 OS=Homo sapiens GN=COL12A1 PE=2 SV=1	COL12A1	206 kDa	0.0015	INF	Fibro-BKO high, Fibro-Naive low	0	0	55	64
Keratin, type I cytoskeletal 19 (Fragment) OS=Homo sapiens GN=KRT19 PE=1 SV=1	KRT19	21 kDa	0.0011	0	Fibro-BKO low, Fibro-Naive high	3	3	0	0
EEF1A protein (Fragment) OS=Homo sapiens GN=EEF1A PE=2 SV=1	EEF1A	24 kDa	0.4	0.5	[]	6	6	5	0
MHC class I antigen (Fragment) OS=Homo sapiens GN=HLA-B PE=3 SV=1	HLA-B	21 kDa	0.017	0	Fibro-BKO low, Fibro-Naive high	5	7	0	0
cDNA FLJ51269, highly similar to Aminopeptidase N (EC 3.4.11.2) OS=Homo sapiens PE=2 SV=1		56 kDa	0.013	INF	Fibro-BKO high, Fibro-Naive low	0	0	11	15
MHC class I antigen (Fragment) OS=Homo sapiens GN=HLA-B PE=3 SV=1	HLA-B	21 kDa	0.94	1.1	[]	0	3	0	3
MHC class I antigen (Fragment) OS=Homo sapiens GN=HLA-A PE=3 SV=1	HLA-A	21 kDa	0.35	0.4	[]	8	12	0	8
cDNA FLJ51311 OS=Homo sapiens PE=2 SV=1		10 kDa	0.31	0.4	[]	1	2	0	1
GCT-A9 light chain variable region (Fragment) OS=Homo sapiens PE=2 SV=1		12 kDa	0.17	INF	[]	0	0	1	3
Lipolysis-stimulated lipoprotein receptor (Fragment) OS=Homo sapiens GN=LSR PE=1 SV=3	LSR	15 kDa	0.42	0	[]	4	0	0	0
Haptoglobin-related protein OS=Homo sapiens GN=HPR PE=1 SV=1	HPR	13 kDa	0.42	INF	[]	0	0	0	3
A30 (Fragment) OS=Homo sapiens PE=4 SV=1		10 kDa	0.079	INF	[]	0	0	1	2
Putative uncharacterized protein DKFZp564O1822 (Fragment) OS=Homo sapiens GN=DKFZp564O1822 PE=2 SV=1	DKFZp564O1822	42 kDa	0.42	INF	[]	0	0	0	2
Signal transducer and activator of transcription 1-alpha/beta OS=Homo sapiens GN=STAT1 PE=1 SV=1	STAT1	23 kDa	0.64	2.4	[]	0	1	2	0
Non-muscle myosin heavy chain (Fragment) OS=Homo sapiens PE=2 SV=1		24 kDa	0.42	0	[]	0	6	0	0

SFRS protein kinase 2, isoform CRA_b OS=Homo sapiens GN=SRPK2 PE=4 SV=1	SRPK2	78 kDa	0.42	0	[]	0	1	0	0
EEF1A1 protein (Fragment) OS=Homo sapiens GN=EEF1A1 PE=2 SV=1	EEF1A1	17 kDa	0.42	0	[]	0	1	0	0
26S proteasome non-ATPase regulatory subunit 6 (Fragment) OS=Homo sapiens GN=PSMD6 PE=1 SV=1	PSMD6	17 kDa	0.42	0	[]	0	2	0	0
HLA class I histocompatibility antigen, alpha chain G OS=Homo sapiens GN=HLA-G PE=1 SV=1	HLA-G	28 kDa	0.42	0	[]	0	3	0	0
Protein 4.1 (Fragment) OS=Homo sapiens GN=EPB41 PE=1 SV=1	EPB41	55 kDa	0.42	0	[]	0	4	0	0
cDNA FLJ76209, highly similar to Homo sapiens inorganic pyrophosphatase 2 (PPA2), transcript variant 2, mRNA OS=Homo sapiens PE=2 SV=		32 kDa	0.42	0	[]	2	0	0	0
cDNA FLJ78437, highly similar to Homo sapiens cartilage oligomeric matrix protein (COMP), mRNA OS=Homo sapiens PE=2 SV=1		83 kDa	0.42	INF	[]	0	0	3	0
Importin subunit alpha OS=Homo sapiens PE=2 SV=1		60 kDa	0.42	0	[]	0	2	0	0
2-oxoglutarate dehydrogenase, mitochondrial OS=Homo sapiens GN=OGDH PE=1 SV=1	OGDH	113 kDa	0.17	0	[]	1	3	0	0
Ig heavy chain variable region (Fragment) OS=Homo sapiens PE=2 SV=1		14 kDa	0.42	INF	[]	0	0	0	2
ABC transporter MOAT-B isoform (Fragment) OS=Homo sapiens GN=MOAT-B PE=2 SV=1	MOAT-B	100 kDa	0.42	0	[]	0	2	0	0
G gamma globin chain OS=Homo sapiens GN=HBG2 PE=3 SV=1	HBG2	16 kDa	0.42	0	[]	2	0	0	0
MHC class I antigen (Fragment) OS=Homo sapiens GN=HLA-C PE=3 SV=1	HLA-C	21 kDa	0.42	INF	[]	0	0	2	0
cDNA FLJ42166 fis, clone THYMU2019210, highly similar to HLA class I histocompatibility antigen, B-40 alpha chain OS=Homo sapiens PE=2		48 kDa	0.76	1.7	[]	0	6	0	9
cDNA FLJ77042, highly similar to Homo sapiens ATP-binding cassette, sub-family C (CFTR/MRP), member 4, mRNA OS=Homo sapiens PE=2 S		97 kDa	0.42	0	[]	0	1	0	0
MHC class I antigen (Fragment) OS=Homo sapiens GN=HLA-C PE=3 SV=1	HLA-C	21 kDa	0.42	0	[]	0	4	0	0
cDNA FLJ52936, weakly similar to Tropomyosin alpha-4 chain OS=Homo sapiens PE=2 SV=1		18 kDa	0.42	INF	[]	0	0	1	0
MHC class I antigen (Fragment) OS=Homo sapiens GN=HLA-C PE=3 SV=1	HLA-C	21 kDa	0.42	0	[]	0	4	0	0
Rho-associated protein kinase 1 OS=Homo sapiens GN=ROCK1 PE=1 SV=1	ROCK1	133 kDa	1	INF	[]	0	0	0	0
cDNA FLJ54081, highly similar to Keratin, type II cytoskeletal 5 OS=Homo sapiens PE=2 SV=1		59 kDa	0.42	INF	[]	0	0	0	3
Truncated MHC class I antigen (Fragment) OS=Homo sapiens GN=HLA-B PE=4 SV=1	HLA-B	13 kDa	0.42	INF	[]	0	0	1	0
MHC class I antigen (Fragment) OS=Homo sapiens GN=HLA-C PE=3 SV=1	HLA-C	21 kDa	0.42	0	[]	3	0	0	0
Immunglobulin heavy chain variable region (Fragment) OS=Homo sapiens PE=4 SV=1		15 kDa	0.42	INF	[]	0	0	0	1
Actin-like protein (Fragment) OS=Homo sapiens GN=ACT PE=4 SV=1	ACT	12 kDa	0.42	INF	[]	0	0	4	0
cDNA FLJ52207, highly similar to HLA class I histocompatibility antigen, B-7 alpha chain OS=Homo sapiens PE=2 SV=1		23 kDa	0.42	0	[]	0	2	0	0
Disco-interacting protein 2 homolog C OS=Homo sapiens GN=DIP2C PE=1 SV=1	DIP2C	178 kDa	0.42	INF	[]	0	0	1	0
MHC class I antigen (Fragment) OS=Homo sapiens GN=HLA-B PE=3 SV=1	HLA-B	21 kDa	0.42	INF	[]	0	0	0	5
RAB6B protein OS=Homo sapiens GN=RAB6B PE=2 SV=1	RAB6B	23 kDa	1	INF	[]	0	0	0	0
Serine/threonine-protein kinase 26 OS=Homo sapiens GN=STK26 PE=1 SV=1	STK26	49 kDa	1	INF	[]	0	0	0	0
