# Supplementary material for: Exosomes isolated from cancer patients’ sera transfer malignant traits and confer the same phenotype of primary tumors to oncosuppressor-mutated cells
Source: J Exp Clin Cancer Res. 2017 Aug 30;36:113. doi: 10.1186/s13046-017-0587-0 (PMC5577828; doi:10.1186/s13046-017-0587-0)
Supplement: Supplementary file 6 — Exosome proteins involved in microvesicles uptake (Ligands for the receptors determined by MS on fibroblasts plasma membrane)*. (PDF 216 kb) [file 13046_2017_587_MOESM6_ESM.pdf]

Supplementary Table 6. Exosome proteins involved in microvesicles uptake (Ligands for the receptors determined by MS on fibroblasts plasma membrane)*

Identified Proteins	Alternate Name	MW	T-Test (p-value)	Fold Change	Quantitative Profile	Control		BRCA1-KO		Reported in Exocarta
						1	2	1	2	
Laminin, gamma 1 (Formerly LAMB2), isoform CRA_a OS=Homo sapiens GN=LAMC1 PE=4 SV=1	LAMC1	174 kDa	0.0047	67	Fibro-BKO high, Fibro-Naive low	0	1	**26**	**32**	yes
Laminin subunit beta-1 OS=Homo sapiens GN=LAMB1 PE=1 SV=1	LAMB1	200 kDa	0.0083	INF	Fibro-BKO high, Fibro-Naive low	0	0	**17**	**22**	yes
Laminin, beta 2 (Laminin S), isoform CRA_a OS=Homo sapiens GN=LAMB2 PE=4 SV=1	LAMB2	196 kDa	0.0014	INF	Fibro-BKO high, Fibro-Naive low	0	0	**5**	**6**	yes
Laminin subunit alpha-2 OS=Homo sapiens GN=LAMA2 PE=1 SV=1	LAMA2	344 kDa	0.079	INF	[]	0	0	1	2	no
Laminin subunit alpha-4 OS=Homo sapiens GN=LAMA4 PE=1 SV=1	LAMA4	203 kDa	0.0019	INF	Fibro-BKO high, Fibro-Naive low	0	0	**17**	**20**	yes
Laminin, alpha 4, isoform CRA_b OS=Homo sapiens GN=LAMA4 PE=4 SV=1	LAMA4	202 kDa	0.0001	INF	Fibro-BKO high, Fibro-Naive low	0	0	**18**	**19**	yes
Thrombospondin 1, isoform CRA_a OS=Homo sapiens GN=THBS1 PE=4 SV=1	THBS1	129 kDa	0.0076	21	Fibro-BKO high, Fibro-Naive low	4	10	**130**	**119**	yes
cDNA FLJ51409, highly similar to Thrombospondin-4 OS=Homo sapiens PE=2 SV=1		96 kDa	0.11	INF	[]	0	0	2	1	yes
Fibronectin 1, isoform CRA_n OS=Homo sapiens GN=FN1 PE=4 SV=1	FN1	259 kDa	0.001	44	Fibro-BKO high, Fibro-Naive low	11	25	**676**	**688**	yes
Fibronectin (Fragment) OS=Homo sapiens GN=FN1 PE=1 SV=1	FN1	121 kDa	0.0012	52	Fibro-BKO high, Fibro-Naive low	7	14	**480**	**484**	yes
Collagen alpha-1(VI) chain OS=Homo sapiens GN=COL6A1 PE=1 SV=1	COL6A1	108 kDa	0.015	14	Fibro-BKO high, Fibro-Naive low	3	3	**41**	**35**	yes
Collagen alpha-1(XII) chain OS=Homo sapiens GN=COL12A1 PE=1 SV=1	COL12A1	333 kDa	0.00087	INF	Fibro-BKO high, Fibro-Naive low	0	0	**84**	**96**	yes
Collagen alpha-2(I) chain OS=Homo sapiens GN=COL1A2 PE=1 SV=1	COL1A2	129 kDa	0.0016	INF	Fibro-BKO high, Fibro-Naive low	0	0	**30**	**35**	yes
Collagen, type VI, alpha 3 OS=Homo sapiens GN=COL6A3 PE=4 SV=1	COL6A3	344 kDa	0.00011	97	Fibro-BKO high, Fibro-Naive low	0	5	**200**	**213**	yes
Collagen, type I, alpha 1, isoform CRA_a OS=Homo sapiens GN=COL1A1 PE=4 SV=1	COL1A1	85 kDa	0.0029	INF	Fibro-BKO high, Fibro-Naive low	0	0	**25**	**30**	yes
Collagen alpha-1(I) chain preproprotein OS=Homo sapiens GN=COL1A1 PE=2 SV=1	COL1A1	100 kDa	0.021	INF	Fibro-BKO high, Fibro-Naive low	0	0	**22**	**32**	yes
Collagen, type XVIII, alpha 1, isoform CRA_d OS=Homo sapiens GN=COL18A1 PE=4 SV=1	COL18A1	136 kDa	0.29	3.1	[]	0	4	5	11	yes
Procollagen C-endopeptidase enhancer OS=Homo sapiens GN=PCOLCE PE=4 SV=1	PCOLCE	48 kDa	0.026	INF	Fibro-BKO high, Fibro-Naive low	0	0	**4**	**6**	yes
Collagen alpha-2(VI) chain (Fragment) OS=Homo sapiens GN=COL6A2 PE=1 SV=1	COL6A2	20 kDa	0.021	INF	Fibro-BKO high, Fibro-Naive low	0	0	**5**	**4**	yes
COL6A2 protein OS=Homo sapiens PE=2 SV=1		47 kDa	0.0025	33	Fibro-BKO high, Fibro-Naive low	0	1	**14**	**14**	yes
COL6A3 protein OS=Homo sapiens GN=COL6A3 PE=2 SV=1	COL6A3	278 kDa	0.0003	84	Fibro-BKO high, Fibro-Naive low	0	5	**175**	**184**	yes
Collagen alpha-2(VI) chain (Fragment) OS=Homo sapiens GN=COL6A2 PE=1 SV=1	COL6A2	26 kDa	0.051	INF	[]	0	0	3	2	yes
Procollagen-lysine 1, 2-oxoglutarate 5-dioxygenase 1, isoform CRA_a OS=Homo sapiens GN=PLOD1 PE=	PLOD1	84 kDa	0.57	3.4	[]	1	0	3	0	yes
Collagen, type XII, alpha 1 OS=Homo sapiens GN=COL12A1 PE=2 SV=1	COL12A1	206 kDa	0.0015	INF	Fibro-BKO high, Fibro-Naive low	0	0	**55**	**64**	yes
Milk fat globule-EGF factor 8 protein, isoform CRA_a OS=Homo sapiens GN=MFGE8 PE=4 SV=1	MFGE8	43 kDa	0.00013	6.5	Fibro-BKO high, Fibro-Naive low	12	13	67	74	yes
Protocadherin Fat 1 OS=Homo sapiens GN=FAT1 PE=1 SV=1	FAT1	506 kDa	0.0067	9.4	Fibro-BKO high, Fibro-Naive low	2	6	**30**	**33**	yes
Cadherin-1 OS=Homo sapiens GN=CDH1 PE=1 SV=1	CDH1	100 kDa	0.77	0.9	[]	10	8	6	9	yes
cDNA FLJ77464, highly similar to Homo sapiens protocadherin 1 (cadherin-like 1) (PCDH1), transcript var		115 kDa	0.042	2.9	Fibro-BKO high, Fibro-Naive low	3	2	**7**	**6**	yes
Tenascin C (Hexabrachion), isoform CRA_a OS=Homo sapiens GN=TNC PE=4 SV=1	TNC	241 kDa	0.00028	INF	Fibro-BKO high, Fibro-Naive low	0	0	**44**	**49**	yes
Tenascin OS=Homo sapiens GN=TNC PE=1 SV=1	TNC	221 kDa	0.00024	INF	Fibro-BKO high, Fibro-Naive low	0	0	**45**	**50**	yes
Gamma filamin variant (Fragment) OS=Homo sapiens PE=2 SV=1		143 kDa	0.0015	27	Fibro-BKO high, Fibro-Naive low	1	0	**12**	**13**	yes
Filamin B, beta (Actin binding protein 278), isoform CRA_a OS=Homo sapiens GN=FLNB PE=4 SV=1	FLNB	280 kDa	0.23	0.3	[]	12	29	6	6	yes
Filamin A OS=Homo sapiens GN=FLNA PE=1 SV=1	FLNA	278 kDa	0.0067	8.3	Fibro-BKO high, Fibro-Naive low	3	10	**47**	**47**	yes
Filamin-A OS=Homo sapiens GN=FLNA PE=1 SV=1	FLNA	277 kDa	0.0059	8.2	Fibro-BKO high, Fibro-Naive low	3	10	**46**	**47**	yes
Filamin-B OS=Homo sapiens GN=FLNB PE=1 SV=1	FLNB	256 kDa	0.23	0.3	[]	10	26	4	5	yes
Integrin beta-1 (Fragment) OS=Homo sapiens GN=ITGB1 PE=1 SV=1	ITGB1	11 kDa	0.3	1.6	[]	5	9	11	8	yes
Integrin alpha-2 OS=Homo sapiens GN=ITGA2 PE=1 SV=1	ITGA2	103 kDa	0.98	1	[]	18	35	22	25	yes
Integrin beta-1 (Fragment) OS=Homo sapiens GN=ITGB1 PE=1 SV=1	ITGB1	11 kDa	0.3	1.6	[]	5	9	11	8	yes
Collagen, type I, alpha 1, isoform CRA_a OS=Homo sapiens GN=COL1A1 PE=4 SV=1	COL1A1	85 kDa	0.0029	INF	Fibro-BKO high, Fibro-Naive low	0	0	**25**	**30**	yes
Heparan sulfate proteoglycan 2 (Perlecan), isoform CRA_b OS=Homo sapiens GN=HSPG2 PE=4 SV=1	HSPG2	464 kDa	0.015	9.2	Fibro-BKO high, Fibro-Naive low	1	76	**283**	**329**	yes
Nectin-2 (Fragment) OS=Homo sapiens GN=NECTIN2 PE=1 SV=1	NECTIN2	23 kDa	0.081	2.2	[]	2	1	3	3	no

*Based on:

Kelly J. McKelvey et al., 2015. Exosomes: Mechanisms of Uptake. J Circ Biomark, 4:7.

Laura Ann Mulcahy et al., 2014. Routes and mechanisms of extracellular vesicle uptake. Journal of Extracellular Vesicles 3: 24641
